# Global Prevalence of Sleep Bruxism and Awake Bruxism in Pediatric and Adult Populations: A Systematic Review and Meta-Analysis

**DOI:** 10.3390/jcm13144259

**Published:** 2024-07-22

**Authors:** Grzegorz Zieliński, Agnieszka Pająk, Marcin Wójcicki

**Affiliations:** 1 Department of Sports Medicine, Medical University of Lublin, 20-093 Lublin, Poland; 2 Clinic of Anaesthesiology and Paediatric Intensive Care, Medical University of Lublin, Gebali Str. 6, 20-093 Lublin, Poland; 3 Independent Unit of Functional Masticatory Disorder, Medical University of Lublin, 20-093 Lublin, Poland

**Keywords:** prevalence, epidemiology, population, nocturnal bruxism, wakefulness bruxism, Europe, Asia, America, Africa, Australia

## Abstract

**Background/Objectives**: The purpose of this systematic review was to assess the global prevalence of sleep bruxism and awake bruxism in pediatric and adult populations. **Methods**: This systematic review was conducted by analyzing studies published from 2003 to 2023. The following keyword combination was utilized: prevalence, epidemiology, population, and bruxism. The PubMed database was analyzed, supplemented by manual searches using the Google search. Additionally, the snowballing procedure method was applied. A double assessment of the quality of publications was carried out to preserve the highest possible quality of evidence (e.g., Joanna Briggs Institute critical appraisal checklist). Analyses were conducted using the R statistical language. **Results**: The global bruxism (sleep and awake) prevalence is 22.22%. The global sleep bruxism prevalence is 21% and awake prevalence is 23%. The occurrence of sleep bruxism, based on polysomnography, was estimated at 43%. The highest prevalence of sleep bruxism was observed in North America at 31%, followed by South America at 23%, Europe at 21%, and Asia at 19%. The prevalence of awake bruxism was highest in South America at 30%, followed by Asia at 25% and Europe at 18%. **Conclusions**: One in four individuals may experience awake bruxism. Bruxism is a significant factor among women. It was observed that age is a significant factor for the occurrence of sleep bruxism in women. Among the limitations of the study is the lack of analysis of the prevalence of bruxism in Africa and Australia due to not collecting an adequate sample for analysis. The study was registered in the Open Science Framework (10.17605/OSF.IO/ZE786).

## 1. Introduction

The term “bruxism” was introduced by Frohman in 1931 [1]. However, earlier attempts to name the reflex of teeth grinding were made by Pietkiewicz (1907), who described it as “la bruxomanie” [2]. Yet, these dates are not the beginnings of research into the reflex of teeth grinding. The first mentions of this habit appeared in scientific literature in 1860 [3], and descriptions of teeth grinding are present in the Psalms of David, dated before our era [4].

Early observations of patients with bruxism emphasized that it is an unconscious habit (1940, Boyens) and that it affects a significant percentage of the population across various age groups (1957, Nadler) [5,6]. Throughout the 20th and 21st centuries, with rapid developments in dental research, bruxism began to be differentiated [7,8]. Miller, in 1950, proposed distinguishing between daytime and nighttime bruxism [9], and in 1983, a distinction was made between eccentric and centric bruxism [4,10]. In the context of limiting tooth wear, Okeson in 1987 proposed the use of occlusal splints [11]. In 2013, Lobbezoo et al. in a consensus on the definition and classification of bruxism emphasized its two manifestations: during sleep and during wakefulness [12].

Over the years, bruxism was described as a parafunction associated with multifactorial etiology, but primarily with a psychological factor. Therefore, a groundbreaking study was Every’s work in 1960, in which he proposed an evolutionary explanation for bruxism, suggesting that teeth grinding is associated with sharpening teeth to use them as tools or weapons [13]. In 1996, a study by Lavigne et al. opened the era of polysomnographic diagnosis research for bruxism. In the diagnostic context of the 21st century, two tools were published that will undoubtedly influence further research in terms of the described phenomenon [14]. This includes the “BruxApp” smartphone app presented in 2015, allowing for self-reporting of bruxism during the day, created by Bracci et al. [15,16], and the “BruxScreen” tool, created in 2023 [17]. It consists of a questionnaire part filled out by the examinee and a clinical assessment form filled out by the specialist [17].

In terms of key works concerning the etiology of bruxism, a study published in 2001 by Lobbezoo and Naeije is significant [18]. The researchers stated that certain neurotransmitters in the central nervous system might influence the modulation of bruxism [18]. In 2018, a team led by Lobbezoo et al. revised the 2013 consensus [19]. In their work, they revised the definitions of bruxism: “(i) sleep and awake bruxism are masticatory muscle activities that occur during sleep (characterised as rhythmic or non-rhythmic) and wakefulness (characterised by repetitive or sustained tooth contact and/or by bracing or thrusting of the mandible), respectively”; and, among other things, noted that in healthy individuals, bruxism should not be considered a disorder: “(ii) in otherwise healthy individuals, bruxism should not be considered as a disorder, but rather as a behaviour that can be a risk (and/or protective) factor for certain clinical consequences” [19].

The etiology of bruxism is multifactorial and is not fully understood [18,20,21], which is why it garners significant interest from researchers. As described in the historical overview above, the etiology of bruxism was linked to various factors. Every suggested it is related to teeth sharpening [13]. Other suggestions include that the alternating movement is related to maintaining airway patency during sleep [12,22,23]. Bracha indicates that clenching teeth during moments of stress increases blood flow to the frontal lobe, which potentially helps in the fight or flight response [24,25]. Manfredini et al. (2020) suggest that the etiology of bruxism is mainly related to central factors, not peripheral [26]. Giovanni and Giorgia in their work note that bruxism stimulates the nuclei of the ascending reticular activating system. The authors suggest that bruxism activates the production of neurotransmitters that stimulate the brain cortex and protect against neurodegenerative diseases such as dementia [27]. The current division of factors causing bruxism includes primary—idiopathic and secondary—radiogenic (occurrence of diseases and use of specific pharmacology) [20].

Due to the multifactorial etiology of the described condition, there are multiple diagnostic possibilities for bruxism. According to Lobbezoo et al., bruxism assessments can employ both non-instrumental approaches (notably self-report) and instrumental approaches (notably electromyography) [19]. During a non-instrumental examination, the patient or their family may report teeth grinding or strange tooth grinding sounds. Clinical examination may reveal increased tooth wear, jaw muscle pain or fatigue, and muscle hypertrophy [12,28]. In addition to the tools described in earlier paragraphs, “BruxApp” and “BruxScreen”, the “BruxChecker” was introduced in 2006 [29]. This is a simple device that patients use at night to visualize patterns of teeth grinding by marking areas where teeth contact occurs [29]. Questions regarding the occurrence of bruxism can also be found in other questionnaires, such as the Research Diagnostic Criteria for Temporomandibular Disorders (RDC/TMD) [30], Diagnostic Criteria for Temporomandibular Disorders (DC/TMD) [31], The Oral Behavior Checklist [32], and the Pittsburgh Sleep Quality Index [33].

In diagnostics, tooth wear was identified as one of the signs of bruxism. It is also one of the main negative consequences of the condition [34].The association of bruxism with temporomandibular disorders (TMDs) and headaches, as well as cervical spine dysfunctions, remains debatable [35,36]. Castrillon and Exposto note that, in light of available scientific evidence, it is doubtful that bruxism is a direct cause of pain [35]. Piekartz et al. emphasize that clinicians should focus on improving orofacial function and the mechanical sensitivity of tissues [36]. Certainly, for individuals without a distinct pathology, bruxism should be reframed not as inherently pathological, but rather as a muscular activity with various potential causes. This behavior can range from detrimental to benign, or even serve a protective function in relation to specific health factors [19,26,37]. In children, the occurrence of sleep bruxism diminishes gradually as they age. Following the natural course, the preferred approach involves observational and non-interventional strategies unless it indicates underlying primary conditions requiring treatment [26].

When treatment for bruxism is needed, the following therapies are applied. Based on the relationship between bruxism and emotional stress, psychological therapies such as cognitive behavioral therapy are used in treatment [38]. Physiotherapy and acupuncture, which have a relaxing effect on overly active muscles, are also employed [39,40]. To protect teeth from damage, occlusal splint therapy is recommended [41]. Biofeedback therapy, based on the premise that individuals with bruxism can unlearn their behavior and attempt to control it, is also utilized [42]. Visual, auditory, vibratory, or gustatory stimuli are used in biofeedback therapy [39]. Pharmacotherapy is also employed, including substances such as botulinum toxin, clonazepam, and clonidine [41,43]. Sleep hygiene is recommended as well, including avoiding stimulants at night (such as coffee, tobacco, and alcohol), limiting physical activity and mental stimulation before sleep [41].

Over the past five years, three meta-analyses regarding the occurrence of bruxism were conducted. Archer et al. conducted a meta-analysis on the prevalence of awake bruxism in the adult population, observing a prevalence rate of 15.44% [44]. Soares et al. conducted a meta-analysis on children with sleep bruxism in the global population, showing a prevalence rate of 31.16% [45]. The meta-analysis by Ferrari-Piloni et al. investigated the occurrence of bruxism in children residing in Brazil, revealing a prevalence of 25.8% in both sleep and awake states. Additionally, Ferrari-Piloni et al. observed regional differences in Brazil: Northeast—35.2%, Southeast—45.0%, and South—19.8% [46].

The meta-analysis by Ferrari-Piloni et al. [46] provided grounds to suggest that bruxism is associated with geographic factors. Additionally, contributing to the debate on the association between TMDs and bruxism, Mortazavi et al. conducted a meta-analysis aiming to examine and quantitatively determine the relationship between bruxism and temporomandibular joint disorders (TMDs). They demonstrated a positive association between bruxism and TMDs, highlighting that the presence of bruxism increases the likelihood of developing TMDs in the future [47]. Furthermore, a recent meta-analysis (2024) published by Zieliński et al. revealed that the occurrence of TMDs varies by continent (South America—47% compared to Asia—33% and Europe—29%, and North America—26%) [48]. Based on the aforementioned information, the authors decided to conduct a meta-analysis with the purpose of assessing the global prevalence of sleep bruxism and awake bruxism in pediatric and adult populations. According to the authors’ best knowledge, such a study was not conducted previously.

Four primary research questions were posed:

1. What is the global prevalence of sleep and awake bruxism in the population?
2. What is the global prevalence of sleep and awake bruxism in the population depending on the continent?
3. What is the global prevalence of sleep bruxism in the population depending on the continent, divided by age and gender?
4. What is the global prevalence of awake bruxism in the population depending on the continent, divided by age and gender?

The secondary research question was described during the registration of the protocol [49].

## 2. Materials and Methods

This systematic review is following the Preferred Reporting Items for Systematic reviews and Meta-Analyses [50] (PRISMA 2020 guidelines [51]) (Supplementary Material S1 and S2). The systematic review protocol was registered in the Open Science Framework (OSF) under the number identifier: DOI 10.17605/OSF.IO/ZE786 [49]. This systematic review and meta-analysis was conducted by analyzing studies published from 1 January 2003, to 31 December 2023. This was a 20-year timeframe, chosen based on earlier works [52,53,54].

### 2.1. The Data Collection Process

**Figure 1:**
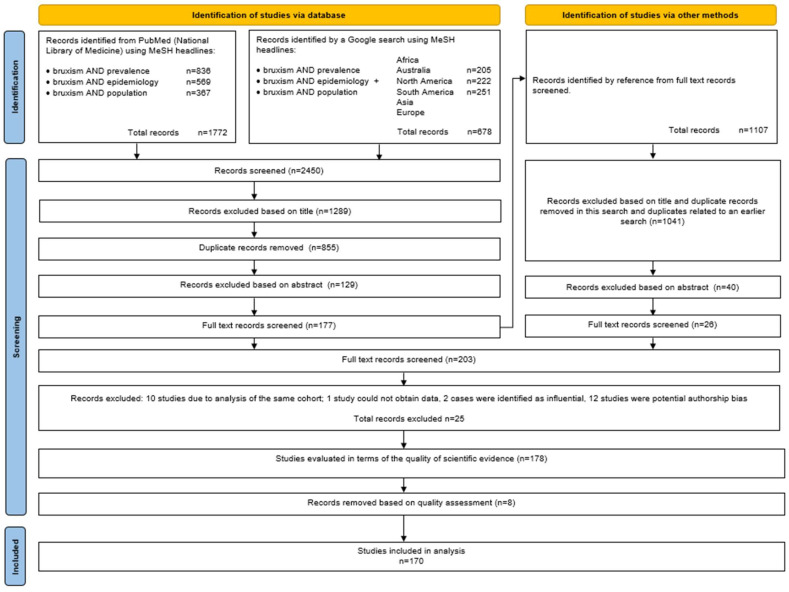
PRISMA flow diagram.

An analysis of the PubMed database (National Library of Medicine) was conducted from 22 March 2024 [49] to 22 June 2024, using keywords consistent with the MeSH (Medical Subject Headings) database. Building upon the previous reviews [48,55], the following keyword combination was utilized: bruxism AND prevalence, bruxism AND epidemiology, and bruxism AND population. Based on earlier work, it was decided to analyze only the PubMed database [48,55,56,57,58], supplemented by manual searches using the Google search engine with keyword combinations including the names of continents. Additionally, the snowballing procedure method was applied according to Wohlin’s recommendations [59] (Figure 1). From each study qualified for full-text assessment, bibliographies were recorded and searched for literature supplementation. This systematic review was carried out by two independent reviewers (G.Z. and A.P.) who first evaluated the titles of the papers, then the abstracts, and finally the full papers. All disputes were assessed by M.W.

From each study, the following structured information was collected (information gathering methods were adapted from the work [45]): DOI number, PMID or other numerical identifier of the study, last author’s surname, publication year, country, sample size, number of women and number of men, age of participants, percentage and number of individuals with bruxism (sleep and awake bruxism or both), diagnostic methods used, information about the approval of the research ethics committee for the study, and the presence or absence of conflicts of interest.

The summary of the PICO standards (P—population, I—intervention, C—comparison, and O—outcome) [60], is found in Table 1.

### 2.2. Evaluation of the Quality of Scientific Evidence

After qualifying 180 papers, it was decided to analyze the quality of the publications. A double assessment of the quality of publications was carried out to preserve the highest possible quality of evidence. In the first step, publications identified as doubtful were screened based on an adapted questionnaire created by Berger et al. [61]. Based on this questionnaire, the first selection was carried out. Publications after this stage were evaluated with a standard tool. The second step involved a standard assessment using the Joanna Briggs Institute (JBI) critical appraisal checklist [62]. The assessment was conducted similarly to the process of searching by two independent researchers (G.Z. and A.P.) in agreement with a third (M.W.).

This was accomplished using an adapted questionnaire utilized by Berger et al. [61]. The questionnaire was expanded with questions related to the tools proposed by Giannakopoulos et al. [63] and Stang et al. [64]. The complete questionnaire is available (Supplementary Material S3). It contains 7 questions, with the first Q1–Q4 pertaining to the research methodology (obtaining ethical approval, description of the study group, inclusion and exclusion criteria). Questions Q5 and Q6 relate to the study of bruxism (analysis of group size and tools used for bruxism assessment). The final question, Q7, addresses potential conflicts of interest. The acceptance threshold for the study was set at 9 points. This is because even the maximum score for questions Q1–Q4 and Q7 yields a value of 8 points. The study must gain additional points for significant sample size in question Q5 (over 100 individuals [61]) or for a more reliable diagnostic value of bruxism in Q6 [20,61,65]; the assessment results of the papers are provided (Supplementary Material S4).

The works [66,67,68,69,70,71,72,73,74,75,76,77,78,79,80,81,82,83,84,85,86,87,88,89,90,91,92,93,94,95,96,97,98,99,100,101,102,103,104,105,106,107,108,109,110,111,111,112,113,114,115,116,117,118,119,120,121,122,123,124,125,126,127,128,129,130,131,132,133,134,135,136,137,138,139,140,141,142,143,144,145,146,147,148,149,150,151,152,153,154,155,156,157,158,159,160,161,162,163,164,165,166,167,168,169,170,171,172,173,174,175,176,177,178,179,180,181,182,183,184,185,186,187,188,189,190,191,192,193,194,195,196,197,198,199,200,201,202,203,204,205,206,207,208,209,210,211,212,213,214,215,216,217,218,219,220,221,222,223,224,225,226,227,228,229,230] were included in the meta-analysis, and the details can be found in Supplementary Material S5.

At this stage, it was noted that only one study concerning Australia [231] and three studies concerning Africa [232,233,234] were qualified. However, the collected sample did not allow for the analysis of the prevalence of bruxism across continents. Nevertheless, the samples were included in the analysis of the global occurrence of bruxism. Works rejected during the analysis of full texts [15,187,235,236,237,238,239,240,241,242,243,244,245,246,247,248,249,250,251,252,253,254,255,256,257,258,259,260,261,262,263,264,265] and based on the above analysis, along with the justification, are included in Supplementary Material S6, (Figure 1).

**Figure 2:**
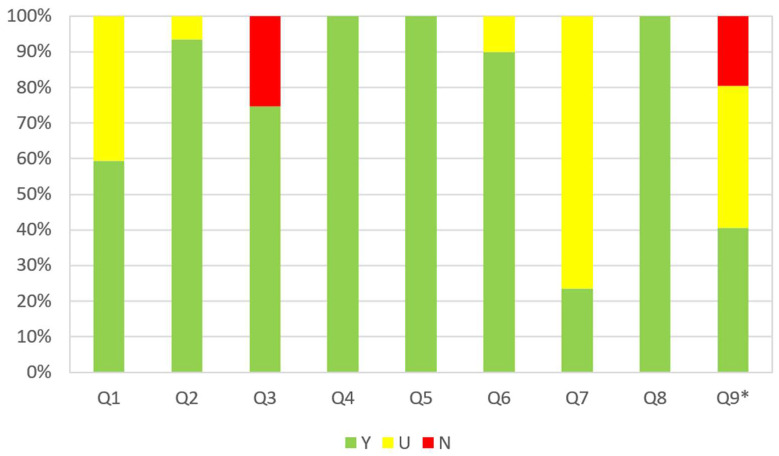
The summary results of the evaluation of studies using a Joanna Briggs classification. Q1—Was the sample frame appropriate to address the target population?; Q2—Were study participants recruited in an appropriate way?; Q3—Was the sample size adequate?; Q4—Were the study subjects and setting described in detail?; Q5—Was data analysis conducted with sufficient coverage of the identified sample?; Q6—Were valid methods used for the identification of the condition?; Q7—Was the condition measured in a standard, reliable way for all participants?; Q8—Was there appropriate statistical analysis?; Q9—Was the response rate adequate, and if not, was the low response rate managed appropriately?; Y—yes; N—no; U—unclear; and *—the question did not apply to 37 studies.

Qualified works were evaluated according to JBI. The assessment tool was provided by the JBI for analyzing analytical cross-sectional, case-control, and longitudinal studies. This tool was previously used in conducted meta-analyses regarding bruxism [44,45]. The JBI critical appraisal checklist consists of 9 questions, to which responses can be Yes (Y), No (N), Unclear (U), or marked as Not Applicable (N/A). The assessment guidelines were adopted according to the work of Munn et al. [266], and adaptations of the questions were made on the basis of previously published works [44,45] (Supplementary Material S7). The full list of questions and the assessment of the studies can be found in Supplementary Material S8. The results are presented graphically in Figure 2.

Based on the analysis using the JBI questionnaire, the greatest risk associated with the studies analyzed is related to the adequate sample size (Q3) and the response rate (Q9). The most significant uncertainties were observed in question Q7. This was due to the fact that most studies were based on proprietary assessment questionnaires or self-reported surveys.

### 2.3. Synthesis Methods

The objective of this analysis was to estimate the prevalence of bruxism across the entire sample, with additional stratifications by age and continent, as well as by gender. This approach allows for a comprehensive assessment of demographic variations in bruxism occurrence.

In this meta-analysis, the threshold for statistical significance was established at α = 0.05. The distribution of categorical variables pertaining to the characteristics of the study samples was detailed by reporting frequencies and percentages for each category. The distribution of numerical variables was described using the median (Mdn) and interquartile range, specifically the first (Q1) and third (Q3) quartiles, as metrics of dispersion.

#### 2.3.1. Pooling Effect Size Estimation

To estimate the pooled effect size, we employed the Generalized Linear Mixed-Effects Model (GLMM) with a binomial logit link. The GLMM integrates both fixed and random effects, and is particularly well-suited for meta-analyses, as it adeptly handles variations within and across a range of studies. Unlike the traditional inverse-variance method, the GLMM excels in managing heterogeneous datasets, ensuring a robust analysis of data involving diverse measurement scales such as counts and proportions [267,268].

The overall proportions were calculated employing the logit transformation, followed by back-transformation to the original scale. The selection of this transformation method was guided by specific characteristics of the dataset and the distribution of proportion values, with the assumption that there were no extreme values (i.e., very small or very large proportions). Confidence intervals for individual study results were estimated using the Agresti-Coull interval [269].

Heterogeneity among study results was quantified using the τ^2^, the square root of tau-squared (τ), and the I^2^ statistic [270]. The Higgins and Thompson’s H statistic was also calculated to measure the consistency of effect sizes across studies.

Variance components for τ^2^ were estimated using the maximum likelihood (ML) estimator, which facilitates an accurate estimation of between-study variance, which is critical given the substantial heterogeneity indicated by our data. The confidence intervals for the I^2^ and H statistics were estimated using methods based on the chi-squared distribution. The presence of heterogeneity was tested using both the Wald and the likelihood ratio test.

The evaluation of subgroup differences within the meta-analysis was performed using the Q-test for heterogeneity. The prediction interval for the prevalence of bruxism was estimated using the t-distribution.

The pooled effect of the studies was graphically depicted using a forest plot. This visualization method systematically represents the individual study results alongside their aggregated effect, facilitating a comprehensive overview of the data.

#### 2.3.2. Leave-One-Out Diagnostics

We utilized several leave-one-out diagnostics, such as externally standardized residual, DFFITS value, Cook’s distance, covariance ratio, the leave-one-out amount of (residual) heterogeneity, the leave-one-out test statistic of the test for (residual) heterogeneity, and DFBETAS values [271,272,273,274,275,276].

A case may be considered to be ‘influential’ if at least one of the following is true:

1. If the absolute value of DFFITS exceeds $3\times \sqrt{\frac{p}{k-p}}$, where *p* denotes the number of model coefficients and *k* the total number of cases. This threshold identifies cases that significantly affect the model’s predictions.
2. A case is considered influential if the lower tail probability of a chi-square distribution, calculated using the degrees of freedom defined by the Cook’s distance, exceeds 50%. This metric highlights cases that have a substantial impact on the parameter estimates across the model.
3. Influence is attributed to a case if its hat value surpasses $3\times \frac{p}{k}$. This measure quantifies the leverage of a case, indicating its relative ability to influence the fit of the model disproportionately.
4. A case is marked as influential if any of its DFBETAS values exceed 1.0. This condition reveals cases that, when excluded, lead to a significant change in the estimated regression coefficients, shifting them by more than one standard deviation.

#### 2.3.3. Publication Bayes

To evaluate publication bias in the study, a funnel plot was utilized for graphical representation. The asymmetry in the funnel plot was quantitatively assessed using Egger’s test as the primary statistical approach. This method involves regressing the standardized effect estimates against their precision, in accordance with the established guidelines by Egger et al. [277]. The regression analysis uses the standard error of the estimates as a predictor, with each study’s impact on the analysis adjusted by weighting inversely to their variance.

#### 2.3.4. Characteristics of the Statistical Tool

Analyses were conducted using the R Statistical language (version 4.3.1; R Core Team, 2023) on Windows 10 Pro 64 bit (build 19045), using the packages meta (version 6.5.0; [278]), dmetar (version 0.1.0; [279]), report (version 0.5.7; [280]), gtsummary (version 1.7.2; [281]), dplyr (version 1.1.3; [282]), and psych (version 2.3.9; [283]).

#### 2.3.5. Characteristics of the Meta-Analysis Sample

The prevalence of bruxism was analyzed across 176 scientific studies (170 publications analyzed 176 populations). Six studies [110,128,156,189,191,198] analyzed more than one population/age group) with sample sizes varied from 30 to 99,416 (Mdn = 533.5, Q1 = 224.3, Q3 = 1097.3). Of these, 97 studies (55.11%) focused on adult populations (aged over 18 years), while the remaining 79 studies targeted minors (up to 18 years of age). Geographically, the studies were distributed as follows: 63 studies (36.63%) involved individuals in South America, 58 studies (33.72%) in Asia, 43 studies (25.00%) in Europe, and 12 studies (6.82%) in North America.

Additionally, the studies explored the incidence of bruxism with regard to two specific variables: gender and the time of occurrence, distinguishing between events during sleep and those occurring after waking.

## 3. Results

Due to the number of analyses, the full description of the results can be found in Supplementary Material S9. Forest plots and funnel plots are included in Supplementary Materials S10 and S11.

### 3.1. Meta-Analysis of Global Bruxism (Sleep and Awake) Prevalence

**Figure 3:**
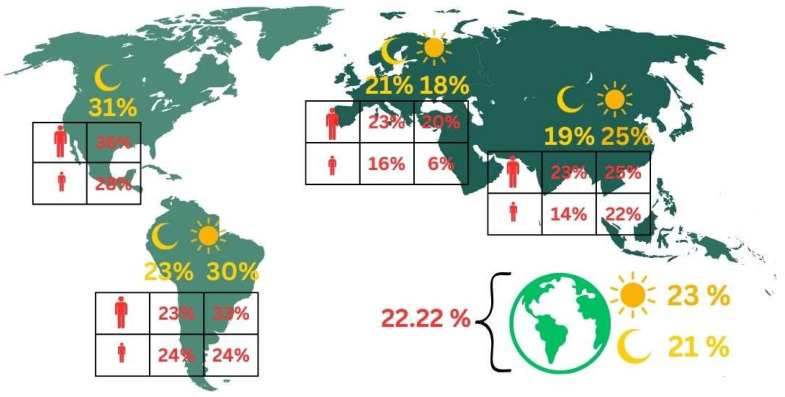
Graphical representation of the obtained results on the occurrence of bruxism in the population.

Using a random effects model, the pooled prevalence of bruxism was estimated at 22.22%, with a 95% confidence interval ranging from 19.59% to 25.11%. Additionally, a prediction interval was calculated, extending from 4.23% to 64.87%, indicating substantial variability in bruxism prevalence across different settings and populations (Figure 3).

### 3.2. Meta-Analysis of Global Bruxism (Sleep and Awake) Prevalence across Continents

The subgroup analysis by continent in Table 2 revealed significant variability in the prevalence of bruxism, reflecting the diverse geographical patterns of this condition. North America exhibits the highest prevalence rate at 29.07%, with a notably broad 95% confidence interval ranging from 15.85% to 47.14%. This wide interval suggests a high degree of uncertainty or variability in the data, possibly due to fewer studies (only eight in total) contributing to this regional estimate, as indicated by the highest τ^2^ = 1.22, suggesting substantial heterogeneity among the studies.

Europe and South America presented more moderate prevalence rates of 22.17% and 24.89%, respectively, with narrower confidence intervals, indicating more precise estimates. Europe’s studies, although more numerous (29 studies), still showed a high level of heterogeneity (I^2^ = 98.6%), which was slightly lower than that of South America, which had an I^2^ = 97.9%.

Asia, with 43 studies, reported the lowest prevalence of bruxism at 19.10%. However, it also presented a high level of heterogeneity (I^2^ = 99.7%), similar to North America, and a high τ^2^ = 1.03. This suggests that, despite having a larger number of studies, there is significant variation in the study results, which could be attributed to differences in study methodologies, populations, and diagnostic criteria across the continent.

The Q-statistics further support the presence of significant heterogeneity within each continent’s subgroup, with extremely high values observed, particularly in Asia (Q = 12,177.51). This reinforces the complexity and variability of bruxism prevalence across different geographical and cultural contexts.

### 3.3. Meta-Analysis of Global Sleep and Awake Bruxism Prevalence

#### 3.3.1. Sleep Bruxism

The analysis, employing a random effects model, estimated the prevalence to show sleep bruxism of 20.99%, with a 95% confidence interval that stretches from 18.69% to 23.50%. This prevalence estimate highlights the notable incidence of sleep bruxism among the global population studied (Figure 3).

#### 3.3.2. Sleep Bruxism as Assessed through Polysomnography

The present meta-analysis examines the global prevalence of sleep bruxism as assessed through polysomnography. It includes data from four studies, notably featuring two influential cases: Raphael et al. [182] and Maluly et al. [255]. This comprehensive dataset encompasses a total of 830 observations and 186 documented instances of sleep bruxism.

The random effects model, optimal for heterogeneous data, suggests a central prevalence estimate of 43.42%, with a 95% confidence interval ranging from 16.65% to 74.68%. This extensive range indicates considerable variation across the studies, likely attributable to differences in population demographics, diagnostic criteria, or methodological approaches.

The prediction interval, spanning from 0.11% to 99.81%, underscores the substantial diversity in sleep bruxism prevalence among different populations as assessed by future studies. This wide interval suggests that while some populations may exhibit almost no occurrence of sleep bruxism, in others, nearly all individuals could be affected, depending on genetic, environmental, and behavioral factors.

Heterogeneity quantification reveals extremely high variability among the included studies (I^2^ = 98.5%). The τ^2^ = 1.81 and τ = 1.35 further confirm significant dispersion in effect sizes beyond what would be expected by sampling error alone. This is also reflected in the high H = 8.16, indicating that the true effect size varies substantially across the studies, much more than would be expected if the studies were homogeneous.

Both the Wald and LRT for heterogeneity confirm this observation, with *p* < 0.001, indicating that the differences in prevalence estimates across studies are indeed statistically significant and not due to random chance. These tests, therefore, reinforce the presence of high heterogeneity, suggesting that factors unique to each study or population significantly influence the prevalence of sleep bruxism.

#### 3.3.3. Awake Bruxism

The results from a random effects model reveal a mean prevalence rate and show awake bruxism of 23.29%, with a 95% confidence interval ranging from 18.78% to 28.52%. This indicates that nearly one in four individuals may experience awake bruxism, a condition characterized by involuntary teeth grinding or jaw clenching while awake (Figure 3).

### 3.4. Meta-Analysis of Global Sleep and Awake Bruxism Prevalence across Continents

#### 3.4.1. Sleep Bruxism

The subgroup analysis of sleep bruxism prevalence by continent in Table 3 reveals a complex picture of how this condition manifests across different geographical regions, influenced by varying study characteristics, methodologies, and potentially underlying regional factors affecting bruxism rates.

In Europe, where 35 studies were analyzed, the prevalence of sleep bruxism is estimated at 21%. The narrower confidence interval of 18% to 26% suggests a somewhat consistent measurement across studies, albeit the high I^2^ = 99.1% indicates that the variability among study findings is predominantly due to heterogeneity rather than chance. This could be reflective of different population demographics studied or varying diagnostic criteria used across European countries.

North America presents the highest prevalence at 31%, but this figure comes with a wide confidence interval from 12% to 57%, largely attributable to the small number (only 5) of studies conducted there. Such a wide interval indicates a significant uncertainty in the estimate, compounded by the highest τ^2^ = 1.52 among the groups, suggesting that the few studies included may be highly diverse in terms of methodology or population characteristics. The extremely high I^2^ = 99.7% further supports this view, pointing to substantial inconsistencies among the findings of these studies.

In contrast, South America, with the highest number of studies (54), shows a prevalence of 23%. The relatively tighter confidence interval of 19% to 28% alongside a τ^2^ = 0.75 suggests that while there is considerable variation among the studies, it is somewhat less than that observed in North America. The high I^2^ = 98.1% still underscores significant heterogeneity, indicating that despite a larger data pool, the consistency across study results is limited.

Asia, with 46 studies, reports the lowest prevalence at 19%, and its confidence interval ranges from 15% to 23%. The τ^2^ = 0.71, closely aligned with that of South America, and the extremely high Q = 10,727.90 combined with an I^2^ = 99.6% highlights that heterogeneity is also a major factor in Asian studies. This suggests that, similar to other continents, varying study designs, populations, and diagnostic criteria across Asian countries contribute to the diverse findings.

The test for subgroup differences across the continents yielded a Q(3) = of 3.07, *p* = 0.380, suggesting that the variations in bruxism prevalence between the continents are not statistically significant, despite the apparent differences in point estimates. This indicates that while there are numerical differences in the reported prevalence rates, these differences could be attributed to the high heterogeneity within each subgroup rather than true differences in prevalence across continents (Figure 3).

#### 3.4.2. Awake Bruxism

The prevalence rates broken down by continent are shown in Table 4. Europe shows the lowest bruxism prevalence at 17.90%, with a very narrow confidence interval but still substantial heterogeneity (I^2^ = 99.0%). South America exhibits the highest prevalence at 29.86%, albeit with a wider confidence interval, reflecting more variability in the estimates and slightly less, yet still very high, heterogeneity (I^2^ = 97.8%). Asia has a prevalence rate of 24.91%, with associated confidence intervals and heterogeneity metrics similar to those of Europe (Table 4).

Despite these differences, the test for subgroup differences across continents (Q = 3.84, df = 2, and *p* = 0.147) indicates that these variations are not statistically significant. This suggests that while numerically different, the variability within each continent’s estimates might be influencing the perception of between-continent differences (Figure 3).

### 3.5. Meta-Analysis of Global Sleep and Awake Bruxism Prevalence among Females

#### 3.5.1. Sleep Bruxism

The results derived from this comprehensive analysis, which employed a random effects model, suggest a central estimate of sleep bruxism prevalence at 11.68%, with a 95% confidence interval ranging from 9.07% to 14.07%. This prevalence rate indicates that sleep bruxism is a relatively common condition among females globally, albeit with significant variability.

When breaking down the data into subgroups of minors and adults, notable differences emerge. The prevalence among minors is 8.90%, with a narrower confidence interval of 6.96% to 11.30%, and among adults, it is higher at 14.49%, with a confidence interval of 11.14% to 18.64%. The heterogeneity within these subgroups remains high, with τ^2^ = 0.62 and 1.00, respectively, and both groups showing I^2^ = 98%.

**Figure 4:**
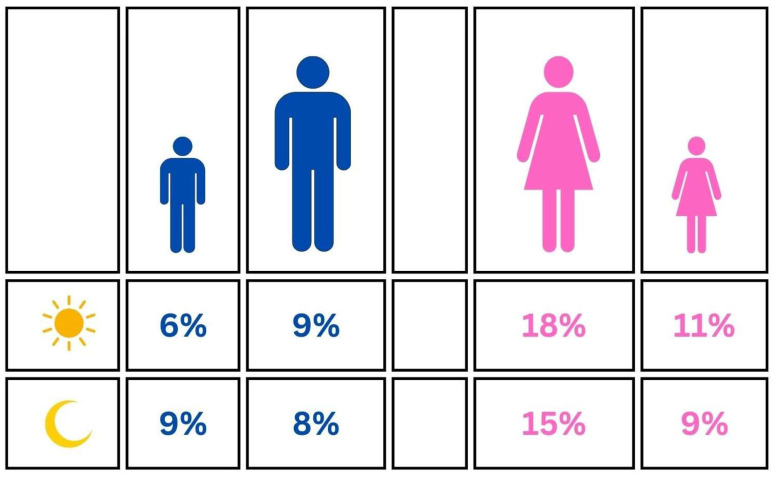
Graphical representation of the prevalence among males and females.

Furthermore, the test for subgroup differences reveals a Q(1) = 7.23 with *p* = 0.007, indicating statistically significant differences between the prevalence rates of minors and adults. This suggests that age is a significant factor influencing the prevalence of sleep bruxism among females (Figure 4).

#### 3.5.2. Awake Bruxism

The findings from this specific subset reveal a pooled prevalence estimate of 17.07%, as indicated by the random effects model with a 95% confidence interval ranging from 12.39% to 23.05%. This prevalence suggests that awake bruxism is a significant concern among females, though the variation is notable.

When examining the results by age subgroups, the prevalence in minors is estimated at 11.28%, with a CI 95% stretching from 5.19% to 22.79%. Adults, however, exhibit a higher prevalence rate of 18.35%, with a CI 95% from 13.02% to 25.21%. Despite the apparent difference in prevalence rates, the test for subgroup differences, which yields a Q = 1.43 with *p* = 0.233, indicates that these differences are not statistically significant. This suggests that while numerically different, the prevalence rates between minors and adults do not diverge sufficiently when accounting for the high levels of variability within each subgroup (Figure 4).

### 3.6. Meta-Analysis of Global Sleep and Awake Bruxism Prevalence among Males

#### 3.6.1. Sleep Bruxism

The results from this analysis, utilizing a random effects model, indicate a pooled prevalence rate of 8.48%, with a CI 95% from 7.25% to 9.89%. This suggests that while sleep bruxism is a notable health concern among males globally, the prevalence is somewhat moderate.

When dissecting the data into age subgroups, the prevalence in minors is slightly higher at 8.96%, with a 95% confidence interval of 7.00% to 11.40%, compared to 8.11% among adults, whose confidence interval ranges from 6.64% to 9.88%. The heterogeneity within these subgroups is notably high, with τ^2^ = 0.59 for minors and 0.47 for adults, and I^2^ = 99.4% and 98.7%, respectively.

The test for subgroup differences presents a Q = 0.38 with one degree of freedom and a *p* = 0.536, indicating that the differences in prevalence between minors and adults are not statistically significant. This suggests that age, within this data set, does not play a major role in differentiating the prevalence of sleep bruxism among males (Figure 4).

#### 3.6.2. Awake Bruxism

Awake bruxism presents a pooled prevalence of 8.33%, as per the random effects model. The 95% confidence interval for this estimate ranges from 6.33% to 10.89%, indicating a moderate level of prevalence, but with considerable uncertainty about the precise rate.

By brokering down into age-specific subgroups, the results reveal that minors have a lower estimated prevalence of awake bruxism at 5.62%, with a confidence interval from 2.31% to 13.05%. Adults, on the other hand, show a higher prevalence of 8.91%, with a confidence interval ranging from 6.75% to 11.69%. Despite these numerical differences, the test for subgroup differences yields a Q = 1.00 with *p* = 0.317, indicating that these differences are not statistically significant.

This suggests that while there appears to be a trend towards higher prevalence in adults, the data do not strongly differentiate between the two age groups in terms of statistical significance, likely due to the high levels of variability and heterogeneity within each subgroup (Figure 4).

### 3.7. Meta-Analysis of Global Sleep and Awake Bruxism Prevalence by Age and Continent

#### 3.7.1. Sleep Bruxism

Subgroup by continent and age group analysis reported a in Table 5 reveals nuanced insights. For instance, the prevalence in European adults is notably higher at 22.55% compared to European minors at 16.17%, with a relatively lower but still substantial heterogeneity in European adults (τ^2^ = 0.47, I^2^ = 99.0%). North American data show higher prevalence rates, particularly among adults at 36.39%, though this subgroup has a notably wide confidence interval, reflecting high uncertainty possibly due to the small number of studies (k = 2). This is supported by a very high τ^2^ = 3.32, indicating extreme variability.

In South America and Asia, the prevalence rates tend to be lower among minors compared to adults, with Asian minors showing the lowest prevalence at 14.40%. The heterogeneity remains very high across all subgroups, suggesting that even within continents and age groups, there are significant differences in study outcomes (Table 5).

The test for subgroup differences across continent-age categories shows a Q (7) = 10.04, *p* = 0.187, suggesting that while there are differences in prevalence rates across different subgroups, these differences are not statistically significant. This could imply that factors other than just continent and age might be influencing the prevalence rates, such as cultural differences, healthcare access, and diagnostic criteria.

#### 3.7.2. Awake Bruxism

The meta-analysis detailed in Table 6 offers a nuanced look at the prevalence of awake bruxism across different continents and age groups, revealing a complex pattern influenced by geographic and demographic factors. The results underscore the variation not only in prevalence rates but also in the degree of heterogeneity among the subgroups analyzed.

Starting with Europe, the contrast between minors and adults in terms of bruxism prevalence is striking. Minors show a notably lower prevalence at 6%, which, despite being based on just two studies, suggests a lesser degree of awake bruxism in younger populations within Europe. However, the high level of heterogeneity (I^2^ = 92.5%) in this subgroup indicates that even within this narrow age range, factors contributing to bruxism are varied and possibly influenced by diverse cultural or environmental conditions. On the other hand, the adult population in Europe shows a higher prevalence at 20%, supported by a more substantial number of studies (20 in total). The even higher heterogeneity seen here (I^2^ = 98.7%) could reflect a wider range of contributing factors, such as stress, lifestyle, and possibly even differences in how bruxism is diagnosed and reported across European countries.

Moving to South America, the findings are intriguing due to the higher prevalence rates observed, especially in adults (33%). This region also exhibits significant variability, particularly among minors where the prevalence rate of 24% comes with a very broad confidence interval (0.08 to 0.54) and an extremely high heterogeneity (I^2^ = 99.0%). This suggests that there are specific local factors, possibly including socio-economic and cultural influences, that might exacerbate or mitigate the incidence of bruxism. The adult group, while also showing substantial heterogeneity (I^2^ = 95.9%), indicates a consistently higher prevalence across studies compared to minors, hinting at age-related factors that might influence the condition’s manifestation.

Asia presents a more consistent picture between minors and adults, with prevalence rates of 22% and 25%, respectively. The lower heterogeneity in minors (I^2^ = 95.9% with a lower τ^2^ value) compared to adults (I^2^ = 98.5%) could indicate more uniform diagnostic criteria or reporting practices within this demographic across the Asian studies. However, the high heterogeneity in adults suggests diverse underlying factors and possibly a range of responses to cultural, environmental, and biological influences.

The test for subgroup differences yielded a Q = 28.13 with a *p* < 0.001, indicating statistically significant differences in awake bruxism prevalence across different continental and age subgroups. This variation could be influenced by a range of factors, including genetic predispositions, cultural differences in stress management, diagnostic criteria, and awareness of the condition.

### 3.8. Meta-Analysis of Sleep and Awake Bruxism Prevalence by Continent and Age

In the meta-analysis of sleep bruxism prevalence among females by age and continent, the test for subgroup differences across continent and age categories reveals a Q(7) = 121.17, *p* < 0.001, indicating significant differences in the prevalence of sleep bruxism among different demographic and geographic groups, probably through small group sizes (Table 7).

In the meta-analysis of awake bruxism prevalence by continent and age among females, the test for subgroup differences confirms significant variability between these groups (Q = 72.17, *p* < 0.001), highlighting the influence of both geographic and age-related factors on awake bruxism prevalence among females. The notable differences in heterogeneity and prevalence rates between continents and age groups suggest that local environmental, cultural, and healthcare factors play critical roles in the manifestation and reporting of awake bruxism among females (Table 7).

In the meta-analysis of sleep bruxism prevalence among males by age and continent, the test for subgroup differences yields a Q = 339.82 with 6 degrees of freedom, accompanied by a *p* < 0.001. This result indicates that the variations in prevalence rates across different demographic and geographic subgroups are statistically significant. The notably high prevalence rate within the North American minors subgroup, which is considerably greater than those observed in other groups, likely contributes to these differences. However, the impact of this subgroup on the overall analysis should be interpreted with caution due to its small size (Table 6).

In the meta-analysis of awake bruxism prevalence by continent and age among males, the significant test for subgroup differences (Q = 53.34, *p* < 0.001) confirms that geographical and age-related factors play a crucial role in the prevalence of awake bruxism among males. These findings underscore the complexity of awake bruxism as a global health concern, influenced by a myriad of factors ranging from genetics and lifestyle to healthcare practices and cultural norms. The marked differences between continents and age groups highlight the need for targeted research and tailored interventions to effectively manage and mitigate awake bruxism in diverse male populations (Table 7).

## 4. Discussion

This meta-analysis had the purpose of assessing the global prevalence of sleep bruxism and awake bruxism in pediatric and adult populations. In response to the research questions posed in the introduction:

Question 1. What is the global prevalence of sleep and awake bruxism in the population? The global prevalence of sleep bruxism is 21%, and awake bruxism prevalence is 23%.

Question 2. What is the global prevalence of sleep and awake bruxism in the population depending on the continent? The prevalence of sleep bruxism was highest in North America at 31%, followed by South America at 23%, Europe at 21%, and Asia at 19%. The prevalence of awake bruxism was highest in South America at 30%, followed by Asia at 25%, and Europe at 18%.

Question 3. What is the global prevalence of sleep bruxism in the population depending on the continent, divided by age and gender? Sleep bruxism prevalence in North America was 36% in adults and 28% in minors, in South America 23% in adults and 24% in minors, in Europe 23% in adults and 16% in minors, and in Asia 23% in adults and 14% in minors.

Question 4. What is the global prevalence of awake bruxism in the population depending on the continent, divided by age and gender? Awake bruxism prevalence in South America was 33% in adults and 24% in minors, in Europe 20% in adults and 6% in minors, and in Asia 25% in adults and 22% in minors.

In the context of the main research questions, we want to highlight the potential impact of study heterogeneity. Regarding the first question and the occurrence of sleep bruxism, the result of the Egger’s test yielded a t (142) = 1.13, *p* = 0.258, indicating no statistically significant evidence of publication bias in the dataset. This suggests that the funnel plot is symmetrical, implying a balanced representation of studies with varying levels of variance. Smaller studies with less favorable outcomes are not disproportionately missing from the analysis. It is important to note that the analysis also considered substantial residual heterogeneity variance (τ^2^ = 151.99), indicating significant variability among the study estimates even after accounting for sampling variance. In the analysis of awake bruxism, the Egger linear regression test of funnel plot asymmetry yielded a t (57) = −1.27, *p* = 0.208. This *p*-value, above the significance threshold of 0.05, suggests no statistically significant evidence of publication bias in the dataset being analyzed. This indicates that the funnel plot is relatively symmetric, and the results of the meta-analysis are unlikely to be significantly influenced by selective publication of studies with more favorable outcomes (Supplementary Material S9).

Analyzing heterogeneity in response to the second question regarding sleep bruxism, the test for subgroup differences across continents yielded a Q(3) = 3.07, *p* = 0.380. This result suggests that the variations in bruxism prevalence between continents are not statistically significant, despite apparent differences in point estimates. This indicates that the numerical differences in reported prevalence rates may be due to high heterogeneity within each subgroup rather than genuine differences in prevalence across continents. In the analysis of awake bruxism, both the Wald and likelihood ratio tests for heterogeneity yielded *p* < 0.001, indicating significant heterogeneity among studies. This confirms that the studies included in the meta-analysis are estimating a range of effect sizes influenced by various factors, rather than a single underlying effect size. Despite this heterogeneity, the test for subgroup differences across continents (Q = 3.84, df = 2, and *p* = 0.147) suggests that these variations are not statistically significant. This implies that although there are numerical differences, the variability within estimates for each continent may be affecting the perception of differences between continents (Supplementary Material S9).

Analyzing question 3 in the context of sleep bruxism, the meta-analysis shows exceptionally high heterogeneity, with I^2^ = 99.4%, τ^2^ = 0.72, and τ = 0.85. These values indicate significant differences in study outcomes, likely stemming from methodological disparities, demographic variations, or differing diagnostic criteria across studies. The heterogeneity is further supported by the Q test (Wald = 21506.21, LRT = 23658.28), both yielding *p* < 0.001, indicating that the variability among studies is statistically significant and not due to chance (Supplementary Material S9).

Analyzing question 4 regarding awake bruxism, the heterogeneity observed in this meta-analysis is exceptionally high, with I^2^ = 98.8%. This suggests that nearly all of the variability in reported prevalence rates arises from genuine differences in study characteristics rather than random variance. Supporting this, τ^2^ = 1.12 and τ = 1.06 highlight substantial differences in true effect sizes across studies. The H statistic (H = 8.97) confirms significant dispersion in effect sizes among the included studies. Both the Wald and likelihood ratio tests for heterogeneity yielded p-values of zero, indicating significant variability that is not attributable to chance. These findings underscore the intricate nature of awake bruxism, influenced by a complex interplay of geographic, demographic, and methodological factors (Supplementary Material S9).

In our study, we determined that the global prevalence of bruxism (sleep and awake) is 22.22%, a result similar to the study by Ferrari-Piloni et al. [46]. It is worth noting further observations that sleep bruxism had the second highest occurrence in South America at 23%, and awake bruxism was highest in South America at 30%. These findings are consistent with those of Ferrari-Piloni et al. [46]. They also align with a meta-analysis on the prevalence of TMDs [48], where the highest occurrence of TMDs was similarly reported in South America at 47%. These results hypothetically suggest a connection between the occurrence of bruxism and TMDs. Such a connection was noted in the study by Mortazavi et al. [47], where the authors highlighted a positive association between bruxism and TMDs, with the presence of bruxism increasing the likelihood of future TMDs development.

It is also worth noting that despite the differences in percentage values in both cases, there were no statistically significant differences in the occurrence depending on the continent for sleep bruxism and awake bruxism. For sleep bruxism, the test for subgroup differences across the continents yielded a Q(3) = of 3.07, *p* = 0.380, suggesting that the variations in bruxism prevalence between the continents are not statistically significant, despite the apparent differences in point estimates. This indicates that while there are numerical differences in the reported prevalence rates, these differences could be attributed to the high heterogeneity within each subgroup rather than true differences in prevalence across continents. Similarly, for awake bruxism, the test for subgroup differences between continents (Q = 3.84, df = 2, and *p* = 0.147) indicates that these variations are not statistically significant. This suggests that, although numerically different, the variability within each continent’s estimates may influence the perception of differences between continents.

Soares et al. conducted a meta-analysis on children with sleep bruxism in the global population, showing a prevalence rate of 31.16% [45]. In our study, it turned out that sleep bruxism affects 21%, including both adults and minors. When divided among minors, bruxism was reported in 9% of minor females and males. Observable differences are likely associated with the period included in the analysis. In the study by Soares et al., this period was 10 years [45], whereas in our study, it was 20 years. However, analysis restricted to polysomnographic studies revealed a prevalence of sleep bruxism at 43%. Due to the small sample size, these results should be interpreted cautiously, and further analyses should be conducted in the future. This underscores the ongoing need to monitor the occurrence of bruxism in the population.

Archer et al. conducted a meta-analysis on the prevalence of awake bruxism in adults, finding a prevalence rate of 15.44% [44]. These results are similar to what we obtained. The global prevalence of awake bruxism was estimated at 23%. Among adult females, it was 18%, and among males, 9%.

The meta-analysis by Ferrari-Piloni et al. investigated the occurrence of bruxism in children residing in Brazil, revealing a prevalence of 25.8% in both sleep and awake states. Additionally, Ferrari-Piloni et al. observed regional differences in Brazil: Northeast—35.2%, Southeast—45.0%, and South—19.8% [46]. The meta-analysis by Ferrari-Piloni et al. [46] provided grounds to suggest that bruxism is associated with geographic factors.

### Limitations of the Study

Among the limitations of the study is the lack of analysis of the prevalence of bruxism (sleep and awake bruxism) on the continents of Africa and Australia. As mentioned in the methodology, this was due to not collecting an adequate sample for analysis (one study concerning Australia [231] and three studies concerning Africa [232,233,234]). This illustrates a dependency that was already observed in an earlier meta-analysis concerning the occurrence of TMDs [48]. An additional limitation is the lack of analyses regarding awake bruxism in North America. At that time, an adequate number of studies needed to conduct the meta-analysis was also not collected. This indicates a research gap and underscores the need to conduct high-quality dental research on the epidemiology of diseases and parafunctions of the stomatognathic system on these continents [8,48,58]. Another limitation is the failure to consider the division in the pediatric population into permanent and mixed dentition. We suggest including these variables in subsequent analyses.

Another limitation of the study is what was observed in the analysis of the JBI questionnaire, specifically Q3, regarding the selection of an appropriate sample size, Q9, addressing the adequacy of the response rate, and Q7, concerning the diagnostic method for bruxism. Most studies relied on custom-made research questionnaires and self-reports from participants or their caregivers (in the case of children). Probably contributing to the high heterogeneity of results in this meta-analysis. Of course, both non-instrumental approaches (e.g., self-reports) and instrumental methods (e.g., electromyography) can be used to assess bruxism [19]. However, different research questionnaires create the potential for errors (for example, studies by Raphael et al. [182] and Restrepo et al. [185] highlight the risk of potential error associated with self-reported and parent-reported bruxism). Therefore, we highlight this potential risk. With the emergence of the standardized diagnostic tool BruxScreen [17], we hope that future epidemiological studies will be conducted using this tool among others.

The final limitation of this meta-analysis is the use of a single database. Despite conducting additional searches using an internet search engine and employing snowball sampling, there is a risk of selection bias.

## 5. Conclusions

- The global bruxism (sleep and awake) prevalence is 22.22%.
- The bruxism (sleep and awake) prevalence in North America is 29%, in South America is 25%, in Europe is 22%, and in Asia is 19%.
- The global sleep bruxism prevalence is 21% and awake prevalence is 23%.
- The occurrence of sleep bruxism, based on polysomnography, was estimated at 43%; however, due to the small sample size, these results should be approached with caution and the analyses should be repeated in the future.
- One in four individuals may experience awake bruxism.
- In the women population, it was determined that 12% of women experience sleep bruxism and 17% experience awake bruxism. Bruxism is a significant factor among women.
- In adult women, the frequency of sleep bruxism was 15%, while in minors it was 9%. This suggests that age is a significant factor influencing the prevalence of sleep bruxism among females.
- Age did not play a key role in the occurrence of awake bruxism in women. In adult women, it occurred at a rate of 18%, while in minors, it was 11%.
- In the male population, it was determined that 9% of men experience sleep bruxism and 8% experience awake bruxism.
- In adult men, the frequency of sleep bruxism was 8%, while in minors it was 9%. This suggests that age, within this data set, does not play a major role in differentiating the prevalence of sleep bruxism among males.
- Age did not play a key role in the occurrence of awake bruxism in men. In adult men, it occurred at a rate of 9%, while in minors, it was 6%.
- The highest prevalence of sleep bruxism was observed in North America at 31%, followed by South America at 23%, Europe at 21%, and Asia at 19%.
- The prevalence of awake bruxism was highest in South America at 30%, followed by Asia at 25%, and Europe at 18%.
- When divided by age, it was observed that sleep bruxism occurred in North America in 36% of adults and 28% of minors, in South America in 23% of adults and 24% of minors, in Europe in 23% of adults and 16% of minors, and in Asia in 23% of adults and 14% of minors.
- When divided by age, it was observed that awake bruxism occurred in South America in 33% of adults and 24% of minors, in Europe in 20% of adults and 6% of minors, and in Asia in 25% of adults and 22% of minors.
- It was observed that age is a significant factor for the occurrence of sleep bruxism in women, but this was not observed in men.

## Figures and Tables

**Table 1:** PICO Summary of Inclusion and Exclusion Criteria.

	Inclusion	Exclusion
**Population**		
	Both adult and pediatric populations.	Studies on bruxism occurrence in populations with: post-traumatic stress disorder, Down syndrome, respiratory disorders, sleep apnea, cerebral palsy, Alzheimer’s disease, Parkinson’s disease, and temporomandibular disorders. Studies on bruxism occurrence in populations subjected to higher stress environments: military personnel, police officers, prison inmates, etc.
**Intervention**		
	Screening for bruxism (during sleep, awake, or both).	
**Comparison**		
	Bruxism subjects (during sleep, awake, or both) versus subjects in good health.	
**Outcome**		
	Information in the study on: epidemiology, prevalence, and bruxism (during sleep, awake, or both) population. Assessment scores equal to or greater than 9 according to the author’s scale.	Assessment scores lower than 9 based on the author’s scale. Absence of information regarding ethical committee approval for the study. Lack of information or questionable diagnosis of bruxism.
**Study Design**		
	Studies aimed at evaluating the prevalence of bruxism (during sleep, awake, or both) in various populations. The inclusion criterion for continents required at least two studies with a combined total of more than 500 participants [48].	Narrative reviews Systematic articles Meta-analyses Expert opinions Case reports or series of patients Working language other than English

**Table 2:** Variability in bruxism (sleep and awake) prevalence: a subgroup analysis by continent.

Continent	k	Proportion	CI 95%	τ^2^	Q	I^2^
Europe	29	0.22	0.18–0.27	0.52	2023.04	98.6%
North America	8	0.29	0.16–0.47	1.22	3041.78	99.8%
South America	56	0.25	0.21–0.29	0.74	2580.68	97.9%
Asia	43	0.19	0.15–0.24	1.03	12,177.51	99.7%

k—number of studies; CI 95%—confidence interval 95%.

**Table 3:** Variability in sleep bruxism prevalence: a subgroup analysis by continent.

Continent	k	Proportion	CI 95%	τ^2^	Q	I^2^
Europe	35	0.21	0.18–0.26	0.49	3855.24	99.1%
North America	5	0.31	0.12–0.57	1.52	1575.75	99.7%
South America	54	0.23	0.19–0.28	0.75	2800.83	98.1%
Asia	46	0.19	0.15–0.23	0.71	10,727.90	99.6%

k—number of studies; CI 95%—confidence interval 95%.

**Table 4:** Variability in awake bruxism prevalence: a subgroup analysis by continent.

Continent	k	Proportion	CI 95%	τ^2^	Q	I^2^
Europe	22	0.18	0.13–0.25	0.88	2034.28	99.0%
South America	15	0.30	0.19–0.44	1.44	648.92	97.8%
Asia	19	0.25	0.18–0.34	0.91	1161.37	98.5%

k—number of studies; CI 95%—confidence interval 95%.

**Table 5:** Variability in sleep bruxism prevalence: a subgroup analysis by age.

Continent	Age	k	Proportion	CI 95%	τ^2^	Q	I^2^
Europe	Minors	6	0.16	0.10–0.25	0.44	260.30	98.1%
Europe	Adults	29	0.23	0.18–0.27	0.47	2803.02	99.0%
North America	Minors	3	0.28	0.16–0.45	0.38	147.98	98.6%
North America	Adults	2	0.36	0.04–0.88	3.31	102.31	99.0%
South America	Minors	34	0.24	0.20–0.28	0.48	1202.59	97.3%
South America	Adults	20	0.23	0.16–0.32	1.18	1579.70	98.8%
Asia	Minors	19	0.14	0.10–0.20	0.78	2527.53	99.3%
Asia	Adults	27	0.23	0.18–0.28	0.54	2612.05	99.0%

k—number of studies; CI 95%—confidence interval 95%.

**Table 6:** Variability in awake bruxism prevalence: a subgroup analysis by continent and age.

Continent	Age	k	Proportion	CI 95%	τ^2^	Q	I^2^
Europe	minors	2	0.06	0.04–0.10	0.14	13.37	92.5%
Europe	adults	20	0.20	0.14–0.27	0.79	1499.74	98.7%
South America	minors	5	0.24	0.08–0.54	2.23	412.04	99.0%
South America	adults	10	0.33	0.21–0.48	0.95	220.62	95.9%
Asia	minors	3	0.22	0.18–0.28	0.06	49.06	95.9%
Asia	adults	16	0.25	0.17–0.36	1.09	987.94	98.5%

k—number of studies; CI 95%—confidence interval 95%.

**Table 7:** Variability in sleep bruxism prevalence by continent and age among sex.

Continent	Age	Proportion Females		Proportion Males	
		Sleep Bruxism	Awake Bruxism	Sleep Bruxism	Awake Bruxism
Europe	Minors	0.05	0.03	0.06	0.01
Europe	Adults	0.15	0.14	0.08	0.06
North America	Minors	0.15	-	0.27	-
North America	Adults	0.78	-	-	-
South America	Minors	0.11	-	0.11	-
South America	Adults	0.15	0.26	0.08	0.10
Asia	Minors	0.07	0.15	0.07	0.09
Asia	Adults	0.13	0.27	0.07	0.17

## Data Availability

Not applicable.

## Supplementary Materials

The following supporting information can be downloaded at: https://www.mdpi.com/article/10.3390/jcm13144259/s1, Supplementary Material S1: PRISMA 2020 Abstract Checklist. Supplementary Material S2: PRISMA 2020 Checklist. Supplementary Material S3: Adapted questionnaire for initial selection. Supplementary Material S4: The results of the evaluation of studies using an adapted questionnaire. Supplementary Material S5: Presentation of the studies included the meta-analysis. Supplementary Material S6: Rejected studies from the meta-analysis. Supplementary Material S7: Adapted Joanna Briggs classification. Supplementary Material S8: The results of the evaluation of studies using an Joanna Briggs classification. Supplementary Material S9: Detailed description of the conducted meta-analysis. Supplementary Material S10: Presentation of forest plots. Supplementary Material S11: Presentation of funnel plots.
