# Supplementary material for: Global Prevalence of Sleep Bruxism and Awake Bruxism in Pediatric and Adult Populations: A Systematic Review and Meta-Analysis
Source: J Clin Med. 2024 Jul 22;13(14):4259. doi: 10.3390/jcm13144259 (PMC11278015; doi:10.3390/jcm13144259)
Supplement: Supplementary file 1 [file jcm-13-04259-s001.zip › Supplementary Material S10 Presentation of forest plots..pdf]

Systematic Review

Global Prevalence of Sleep Bruxism and Awake Bruxism in Pediatric and Adult Populations: A Systematic Review and Meta-Analysis

Grzegorz Zieliński [1,*], Agnieszka Pająk [2], Marcin Wójcicki [3]

[1] Department of Sports Medicine, Medical University of Lublin, 20-093 Lublin, Poland
[2] Clinic of Anaesthesiology and Paediatric Intensive Care, Medical University of Lublin, Gebali Str. 6, 20-093 Lublin, Poland
[3] Independent Unit of Functional Masticatory Disorder, Medical University of Lublin, 20-093 Lublin, Poland
***** Correspondence: grzegorz.zielinski@umlub.pl

Figure S1. Global prevalence of bruxism regardless of type.

Study	Events	Total	Proportion	95%-CI
Ahlberg, 2023	2755	11427	0.24	[0.23; 0.25]
Ahlberg, 2008	82	750	0.11	[0.09; 0.13]
Alfano, 2018	10	31	0.32	[0.18; 0.50]
Alonso, 2021	190	434	0.44	[0.39; 0.48]
Al-Swaje, 2019	118	200	0.59	[0.52; 0.66]
Amaral, 2022	88	556	0.16	[0.13; 0.19]
Bharti, 2005	12	103	0.12	[0.07; 0.19]
Bolsson, 2023	105	429	0.24	[0.21; 0.29]
Borie, 2016	42	182	0.23	[0.18; 0.30]
Bortoletto, 2017	49	103	0.48	[0.38; 0.57]
Botelho, 2019	228	427	0.53	[0.49; 0.58]
Brancher, 2020	167	556	0.30	[0.26; 0.34]
Breda, 2023	406	4321	0.09	[0.09; 0.10]
Bucci, 2018	9	47	0.19	[0.10; 0.33]
Cai, 2013	423	2591	0.16	[0.15; 0.18]
Câmara-Souza, 2023	26	69	0.38	[0.27; 0.49]
Carra, 2011	91	604	0.15	[0.12; 0.18]
Cavalcante-Leão, 2017	85	531	0.16	[0.13; 0.19]
Chattrattrai, 2022	352	2251	0.16	[0.14; 0.17]
Cheifetz, 2005	325	854	0.38	[0.35; 0.41]
Ciancaglini, 2008	155	489	0.32	[0.28; 0.36]
Clementino, 2017	48	148	0.32	[0.25; 0.40]
Colonna, 2021	209	506	0.41	[0.37; 0.46]
Costa, 2021	225	475	0.47	[0.43; 0.52]
Coutinho, 2020	42	226	0.19	[0.14; 0.24]
Silva, 2023	110	739	0.15	[0.12; 0.18]
Pontes, 2019	104	1280	0.08	[0.07; 0.10]
Dantas-Neta, 2014	105	306	0.34	[0.29; 0.40]
Almeida, 2022	334	1900	0.18	[0.16; 0.19]
Holanda, 2022	72	1936	0.04	[0.03; 0.05]
Holanda, 2022	7	289	0.02	[0.01; 0.05]
Holanda, 2020	17	240	0.07	[0.04; 0.11]
Bach, 2019	88	551	0.16	[0.13; 0.19]
Siqueira, 2013	94	890	0.11	[0.09; 0.13]
Delgado-Delgado, 2020	3	59	0.05	[0.01; 0.14]
Demir, 2004	122	965	0.13	[0.11; 0.15]
Diéguez-Pérez, 2023	99	244	0.41	[0.35; 0.47]
Costa, 2023	507	2059	0.25	[0.23; 0.27]
Drumond, 2018	176	440	0.40	[0.36; 0.45]
Duarte, 2019	113	544	0.21	[0.18; 0.24]
Ekman, 2020	777	1962	0.40	[0.37; 0.42]
Emmanuelli, 2023	197	345	0.57	[0.52; 0.62]
Fonseca, 2011	26	170	0.15	[0.11; 0.22]
Farsi, 2003	93	1113	0.08	[0.07; 0.10]
Ferreira, 2016	127	496	0.26	[0.22; 0.30]
Feteih, 2006	29	385	0.08	[0.05; 0.11]
Flores, 2023	56	122	0.46	[0.37; 0.55]
Fulgencio, 2016	205	1344	0.15	[0.13; 0.17]
Gao, 2023	242	1336	0.18	[0.16; 0.20]
Garde, 2014	144	832	0.17	[0.15; 0.20]
Ghafournia, 2012	51	400	0.13	[0.10; 0.16]
Ghalebandi, 2011	247	4309	0.06	[0.05; 0.06]
Goettems, 2017	139	536	0.26	[0.22; 0.30]
Gomes, 2018	205	761	0.27	[0.24; 0.30]
Pineda, 2020	60	144	0.42	[0.34; 0.50]
Goulart, 2021	152	366	0.42	[0.37; 0.47]
Insana , 2013	2054	4841	0.42	[0.41; 0.44]
Itani, 2013	10333	99416	0.10	[0.10; 0.11]
Johansso, 2004	172	941	0.18	[0.16; 0.21]
Karagoz, 2021	55	217	0.25	[0.20; 0.32]
Kataoka, 2015	101	1503	0.07	[0.06; 0.08]
Kato , 2012	152	1930	0.08	[0.07; 0.09]
Kaya, 2022	100	250	0.40	[0.34; 0.46]
Khatami, 2006	17	90	0.19	[0.12; 0.28]
Khoury, 2016	547	6357	0.09	[0.08; 0.09]
Kilincaslan, 2014	178	3485	0.05	[0.04; 0.06]
Kim, 2017	190	936	0.20	[0.18; 0.23]
Kolak, 2022	62	178	0.35	[0.28; 0.42]
Lam, 2011	382	6471	0.06	[0.05; 0.07]
Leal, 2021	67	739	0.09	[0.07; 0.11]
le, 2022	1422	14657	0.10	[0.09; 0.10]
Lima, 2022	38	105	0.36	[0.28; 0.46]
Liu, 2006	388	5979	0.06	[0.06; 0.07]
Macfarlane, 2003	193	2137	0.09	[0.08; 0.10]
Maluly, 2020	127	1042	0.12	[0.10; 0.14]
Manfredini, 2017	408	1556	0.26	[0.24; 0.28]
Massignan, 2019	274	935	0.29	[0.26; 0.32]
Melo , 2014	33	107	0.31	[0.23; 0.40]
Miamoto , 2011	15	60	0.25	[0.16; 0.37]
Montero , 2017	106	526	0.20	[0.17; 0.24]
Nahás-Scocate, 2014	251	873	0.29	[0.26; 0.32]
Nakata, 2007	1462	2680	0.55	[0.53; 0.56]
Nazzal, 2023	11	194	0.06	[0.03; 0.10]
Nekora-Azak, 2009	535	795	0.67	[0.64; 0.70]
Nykinen, 2023	11	46	0.24	[0.14; 0.38]
Okawara, 2022	135	770	0.18	[0.15; 0.20]
Panek , 2012	24	303	0.08	[0.05; 0.12]
Pereira, 2020	22	38	0.58	[0.42; 0.72]
Ramos, 2021	555	862	0.64	[0.61; 0.68]
Rana, 2017	141	409	0.34	[0.30; 0.39]
Rao, 2011	87	147	0.59	[0.51; 0.67]
Raphael, 2015	36	46	0.78	[0.64; 0.88]
Rauch , 2023	41	166	0.25	[0.19; 0.32]
Renner, 2011	491	1674	0.29	[0.27; 0.32]
Restrepo, 2016	14	37	0.38	[0.24; 0.54]
Ribeiro, 2018	69	207	0.33	[0.27; 0.40]
Rintakoski, 2012	1003	3126	0.32	[0.30; 0.34]
Sousa, 2018	132	594	0.22	[0.19; 0.26]
Selms, 2019	455	1431	0.32	[0.29; 0.34]
Selms, 2019	220	1131	0.19	[0.17; 0.22]
Seraj, 2010	157	600	0.26	[0.23; 0.30]
Serra-Negra, 2021	21	69	0.30	[0.21; 0.42]
Serra-Negra, 2021	48	136	0.35	[0.28; 0.44]
Serra-Negra, 2009	230	652	0.35	[0.32; 0.39]
Shahbour, 2022	21	1000	0.02	[0.01; 0.03]
Prakash, 2022	102	600	0.17	[0.14; 0.20]
Shokry , 2016	168	549	0.31	[0.27; 0.35]
Silva, 2016	31	134	0.23	[0.17; 0.31]
Silva, 2016	16	132	0.12	[0.08; 0.19]
Siva, 2021	280	6122	0.05	[0.04; 0.05]
Soares, 2017	80	253	0.32	[0.26; 0.38]
Soares, 2018	74	429	0.17	[0.14; 0.21]
Somay, 2020	24	69	0.35	[0.25; 0.47]
Suwa, 2009	355	1956	0.18	[0.17; 0.20]
Tachibana, 2016	1263	6023	0.21	[0.20; 0.22]
Tay, 2020	114	2417	0.05	[0.04; 0.06]
Phuong, 2020	291	568	0.51	[0.47; 0.55]
Toyama, 2020	112	1781	0.06	[0.05; 0.08]
Traebert, 2020	41	389	0.11	[0.08; 0.14]
Tsuchiya, 2022	20344	90148	0.23	[0.22; 0.23]
Uca, 2015	46	301	0.15	[0.12; 0.20]
Uma, 2021	90	464	0.19	[0.16; 0.23]
Unell, 2011	1635	9093	0.18	[0.17; 0.19]
Us, 2021	160	500	0.32	[0.28; 0.36]
Vieira, 2020	114	149	0.77	[0.69; 0.83]
Vieira-Andrade, 2014	103	749	0.14	[0.11; 0.16]
Vlăduțu, 2022	129	328	0.39	[0.34; 0.45]
Yachida, 2016	40	115	0.35	[0.27; 0.44]
Yeler, 2016	147	519	0.28	[0.25; 0.32]
Yıldırım, 2021	51	212	0.24	[0.19; 0.30]
Zani, 2019	11	30	0.37	[0.22; 0.55]
Arsan, 2022	333	1688	0.20	[0.18; 0.22]
Prado , 2019	204	1325	0.15	[0.14; 0.17]
Prado , 2019	318	1325	0.24	[0.22; 0.26]
Nagamatsu-Sakaguchi , 2008	80	127	0.63	[0.54; 0.71]
Antunes, 2016	21	61	0.34	[0.24; 0.47]
Hilgenberg-Sydney, 2022	24	50	0.48	[0.35; 0.61]
**Random effects model**		**356423**	**0.22**	**[0.20; 0.25]**
**Prediction interval**				**[0.04; 0.65]**

Heterogeneity: I^2 = 99%, τ^2 = 0.8835, p = 0

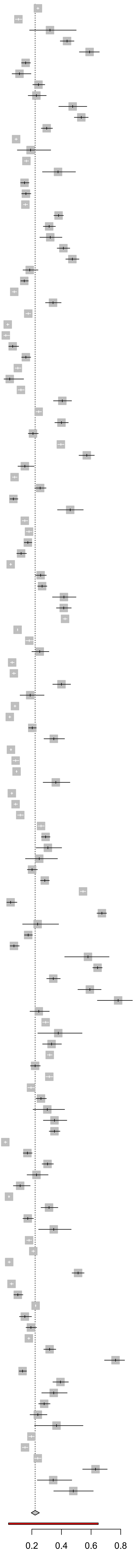

Figure S2. Global prevalence of bruxism regardless of type by continent.

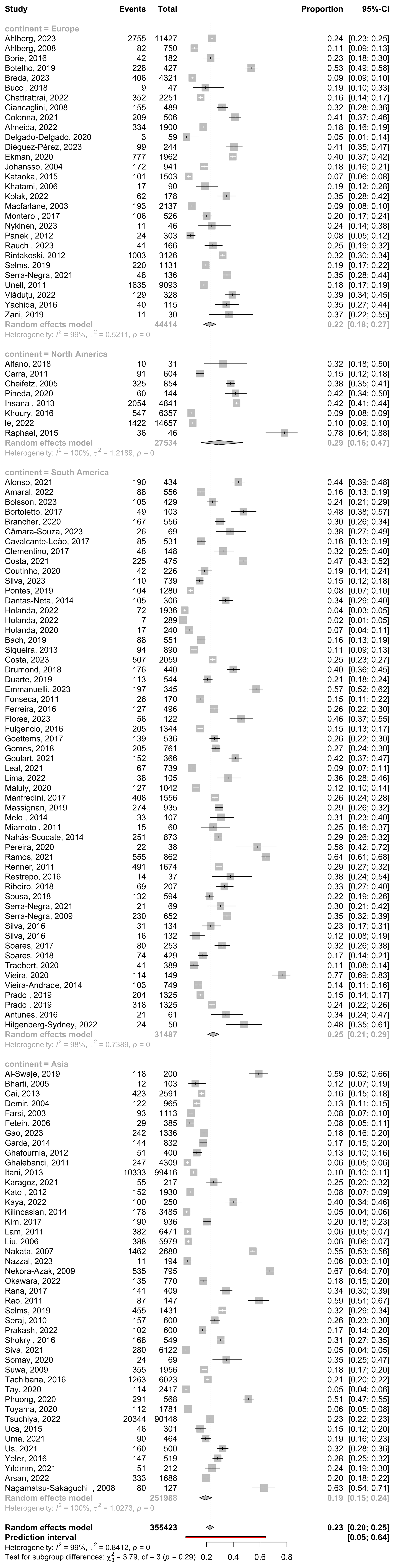
Study Events Total Proportion 95%-CI
continent = Europe
Ahlberg, 2023 2755 11427 0.24 [0.23; 0.25]
Ahlberg, 2008 82 750 0.11 [0.09; 0.13]
Borie, 2016 42 182 0.23 [0.18; 0.30]
Botelho, 2019 228 427 0.53 [0.49; 0.58]
Breda, 2023 406 4321 0.09 [0.09; 0.10]
Bucci, 2018 9 47 0.19 [0.10; 0.33]
Chattrattrai, 2022 352 2251 0.16 [0.14; 0.17]
Ciancaglini, 2008 155 489 0.32 [0.28; 0.36]
Colonna, 2021 209 506 0.41 [0.37; 0.46]
Almeida, 2022 334 1900 0.18 [0.16; 0.19]
Delgado-Delgado, 2020 3 59 0.05 [0.01; 0.14]
Diéguez-Pérez, 2023 99 244 0.41 [0.35; 0.47]
Ekman, 2020 777 1962 0.40 [0.37; 0.42]
Johansso, 2004 172 941 0.18 [0.16; 0.21]
Kataoka, 2015 101 1503 0.07 [0.06; 0.08]
Khatami, 2006 17 90 0.19 [0.12; 0.28]
Kolak, 2022 62 178 0.35 [0.28; 0.42]
Macfarlane, 2003 193 2137 0.09 [0.08; 0.10]
Montero , 2017 106 526 0.20 [0.17; 0.24]
Nykinen, 2023 11 46 0.24 [0.14; 0.38]
Panek , 2012 24 303 0.08 [0.05; 0.12]
Rauch , 2023 41 166 0.25 [0.19; 0.32]
Rintakoski, 2012 1003 3126 0.32 [0.30; 0.34]
Selms, 2019 220 1131 0.19 [0.17; 0.22]
Serra-Negra, 2021 48 136 0.35 [0.28; 0.44]
Unell, 2011 1635 9093 0.18 [0.17; 0.19]
Vlăduțu, 2022 129 328 0.39 [0.34; 0.45]
Yachida, 2016 40 115 0.35 [0.27; 0.44]
Zani, 2019 11 30 0.37 [0.22; 0.55]
Random effects model 44414 0.22 [0.18; 0.27]
Heterogeneity: I^2 = 99%, τ^2 = 0.5211, p = 0
continent = North America
Alfano, 2018 10 31 0.32 [0.18; 0.50]
Carra, 2011 91 604 0.15 [0.12; 0.18]
Cheifetz, 2005 325 854 0.38 [0.35; 0.41]
Pineda, 2020 60 144 0.42 [0.34; 0.50]
Insana , 2013 2054 4841 0.42 [0.41; 0.44]
Khoury, 2016 547 6357 0.09 [0.08; 0.09]
Ie, 2022 1422 14657 0.10 [0.09; 0.10]
Raphael, 2015 36 46 0.78 [0.64; 0.88]
Random effects model 27534 0.29 [0.16; 0.47]
Heterogeneity: I^2 = 100%, τ^2 = 1.2189, p = 0
continent = South America
Alonso, 2021 190 434 0.44 [0.39; 0.48]
Amaral, 2022 88 556 0.16 [0.13; 0.19]
Bolsson, 2023 105 429 0.24 [0.21; 0.29]
Bortoletto, 2017 49 103 0.48 [0.38; 0.57]
Brancher, 2020 167 556 0.30 [0.26; 0.34]
Câmara-Souza, 2023 26 69 0.38 [0.27; 0.49]
Cavalcante-Leão, 2017 85 531 0.16 [0.13; 0.19]
Clementino, 2017 48 148 0.32 [0.25; 0.40]
Costa, 2021 225 475 0.47 [0.43; 0.52]
Coutinho, 2020 42 226 0.19 [0.14; 0.24]
Silva, 2023 110 739 0.15 [0.12; 0.18]
Pontes, 2019 104 1280 0.08 [0.07; 0.10]
Dantas-Neta, 2014 105 306 0.34 [0.29; 0.40]
Holanda, 2022 72 1936 0.04 [0.03; 0.05]
Holanda, 2022 7 289 0.02 [0.01; 0.05]
Holanda, 2020 17 240 0.07 [0.04; 0.11]
Bach, 2019 88 551 0.16 [0.13; 0.19]
Siqueira, 2013 94 890 0.11 [0.09; 0.13]
Costa, 2023 507 2059 0.25 [0.23; 0.27]
Drumond, 2018 176 440 0.40 [0.36; 0.45]
Duarte, 2019 113 544 0.21 [0.18; 0.24]
Emmanuelli, 2023 197 345 0.57 [0.52; 0.62]
Fonseca, 2011 26 170 0.15 [0.11; 0.22]
Ferreira, 2016 127 496 0.26 [0.22; 0.30]
Flores, 2023 56 122 0.46 [0.37; 0.55]
Fulgencio, 2016 205 1344 0.15 [0.13; 0.17]
Goettems, 2017 139 536 0.26 [0.22; 0.30]
Gomes, 2018 205 761 0.27 [0.24; 0.30]
Goulart, 2021 152 366 0.42 [0.37; 0.47]
Leal, 2021 67 739 0.09 [0.07; 0.11]
Lima, 2022 38 105 0.36 [0.28; 0.46]
Maluly, 2020 127 1042 0.12 [0.10; 0.14]
Manfredini, 2017 408 1556 0.26 [0.24; 0.28]
Massignan, 2019 274 935 0.29 [0.26; 0.32]
Melo , 2014 33 107 0.31 [0.23; 0.40]
Miamoto , 2011 15 60 0.25 [0.16; 0.37]
Nahás-Scocate, 2014 251 873 0.29 [0.26; 0.32]
Pereira, 2020 22 38 0.58 [0.42; 0.72]
Ramos, 2021 555 862 0.64 [0.61; 0.68]
Renner, 2011 491 1674 0.29 [0.27; 0.32]
Restrepo, 2016 14 37 0.38 [0.24; 0.54]
Ribeiro, 2018 69 207 0.33 [0.27; 0.40]
Sousa, 2018 132 594 0.22 [0.19; 0.26]
Serra-Negra, 2021 21 69 0.30 [0.21; 0.42]
Serra-Negra, 2009 230 652 0.35 [0.32; 0.39]
Silva, 2016 31 134 0.23 [0.17; 0.31]
Silva, 2016 16 132 0.12 [0.08; 0.19]
Soares, 2017 80 253 0.32 [0.26; 0.38]
Soares, 2018 74 429 0.17 [0.14; 0.21]
Traebert, 2020 41 389 0.11 [0.08; 0.14]
Vieira, 2020 114 149 0.77 [0.69; 0.83]
Vieira-Andrade, 2014 103 749 0.14 [0.11; 0.16]
Prado , 2019 204 1325 0.15 [0.14; 0.17]
Prado , 2019 318 1325 0.24 [0.22; 0.26]
Antunes, 2016 21 61 0.34 [0.24; 0.47]
Hilgenberg-Sydney, 2022 24 50 0.48 [0.35; 0.61]
Random effects model 31487 0.25 [0.21; 0.29]
Heterogeneity: I^2 = 98%, τ^2 = 0.7389, p = 0
continent = Asia
Al-Swaje, 2019 118 200 0.59 [0.52; 0.66]
Bharti, 2005 12 103 0.12 [0.07; 0.19]
Cai, 2013 423 2591 0.16 [0.15; 0.18]
Demir, 2004 122 965 0.13 [0.11; 0.15]
Farsi, 2003 93 1113 0.08 [0.07; 0.10]
Feteih, 2006 29 385 0.08 [0.05; 0.11]
Gao, 2023 242 1336 0.18 [0.16; 0.20]
Garde, 2014 144 832 0.17 [0.15; 0.20]
Ghafournia, 2012 51 400 0.13 [0.10; 0.16]
Ghalebandi, 2011 247 4309 0.06 [0.05; 0.06]
Itani, 2013 10333 99416 0.10 [0.10; 0.11]
Karagoz, 2021 55 217 0.25 [0.20; 0.32]
Kato , 2012 152 1930 0.08 [0.07; 0.09]
Kaya, 2022 100 250 0.40 [0.34; 0.46]
Kilincaslan, 2014 178 3485 0.05 [0.04; 0.06]
Kim, 2017 190 936 0.20 [0.18; 0.23]
Lam, 2011 382 6471 0.06 [0.05; 0.07]
Liu, 2006 388 5979 0.06 [0.06; 0.07]
Nakata, 2007 1462 2680 0.55 [0.53; 0.56]
Nazzal, 2023 11 194 0.06 [0.03; 0.10]
Nekora-Azak, 2009 535 795 0.67 [0.64; 0.70]
Okawara, 2022 135 770 0.18 [0.15; 0.20]
Rana, 2017 141 409 0.34 [0.30; 0.39]
Rao, 2011 87 147 0.59 [0.51; 0.67]
Selms, 2019 455 1431 0.32 [0.29; 0.34]
Seraj, 2010 157 600 0.26 [0.23; 0.30]
Prakash, 2022 102 600 0.17 [0.14; 0.20]
Shokry , 2016 168 549 0.31 [0.27; 0.35]
Siva, 2021 280 6122 0.05 [0.04; 0.05]
Somay, 2020 24 69 0.35 [0.25; 0.47]
Suwa, 2009 355 1956 0.18 [0.17; 0.20]
Tachibana, 2016 1263 6023 0.21 [0.20; 0.22]
Tay, 2020 114 2417 0.05 [0.04; 0.06]
Phuong, 2020 291 568 0.51 [0.47; 0.55]
Toyama, 2020 112 1781 0.06 [0.05; 0.08]
Tsuchiya, 2022 20344 90148 0.23 [0.22; 0.23]
Uca, 2015 46 301 0.15 [0.12; 0.20]
Uma, 2021 90 464 0.19 [0.16; 0.23]
Us, 2021 160 500 0.32 [0.28; 0.36]
Yeler, 2016 147 519 0.28 [0.25; 0.32]
Yıldırım, 2021 51 212 0.24 [0.19; 0.30]
Arsan, 2022 333 1688 0.20 [0.18; 0.22]
Nagamatsu-Sakaguchi , 2008 80 127 0.63 [0.54; 0.71]
Random effects model 251988 0.19 [0.15; 0.24]
Heterogeneity: I^2 = 100%, τ^2 = 1.0273, p = 0
Random effects model 355423 0.23 [0.20; 0.25]
Prediction interval [0.05; 0.64]
Heterogeneity: I^2 = 99%, τ^2 = 0.8412, p = 0
Test for subgroup differences: χ^2_3 = 3.79, df = 3 (p = 0.29)
0.2 0.4 0.6 0.8

Figure S3. Global prevalence of sleep bruxism.

Study	Events	Total	Proportion	95%-CI
Aguiar, 2018	17	152	0.11	[0.07; 0.17]
Ahlberg, 2023	2755	11427	0.24	[0.23; 0.25]
Ahlberg, 2008	82	750	0.11	[0.09; 0.13]
Alfano, 2018	10	31	0.32	[0.18; 0.50]
Almutairi, 2021	446	1403	0.32	[0.29; 0.34]
Amaral, 2022	88	556	0.16	[0.13; 0.19]
Azodo, 2016	22	578	0.04	[0.02; 0.06]
Başpınar, 2023	103	249	0.41	[0.35; 0.48]
Berger, 2017	94	508	0.19	[0.15; 0.22]
Bharti, 2005	12	103	0.12	[0.07; 0.19]
Bolsson, 2023	105	429	0.24	[0.21; 0.29]
Borie, 2016	42	182	0.23	[0.18; 0.30]
Brancher, 2020	167	556	0.30	[0.26; 0.34]
Breda, 2023	406	4321	0.09	[0.09; 0.10]
Bucci, 2018	9	47	0.19	[0.10; 0.33]
Cai, 2013	423	2591	0.16	[0.15; 0.18]
Carra, 2011	91	604	0.15	[0.12; 0.18]
Cavalcante-Leão, 2017	85	531	0.16	[0.13; 0.19]
Cavallo, 2016	88	278	0.32	[0.26; 0.37]
Chattrattrai, 2022	352	2251	0.16	[0.14; 0.17]
Clementino, 2017	48	148	0.32	[0.25; 0.40]
Colonna, 2021	209	506	0.41	[0.37; 0.46]
Costa, 2021	225	475	0.47	[0.43; 0.52]
Coutinho, 2020	42	226	0.19	[0.14; 0.24]
Pontes, 2019	104	1280	0.08	[0.07; 0.10]
Dantas-Neta, 2014	40	306	0.13	[0.10; 0.17]
Almeida, 2022	334	1900	0.18	[0.16; 0.19]
Holanda, 2022	72	1936	0.04	[0.03; 0.05]
Holanda, 2022	7	289	0.02	[0.01; 0.05]
Holanda, 2020	17	240	0.07	[0.04; 0.11]
Bach, 2019	88	551	0.16	[0.13; 0.19]
Siqueira, 2013	94	890	0.11	[0.09; 0.13]
Delgado-Delgado, 2020	3	59	0.05	[0.01; 0.14]
Demir, 2004	122	965	0.13	[0.11; 0.15]
Diéguez-Pérez, 2023	99	244	0.41	[0.35; 0.47]
Costa, 2023	507	2059	0.25	[0.23; 0.27]
Drumond, 2018	176	440	0.40	[0.36; 0.45]
Duarte, 2019	113	544	0.21	[0.18; 0.24]
Ekman, 2020	714	1962	0.36	[0.34; 0.39]
Eli, 2022	87	304	0.29	[0.24; 0.34]
Emmanuelli, 2023	197	345	0.57	[0.52; 0.62]
Emodi-Perlman, 2020	94	700	0.13	[0.11; 0.16]
Emodi-Perlman, 2020	311	1092	0.28	[0.26; 0.31]
Emodi-Perlman, 2016	92	1000	0.09	[0.08; 0.11]
Fonseca, 2011	26	170	0.15	[0.11; 0.22]
Fan, 2018	289	912	0.32	[0.29; 0.35]
Ferreira, 2016	127	496	0.26	[0.22; 0.30]
Flores, 2023	56	122	0.46	[0.37; 0.55]
Flueraşu, 2022	101	308	0.33	[0.28; 0.38]
Fulgencio, 2016	205	1344	0.15	[0.13; 0.17]
Gao, 2023	242	1336	0.18	[0.16; 0.20]
Ghalebandi, 2011	247	4309	0.06	[0.05; 0.06]
Goettems, 2017	139	536	0.26	[0.22; 0.30]
Gomes, 2018	205	761	0.27	[0.24; 0.30]
Goulart, 2021	149	366	0.41	[0.36; 0.46]
Hermesh, 2014	3	33	0.09	[0.02; 0.24]
Huhtela, 2021	3126	7253	0.43	[0.42; 0.44]
Insana , 2013	2054	4841	0.42	[0.41; 0.44]
Itani, 2013	10333	99416	0.10	[0.10; 0.11]
Johansso, 2004	172	941	0.18	[0.16; 0.21]
Jokubauskas, 2019	102	228	0.45	[0.38; 0.51]
Junqueira , 2013	256	937	0.27	[0.25; 0.30]
Karagoz, 2021	55	217	0.25	[0.20; 0.32]
Kataoka, 2015	101	1503	0.07	[0.06; 0.08]
Kato , 2012	152	1930	0.08	[0.07; 0.09]
Khatami, 2006	17	90	0.19	[0.12; 0.28]
Khayat, 2019	43	149	0.29	[0.22; 0.37]
Khoury, 2016	547	6357	0.09	[0.08; 0.09]
Kilincaslan, 2014	178	3485	0.05	[0.04; 0.06]
Kim, 2017	190	936	0.20	[0.18; 0.23]
Lam, 2011	382	6471	0.06	[0.05; 0.07]
Leal, 2021	67	739	0.09	[0.07; 0.11]
Levartovsky, 2022	97	387	0.25	[0.21; 0.30]
Lima, 2022	38	105	0.36	[0.28; 0.46]
Liu, 2006	388	5979	0.06	[0.06; 0.07]
Maluly, 2020	127	1042	0.12	[0.10; 0.14]
Manfredini, 2017	408	1556	0.26	[0.24; 0.28]
Manfredini , 2012	76	397	0.19	[0.16; 0.23]
Manfredini , 2012	30	219	0.14	[0.10; 0.19]
Massignan, 2019	274	935	0.29	[0.26; 0.32]
Melis, 2003	117	1014	0.12	[0.10; 0.14]
Melo , 2014	33	107	0.31	[0.23; 0.40]
Miamoto , 2011	15	60	0.25	[0.16; 0.37]
Montero , 2017	106	526	0.20	[0.17; 0.24]
Nakata, 2007	1462	2680	0.55	[0.53; 0.56]
Nazzal, 2023	11	194	0.06	[0.03; 0.10]
Souza, 2020	90	403	0.22	[0.19; 0.27]
Osses-Anguita, 2023	88	274	0.32	[0.27; 0.38]
Peixoto, 2021	372	641	0.58	[0.54; 0.62]
Perlman, 2016	63	685	0.09	[0.07; 0.12]
Prado, 2018	39	231	0.17	[0.13; 0.22]
Ramos, 2021	555	862	0.64	[0.61; 0.68]
Rana, 2017	141	409	0.34	[0.30; 0.39]
Raphael, 2015	36	46	0.78	[0.64; 0.88]
Rauch , 2023	27	166	0.16	[0.11; 0.23]
Restrepo, 2016	14	37	0.38	[0.24; 0.54]
Ribeiro, 2018	69	207	0.33	[0.27; 0.40]
Rintakoski, 2012	1003	3126	0.32	[0.30; 0.34]
Juliatte, 2022	171	384	0.45	[0.40; 0.50]
Rubin, 2018	26	153	0.17	[0.12; 0.24]
Saczuk, 2022	595	1018	0.58	[0.55; 0.61]
Sousa, 2018	132	594	0.22	[0.19; 0.26]
Selms, 2019	455	1431	0.32	[0.29; 0.34]
Selms, 2019	220	1131	0.19	[0.17; 0.22]
Serra-Negra, 2009	230	652	0.35	[0.32; 0.39]
Shahbour, 2022	21	1000	0.02	[0.01; 0.03]
Shalev-Antsel, 2023	195	587	0.33	[0.30; 0.37]
Shokry , 2016	168	549	0.31	[0.27; 0.35]
Sierwald, 2015	209	890	0.23	[0.21; 0.26]
Silva, 2016	31	134	0.23	[0.17; 0.31]
Silva, 2016	16	132	0.12	[0.08; 0.19]
Soares, 2017	80	253	0.32	[0.26; 0.38]
Soares, 2018	74	429	0.17	[0.14; 0.21]
Somay, 2020	24	69	0.35	[0.25; 0.47]
Suwa, 2009	355	1956	0.18	[0.17; 0.20]
Tachibana, 2016	1263	6023	0.21	[0.20; 0.22]
Tay, 2020	114	2417	0.05	[0.04; 0.06]
Phuong, 2020	217	568	0.38	[0.34; 0.42]
Tinastepe, 2021	61	167	0.37	[0.30; 0.44]
Toyama, 2020	112	1781	0.06	[0.05; 0.08]
Traebert, 2020	41	389	0.11	[0.08; 0.14]
Tsuchiya, 2022	20344	90148	0.23	[0.22; 0.23]
Uca, 2015	46	301	0.15	[0.12; 0.20]
Uma, 2021	202	464	0.44	[0.39; 0.48]
Unell, 2011	1635	9093	0.18	[0.17; 0.19]
Us, 2021	160	500	0.32	[0.28; 0.36]
Selms, 2012	622	4205	0.15	[0.14; 0.16]
Vieira, 2020	114	149	0.77	[0.69; 0.83]
Vieira-Andrade, 2014	103	749	0.14	[0.11; 0.16]
Vlăduţu, 2022	40	328	0.12	[0.09; 0.16]
Wetselaar, 2019	200	1209	0.17	[0.15; 0.19]
Wetselaar, 2020	43	562	0.08	[0.06; 0.10]
Prado, 2020	125	403	0.31	[0.27; 0.36]
Winocur, 2019	327	2347	0.14	[0.13; 0.15]
Winocur-Arias, 2023	72	311	0.23	[0.19; 0.28]
Yachida, 2016	40	115	0.35	[0.27; 0.44]
Yeler, 2016	147	519	0.28	[0.25; 0.32]
Yıldırım, 2021	51	212	0.24	[0.19; 0.30]
Arsan, 2022	333	1688	0.20	[0.18; 0.22]
Prado , 2019	204	1325	0.15	[0.14; 0.17]
Prado , 2019	318	1325	0.24	[0.22; 0.26]
Yoshinaka , 2010	102	664	0.15	[0.13; 0.18]
Nagamatsu-Sakaguchi , 2008	80	127	0.63	[0.54; 0.71]
Antunes, 2016	21	61	0.34	[0.24; 0.47]
**Random effects model**		**353994**	**0.21**	**[0.19; 0.24]**
**Prediction interval**				**[0.04; 0.60]**

Heterogeneity: $I^2 = 99\%$, $\tau^2 = 0.7672$, $p = 0$

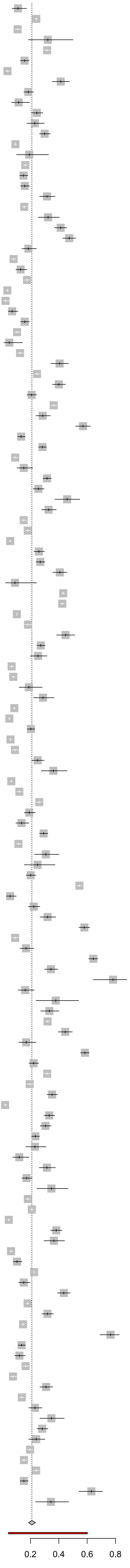

Figure S4. Global prevalence of sleep bruxism by continent.

Study	Events	Total	Proportion	95%-CI
**continent = Europe**				
Ahlberg, 2023	2755	11427	0.24	[0.23; 0.25]
Ahlberg, 2008	82	750	0.11	[0.09; 0.13]
Berger, 2017	94	508	0.19	[0.15; 0.22]
Borie, 2016	42	182	0.23	[0.18; 0.30]
Breda, 2023	406	4321	0.09	[0.09; 0.10]
Bucci, 2018	9	47	0.19	[0.10; 0.33]
Cavallo, 2016	88	278	0.32	[0.26; 0.37]
Chattrattrai, 2022	352	2251	0.16	[0.14; 0.17]
Colonna, 2021	209	506	0.41	[0.37; 0.46]
Almeida, 2022	334	1900	0.18	[0.16; 0.19]
Delgado-Delgado, 2020	3	59	0.05	[0.01; 0.14]
Diéguez-Pérez, 2023	99	244	0.41	[0.35; 0.47]
Ekman, 2020	714	1962	0.36	[0.34; 0.39]
Emodi-Perlman, 2020	311	1092	0.28	[0.26; 0.31]
Flueraşu, 2022	101	308	0.33	[0.28; 0.38]
Huhtela, 2021	3126	7253	0.43	[0.42; 0.44]
Johansso, 2004	172	941	0.18	[0.16; 0.21]
Jokubauskas, 2019	102	228	0.45	[0.38; 0.51]
Kataoka, 2015	101	1503	0.07	[0.06; 0.08]
Khatami, 2006	17	90	0.19	[0.12; 0.28]
Manfredini , 2012	30	219	0.14	[0.10; 0.19]
Melis, 2003	117	1014	0.12	[0.10; 0.14]
Montero , 2017	106	526	0.20	[0.17; 0.24]
Osses-Anguita, 2023	88	274	0.32	[0.27; 0.38]
Rauch , 2023	27	166	0.16	[0.11; 0.23]
Rintakoski, 2012	1003	3126	0.32	[0.30; 0.34]
Saczuk, 2022	595	1018	0.58	[0.55; 0.61]
Selms, 2019	220	1131	0.19	[0.17; 0.22]
Sierwald, 2015	209	890	0.23	[0.21; 0.26]
Unell, 2011	1635	9093	0.18	[0.17; 0.19]
Selms, 2012	622	4205	0.15	[0.14; 0.16]
Vlăduțu, 2022	40	328	0.12	[0.09; 0.16]
Wetselaar, 2019	200	1209	0.17	[0.15; 0.19]
Wetselaar, 2020	43	562	0.08	[0.06; 0.10]
Yachida, 2016	40	115	0.35	[0.27; 0.44]
**Random effects model**		**59726**	**0.21**	**[0.18; 0.26]**
Heterogeneity: $I^2 = 99\%$, $\tau^2 = 0.4909$, $p = 0$				
**continent = North America**				
Alfano, 2018	10	31	0.32	[0.18; 0.50]
Carra, 2011	91	604	0.15	[0.12; 0.18]
Insana , 2013	2054	4841	0.42	[0.41; 0.44]
Khoury, 2016	547	6357	0.09	[0.08; 0.09]
Raphael, 2015	36	46	0.78	[0.64; 0.88]
**Random effects model**		**11879**	**0.31**	**[0.13; 0.57]**
Heterogeneity: $I^2 = 100\%$, $\tau^2 = 1.5240$, $p = 0$				
**continent = South America**				
Aguiar, 2018	17	152	0.11	[0.07; 0.17]
Amaral, 2022	88	556	0.16	[0.13; 0.19]
Bolsson, 2023	105	429	0.24	[0.21; 0.29]
Brancher, 2020	167	556	0.30	[0.26; 0.34]
Cavalcante-Leão, 2017	85	531	0.16	[0.13; 0.19]
Clementino, 2017	48	148	0.32	[0.25; 0.40]
Costa, 2021	225	475	0.47	[0.43; 0.52]
Coutinho, 2020	42	226	0.19	[0.14; 0.24]
Pontes, 2019	104	1280	0.08	[0.07; 0.10]
Dantas-Neta, 2014	40	306	0.13	[0.10; 0.17]
Holanda, 2022	72	1936	0.04	[0.03; 0.05]
Holanda, 2022	7	289	0.02	[0.01; 0.05]
Holanda, 2020	17	240	0.07	[0.04; 0.11]
Bach, 2019	88	551	0.16	[0.13; 0.19]
Siqueira, 2013	94	890	0.11	[0.09; 0.13]
Costa, 2023	507	2059	0.25	[0.23; 0.27]
Drumond, 2018	176	440	0.40	[0.36; 0.45]
Duarte, 2019	113	544	0.21	[0.18; 0.24]
Emmanuelli, 2023	197	345	0.57	[0.52; 0.62]
Fonseca, 2011	26	170	0.15	[0.11; 0.22]
Ferreira, 2016	127	496	0.26	[0.22; 0.30]
Flores, 2023	56	122	0.46	[0.37; 0.55]
Fulgencio, 2016	205	1344	0.15	[0.13; 0.17]
Goettems, 2017	139	536	0.26	[0.22; 0.30]
Gomes, 2018	205	761	0.27	[0.24; 0.30]
Goulart, 2021	149	366	0.41	[0.36; 0.46]
Junqueira , 2013	256	937	0.27	[0.25; 0.30]
Leal, 2021	67	739	0.09	[0.07; 0.11]
Lima, 2022	38	105	0.36	[0.28; 0.46]
Maluly, 2020	127	1042	0.12	[0.10; 0.14]
Manfredini, 2017	408	1556	0.26	[0.24; 0.28]
Massignan, 2019	274	935	0.29	[0.26; 0.32]
Melo , 2014	33	107	0.31	[0.23; 0.40]
Miamoto , 2011	15	60	0.25	[0.16; 0.37]
Souza, 2020	90	403	0.22	[0.19; 0.27]
Peixoto, 2021	372	641	0.58	[0.54; 0.62]
Prado, 2018	39	231	0.17	[0.13; 0.22]
Ramos, 2021	555	862	0.64	[0.61; 0.68]
Restrepo, 2016	14	37	0.38	[0.24; 0.54]
Ribeiro, 2018	69	207	0.33	[0.27; 0.40]
Juliatte, 2022	171	384	0.45	[0.40; 0.50]
Sousa, 2018	132	594	0.22	[0.19; 0.26]
Serra-Negra, 2009	230	652	0.35	[0.32; 0.39]
Silva, 2016	31	134	0.23	[0.17; 0.31]
Silva, 2016	16	132	0.12	[0.08; 0.19]
Soares, 2017	80	253	0.32	[0.26; 0.38]
Soares, 2018	74	429	0.17	[0.14; 0.21]
Traebert, 2020	41	389	0.11	[0.08; 0.14]
Vieira, 2020	114	149	0.77	[0.69; 0.83]
Vieira-Andrade, 2014	103	749	0.14	[0.11; 0.16]
Prado, 2020	125	403	0.31	[0.27; 0.36]
Prado , 2019	204	1325	0.15	[0.14; 0.17]
Prado , 2019	318	1325	0.24	[0.22; 0.26]
Antunes, 2016	21	61	0.34	[0.24; 0.47]
**Random effects model**		**30589**	**0.23**	**[0.19; 0.28]**
Heterogeneity: $I^2 = 98\%$, $\tau^2 = 0.7461$, $p = 0$				
**continent = Asia**				
Almutairi, 2021	446	1403	0.32	[0.29; 0.34]
Başpınar, 2023	103	249	0.41	[0.35; 0.48]
Bharti, 2005	12	103	0.12	[0.07; 0.19]
Cai, 2013	423	2591	0.16	[0.15; 0.18]
Demir, 2004	122	965	0.13	[0.11; 0.15]
Eli, 2022	87	304	0.29	[0.24; 0.34]
Emodi-Perlman, 2020	94	700	0.13	[0.11; 0.16]
Emodi-Perlman, 2016	92	1000	0.09	[0.08; 0.11]
Gao, 2023	242	1336	0.18	[0.16; 0.20]
Ghalebandi, 2011	247	4309	0.06	[0.05; 0.06]
Hermesh, 2014	3	33	0.09	[0.02; 0.24]
Itani, 2013	10333	99416	0.10	[0.10; 0.11]
Karagoz, 2021	55	217	0.25	[0.20; 0.32]
Kato , 2012	152	1930	0.08	[0.07; 0.09]
Khayat, 2019	43	149	0.29	[0.22; 0.37]
Kilincaslan, 2014	178	3485	0.05	[0.04; 0.06]
Kim, 2017	190	936	0.20	[0.18; 0.23]
Lam, 2011	382	6471	0.06	[0.05; 0.07]
Levartovsky, 2022	97	387	0.25	[0.21; 0.30]
Liu, 2006	388	5979	0.06	[0.06; 0.07]
Manfredini , 2012	76	397	0.19	[0.16; 0.23]
Nakata, 2007	1462	2680	0.55	[0.53; 0.56]
Nazzal, 2023	11	194	0.06	[0.03; 0.10]
Perlman, 2016	63	685	0.09	[0.07; 0.12]
Rana, 2017	141	409	0.34	[0.30; 0.39]
Selms, 2019	455	1431	0.32	[0.29; 0.34]
Shalev-Antsel, 2023	195	587	0.33	[0.30; 0.37]
Shokry , 2016	168	549	0.31	[0.27; 0.35]
Somay, 2020	24	69	0.35	[0.25; 0.47]
Suwa, 2009	355	1956	0.18	[0.17; 0.20]
Tachibana, 2016	1263	6023	0.21	[0.20; 0.22]
Tay, 2020	114	2417	0.05	[0.04; 0.06]
Phuong, 2020	217	568	0.38	[0.34; 0.42]
Tinastepe, 2021	61	167	0.37	[0.30; 0.44]
Toyama, 2020	112	1781	0.06	[0.05; 0.08]
Tsuchiya, 2022	20344	90148	0.23	[0.22; 0.23]
Uca, 2015	46	301	0.15	[0.12; 0.20]
Uma, 2021	202	464	0.44	[0.39; 0.48]
Us, 2021	160	500	0.32	[0.28; 0.36]
Winocur, 2019	327	2347	0.14	[0.13; 0.15]
Winocur-Arias, 2023	72	311	0.23	[0.19; 0.28]
Yeler, 2016	147	519	0.28	[0.25; 0.32]
Yıldırım, 2021	51	212	0.24	[0.19; 0.30]
Arsan, 2022	333	1688	0.20	[0.18; 0.22]
Yoshinaka , 2010	102	664	0.15	[0.13; 0.18]
Nagamatsu-Sakaguchi , 2008	80	127	0.63	[0.54; 0.71]
**Random effects model**		**249157**	**0.19**	**[0.15; 0.23]**
Heterogeneity: $I^2 = 100\%$, $\tau^2 = 0.7170$, $p = 0$				
**Random effects model**		**351351**	**0.21**	**[0.19; 0.24]**
**Prediction interval**				**[0.05; 0.59]**
Heterogeneity: $I^2 = 99\%$, $\tau^2 = 0.7160$, $p = 0$				
Test for subgroup differences: $\chi^2_3 = 3.07$, df = 3 ($p = 0.38$)				

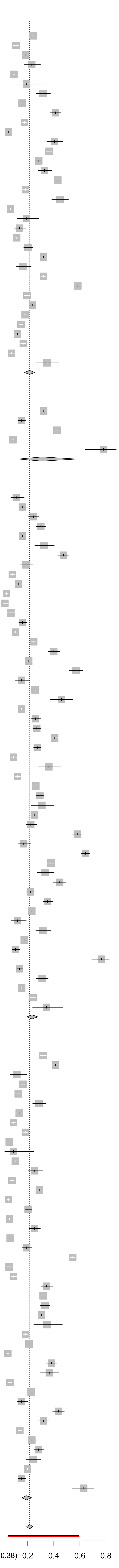

Figure S6. Global prevalence of sleep bruxism in the female population.

Study	Events	Total	Proportion	95%-CI
Ahlberg, 2023	1397	11427	0.12	[0.12; 0.13]
Almutairi, 2021	244	1403	0.17	[0.15; 0.19]
Amaral, 2022	35	556	0.06	[0.05; 0.09]
Azodo, 2016	12	578	0.02	[0.01; 0.04]
Berger, 2017	59	508	0.12	[0.09; 0.15]
Borie, 2016	27	182	0.15	[0.10; 0.21]
Brancher, 2020	84	556	0.15	[0.12; 0.18]
Breda, 2023	176	4321	0.04	[0.04; 0.05]
Cai, 2013	423	2591	0.16	[0.15; 0.18]
Cavallo, 2016	54	278	0.19	[0.15; 0.24]
Chattrattrai, 2022	279	2251	0.12	[0.11; 0.14]
Clementino, 2017	31	148	0.21	[0.15; 0.28]
Costa, 2021	108	475	0.23	[0.19; 0.27]
Dantas-Neta, 2014	22	306	0.07	[0.05; 0.11]
Almeida, 2022	152	1900	0.08	[0.07; 0.09]
Holanda, 2022	72	1936	0.04	[0.03; 0.05]
Holanda, 2022	7	289	0.02	[0.01; 0.05]
Holanda, 2020	11	240	0.05	[0.02; 0.08]
Bach, 2019	35	551	0.06	[0.05; 0.09]
Demir, 2004	61	965	0.06	[0.05; 0.08]
Costa, 2023	285	2059	0.14	[0.12; 0.15]
Drumond, 2018	86	440	0.20	[0.16; 0.24]
Duarte, 2019	65	544	0.12	[0.09; 0.15]
Ekman, 2020	429	1962	0.22	[0.20; 0.24]
Emmanuelli, 2023	164	345	0.48	[0.42; 0.53]
Fonseca, 2011	11	170	0.06	[0.04; 0.11]
Fan, 2018	146	912	0.16	[0.14; 0.19]
Flueraşu, 2022	51	308	0.17	[0.13; 0.21]
Gao, 2023	105	1336	0.08	[0.07; 0.09]
Ghalebandi, 2011	136	4309	0.03	[0.03; 0.04]
Goettems, 2017	62	536	0.12	[0.09; 0.15]
Huhtela, 2021	2294	7253	0.32	[0.31; 0.33]
Insana , 2013	733	4841	0.15	[0.14; 0.16]
Itani, 2013	5535	99416	0.06	[0.05; 0.06]
Johansso, 2004	100	941	0.11	[0.09; 0.13]
Jokubauskas, 2019	80	228	0.35	[0.29; 0.41]
Karagoz, 2021	43	217	0.20	[0.15; 0.26]
Kataoka, 2015	52	1503	0.03	[0.03; 0.05]
Kato , 2012	65	1930	0.03	[0.03; 0.04]
Lam, 2011	150	6471	0.02	[0.02; 0.03]
Leal, 2021	32	739	0.04	[0.03; 0.06]
Liu, 2006	179	5979	0.03	[0.03; 0.03]
Maluly, 2020	80	1042	0.08	[0.06; 0.09]
Manfredini, 2017	192	1556	0.12	[0.11; 0.14]
Melis, 2003	66	1014	0.07	[0.05; 0.08]
Nakata, 2007	327	2680	0.12	[0.11; 0.13]
Perlman, 2016	43	685	0.06	[0.05; 0.08]
Prado, 2018	21	231	0.09	[0.06; 0.14]
Ramos, 2021	272	862	0.32	[0.29; 0.35]
Raphael, 2015	36	46	0.78	[0.64; 0.88]
Ribeiro, 2018	62	207	0.30	[0.24; 0.37]
Rintakoski, 2012	558	3126	0.18	[0.17; 0.19]
Juliatte, 2022	144	384	0.38	[0.33; 0.42]
Rubin, 2018	12	153	0.08	[0.04; 0.13]
Saczuk, 2022	482	1018	0.47	[0.44; 0.50]
Sousa, 2018	71	594	0.12	[0.10; 0.15]
Serra-Negra, 2009	130	652	0.20	[0.17; 0.23]
Shahbour, 2022	82	1000	0.08	[0.07; 0.10]
Shalev-Antsel, 2023	107	587	0.18	[0.15; 0.22]
Shokry , 2016	143	549	0.26	[0.23; 0.30]
Soares, 2018	30	429	0.07	[0.05; 0.10]
Suwa, 2009	164	1956	0.08	[0.07; 0.10]
Tay, 2020	4	2417	0.00	[0.00; 0.00]
Toyama, 2020	65	1781	0.04	[0.03; 0.05]
Traebert, 2020	20	389	0.05	[0.03; 0.08]
Tsuchiya, 2022	8946	90148	0.10	[0.10; 0.10]
Uma, 2021	128	464	0.28	[0.24; 0.32]
Unell, 2011	962	9093	0.11	[0.10; 0.11]
Us, 2021	62	500	0.12	[0.10; 0.16]
Vieira, 2020	94	149	0.63	[0.55; 0.70]
Vieira-Andrade, 2014	58	749	0.08	[0.06; 0.10]
Vlăduțu, 2022	25	328	0.08	[0.05; 0.11]
Wetselaar, 2019	125	1209	0.10	[0.09; 0.12]
Wetselaar, 2020	25	562	0.04	[0.03; 0.07]
Winocur, 2019	205	2347	0.09	[0.08; 0.10]
Winocur-Arias, 2023	36	311	0.12	[0.08; 0.16]
Yıldırım, 2021	41	212	0.19	[0.15; 0.25]
Arsan, 2022	209	1688	0.12	[0.11; 0.14]
Nagamatsu-Sakaguchi , 2008	46	127	0.36	[0.28; 0.45]
**Random effects model**		**305145**	**0.12**	**[0.10; 0.14]**
**Prediction interval**				**[0.02; 0.47]**

Heterogeneity: $I^2 = 99\%$, $\tau^2 = 0.9006$, $p = 0$

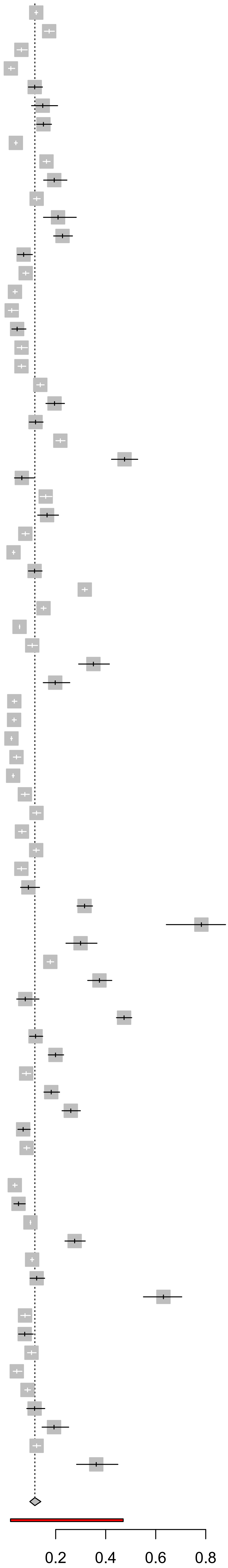

Figure S6. Global prevalence of sleep bruxism in the male population.

Study	Events	Total	Proportion	95%-CI
Ahlberg, 2023	1358	11427	0.12	[0.11; 0.12]
Almutairi, 2021	202	1403	0.14	[0.13; 0.16]
Amaral, 2022	53	556	0.10	[0.07; 0.12]
Azodo, 2016	10	578	0.02	[0.01; 0.03]
Berger, 2017	35	508	0.07	[0.05; 0.09]
Borie, 2016	15	182	0.08	[0.05; 0.13]
Brancher, 2020	83	556	0.15	[0.12; 0.18]
Breda, 2023	230	4321	0.05	[0.05; 0.06]
Cavallo, 2016	34	278	0.12	[0.09; 0.17]
Chattrattrai, 2022	73	2251	0.03	[0.03; 0.04]
Clementino, 2017	17	148	0.11	[0.07; 0.18]
Costa, 2021	117	475	0.25	[0.21; 0.29]
Dantas-Neta, 2014	18	306	0.06	[0.04; 0.09]
Almeida, 2022	182	1900	0.10	[0.08; 0.11]
Holanda, 2020	6	240	0.02	[0.01; 0.05]
Bach, 2019	53	551	0.10	[0.07; 0.12]
Demir, 2004	61	965	0.06	[0.05; 0.08]
Costa, 2023	222	2059	0.11	[0.10; 0.12]
Drumond, 2018	90	440	0.20	[0.17; 0.24]
Duarte, 2019	48	544	0.09	[0.07; 0.12]
Ekman, 2020	285	1962	0.15	[0.13; 0.16]
Emmanuelli, 2023	33	345	0.10	[0.07; 0.13]
Fonseca, 2011	15	170	0.09	[0.05; 0.14]
Fan, 2018	143	912	0.16	[0.13; 0.18]
Flueraşu, 2022	50	308	0.16	[0.13; 0.21]
Gao, 2023	137	1336	0.10	[0.09; 0.12]
Ghalebandi, 2011	111	4309	0.03	[0.02; 0.03]
Goettems, 2017	77	536	0.14	[0.12; 0.18]
Huhtela, 2021	832	7253	0.11	[0.11; 0.12]
Insana , 2013	1321	4841	0.27	[0.26; 0.29]
Itani, 2013	4798	99416	0.05	[0.05; 0.05]
Johansso, 2004	72	941	0.08	[0.06; 0.10]
Jokubauskas, 2019	22	228	0.10	[0.06; 0.14]
Karagoz, 2021	12	217	0.06	[0.03; 0.10]
Kataoka, 2015	49	1503	0.03	[0.02; 0.04]
Kato , 2012	87	1930	0.05	[0.04; 0.06]
Lam, 2011	232	6471	0.04	[0.03; 0.04]
Leal, 2021	35	739	0.05	[0.03; 0.07]
Liu, 2006	209	5979	0.03	[0.03; 0.04]
Maluly, 2020	47	1042	0.05	[0.03; 0.06]
Manfredini, 2017	216	1556	0.14	[0.12; 0.16]
Melis, 2003	51	1014	0.05	[0.04; 0.07]
Nakata, 2007	1135	2680	0.42	[0.40; 0.44]
Perlman, 2016	20	685	0.03	[0.02; 0.04]
Prado, 2018	18	231	0.08	[0.05; 0.12]
Ramos, 2021	283	862	0.33	[0.30; 0.36]
Ribeiro, 2018	7	207	0.03	[0.02; 0.07]
Rintakoski, 2012	445	3126	0.14	[0.13; 0.16]
Juliatte, 2022	27	384	0.07	[0.05; 0.10]
Rubin, 2018	21	153	0.14	[0.09; 0.20]
Saczuk, 2022	113	1018	0.11	[0.09; 0.13]
Sousa, 2018	61	594	0.10	[0.08; 0.13]
Serra-Negra, 2009	100	652	0.15	[0.13; 0.18]
Shahbour, 2022	4	1000	0.00	[0.00; 0.01]
Shalev-Antsel, 2023	88	587	0.15	[0.12; 0.18]
Shokry , 2016	25	549	0.05	[0.03; 0.07]
Soares, 2018	44	429	0.10	[0.08; 0.14]
Suwa, 2009	191	1956	0.10	[0.09; 0.11]
Tay, 2020	110	2417	0.05	[0.04; 0.05]
Toyama, 2020	47	1781	0.03	[0.02; 0.03]
Traebert, 2020	21	389	0.05	[0.04; 0.08]
Tsuchiya, 2022	11398	90148	0.13	[0.12; 0.13]
Uma, 2021	74	464	0.16	[0.13; 0.20]
Unell, 2011	673	9093	0.07	[0.07; 0.08]
Us, 2021	98	500	0.20	[0.16; 0.23]
Vieira, 2020	20	149	0.13	[0.09; 0.20]
Vieira-Andrade, 2014	45	749	0.06	[0.05; 0.08]
Vlăduțu, 2022	15	328	0.05	[0.03; 0.07]
Wetselaar, 2019	75	1209	0.06	[0.05; 0.08]
Wetselaar, 2020	18	562	0.03	[0.02; 0.05]
Winocur, 2019	122	2347	0.05	[0.04; 0.06]
Winocur-Arias, 2023	36	311	0.12	[0.08; 0.16]
Yıldırım, 2021	10	212	0.05	[0.02; 0.09]
Arsan, 2022	124	1688	0.07	[0.06; 0.09]
Nagamatsu-Sakaguchi , 2008	34	127	0.27	[0.20; 0.35]
**Random effects model**		**300283**	**0.08**	**[0.07; 0.10]**
**Prediction interval**				**[0.02; 0.28]**

Heterogeneity: $I^2 = 99\%$, $\tau^2 = 0.5274$, $p = 0$

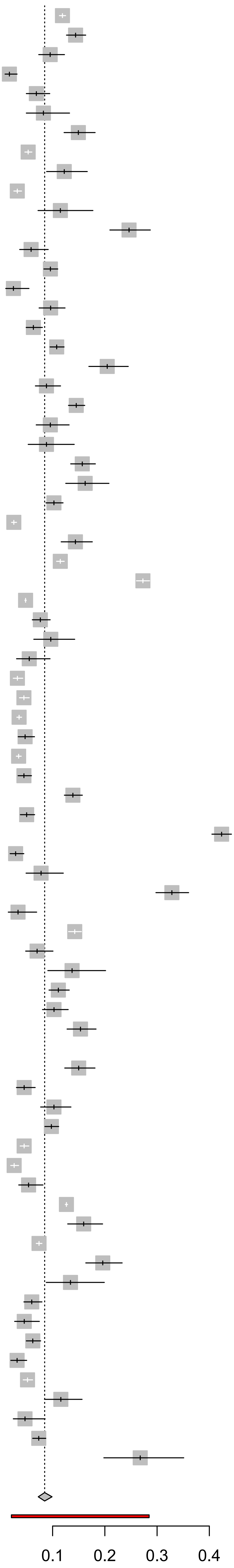

Figure S7. Global prevalence of sleep bruxism by age.

Study	Events	Total	Proportion	95%-CI
**age = minors**				
Alfano, 2018	10	31	0.32	[0.18; 0.50]
Amaral, 2022	88	556	0.16	[0.13; 0.19]
Bharti, 2005	12	103	0.12	[0.07; 0.19]
Bolsson, 2023	105	429	0.24	[0.21; 0.29]
Brancher, 2020	167	556	0.30	[0.26; 0.34]
Breda, 2023	406	4321	0.09	[0.09; 0.10]
Carra, 2011	91	604	0.15	[0.12; 0.18]
Cavalcante-Leão, 2017	85	531	0.16	[0.13; 0.19]
Clementino, 2017	48	148	0.32	[0.25; 0.40]
Costa, 2021	225	475	0.47	[0.43; 0.52]
Almeida, 2022	334	1900	0.18	[0.16; 0.19]
Holanda, 2022	7	289	0.02	[0.01; 0.05]
Bach, 2019	88	551	0.16	[0.13; 0.19]
Demir, 2004	122	965	0.13	[0.11; 0.15]
Diéguez-Pérez, 2023	99	244	0.41	[0.35; 0.47]
Drumond, 2018	176	440	0.40	[0.36; 0.45]
Duarte, 2019	113	544	0.21	[0.18; 0.24]
Emodi-Perlman, 2016	92	1000	0.09	[0.08; 0.11]
Fonseca, 2011	26	170	0.15	[0.11; 0.22]
Ferreira, 2016	127	496	0.26	[0.22; 0.30]
Fulgencio, 2016	205	1344	0.15	[0.13; 0.17]
Gao, 2023	242	1336	0.18	[0.16; 0.20]
Ghalebandi, 2011	247	4309	0.06	[0.05; 0.06]
Gomes, 2018	205	761	0.27	[0.24; 0.30]
Insana , 2013	2054	4841	0.42	[0.41; 0.44]
Itani, 2013	10333	99416	0.10	[0.10; 0.11]
Junqueira , 2013	256	937	0.27	[0.25; 0.30]
Kilincaslan, 2014	178	3485	0.05	[0.04; 0.06]
Kim, 2017	190	936	0.20	[0.18; 0.23]
Lam, 2011	382	6471	0.06	[0.05; 0.07]
Leal, 2021	67	739	0.09	[0.07; 0.11]
Lima, 2022	38	105	0.36	[0.28; 0.46]
Liu, 2006	388	5979	0.06	[0.06; 0.07]
Manfredini, 2017	408	1556	0.26	[0.24; 0.28]
Massignan, 2019	274	935	0.29	[0.26; 0.32]
Melo , 2014	33	107	0.31	[0.23; 0.40]
Miamoto , 2011	15	60	0.25	[0.16; 0.37]
Nazzal, 2023	11	194	0.06	[0.03; 0.10]
Souza, 2020	90	403	0.22	[0.19; 0.27]
Perlman, 2016	63	685	0.09	[0.07; 0.12]
Ramos, 2021	555	862	0.64	[0.61; 0.68]
Rana, 2017	141	409	0.34	[0.30; 0.39]
Restrepo, 2016	14	37	0.38	[0.24; 0.54]
Ribeiro, 2018	69	207	0.33	[0.27; 0.40]
Rubin, 2018	26	153	0.17	[0.12; 0.24]
Sousa, 2018	132	594	0.22	[0.19; 0.26]
Selms, 2019	455	1431	0.32	[0.29; 0.34]
Selms, 2019	220	1131	0.19	[0.17; 0.22]
Serra-Negra, 2009	230	652	0.35	[0.32; 0.39]
Shahbour, 2022	21	1000	0.02	[0.01; 0.03]
Silva, 2016	16	132	0.12	[0.08; 0.19]
Soares, 2018	74	429	0.17	[0.14; 0.21]
Suwa, 2009	355	1956	0.18	[0.17; 0.20]
Tachibana, 2016	1263	6023	0.21	[0.20; 0.22]
Traebert, 2020	41	389	0.11	[0.08; 0.14]
Us, 2021	160	500	0.32	[0.28; 0.36]
Selms, 2012	622	4205	0.15	[0.14; 0.16]
Vieira-Andrade, 2014	103	749	0.14	[0.11; 0.16]
Wetselaar, 2020	43	562	0.08	[0.06; 0.10]
Prado, 2020	125	403	0.31	[0.27; 0.36]
Winocur, 2019	327	2347	0.14	[0.13; 0.15]
Prado , 2019	318	1325	0.24	[0.22; 0.26]
Nagamatsu-Sakaguchi , 2008	80	127	0.63	[0.54; 0.71]
Antunes, 2016	21	61	0.34	[0.24; 0.47]
**Random effects model**		174636	0.19	[0.16; 0.23]
Heterogeneity: $I^2 = 99\%$, $\tau^2 = 0.7228$, $p = 0$				
**age = adults**				
Aguiar, 2018	17	152	0.11	[0.07; 0.17]
Ahlberg, 2023	2755	11427	0.24	[0.23; 0.25]
Ahlberg, 2008	82	750	0.11	[0.09; 0.13]
Almutairi, 2021	446	1403	0.32	[0.29; 0.34]
Azodo, 2016	22	578	0.04	[0.02; 0.06]
Başpınar, 2023	103	249	0.41	[0.35; 0.48]
Berger, 2017	94	508	0.19	[0.15; 0.22]
Borie, 2016	42	182	0.23	[0.18; 0.30]
Bucci, 2018	9	47	0.19	[0.10; 0.33]
Cai, 2013	423	2591	0.16	[0.15; 0.18]
Cavallo, 2016	88	278	0.32	[0.26; 0.37]
Chattrattrai, 2022	352	2251	0.16	[0.14; 0.17]
Colonna, 2021	209	506	0.41	[0.37; 0.46]
Coutinho, 2020	42	226	0.19	[0.14; 0.24]
Pontes, 2019	104	1280	0.08	[0.07; 0.10]
Dantas-Neta, 2014	40	306	0.13	[0.10; 0.17]
Holanda, 2022	72	1936	0.04	[0.03; 0.05]
Holanda, 2020	17	240	0.07	[0.04; 0.11]
Siqueira, 2013	94	890	0.11	[0.09; 0.13]
Delgado-Delgado, 2020	3	59	0.05	[0.01; 0.14]
Costa, 2023	507	2059	0.25	[0.23; 0.27]
Ekman, 2020	714	1962	0.36	[0.34; 0.39]
Eli, 2022	87	304	0.29	[0.24; 0.34]
Emmanuelli, 2023	197	345	0.57	[0.52; 0.62]
Emodi-Perlman, 2020	94	700	0.13	[0.11; 0.16]
Emodi-Perlman, 2020	311	1092	0.28	[0.26; 0.31]
Fan, 2018	289	912	0.32	[0.29; 0.35]
Flores, 2023	56	122	0.46	[0.37; 0.55]
Flueraşu, 2022	101	308	0.33	[0.28; 0.38]
Goettems, 2017	139	536	0.26	[0.22; 0.30]
Goulart, 2021	149	366	0.41	[0.36; 0.46]
Hermesh, 2014	3	33	0.09	[0.02; 0.24]
Huhtela, 2021	3126	7253	0.43	[0.42; 0.44]
Johansso, 2004	172	941	0.18	[0.16; 0.21]
Jokubauskas, 2019	102	228	0.45	[0.38; 0.51]
Karagoz, 2021	55	217	0.25	[0.20; 0.32]
Kataoka, 2015	101	1503	0.07	[0.06; 0.08]
Kato , 2012	152	1930	0.08	[0.07; 0.09]
Khatami, 2006	17	90	0.19	[0.12; 0.28]
Khayat, 2019	43	149	0.29	[0.22; 0.37]
Khoury, 2016	547	6357	0.09	[0.08; 0.09]
Levartovsky, 2022	97	387	0.25	[0.21; 0.30]
Maluly, 2020	127	1042	0.12	[0.10; 0.14]
Manfredini , 2012	76	397	0.19	[0.16; 0.23]
Manfredini , 2012	30	219	0.14	[0.10; 0.19]
Melis, 2003	117	1014	0.12	[0.10; 0.14]
Montero , 2017	106	526	0.20	[0.17; 0.24]
Nakata, 2007	1462	2680	0.55	[0.53; 0.56]
Osses-Anguita, 2023	88	274	0.32	[0.27; 0.38]
Peixoto, 2021	372	641	0.58	[0.54; 0.62]
Prado, 2018	39	231	0.17	[0.13; 0.22]
Raphael, 2015	36	46	0.78	[0.64; 0.88]
Rauch , 2023	27	166	0.16	[0.11; 0.23]
Rintakoski, 2012	1003	3126	0.32	[0.30; 0.34]
Juliatte, 2022	171	384	0.45	[0.40; 0.50]
Saczuk, 2022	595	1018	0.58	[0.55; 0.61]
Shalev-Antsel, 2023	195	587	0.33	[0.30; 0.37]
Shokry , 2016	168	549	0.31	[0.27; 0.35]
Sierwald, 2015	209	890	0.23	[0.21; 0.26]
Silva, 2016	31	134	0.23	[0.17; 0.31]
Soares, 2017	80	253	0.32	[0.26; 0.38]
Somay, 2020	24	69	0.35	[0.25; 0.47]
Tay, 2020	114	2417	0.05	[0.04; 0.06]
Phuong, 2020	217	568	0.38	[0.34; 0.42]
Tinastepe, 2021	61	167	0.37	[0.30; 0.44]
Toyama, 2020	112	1781	0.06	[0.05; 0.08]
Tsuchiya, 2022	20344	90148	0.23	[0.22; 0.23]
Uca, 2015	46	301	0.15	[0.12; 0.20]
Uma, 2021	202	464	0.44	[0.39; 0.48]
Unell, 2011	1635	9093	0.18	[0.17; 0.19]
Vieira, 2020	114	149	0.77	[0.69; 0.83]
Vlăduţu, 2022	40	328	0.12	[0.09; 0.16]
Wetselaar, 2019	200	1209	0.17	[0.15; 0.19]
Winocur-Arias, 2023	72	311	0.23	[0.19; 0.28]
Yachida, 2016	40	115	0.35	[0.27; 0.44]
Yeler, 2016	147	519	0.28	[0.25; 0.32]
Yıldırım, 2021	51	212	0.24	[0.19; 0.30]
Arsan, 2022	333	1688	0.20	[0.18; 0.22]
Prado , 2019	204	1325	0.15	[0.14; 0.17]
Yoshinaka , 2010	102	664	0.15	[0.13; 0.18]
**Random effects model**		179358	0.23	[0.19; 0.26]
Heterogeneity: $I^2 = 99\%$, $\tau^2 = 0.7821$, $p = 0$				
**Random effects model**		**353994**	**0.21**	**[0.19; 0.24]**
**Prediction interval**				**[0.04; 0.60]**

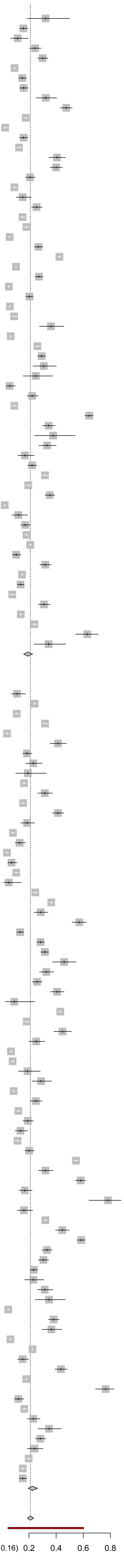

Heterogeneity: $I^2 = 99\%$, $\tau^2 = 0.7672$, $p = 0$

Test for subgroup differences: $\chi^2_1 = 2.02$, df = 1 ($p = 0.16$)

Figure S8. Global prevalence of sleep bruxism by continent and age.

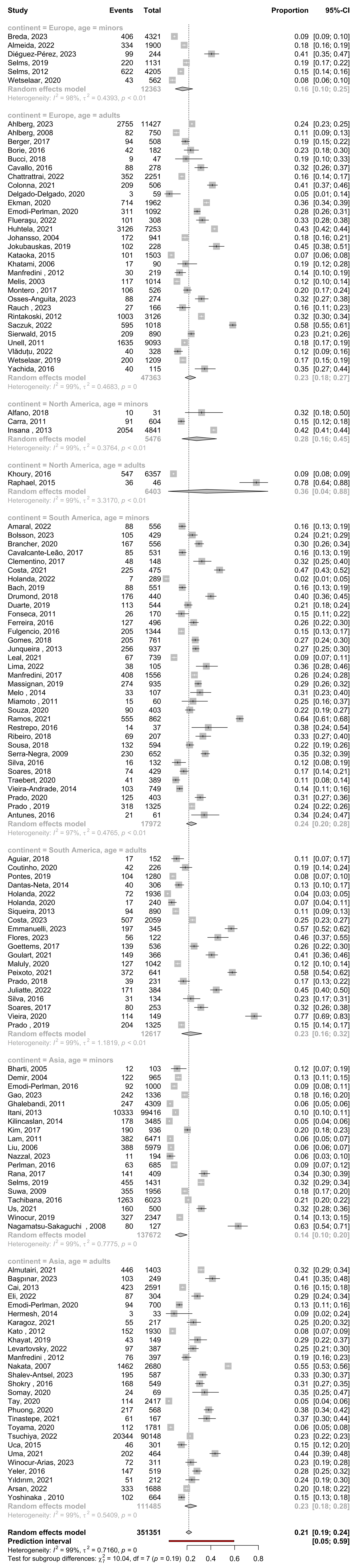

Study Events Total Proportion 95%-CI
continent = Europe, age = minors
Breda, 2023 406 4321 0.09 [0.09; 0.10]
Almeida, 2022 334 1900 0.18 [0.16; 0.19]
Diéguez-Pérez, 2023 99 244 0.41 [0.35; 0.47]
Selms, 2019 220 1131 0.19 [0.17; 0.22]
Selms, 2012 622 4205 0.15 [0.14; 0.16]
Wetselaar, 2020 43 562 0.08 [0.06; 0.10]
Random effects model 12363 0.16 [0.10; 0.25]
Heterogeneity: I^2 = 98%, τ^2 = 0.4393, p < 0.01
continent = Europe, age = adults
Ahlberg, 2023 2755 11427 0.24 [0.23; 0.25]
Ahlberg, 2008 82 750 0.11 [0.09; 0.13]
Berger, 2017 94 508 0.19 [0.15; 0.22]
Borie, 2016 42 182 0.23 [0.18; 0.30]
Bucci, 2018 9 47 0.19 [0.10; 0.33]
Cavallo, 2016 88 278 0.32 [0.26; 0.37]
Chattrattrai, 2022 352 2251 0.16 [0.14; 0.17]
Colonna, 2021 209 506 0.41 [0.37; 0.46]
Delgado-Delgado, 2020 3 59 0.05 [0.01; 0.14]
Ekman, 2020 714 1962 0.36 [0.34; 0.39]
Emodi-Perlman, 2020 311 1092 0.28 [0.26; 0.31]
Flueraşu, 2022 101 308 0.33 [0.28; 0.38]
Huhtela, 2021 3126 7253 0.43 [0.42; 0.44]
Johansso, 2004 172 941 0.18 [0.16; 0.21]
Jokubauskas, 2019 102 228 0.45 [0.38; 0.51]
Kataoka, 2015 101 1503 0.07 [0.06; 0.08]
Khatami, 2006 17 90 0.19 [0.12; 0.28]
Manfredini , 2012 30 219 0.14 [0.10; 0.19]
Melis, 2003 117 1014 0.12 [0.10; 0.14]
Montero , 2017 106 526 0.20 [0.17; 0.24]
Osses-Anguita, 2023 88 274 0.32 [0.27; 0.38]
Rauch , 2023 27 166 0.16 [0.11; 0.23]
Rintakoski, 2012 1003 3126 0.32 [0.30; 0.34]
Saczuk, 2022 595 1018 0.58 [0.55; 0.61]
Sierwald, 2015 209 890 0.23 [0.21; 0.26]
Unell, 2011 1635 9093 0.18 [0.17; 0.19]
Vlăduțu, 2022 40 328 0.12 [0.09; 0.16]
Wetselaar, 2019 200 1209 0.17 [0.15; 0.19]
Yachida, 2016 40 115 0.35 [0.27; 0.44]
Random effects model 47363 0.23 [0.18; 0.27]
Heterogeneity: I^2 = 99%, τ^2 = 0.4683, p = 0
continent = North America, age = minors
Alfano, 2018 10 31 0.32 [0.18; 0.50]
Carra, 2011 91 604 0.15 [0.12; 0.18]
Insana , 2013 2054 4841 0.42 [0.41; 0.44]
Random effects model 5476 0.28 [0.16; 0.45]
Heterogeneity: I^2 = 99%, τ^2 = 0.3764, p < 0.01
continent = North America, age = adults
Khoury, 2016 547 6357 0.09 [0.08; 0.09]
Raphael, 2015 36 46 0.78 [0.64; 0.88]
Random effects model 6403 0.36 [0.04; 0.88]
Heterogeneity: I^2 = 99%, τ^2 = 3.3170, p < 0.01
continent = South America, age = minors
Amaral, 2022 88 556 0.16 [0.13; 0.19]
Bolsson, 2023 105 429 0.24 [0.21; 0.29]
Brancher, 2020 167 556 0.30 [0.26; 0.34]
Cavalcante-Leão, 2017 85 531 0.16 [0.13; 0.19]
Clementino, 2017 48 148 0.32 [0.25; 0.40]
Costa, 2021 225 475 0.47 [0.43; 0.52]
Holanda, 2022 7 289 0.02 [0.01; 0.05]
Bach, 2019 88 551 0.16 [0.13; 0.19]
Drumond, 2018 176 440 0.40 [0.36; 0.45]
Duarte, 2019 113 544 0.21 [0.18; 0.24]
Fonseca, 2011 26 170 0.15 [0.11; 0.22]
Ferreira, 2016 127 496 0.26 [0.22; 0.30]
Fulgencio, 2016 205 1344 0.15 [0.13; 0.17]
Gomes, 2018 205 761 0.27 [0.24; 0.30]
Junqueira , 2013 256 937 0.27 [0.25; 0.30]
Leal, 2021 67 739 0.09 [0.07; 0.11]
Lima, 2022 38 105 0.36 [0.28; 0.46]
Manfredini, 2017 408 1556 0.26 [0.24; 0.28]
Massignan, 2019 274 935 0.29 [0.26; 0.32]
Melo , 2014 33 107 0.31 [0.23; 0.40]
Miamoto , 2011 15 60 0.25 [0.16; 0.37]
Souza, 2020 90 403 0.22 [0.19; 0.27]
Ramos, 2021 555 862 0.64 [0.61; 0.68]
Restrepo, 2016 14 37 0.38 [0.24; 0.54]
Ribeiro, 2018 69 207 0.33 [0.27; 0.40]
Sousa, 2018 132 594 0.22 [0.19; 0.26]
Serra-Negra, 2009 230 652 0.35 [0.32; 0.39]
Silva, 2016 16 132 0.12 [0.08; 0.19]
Soares, 2018 74 429 0.17 [0.14; 0.21]
Traebert, 2020 41 389 0.11 [0.08; 0.14]
Vieira-Andrade, 2014 103 749 0.14 [0.11; 0.16]
Prado, 2020 125 403 0.31 [0.27; 0.36]
Prado , 2019 318 1325 0.24 [0.22; 0.26]
Antunes, 2016 21 61 0.34 [0.24; 0.47]
Random effects model 17972 0.24 [0.20; 0.28]
Heterogeneity: I^2 = 97%, τ^2 = 0.4765, p < 0.01
continent = South America, age = adults
Aguiar, 2018 17 152 0.11 [0.07; 0.17]
Coutinho, 2020 42 226 0.19 [0.14; 0.24]
Pontes, 2019 104 1280 0.08 [0.07; 0.10]
Dantas-Neta, 2014 40 306 0.13 [0.10; 0.17]
Holanda, 2022 72 1936 0.04 [0.03; 0.05]
Holanda, 2020 17 240 0.07 [0.04; 0.11]
Siqueira, 2013 94 890 0.11 [0.09; 0.13]
Costa, 2023 507 2059 0.25 [0.23; 0.27]
Emmanuelli, 2023 197 345 0.57 [0.52; 0.62]
Flores, 2023 56 122 0.46 [0.37; 0.55]
Goettems, 2017 139 536 0.26 [0.22; 0.30]
Goulart, 2021 149 366 0.41 [0.36; 0.46]
Maluly, 2020 127 1042 0.12 [0.10; 0.14]
Peixoto, 2021 372 641 0.58 [0.54; 0.62]
Prado, 2018 39 231 0.17 [0.13; 0.22]
Juliatte, 2022 171 384 0.45 [0.40; 0.50]
Silva, 2016 31 134 0.23 [0.17; 0.31]
Soares, 2017 80 253 0.32 [0.26; 0.38]
Vieira, 2020 114 149 0.77 [0.69; 0.83]
Prado , 2019 204 1325 0.15 [0.14; 0.17]
Random effects model 12617 0.23 [0.16; 0.32]
Heterogeneity: I^2 = 99%, τ^2 = 1.1819, p < 0.01
continent = Asia, age = minors
Bharti, 2005 12 103 0.12 [0.07; 0.19]
Demir, 2004 122 965 0.13 [0.11; 0.15]
Emodi-Perlman, 2016 92 1000 0.09 [0.08; 0.11]
Gao, 2023 242 1336 0.18 [0.16; 0.20]
Ghalebandi, 2011 247 4309 0.06 [0.05; 0.06]
Itani, 2013 10333 99416 0.10 [0.10; 0.11]
Kilincaslan, 2014 178 3485 0.05 [0.04; 0.06]
Kim, 2017 190 936 0.20 [0.18; 0.23]
Lam, 2011 382 6471 0.06 [0.05; 0.07]
Liu, 2006 388 5979 0.06 [0.06; 0.07]
Nazzal, 2023 11 194 0.06 [0.03; 0.10]
Perlman, 2016 63 685 0.09 [0.07; 0.12]
Rana, 2017 141 409 0.34 [0.30; 0.39]
Selms, 2019 455 1431 0.32 [0.29; 0.34]
Suwa, 2009 355 1956 0.18 [0.17; 0.20]
Tachibana, 2016 1263 6023 0.21 [0.20; 0.22]
Us, 2021 160 500 0.32 [0.28; 0.36]
Winocur, 2019 327 2347 0.14 [0.13; 0.15]
Nagamatsu-Sakaguchi , 2008 80 127 0.63 [0.54; 0.71]
Random effects model 137672 0.14 [0.10; 0.20]
Heterogeneity: I^2 = 99%, τ^2 = 0.7775, p = 0
continent = Asia, age = adults
Almutairi, 2021 446 1403 0.32 [0.29; 0.34]
Başpınar, 2023 103 249 0.41 [0.35; 0.48]
Cai, 2013 423 2591 0.16 [0.15; 0.18]
Eli, 2022 87 304 0.29 [0.24; 0.34]
Emodi-Perlman, 2020 94 700 0.13 [0.11; 0.16]
Hermesh, 2014 3 33 0.09 [0.02; 0.24]
Karagoz, 2021 55 217 0.25 [0.20; 0.32]
Kato , 2012 152 1930 0.08 [0.07; 0.09]
Khayat, 2019 43 149 0.29 [0.22; 0.37]
Levartovsky, 2022 97 387 0.25 [0.21; 0.30]
Manfredini , 2012 76 397 0.19 [0.16; 0.23]
Nakata, 2007 1462 2680 0.55 [0.53; 0.56]
Shalev-Antsel, 2023 195 587 0.33 [0.30; 0.37]
Shokry , 2016 168 549 0.31 [0.27; 0.35]
Somay, 2020 24 69 0.35 [0.25; 0.47]
Tay, 2020 114 2417 0.05 [0.04; 0.06]
Phuong, 2020 217 568 0.38 [0.34; 0.42]
Tinastepe, 2021 61 167 0.37 [0.30; 0.44]
Toyama, 2020 112 1781 0.06 [0.05; 0.08]
Tsuchiya, 2022 20344 90148 0.23 [0.22; 0.23]
Uca, 2015 46 301 0.15 [0.12; 0.20]
Uma, 2021 202 464 0.44 [0.39; 0.48]
Winocur-Arias, 2023 72 311 0.23 [0.19; 0.28]
Yeler, 2016 147 519 0.28 [0.25; 0.32]
Yıldırım, 2021 51 212 0.24 [0.19; 0.30]
Arsan, 2022 333 1688 0.20 [0.18; 0.22]
Yoshinaka , 2010 102 664 0.15 [0.13; 0.18]
Random effects model 111485 0.23 [0.18; 0.28]
Heterogeneity: I^2 = 99%, τ^2 = 0.5409, p = 0
Random effects model 351351 0.21 [0.19; 0.24]
Prediction interval [0.05; 0.59]
Heterogeneity: I^2 = 99%, τ^2 = 0.7160, p = 0
Test for subgroup differences: χ^2_7 = 10.04, df = 7 (p = 0.19)
0.2 0.4 0.6 0.8

Figure S9. Global prevalence of females sleep bruxism by continent and age.

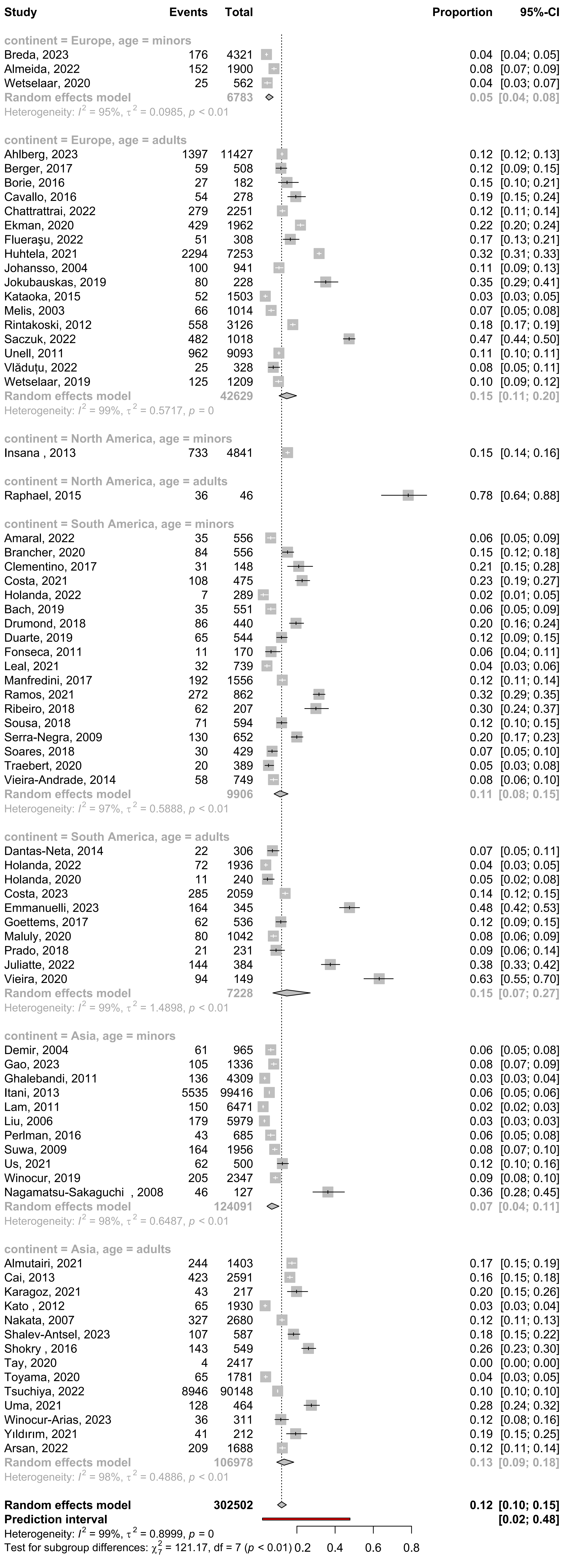

Study Events Total Proportion 95%-CI
continent = Europe, age = minors
Breda, 2023 176 4321 0.04 [0.04; 0.05]
Almeida, 2022 152 1900 0.08 [0.07; 0.09]
Wetselaar, 2020 25 562 0.04 [0.03; 0.07]
Random effects model 6783 0.05 [0.04; 0.08]
Heterogeneity: I^2 = 95%, τ^2 = 0.0985, p < 0.01
continent = Europe, age = adults
Ahlberg, 2023 1397 11427 0.12 [0.12; 0.13]
Berger, 2017 59 508 0.12 [0.09; 0.15]
Borie, 2016 27 182 0.15 [0.10; 0.21]
Cavallo, 2016 54 278 0.19 [0.15; 0.24]
Chattrattrai, 2022 279 2251 0.12 [0.11; 0.14]
Ekman, 2020 429 1962 0.22 [0.20; 0.24]
Flueraşu, 2022 51 308 0.17 [0.13; 0.21]
Huhtela, 2021 2294 7253 0.32 [0.31; 0.33]
Johansso, 2004 100 941 0.11 [0.09; 0.13]
Jokubauskas, 2019 80 228 0.35 [0.29; 0.41]
Kataoka, 2015 52 1503 0.03 [0.03; 0.05]
Melis, 2003 66 1014 0.07 [0.05; 0.08]
Rintakoski, 2012 558 3126 0.18 [0.17; 0.19]
Saczuk, 2022 482 1018 0.47 [0.44; 0.50]
Unell, 2011 962 9093 0.11 [0.10; 0.11]
Vlăduțu, 2022 25 328 0.08 [0.05; 0.11]
Wetselaar, 2019 125 1209 0.10 [0.09; 0.12]
Random effects model 42629 0.15 [0.11; 0.20]
Heterogeneity: I^2 = 99%, τ^2 = 0.5717, p = 0
continent = North America, age = minors
Insana , 2013 733 4841 0.15 [0.14; 0.16]
continent = North America, age = adults
Raphael, 2015 36 46 0.78 [0.64; 0.88]
continent = South America, age = minors
Amaral, 2022 35 556 0.06 [0.05; 0.09]
Brancher, 2020 84 556 0.15 [0.12; 0.18]
Clementino, 2017 31 148 0.21 [0.15; 0.28]
Costa, 2021 108 475 0.23 [0.19; 0.27]
Holanda, 2022 7 289 0.02 [0.01; 0.05]
Bach, 2019 35 551 0.06 [0.05; 0.09]
Drumond, 2018 86 440 0.20 [0.16; 0.24]
Duarte, 2019 65 544 0.12 [0.09; 0.15]
Fonseca, 2011 11 170 0.06 [0.04; 0.11]
Leal, 2021 32 739 0.04 [0.03; 0.06]
Manfredini, 2017 192 1556 0.12 [0.11; 0.14]
Ramos, 2021 272 862 0.32 [0.29; 0.35]
Ribeiro, 2018 62 207 0.30 [0.24; 0.37]
Sousa, 2018 71 594 0.12 [0.10; 0.15]
Serra-Negra, 2009 130 652 0.20 [0.17; 0.23]
Soares, 2018 30 429 0.07 [0.05; 0.10]
Traebert, 2020 20 389 0.05 [0.03; 0.08]
Vieira-Andrade, 2014 58 749 0.08 [0.06; 0.10]
Random effects model 9906 0.11 [0.08; 0.15]
Heterogeneity: I^2 = 97%, τ^2 = 0.5888, p < 0.01
continent = South America, age = adults
Dantas-Neta, 2014 22 306 0.07 [0.05; 0.11]
Holanda, 2022 72 1936 0.04 [0.03; 0.05]
Holanda, 2020 11 240 0.05 [0.02; 0.08]
Costa, 2023 285 2059 0.14 [0.12; 0.15]
Emmanuelli, 2023 164 345 0.48 [0.42; 0.53]
Goettems, 2017 62 536 0.12 [0.09; 0.15]
Maluly, 2020 80 1042 0.08 [0.06; 0.09]
Prado, 2018 21 231 0.09 [0.06; 0.14]
Juliatte, 2022 144 384 0.38 [0.33; 0.42]
Vieira, 2020 94 149 0.63 [0.55; 0.70]
Random effects model 7228 0.15 [0.07; 0.27]
Heterogeneity: I^2 = 99%, τ^2 = 1.4898, p < 0.01
continent = Asia, age = minors
Demir, 2004 61 965 0.06 [0.05; 0.08]
Gao, 2023 105 1336 0.08 [0.07; 0.09]
Ghalebandi, 2011 136 4309 0.03 [0.03; 0.04]
Itani, 2013 5535 99416 0.06 [0.05; 0.06]
Lam, 2011 150 6471 0.02 [0.02; 0.03]
Liu, 2006 179 5979 0.03 [0.03; 0.03]
Perlman, 2016 43 685 0.06 [0.05; 0.08]
Suwa, 2009 164 1956 0.08 [0.07; 0.10]
Us, 2021 62 500 0.12 [0.10; 0.16]
Winocur, 2019 205 2347 0.09 [0.08; 0.10]
Nagamatsu-Sakaguchi , 2008 46 127 0.36 [0.28; 0.45]
Random effects model 124091 0.07 [0.04; 0.11]
Heterogeneity: I^2 = 98%, τ^2 = 0.6487, p < 0.01
continent = Asia, age = adults
Almutairi, 2021 244 1403 0.17 [0.15; 0.19]
Cai, 2013 423 2591 0.16 [0.15; 0.18]
Karagoz, 2021 43 217 0.20 [0.15; 0.26]
Kato , 2012 65 1930 0.03 [0.03; 0.04]
Nakata, 2007 327 2680 0.12 [0.11; 0.13]
Shalev-Antsel, 2023 107 587 0.18 [0.15; 0.22]
Shokry , 2016 143 549 0.26 [0.23; 0.30]
Tay, 2020 4 2417 0.00 [0.00; 0.00]
Toyama, 2020 65 1781 0.04 [0.03; 0.05]
Tsuchiya, 2022 8946 90148 0.10 [0.10; 0.10]
Uma, 2021 128 464 0.28 [0.24; 0.32]
Winocur-Arias, 2023 36 311 0.12 [0.08; 0.16]
Yıldırım, 2021 41 212 0.19 [0.15; 0.25]
Arsan, 2022 209 1688 0.12 [0.11; 0.14]
Random effects model 106978 0.13 [0.09; 0.18]
Heterogeneity: I^2 = 98%, τ^2 = 0.4886, p < 0.01
Random effects model 302502 0.12 [0.10; 0.15]
Prediction interval [0.02; 0.48]
Heterogeneity: I^2 = 99%, τ^2 = 0.8999, p = 0
Test for subgroup differences: χ^2_7 = 121.17, df = 7 (p < 0.01)
0.2 0.4 0.6 0.8

Figure S10. Global prevalence of males sleep bruxism by continent and age.

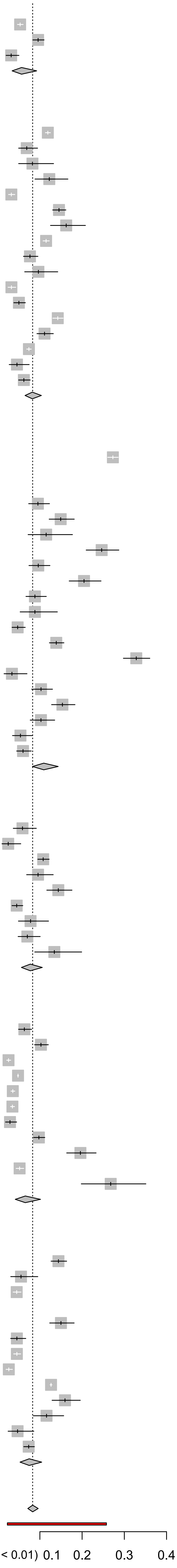

Study	Events	Total	Proportion	95%-CI
**continent = Europe, age = minors**				
Breda, 2023	230	4321	0.05	[0.05; 0.06]
Almeida, 2022	182	1900	0.10	[0.08; 0.11]
Wetselaar, 2020	18	562	0.03	[0.02; 0.05]
**Random effects model**		**6783**	**0.06**	**[0.03; 0.09]**
Heterogeneity: $I^2 = 96\%$, $\tau^2 = 0.1962$, $p < 0.01$				
**continent = Europe, age = adults**				
Ahlberg, 2023	1358	11427	0.12	[0.11; 0.12]
Berger, 2017	35	508	0.07	[0.05; 0.09]
Borie, 2016	15	182	0.08	[0.05; 0.13]
Cavallo, 2016	34	278	0.12	[0.09; 0.17]
Chattrattrai, 2022	73	2251	0.03	[0.03; 0.04]
Ekman, 2020	285	1962	0.15	[0.13; 0.16]
Flueraşu, 2022	50	308	0.16	[0.13; 0.21]
Huhtela, 2021	832	7253	0.11	[0.11; 0.12]
Johansso, 2004	72	941	0.08	[0.06; 0.10]
Jokubauskas, 2019	22	228	0.10	[0.06; 0.14]
Kataoka, 2015	49	1503	0.03	[0.02; 0.04]
Melis, 2003	51	1014	0.05	[0.04; 0.07]
Rintakoski, 2012	445	3126	0.14	[0.13; 0.16]
Saczuk, 2022	113	1018	0.11	[0.09; 0.13]
Unell, 2011	673	9093	0.07	[0.07; 0.08]
Vlăduțu, 2022	15	328	0.05	[0.03; 0.07]
Wetselaar, 2019	75	1209	0.06	[0.05; 0.08]
**Random effects model**		**42629**	**0.08**	**[0.06; 0.10]**
Heterogeneity: $I^2 = 97\%$, $\tau^2 = 0.2666$, $p < 0.01$				
**continent = North America,age = minors**				
Insana , 2013	1321	4841	0.27	[0.26; 0.29]
**continent = South America, age = minors**				
Amaral, 2022	53	556	0.10	[0.07; 0.12]
Brancher, 2020	83	556	0.15	[0.12; 0.18]
Clementino, 2017	17	148	0.11	[0.07; 0.18]
Costa, 2021	117	475	0.25	[0.21; 0.29]
Bach, 2019	53	551	0.10	[0.07; 0.12]
Drumond, 2018	90	440	0.20	[0.17; 0.24]
Duarte, 2019	48	544	0.09	[0.07; 0.12]
Fonseca, 2011	15	170	0.09	[0.05; 0.14]
Leal, 2021	35	739	0.05	[0.03; 0.07]
Manfredini, 2017	216	1556	0.14	[0.12; 0.16]
Ramos, 2021	283	862	0.33	[0.30; 0.36]
Ribeiro, 2018	7	207	0.03	[0.02; 0.07]
Sousa, 2018	61	594	0.10	[0.08; 0.13]
Serra-Negra, 2009	100	652	0.15	[0.13; 0.18]
Soares, 2018	44	429	0.10	[0.08; 0.14]
Traebert, 2020	21	389	0.05	[0.04; 0.08]
Vieira-Andrade, 2014	45	749	0.06	[0.05; 0.08]
**Random effects model**		**9617**	**0.11**	**[0.08; 0.14]**
Heterogeneity: $I^2 = 96\%$, $\tau^2 = 0.3991$, $p < 0.01$				
**continent = South America, age = adults**				
Dantas-Neta, 2014	18	306	0.06	[0.04; 0.09]
Holanda, 2020	6	240	0.02	[0.01; 0.05]
Costa, 2023	222	2059	0.11	[0.10; 0.12]
Emmanuelli, 2023	33	345	0.10	[0.07; 0.13]
Goettems, 2017	77	536	0.14	[0.12; 0.18]
Maluly, 2020	47	1042	0.05	[0.03; 0.06]
Prado, 2018	18	231	0.08	[0.05; 0.12]
Juliatte, 2022	27	384	0.07	[0.05; 0.10]
Vieira, 2020	20	149	0.13	[0.09; 0.20]
**Random effects model**		**5292**	**0.08**	**[0.06; 0.11]**
Heterogeneity: $I^2 = 88\%$, $\tau^2 = 0.2293$, $p < 0.01$				
**continent = Asia, age = minors**				
Demir, 2004	61	965	0.06	[0.05; 0.08]
Gao, 2023	137	1336	0.10	[0.09; 0.12]
Ghalebandi, 2011	111	4309	0.03	[0.02; 0.03]
Itani, 2013	4798	99416	0.05	[0.05; 0.05]
Lam, 2011	232	6471	0.04	[0.03; 0.04]
Liu, 2006	209	5979	0.03	[0.03; 0.04]
Perlman, 2016	20	685	0.03	[0.02; 0.04]
Suwa, 2009	191	1956	0.10	[0.09; 0.11]
Us, 2021	98	500	0.20	[0.16; 0.23]
Winocur, 2019	122	2347	0.05	[0.04; 0.06]
Nagamatsu-Sakaguchi , 2008	34	127	0.27	[0.20; 0.35]
**Random effects model**		**124091**	**0.07**	**[0.04; 0.10]**
Heterogeneity: $I^2 = 98\%$, $\tau^2 = 0.6371$, $p < 0.01$				
**continent = Asia, age = adults**				
Almutairi, 2021	202	1403	0.14	[0.13; 0.16]
Karagoz, 2021	12	217	0.06	[0.03; 0.10]
Kato , 2012	87	1930	0.05	[0.04; 0.06]
Nakata, 2007	1135	2680	0.42	[0.40; 0.44]
Shalev-Antsel, 2023	88	587	0.15	[0.12; 0.18]
Shokry , 2016	25	549	0.05	[0.03; 0.07]
Tay, 2020	110	2417	0.05	[0.04; 0.05]
Toyama, 2020	47	1781	0.03	[0.02; 0.03]
Tsuchiya, 2022	11398	90148	0.13	[0.12; 0.13]
Uma, 2021	74	464	0.16	[0.13; 0.20]
Winocur-Arias, 2023	36	311	0.12	[0.08; 0.16]
Yıldırım, 2021	10	212	0.05	[0.02; 0.09]
Arsan, 2022	124	1688	0.07	[0.06; 0.09]
**Random effects model**		**104387**	**0.07**	**[0.05; 0.10]**
Heterogeneity: $I^2 = 98\%$, $\tau^2 = 0.3929$, $p < 0.01$				
**Random effects model**		**297640**	**0.08**	**[0.07; 0.10]**
**Prediction interval**				**[0.02; 0.26]**

Heterogeneity: $I^2 = 99\%$, $\tau^2 = 0.4479$, $p = 0$

Test for subgroup differences: $\chi^2_6 = 339.82$, df = 6 ($p < 0.01$)

Figure S11. Global prevalence of females sleep bruxism by age.

Study	Events	Total	Proportion	95%-CI
**age = minors**				
Amaral, 2022	35	556	0.06	[0.05; 0.09]
Brancher, 2020	84	556	0.15	[0.12; 0.18]
Breda, 2023	176	4321	0.04	[0.04; 0.05]
Clementino, 2017	31	148	0.21	[0.15; 0.28]
Costa, 2021	108	475	0.23	[0.19; 0.27]
Almeida, 2022	152	1900	0.08	[0.07; 0.09]
Holanda, 2022	7	289	0.02	[0.01; 0.05]
Bach, 2019	35	551	0.06	[0.05; 0.09]
Demir, 2004	61	965	0.06	[0.05; 0.08]
Drumond, 2018	86	440	0.20	[0.16; 0.24]
Duarte, 2019	65	544	0.12	[0.09; 0.15]
Fonseca, 2011	11	170	0.06	[0.04; 0.11]
Gao, 2023	105	1336	0.08	[0.07; 0.09]
Ghalebandi, 2011	136	4309	0.03	[0.03; 0.04]
Insana , 2013	733	4841	0.15	[0.14; 0.16]
Itani, 2013	5535	99416	0.06	[0.05; 0.06]
Lam, 2011	150	6471	0.02	[0.02; 0.03]
Leal, 2021	32	739	0.04	[0.03; 0.06]
Liu, 2006	179	5979	0.03	[0.03; 0.03]
Manfredini, 2017	192	1556	0.12	[0.11; 0.14]
Perlman, 2016	43	685	0.06	[0.05; 0.08]
Ramos, 2021	272	862	0.32	[0.29; 0.35]
Ribeiro, 2018	62	207	0.30	[0.24; 0.37]
Rubin, 2018	12	153	0.08	[0.04; 0.13]
Sousa, 2018	71	594	0.12	[0.10; 0.15]
Serra-Negra, 2009	130	652	0.20	[0.17; 0.23]
Shahbour, 2022	82	1000	0.08	[0.07; 0.10]
Soares, 2018	30	429	0.07	[0.05; 0.10]
Suwa, 2009	164	1956	0.08	[0.07; 0.10]
Traebert, 2020	20	389	0.05	[0.03; 0.08]
Us, 2021	62	500	0.12	[0.10; 0.16]
Vieira-Andrade, 2014	58	749	0.08	[0.06; 0.10]
Wetselaar, 2020	25	562	0.04	[0.03; 0.07]
Winocur, 2019	205	2347	0.09	[0.08; 0.10]
Nagamatsu-Sakaguchi , 2008	46	127	0.36	[0.28; 0.45]
**Random effects model**		**146774**	**0.09**	**[0.07; 0.11]**
Heterogeneity: I^2 = 99%, τ^2 = 0.6201, p = 0				
**age = adults**				
Ahlberg, 2023	1397	11427	0.12	[0.12; 0.13]
Almutairi, 2021	244	1403	0.17	[0.15; 0.19]
Azodo, 2016	12	578	0.02	[0.01; 0.04]
Berger, 2017	59	508	0.12	[0.09; 0.15]
Borie, 2016	27	182	0.15	[0.10; 0.21]
Cai, 2013	423	2591	0.16	[0.15; 0.18]
Cavallo, 2016	54	278	0.19	[0.15; 0.24]
Chattrattrai, 2022	279	2251	0.12	[0.11; 0.14]
Dantas-Neta, 2014	22	306	0.07	[0.05; 0.11]
Holanda, 2022	72	1936	0.04	[0.03; 0.05]
Holanda, 2020	11	240	0.05	[0.02; 0.08]
Costa, 2023	285	2059	0.14	[0.12; 0.15]
Ekman, 2020	429	1962	0.22	[0.20; 0.24]
Emmanuelli, 2023	164	345	0.48	[0.42; 0.53]
Fan, 2018	146	912	0.16	[0.14; 0.19]
Flueraşu, 2022	51	308	0.17	[0.13; 0.21]
Goettems, 2017	62	536	0.12	[0.09; 0.15]
Huhtela, 2021	2294	7253	0.32	[0.31; 0.33]
Johansso, 2004	100	941	0.11	[0.09; 0.13]
Jokubauskas, 2019	80	228	0.35	[0.29; 0.41]
Karagoz, 2021	43	217	0.20	[0.15; 0.26]
Kataoka, 2015	52	1503	0.03	[0.03; 0.05]
Kato , 2012	65	1930	0.03	[0.03; 0.04]
Maluly, 2020	80	1042	0.08	[0.06; 0.09]
Melis, 2003	66	1014	0.07	[0.05; 0.08]
Nakata, 2007	327	2680	0.12	[0.11; 0.13]
Prado, 2018	21	231	0.09	[0.06; 0.14]
Raphael, 2015	36	46	0.78	[0.64; 0.88]
Rintakoski, 2012	558	3126	0.18	[0.17; 0.19]
Juliatte, 2022	144	384	0.38	[0.33; 0.42]
Saczuk, 2022	482	1018	0.47	[0.44; 0.50]
Shalev-Antsel, 2023	107	587	0.18	[0.15; 0.22]
Shokry , 2016	143	549	0.26	[0.23; 0.30]
Tay, 2020	4	2417	0.00	[0.00; 0.00]
Toyama, 2020	65	1781	0.04	[0.03; 0.05]
Tsuchiya, 2022	8946	90148	0.10	[0.10; 0.10]
Uma, 2021	128	464	0.28	[0.24; 0.32]
Unell, 2011	962	9093	0.11	[0.10; 0.11]
Vieira, 2020	94	149	0.63	[0.55; 0.70]
Vlăduţu, 2022	25	328	0.08	[0.05; 0.11]
Wetselaar, 2019	125	1209	0.10	[0.09; 0.12]
Winocur-Arias, 2023	36	311	0.12	[0.08; 0.16]
Yıldırım, 2021	41	212	0.19	[0.15; 0.25]
Arsan, 2022	209	1688	0.12	[0.11; 0.14]
**Random effects model**		**158371**	**0.14**	**[0.11; 0.19]**
Heterogeneity: I^2 = 99%, τ^2 = 0.9955, p = 0				
**Random effects model**		**305145**	**0.12**	**[0.10; 0.14]**
**Prediction interval**				**[0.02; 0.47]**
Heterogeneity: I^2 = 99%, τ^2 = 0.9006, p = 0				
Test for subgroup differences: χ^2_1 = 7.23, df = 1 (p < 0.01)				

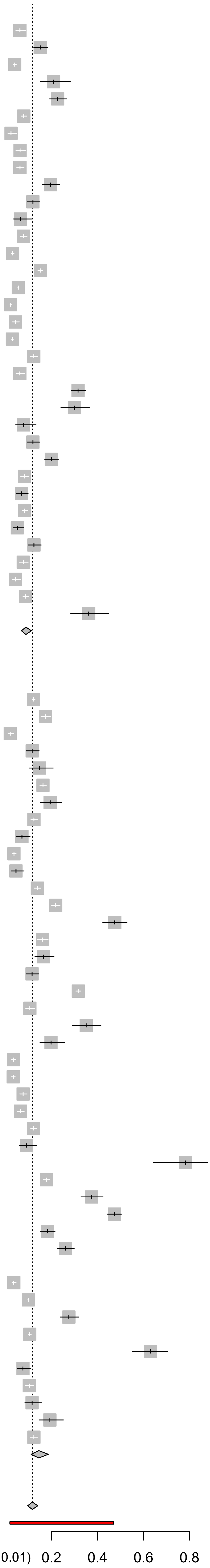

Figure S12. Global prevalence of males sleep bruxism by age.

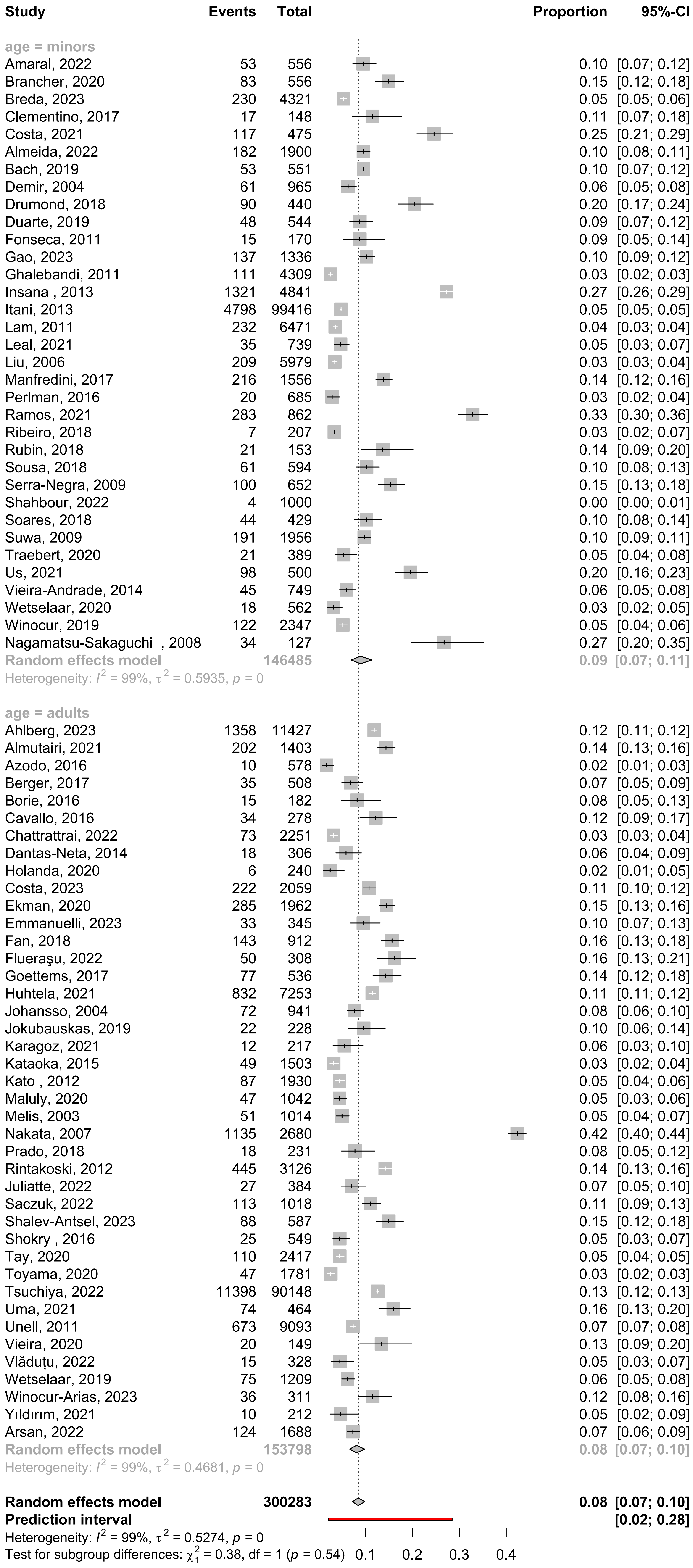
Study Events Total Proportion 95%-CI
age = minors
Amaral, 2022 53 556 0.10 [0.07; 0.12]
Brancher, 2020 83 556 0.15 [0.12; 0.18]
Breda, 2023 230 4321 0.05 [0.05; 0.06]
Clementino, 2017 17 148 0.11 [0.07; 0.18]
Costa, 2021 117 475 0.25 [0.21; 0.29]
Almeida, 2022 182 1900 0.10 [0.08; 0.11]
Bach, 2019 53 551 0.10 [0.07; 0.12]
Demir, 2004 61 965 0.06 [0.05; 0.08]
Drumond, 2018 90 440 0.20 [0.17; 0.24]
Duarte, 2019 48 544 0.09 [0.07; 0.12]
Fonseca, 2011 15 170 0.09 [0.05; 0.14]
Gao, 2023 137 1336 0.10 [0.09; 0.12]
Ghalebandi, 2011 111 4309 0.03 [0.02; 0.03]
Insana , 2013 1321 4841 0.27 [0.26; 0.29]
Itani, 2013 4798 99416 0.05 [0.05; 0.05]
Lam, 2011 232 6471 0.04 [0.03; 0.04]
Leal, 2021 35 739 0.05 [0.03; 0.07]
Liu, 2006 209 5979 0.03 [0.03; 0.04]
Manfredini, 2017 216 1556 0.14 [0.12; 0.16]
Perlman, 2016 20 685 0.03 [0.02; 0.04]
Ramos, 2021 283 862 0.33 [0.30; 0.36]
Ribeiro, 2018 7 207 0.03 [0.02; 0.07]
Rubin, 2018 21 153 0.14 [0.09; 0.20]
Sousa, 2018 61 594 0.10 [0.08; 0.13]
Serra-Negra, 2009 100 652 0.15 [0.13; 0.18]
Shahbour, 2022 4 1000 0.00 [0.00; 0.01]
Soares, 2018 44 429 0.10 [0.08; 0.14]
Suwa, 2009 191 1956 0.10 [0.09; 0.11]
Traebert, 2020 21 389 0.05 [0.04; 0.08]
Us, 2021 98 500 0.20 [0.16; 0.23]
Vieira-Andrade, 2014 45 749 0.06 [0.05; 0.08]
Wetselaar, 2020 18 562 0.03 [0.02; 0.05]
Winocur, 2019 122 2347 0.05 [0.04; 0.06]
Nagamatsu-Sakaguchi , 2008 34 127 0.27 [0.20; 0.35]
Random effects model 146485 0.09 [0.07; 0.11]
Heterogeneity: I^2 = 99%, τ^2 = 0.5935, p = 0
age = adults
Ahlberg, 2023 1358 11427 0.12 [0.11; 0.12]
Almutairi, 2021 202 1403 0.14 [0.13; 0.16]
Azodo, 2016 10 578 0.02 [0.01; 0.03]
Berger, 2017 35 508 0.07 [0.05; 0.09]
Borie, 2016 15 182 0.08 [0.05; 0.13]
Cavallo, 2016 34 278 0.12 [0.09; 0.17]
Chattrattrai, 2022 73 2251 0.03 [0.03; 0.04]
Dantas-Neta, 2014 18 306 0.06 [0.04; 0.09]
Holanda, 2020 6 240 0.02 [0.01; 0.05]
Costa, 2023 222 2059 0.11 [0.10; 0.12]
Ekman, 2020 285 1962 0.15 [0.13; 0.16]
Emmanuelli, 2023 33 345 0.10 [0.07; 0.13]
Fan, 2018 143 912 0.16 [0.13; 0.18]
Flueraşu, 2022 50 308 0.16 [0.13; 0.21]
Goettems, 2017 77 536 0.14 [0.12; 0.18]
Huhtela, 2021 832 7253 0.11 [0.11; 0.12]
Johansso, 2004 72 941 0.08 [0.06; 0.10]
Jokubauskas, 2019 22 228 0.10 [0.06; 0.14]
Karagoz, 2021 12 217 0.06 [0.03; 0.10]
Kataoka, 2015 49 1503 0.03 [0.02; 0.04]
Kato , 2012 87 1930 0.05 [0.04; 0.06]
Maluly, 2020 47 1042 0.05 [0.03; 0.06]
Melis, 2003 51 1014 0.05 [0.04; 0.07]
Nakata, 2007 1135 2680 0.42 [0.40; 0.44]
Prado, 2018 18 231 0.08 [0.05; 0.12]
Rintakoski, 2012 445 3126 0.14 [0.13; 0.16]
Juliatte, 2022 27 384 0.07 [0.05; 0.10]
Saczuk, 2022 113 1018 0.11 [0.09; 0.13]
Shalev-Antsel, 2023 88 587 0.15 [0.12; 0.18]
Shokry , 2016 25 549 0.05 [0.03; 0.07]
Tay, 2020 110 2417 0.05 [0.04; 0.05]
Toyama, 2020 47 1781 0.03 [0.02; 0.03]
Tsuchiya, 2022 11398 90148 0.13 [0.12; 0.13]
Uma, 2021 74 464 0.16 [0.13; 0.20]
Unell, 2011 673 9093 0.07 [0.07; 0.08]
Vieira, 2020 20 149 0.13 [0.09; 0.20]
Vlăduțu, 2022 15 328 0.05 [0.03; 0.07]
Wetselaar, 2019 75 1209 0.06 [0.05; 0.08]
Winocur-Arias, 2023 36 311 0.12 [0.08; 0.16]
Yıldırım, 2021 10 212 0.05 [0.02; 0.09]
Arsan, 2022 124 1688 0.07 [0.06; 0.09]
Random effects model 153798 0.08 [0.07; 0.10]
Heterogeneity: I^2 = 99%, τ^2 = 0.4681, p = 0
Random effects model 300283 0.08 [0.07; 0.10]
Prediction interval [0.02; 0.28]
Heterogeneity: I^2 = 99%, τ^2 = 0.5274, p = 0
Test for subgroup differences: χ^2_1 = 0.38, df = 1 (p = 0.54)
0.1 0.2 0.3 0.4

Figure S13. Global prevalence of awake bruxism.

Study	Events	Total	Proportion	95%-CI
Aguiar, 2018	56	152	0.37	[0.30; 0.45]
Almutairi, 2021	632	1403	0.45	[0.42; 0.48]
Alonso, 2021	190	434	0.44	[0.39; 0.48]
Azodo, 2016	52	578	0.09	[0.07; 0.12]
Başpınar, 2023	54	249	0.22	[0.17; 0.27]
Berger, 2017	57	508	0.11	[0.09; 0.14]
Câmara-Souza, 2023	26	69	0.38	[0.27; 0.49]
Cavallo, 2016	106	278	0.38	[0.33; 0.44]
Silva, 2023	110	739	0.15	[0.12; 0.18]
Dantas-Neta, 2014	85	306	0.28	[0.23; 0.33]
Ekman, 2020	193	1962	0.10	[0.09; 0.11]
Eli, 2022	31	304	0.10	[0.07; 0.14]
Emodi-Perlman, 2020	111	700	0.16	[0.13; 0.19]
Emodi-Perlman, 2020	372	1092	0.34	[0.31; 0.37]
Emodi-Perlman, 2016	192	1000	0.19	[0.17; 0.22]
Fan, 2018	441	912	0.48	[0.45; 0.52]
Flueraşu, 2022	47	308	0.15	[0.12; 0.20]
Goulart, 2021	12	366	0.03	[0.02; 0.06]
Hermesh, 2014	1	33	0.03	[0.00; 0.17]
Huhtela, 2021	2038	7253	0.28	[0.27; 0.29]
Jokubauskas, 2019	132	228	0.58	[0.51; 0.64]
Junqueira , 2013	19	937	0.02	[0.01; 0.03]
Khayat, 2019	22	149	0.15	[0.10; 0.21]
Levartovsky, 2022	90	387	0.23	[0.19; 0.28]
Macfarlane, 2003	193	2137	0.09	[0.08; 0.10]
Manfredini , 2012	43	397	0.11	[0.08; 0.14]
Manfredini , 2012	25	219	0.11	[0.08; 0.16]
Melis, 2003	95	1014	0.09	[0.08; 0.11]
Nekora-Azak, 2009	535	795	0.67	[0.64; 0.70]
Souza, 2020	198	403	0.49	[0.44; 0.54]
Nykinen, 2023	11	46	0.24	[0.14; 0.38]
Osses-Anguita, 2023	85	274	0.31	[0.26; 0.37]
Panek , 2012	24	303	0.08	[0.05; 0.12]
Peixoto, 2021	345	641	0.54	[0.50; 0.58]
Pereira, 2020	22	38	0.58	[0.42; 0.72]
Perlman, 2016	131	685	0.19	[0.16; 0.22]
Prado, 2018	55	231	0.24	[0.19; 0.30]
Rao, 2011	87	147	0.59	[0.51; 0.67]
Rauch , 2023	20	166	0.12	[0.08; 0.18]
Juliatte, 2022	194	384	0.51	[0.46; 0.55]
Rubin, 2018	57	153	0.37	[0.30; 0.45]
Saczuk, 2022	480	1018	0.47	[0.44; 0.50]
Serra-Negra, 2021	21	69	0.30	[0.21; 0.42]
Serra-Negra, 2021	48	136	0.35	[0.28; 0.44]
Shalev-Antsel, 2023	241	587	0.41	[0.37; 0.45]
Sierwald, 2015	100	890	0.11	[0.09; 0.13]
Phuong, 2020	133	568	0.23	[0.20; 0.27]
Tinastepe, 2021	97	167	0.58	[0.50; 0.65]
Uma, 2021	166	464	0.36	[0.32; 0.40]
Selms, 2012	366	4205	0.09	[0.08; 0.10]
Vlăduțu, 2022	108	328	0.33	[0.28; 0.38]
Wetselaar, 2019	60	1209	0.05	[0.04; 0.06]
Wetselaar, 2020	23	562	0.04	[0.03; 0.06]
Prado, 2020	208	403	0.52	[0.47; 0.56]
Winocur, 2019	678	2347	0.29	[0.27; 0.31]
Winocur-Arias, 2023	96	311	0.31	[0.26; 0.36]
Zani, 2019	11	30	0.37	[0.22; 0.55]
Yoshinaka , 2010	42	664	0.06	[0.05; 0.08]
Hilgenberg-Sydney, 2022	24	50	0.48	[0.35; 0.61]
**Random effects model**		**42388**	**0.23**	**[0.19; 0.29]**
**Prediction interval**				**[0.03; 0.72]**

Heterogeneity: $I^2 = 99\%$, $\tau^2 = 1.1113$, $p = 0$

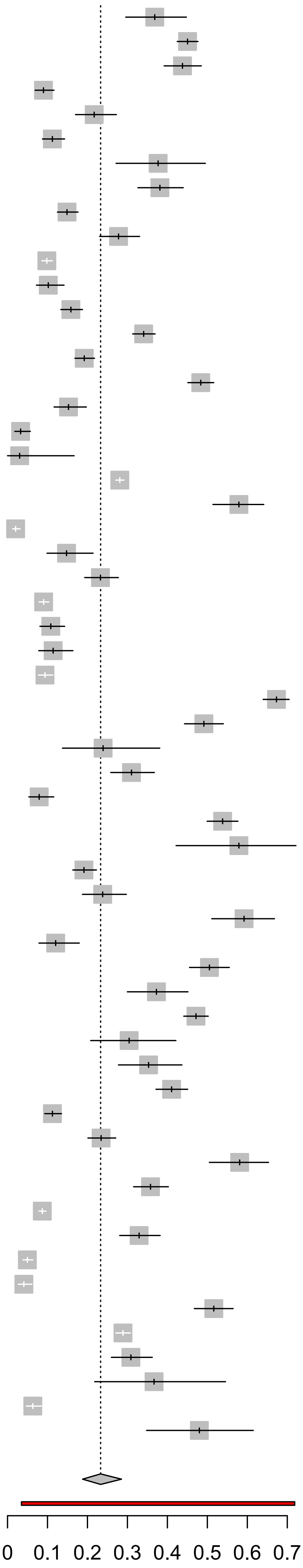

Figure S14. Global prevalence of awake bruxism by age.

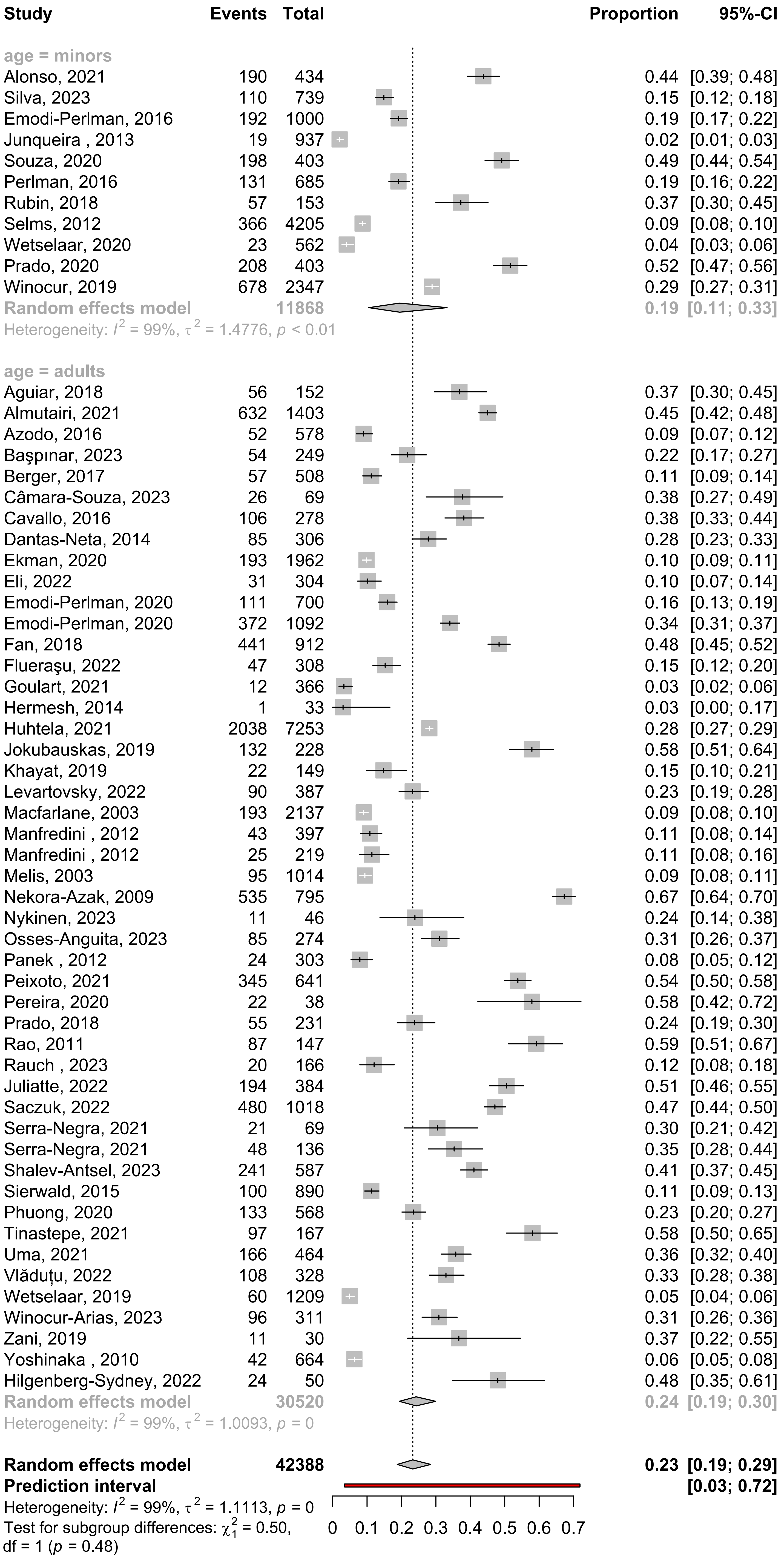
Study Events Total Proportion 95%-CI
age = minors
Alonso, 2021 190 434 0.44 [0.39; 0.48]
Silva, 2023 110 739 0.15 [0.12; 0.18]
Emodi-Perlman, 2016 192 1000 0.19 [0.17; 0.22]
Junqueira , 2013 19 937 0.02 [0.01; 0.03]
Souza, 2020 198 403 0.49 [0.44; 0.54]
Perlman, 2016 131 685 0.19 [0.16; 0.22]
Rubin, 2018 57 153 0.37 [0.30; 0.45]
Selms, 2012 366 4205 0.09 [0.08; 0.10]
Wetselaar, 2020 23 562 0.04 [0.03; 0.06]
Prado, 2020 208 403 0.52 [0.47; 0.56]
Winocur, 2019 678 2347 0.29 [0.27; 0.31]
Random effects model 11868 0.19 [0.11; 0.33]
Heterogeneity: I^2 = 99%, τ^2 = 1.4776, p < 0.01
age = adults
Aguiar, 2018 56 152 0.37 [0.30; 0.45]
Almutairi, 2021 632 1403 0.45 [0.42; 0.48]
Azodo, 2016 52 578 0.09 [0.07; 0.12]
Başpınar, 2023 54 249 0.22 [0.17; 0.27]
Berger, 2017 57 508 0.11 [0.09; 0.14]
Câmara-Souza, 2023 26 69 0.38 [0.27; 0.49]
Cavallo, 2016 106 278 0.38 [0.33; 0.44]
Dantas-Neta, 2014 85 306 0.28 [0.23; 0.33]
Ekman, 2020 193 1962 0.10 [0.09; 0.11]
Eli, 2022 31 304 0.10 [0.07; 0.14]
Emodi-Perlman, 2020 111 700 0.16 [0.13; 0.19]
Emodi-Perlman, 2020 372 1092 0.34 [0.31; 0.37]
Fan, 2018 441 912 0.48 [0.45; 0.52]
Flueraşu, 2022 47 308 0.15 [0.12; 0.20]
Goulart, 2021 12 366 0.03 [0.02; 0.06]
Hermesh, 2014 1 33 0.03 [0.00; 0.17]
Huhtela, 2021 2038 7253 0.28 [0.27; 0.29]
Jokubauskas, 2019 132 228 0.58 [0.51; 0.64]
Khayat, 2019 22 149 0.15 [0.10; 0.21]
Levartovsky, 2022 90 387 0.23 [0.19; 0.28]
Macfarlane, 2003 193 2137 0.09 [0.08; 0.10]
Manfredini , 2012 43 397 0.11 [0.08; 0.14]
Manfredini , 2012 25 219 0.11 [0.08; 0.16]
Melis, 2003 95 1014 0.09 [0.08; 0.11]
Nekora-Azak, 2009 535 795 0.67 [0.64; 0.70]
Nykinen, 2023 11 46 0.24 [0.14; 0.38]
Osses-Anguita, 2023 85 274 0.31 [0.26; 0.37]
Panek , 2012 24 303 0.08 [0.05; 0.12]
Peixoto, 2021 345 641 0.54 [0.50; 0.58]
Pereira, 2020 22 38 0.58 [0.42; 0.72]
Prado, 2018 55 231 0.24 [0.19; 0.30]
Rao, 2011 87 147 0.59 [0.51; 0.67]
Rauch , 2023 20 166 0.12 [0.08; 0.18]
Juliatte, 2022 194 384 0.51 [0.46; 0.55]
Saczuk, 2022 480 1018 0.47 [0.44; 0.50]
Serra-Negra, 2021 21 69 0.30 [0.21; 0.42]
Serra-Negra, 2021 48 136 0.35 [0.28; 0.44]
Shalev-Antsel, 2023 241 587 0.41 [0.37; 0.45]
Sierwald, 2015 100 890 0.11 [0.09; 0.13]
Phuong, 2020 133 568 0.23 [0.20; 0.27]
Tinastepe, 2021 97 167 0.58 [0.50; 0.65]
Uma, 2021 166 464 0.36 [0.32; 0.40]
Vlăduțu, 2022 108 328 0.33 [0.28; 0.38]
Wetselaar, 2019 60 1209 0.05 [0.04; 0.06]
Winocur-Arias, 2023 96 311 0.31 [0.26; 0.36]
Zani, 2019 11 30 0.37 [0.22; 0.55]
Yoshinaka , 2010 42 664 0.06 [0.05; 0.08]
Hilgenberg-Sydney, 2022 24 50 0.48 [0.35; 0.61]
Random effects model 30520 0.24 [0.19; 0.30]
Heterogeneity: I^2 = 99%, τ^2 = 1.0093, p = 0
Random effects model 42388 0.23 [0.19; 0.29]
Prediction interval [0.03; 0.72]
Heterogeneity: I^2 = 99%, τ^2 = 1.1113, p = 0
Test for subgroup differences: χ^2_1 = 0.50, df = 1 (p = 0.48)
0 0.1 0.2 0.3 0.4 0.5 0.6 0.7

Figure S15. Global prevalence of awake bruxism in the female population.

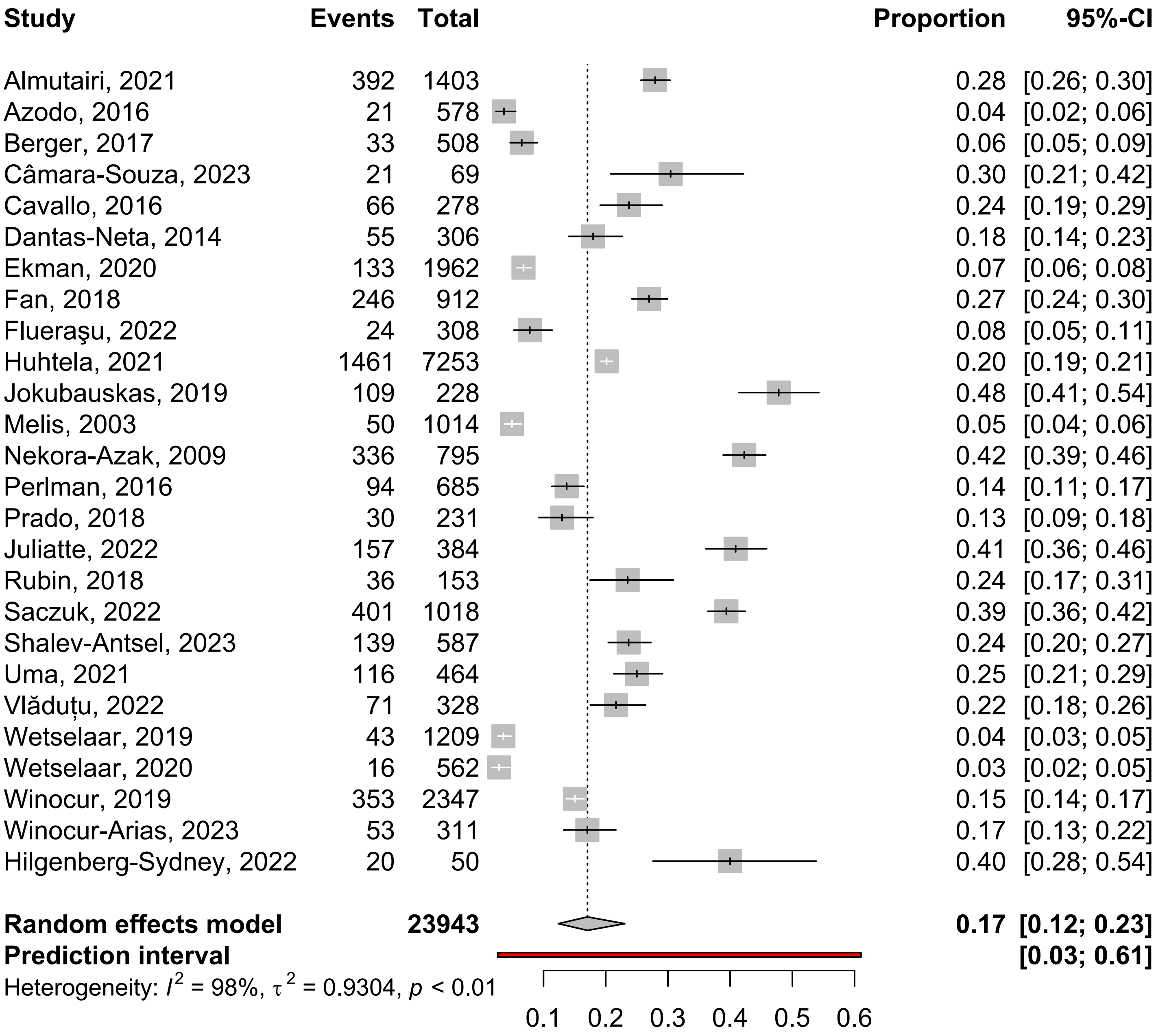
Study
Events
Total
Proportion
95%-CI
Almutairi, 2021 392 1403 0.28 [0.26; 0.30]
Azodo, 2016 21 578 0.04 [0.02; 0.06]
Berger, 2017 33 508 0.06 [0.05; 0.09]
Câmara-Souza, 2023 21 69 0.30 [0.21; 0.42]
Cavallo, 2016 66 278 0.24 [0.19; 0.29]
Dantas-Neta, 2014 55 306 0.18 [0.14; 0.23]
Ekman, 2020 133 1962 0.07 [0.06; 0.08]
Fan, 2018 246 912 0.27 [0.24; 0.30]
Flueraşu, 2022 24 308 0.08 [0.05; 0.11]
Huhtela, 2021 1461 7253 0.20 [0.19; 0.21]
Jokubauskas, 2019 109 228 0.48 [0.41; 0.54]
Melis, 2003 50 1014 0.05 [0.04; 0.06]
Nekora-Azak, 2009 336 795 0.42 [0.39; 0.46]
Perlman, 2016 94 685 0.14 [0.11; 0.17]
Prado, 2018 30 231 0.13 [0.09; 0.18]
Juliatte, 2022 157 384 0.41 [0.36; 0.46]
Rubin, 2018 36 153 0.24 [0.17; 0.31]
Saczuk, 2022 401 1018 0.39 [0.36; 0.42]
Shalev-Antsel, 2023 139 587 0.24 [0.20; 0.27]
Uma, 2021 116 464 0.25 [0.21; 0.29]
Vlăduțu, 2022 71 328 0.22 [0.18; 0.26]
Wetselaar, 2019 43 1209 0.04 [0.03; 0.05]
Wetselaar, 2020 16 562 0.03 [0.02; 0.05]
Winocur, 2019 353 2347 0.15 [0.14; 0.17]
Winocur-Arias, 2023 53 311 0.17 [0.13; 0.22]
Hilgenberg-Sydney, 2022 20 50 0.40 [0.28; 0.54]
Random effects model 23943 0.17 [0.12; 0.23]
Prediction interval [0.03; 0.61]
Heterogeneity: I^2 = 98%, τ^2 = 0.9304, p < 0.01
0.1
0.2
0.3
0.4
0.5
0.6

Figure S16. Global prevalence of awake bruxism in the male population.

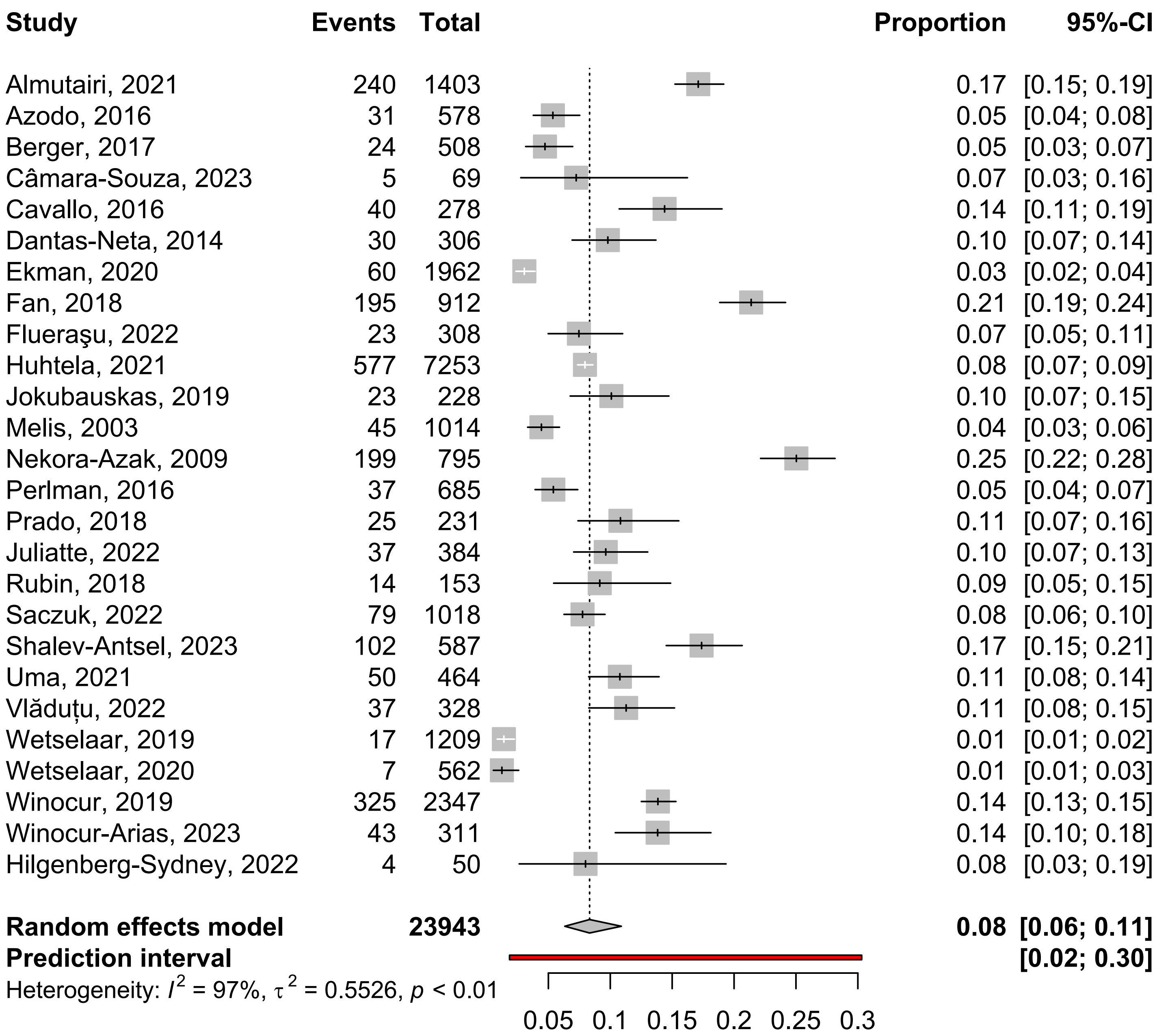
Study
Events
Total
Proportion
95%-CI
Almutairi, 2021 240 1403 0.17 [0.15; 0.19]
Azodo, 2016 31 578 0.05 [0.04; 0.08]
Berger, 2017 24 508 0.05 [0.03; 0.07]
Câmara-Souza, 2023 5 69 0.07 [0.03; 0.16]
Cavallo, 2016 40 278 0.14 [0.11; 0.19]
Dantas-Neta, 2014 30 306 0.10 [0.07; 0.14]
Ekman, 2020 60 1962 0.03 [0.02; 0.04]
Fan, 2018 195 912 0.21 [0.19; 0.24]
Flueraşu, 2022 23 308 0.07 [0.05; 0.11]
Huhtela, 2021 577 7253 0.08 [0.07; 0.09]
Jokubauskas, 2019 23 228 0.10 [0.07; 0.15]
Melis, 2003 45 1014 0.04 [0.03; 0.06]
Nekora-Azak, 2009 199 795 0.25 [0.22; 0.28]
Perlman, 2016 37 685 0.05 [0.04; 0.07]
Prado, 2018 25 231 0.11 [0.07; 0.16]
Juliatte, 2022 37 384 0.10 [0.07; 0.13]
Rubin, 2018 14 153 0.09 [0.05; 0.15]
Saczuk, 2022 79 1018 0.08 [0.06; 0.10]
Shalev-Antsel, 2023 102 587 0.17 [0.15; 0.21]
Uma, 2021 50 464 0.11 [0.08; 0.14]
Vlăduțu, 2022 37 328 0.11 [0.08; 0.15]
Wetselaar, 2019 17 1209 0.01 [0.01; 0.02]
Wetselaar, 2020 7 562 0.01 [0.01; 0.03]
Winocur, 2019 325 2347 0.14 [0.13; 0.15]
Winocur-Arias, 2023 43 311 0.14 [0.10; 0.18]
Hilgenberg-Sydney, 2022 4 50 0.08 [0.03; 0.19]
Random effects model 23943 0.08 [0.06; 0.11]
Prediction interval [0.02; 0.30]
Heterogeneity: I^2 = 97%, τ^2 = 0.5526, p < 0.01
0.05 0.1 0.15 0.2 0.25 0.3

Figure S17. Global prevalence of awake bruxism by continent.

Study	Events	Total	Proportion	95%-CI
**continent = Europe**				
Berger, 2017	57	508	0.11	[0.09; 0.14]
Cavallo, 2016	106	278	0.38	[0.33; 0.44]
Ekman, 2020	193	1962	0.10	[0.09; 0.11]
Emodi-Perlman, 2020	372	1092	0.34	[0.31; 0.37]
Flueraşu, 2022	47	308	0.15	[0.12; 0.20]
Huhtela, 2021	2038	7253	0.28	[0.27; 0.29]
Jokubauskas, 2019	132	228	0.58	[0.51; 0.64]
Macfarlane, 2003	193	2137	0.09	[0.08; 0.10]
Manfredini , 2012	25	219	0.11	[0.08; 0.16]
Melis, 2003	95	1014	0.09	[0.08; 0.11]
Nykinen, 2023	11	46	0.24	[0.14; 0.38]
Osses-Anguita, 2023	85	274	0.31	[0.26; 0.37]
Panek , 2012	24	303	0.08	[0.05; 0.12]
Rauch , 2023	20	166	0.12	[0.08; 0.18]
Saczuk, 2022	480	1018	0.47	[0.44; 0.50]
Serra-Negra, 2021	48	136	0.35	[0.28; 0.44]
Sierwald, 2015	100	890	0.11	[0.09; 0.13]
Selms, 2012	366	4205	0.09	[0.08; 0.10]
Vlăduțu, 2022	108	328	0.33	[0.28; 0.38]
Wetselaar, 2019	60	1209	0.05	[0.04; 0.06]
Wetselaar, 2020	23	562	0.04	[0.03; 0.06]
Zani, 2019	11	30	0.37	[0.22; 0.55]
**Random effects model**		**24166**	**0.18**	**[0.13; 0.25]**
Heterogeneity: $I^2 = 99\%$, $\tau^2 = 0.8781$, $p = 0$				
**continent = South America**				
Aguiar, 2018	56	152	0.37	[0.30; 0.45]
Alonso, 2021	190	434	0.44	[0.39; 0.48]
Câmara-Souza, 2023	26	69	0.38	[0.27; 0.49]
Silva, 2023	110	739	0.15	[0.12; 0.18]
Dantas-Neta, 2014	85	306	0.28	[0.23; 0.33]
Goulart, 2021	12	366	0.03	[0.02; 0.06]
Junqueira , 2013	19	937	0.02	[0.01; 0.03]
Souza, 2020	198	403	0.49	[0.44; 0.54]
Peixoto, 2021	345	641	0.54	[0.50; 0.58]
Pereira, 2020	22	38	0.58	[0.42; 0.72]
Prado, 2018	55	231	0.24	[0.19; 0.30]
Juliatte, 2022	194	384	0.51	[0.46; 0.55]
Serra-Negra, 2021	21	69	0.30	[0.21; 0.42]
Prado, 2020	208	403	0.52	[0.47; 0.56]
Hilgenberg-Sydney, 2022	24	50	0.48	[0.35; 0.61]
**Random effects model**		**5222**	**0.30**	**[0.19; 0.44]**
Heterogeneity: $I^2 = 98\%$, $\tau^2 = 1.4363$, $p < 0.01$				
**continent = Asia**				
Almutairi, 2021	632	1403	0.45	[0.42; 0.48]
Başpınar, 2023	54	249	0.22	[0.17; 0.27]
Eli, 2022	31	304	0.10	[0.07; 0.14]
Emodi-Perlman, 2020	111	700	0.16	[0.13; 0.19]
Emodi-Perlman, 2016	192	1000	0.19	[0.17; 0.22]
Hermesh, 2014	1	33	0.03	[0.00; 0.17]
Khayat, 2019	22	149	0.15	[0.10; 0.21]
Levartovsky, 2022	90	387	0.23	[0.19; 0.28]
Manfredini , 2012	43	397	0.11	[0.08; 0.14]
Nekora-Azak, 2009	535	795	0.67	[0.64; 0.70]
Perlman, 2016	131	685	0.19	[0.16; 0.22]
Rao, 2011	87	147	0.59	[0.51; 0.67]
Shalev-Antsel, 2023	241	587	0.41	[0.37; 0.45]
Phuong, 2020	133	568	0.23	[0.20; 0.27]
Tinastepe, 2021	97	167	0.58	[0.50; 0.65]
Uma, 2021	166	464	0.36	[0.32; 0.40]
Winocur, 2019	678	2347	0.29	[0.27; 0.31]
Winocur-Arias, 2023	96	311	0.31	[0.26; 0.36]
Yoshinaka , 2010	42	664	0.06	[0.05; 0.08]
**Random effects model**		**11357**	**0.25**	**[0.18; 0.34]**
Heterogeneity: $I^2 = 98\%$, $\tau^2 = 0.9091$, $p < 0.01$				
		**40745**	**0.23**	**[0.18; 0.28]**
**Random effects model Prediction interval**				**[0.03; 0.72]**

Heterogeneity: $I^2 = 99\%$, $\tau^2 = 1.1186$, $p = 0$

Test for subgroup differences: $\chi^2_2 = 3.84$, df = 2 ($p = 0.15$)

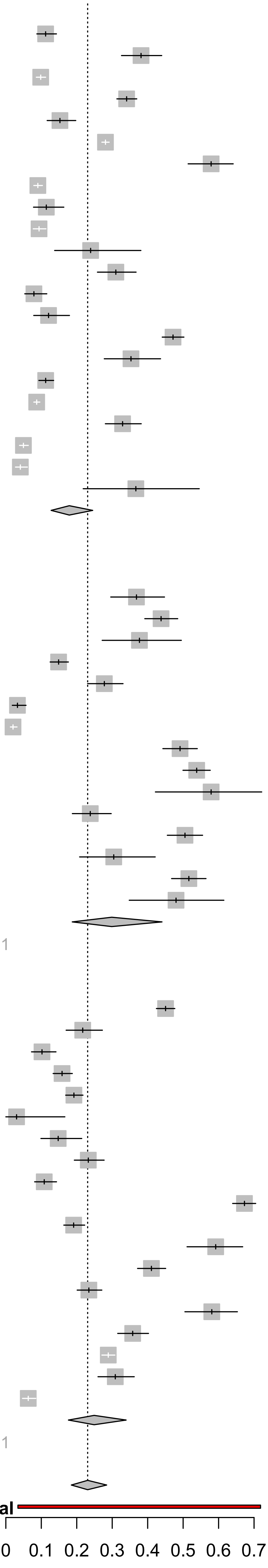

Figure S18. Global prevalence of females awake bruxism by age.

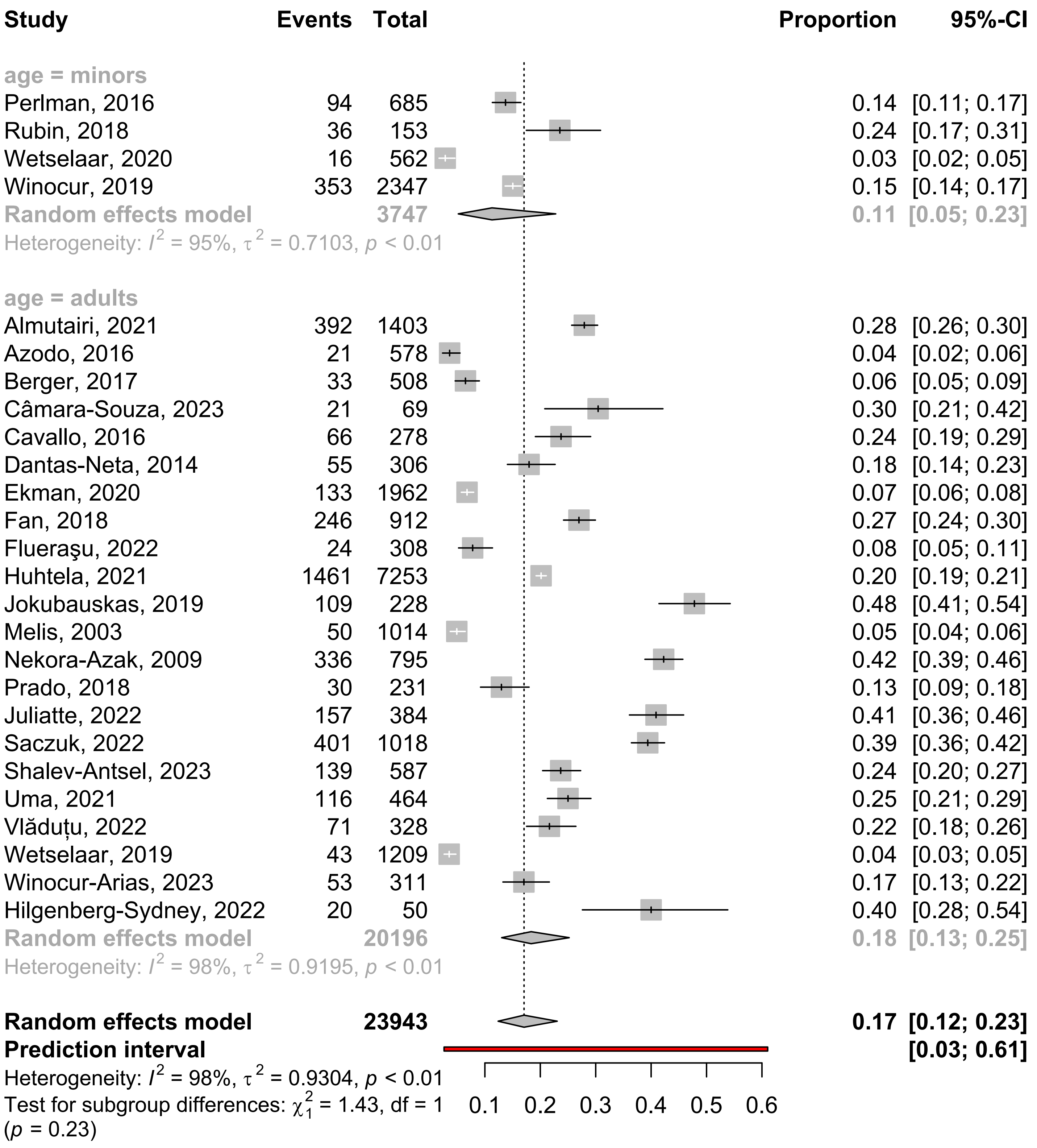
Study Events Total Proportion 95%-CI
age = minors
Perlman, 2016 94 685 0.14 [0.11; 0.17]
Rubin, 2018 36 153 0.24 [0.17; 0.31]
Wetselaar, 2020 16 562 0.03 [0.02; 0.05]
Winocur, 2019 353 2347 0.15 [0.14; 0.17]
Random effects model 3747 0.11 [0.05; 0.23]
Heterogeneity: I^2 = 95%, τ^2 = 0.7103, p < 0.01
age = adults
Almutairi, 2021 392 1403 0.28 [0.26; 0.30]
Azodo, 2016 21 578 0.04 [0.02; 0.06]
Berger, 2017 33 508 0.06 [0.05; 0.09]
Câmara-Souza, 2023 21 69 0.30 [0.21; 0.42]
Cavallo, 2016 66 278 0.24 [0.19; 0.29]
Dantas-Neta, 2014 55 306 0.18 [0.14; 0.23]
Ekman, 2020 133 1962 0.07 [0.06; 0.08]
Fan, 2018 246 912 0.27 [0.24; 0.30]
Flueraşu, 2022 24 308 0.08 [0.05; 0.11]
Huhtela, 2021 1461 7253 0.20 [0.19; 0.21]
Jokubauskas, 2019 109 228 0.48 [0.41; 0.54]
Melis, 2003 50 1014 0.05 [0.04; 0.06]
Nekora-Azak, 2009 336 795 0.42 [0.39; 0.46]
Prado, 2018 30 231 0.13 [0.09; 0.18]
Juliatte, 2022 157 384 0.41 [0.36; 0.46]
Saczuk, 2022 401 1018 0.39 [0.36; 0.42]
Shalev-Antsel, 2023 139 587 0.24 [0.20; 0.27]
Uma, 2021 116 464 0.25 [0.21; 0.29]
Vlăduțu, 2022 71 328 0.22 [0.18; 0.26]
Wetselaar, 2019 43 1209 0.04 [0.03; 0.05]
Winocur-Arias, 2023 53 311 0.17 [0.13; 0.22]
Hilgenberg-Sydney, 2022 20 50 0.40 [0.28; 0.54]
Random effects model 20196 0.18 [0.13; 0.25]
Heterogeneity: I^2 = 98%, τ^2 = 0.9195, p < 0.01
Random effects model 23943 0.17 [0.12; 0.23]
Prediction interval [0.03; 0.61]
Heterogeneity: I^2 = 98%, τ^2 = 0.9304, p < 0.01
Test for subgroup differences: χ^2_1 = 1.43, df = 1 (p = 0.23)
0.1 0.2 0.3 0.4 0.5 0.6

Figure S19. Global prevalence of males awake bruxism by age.

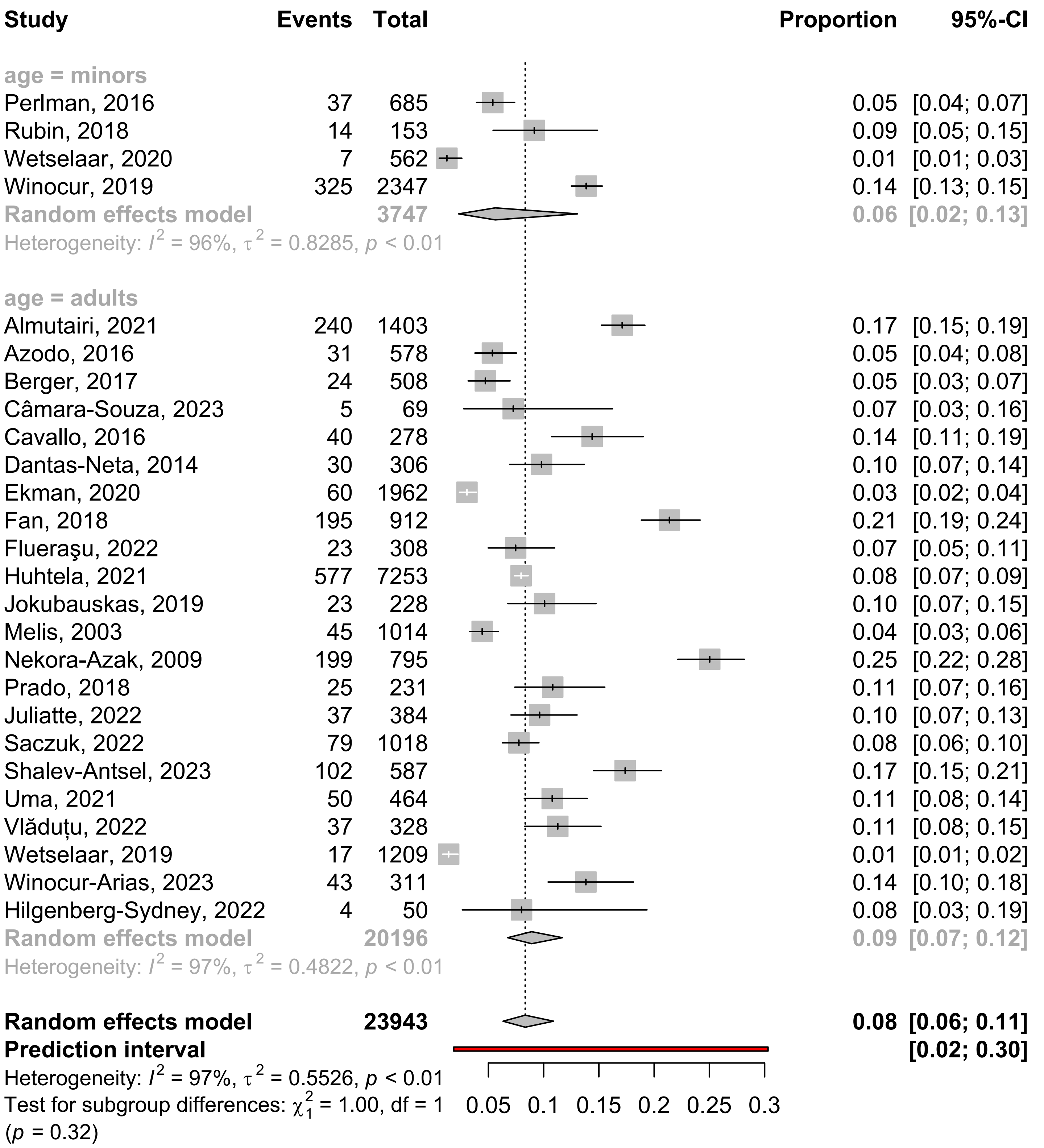
Study Events Total Proportion 95%-CI
age = minors
Perlman, 2016 37 685 0.05 [0.04; 0.07]
Rubin, 2018 14 153 0.09 [0.05; 0.15]
Wetselaar, 2020 7 562 0.01 [0.01; 0.03]
Winocur, 2019 325 2347 0.14 [0.13; 0.15]
Random effects model 3747 0.06 [0.02; 0.13]
Heterogeneity: I^2 = 96%, τ^2 = 0.8285, p < 0.01
age = adults
Almutairi, 2021 240 1403 0.17 [0.15; 0.19]
Azodo, 2016 31 578 0.05 [0.04; 0.08]
Berger, 2017 24 508 0.05 [0.03; 0.07]
Câmara-Souza, 2023 5 69 0.07 [0.03; 0.16]
Cavallo, 2016 40 278 0.14 [0.11; 0.19]
Dantas-Neta, 2014 30 306 0.10 [0.07; 0.14]
Ekman, 2020 60 1962 0.03 [0.02; 0.04]
Fan, 2018 195 912 0.21 [0.19; 0.24]
Flueraşu, 2022 23 308 0.07 [0.05; 0.11]
Huhtela, 2021 577 7253 0.08 [0.07; 0.09]
Jokubauskas, 2019 23 228 0.10 [0.07; 0.15]
Melis, 2003 45 1014 0.04 [0.03; 0.06]
Nekora-Azak, 2009 199 795 0.25 [0.22; 0.28]
Prado, 2018 25 231 0.11 [0.07; 0.16]
Juliatte, 2022 37 384 0.10 [0.07; 0.13]
Saczuk, 2022 79 1018 0.08 [0.06; 0.10]
Shalev-Antsel, 2023 102 587 0.17 [0.15; 0.21]
Uma, 2021 50 464 0.11 [0.08; 0.14]
Vlăduțu, 2022 37 328 0.11 [0.08; 0.15]
Wetselaar, 2019 17 1209 0.01 [0.01; 0.02]
Winocur-Arias, 2023 43 311 0.14 [0.10; 0.18]
Hilgenberg-Sydney, 2022 4 50 0.08 [0.03; 0.19]
Random effects model 20196 0.09 [0.07; 0.12]
Heterogeneity: I^2 = 97%, τ^2 = 0.4822, p < 0.01
Random effects model 23943 0.08 [0.06; 0.11]
Prediction interval [0.02; 0.30]
Heterogeneity: I^2 = 97%, τ^2 = 0.5526, p < 0.01
Test for subgroup differences: χ^2_1 = 1.00, df = 1 (p = 0.32)
0.05 0.1 0.15 0.2 0.25 0.3

Figure S20. Global prevalence of females awake bruxism by continent.

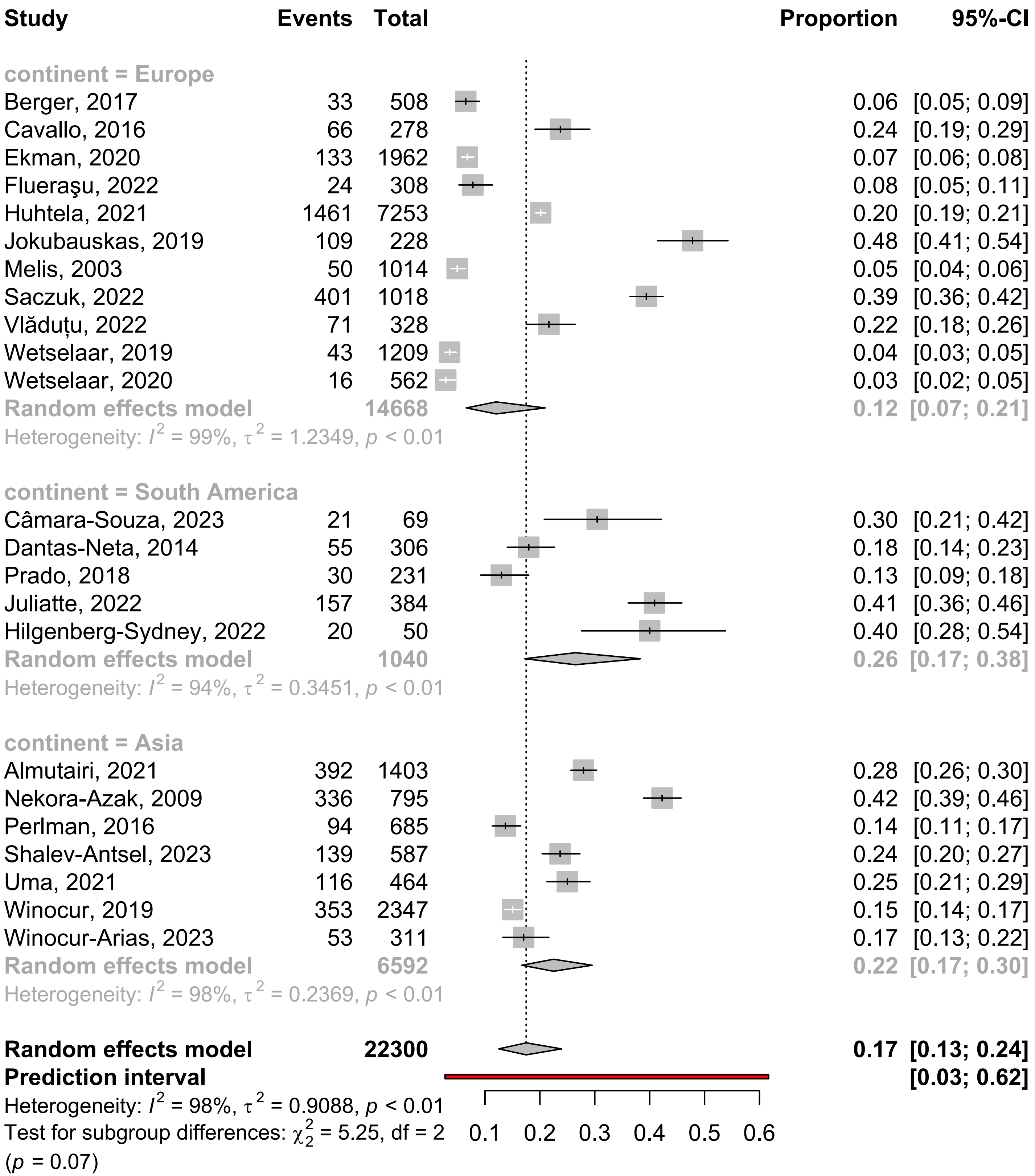

Study Events Total Proportion 95%-CI
continent = Europe
Berger, 2017 33 508 0.06 [0.05; 0.09]
Cavallo, 2016 66 278 0.24 [0.19; 0.29]
Ekman, 2020 133 1962 0.07 [0.06; 0.08]
Flueraşu, 2022 24 308 0.08 [0.05; 0.11]
Huhtela, 2021 1461 7253 0.20 [0.19; 0.21]
Jokubauskas, 2019 109 228 0.48 [0.41; 0.54]
Melis, 2003 50 1014 0.05 [0.04; 0.06]
Saczuk, 2022 401 1018 0.39 [0.36; 0.42]
Vlăduțu, 2022 71 328 0.22 [0.18; 0.26]
Wetselaar, 2019 43 1209 0.04 [0.03; 0.05]
Wetselaar, 2020 16 562 0.03 [0.02; 0.05]
Random effects model 14668 0.12 [0.07; 0.21]
Heterogeneity: I^2 = 99%, τ^2 = 1.2349, p < 0.01
continent = South America
Câmara-Souza, 2023 21 69 0.30 [0.21; 0.42]
Dantas-Neta, 2014 55 306 0.18 [0.14; 0.23]
Prado, 2018 30 231 0.13 [0.09; 0.18]
Juliatte, 2022 157 384 0.41 [0.36; 0.46]
Hilgenberg-Sydney, 2022 20 50 0.40 [0.28; 0.54]
Random effects model 1040 0.26 [0.17; 0.38]
Heterogeneity: I^2 = 94%, τ^2 = 0.3451, p < 0.01
continent = Asia
Almutairi, 2021 392 1403 0.28 [0.26; 0.30]
Nekora-Azak, 2009 336 795 0.42 [0.39; 0.46]
Perlman, 2016 94 685 0.14 [0.11; 0.17]
Shalev-Antsel, 2023 139 587 0.24 [0.20; 0.27]
Uma, 2021 116 464 0.25 [0.21; 0.29]
Winocur, 2019 353 2347 0.15 [0.14; 0.17]
Winocur-Arias, 2023 53 311 0.17 [0.13; 0.22]
Random effects model 6592 0.22 [0.17; 0.30]
Heterogeneity: I^2 = 98%, τ^2 = 0.2369, p < 0.01
Random effects model 22300 0.17 [0.13; 0.24]
Prediction interval [0.03; 0.62]
Heterogeneity: I^2 = 98%, τ^2 = 0.9088, p < 0.01
Test for subgroup differences: χ^2_2 = 5.25, df = 2 (p = 0.07)
0.1 0.2 0.3 0.4 0.5 0.6

Figure S21. Global prevalence of males awake bruxism by continent.

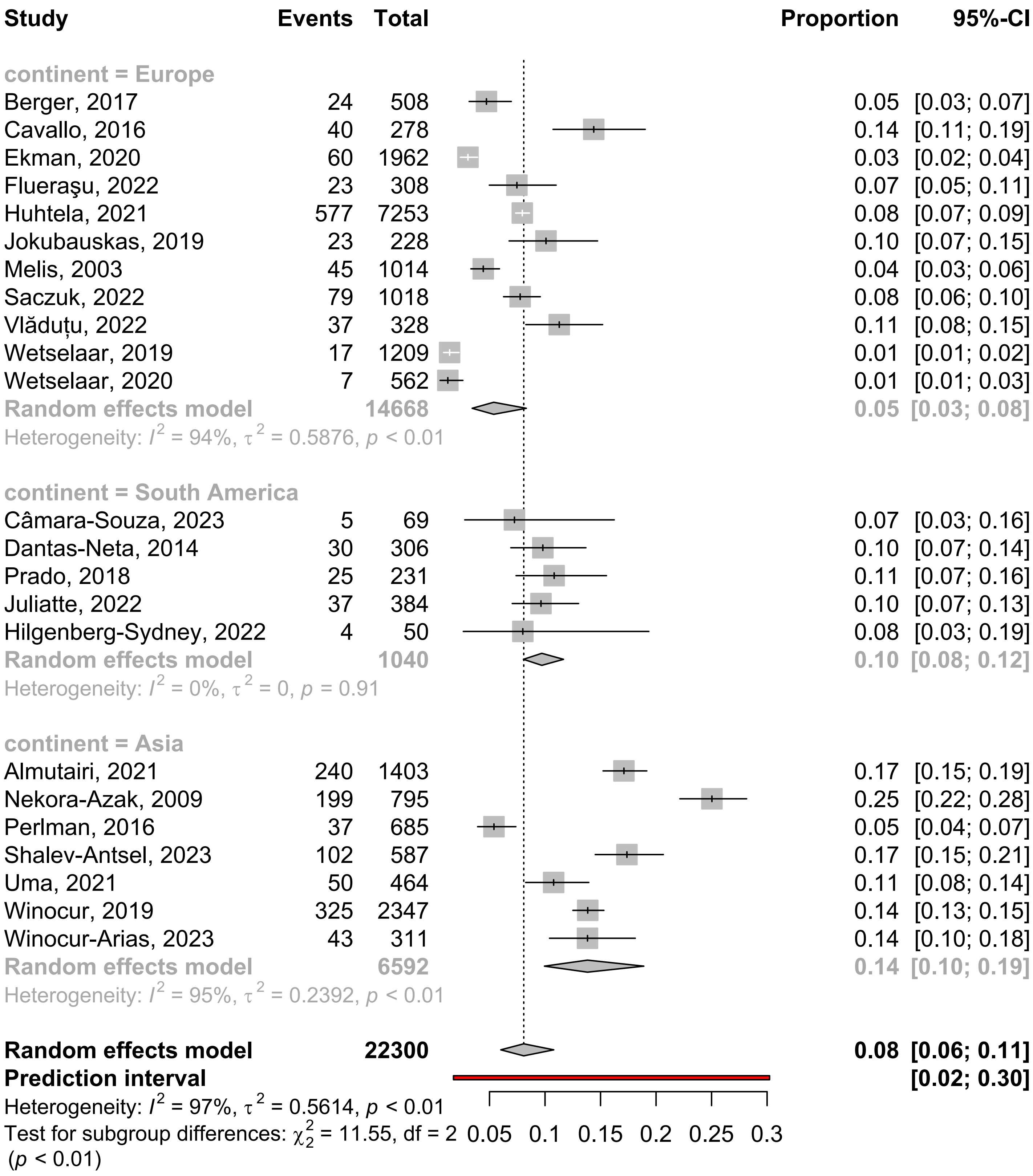

Study Events Total Proportion 95%-CI
continent = Europe
Berger, 2017 24 508 0.05 [0.03; 0.07]
Cavallo, 2016 40 278 0.14 [0.11; 0.19]
Ekman, 2020 60 1962 0.03 [0.02; 0.04]
Flueraşu, 2022 23 308 0.07 [0.05; 0.11]
Huhtela, 2021 577 7253 0.08 [0.07; 0.09]
Jokubauskas, 2019 23 228 0.10 [0.07; 0.15]
Melis, 2003 45 1014 0.04 [0.03; 0.06]
Saczuk, 2022 79 1018 0.08 [0.06; 0.10]
Vlăduțu, 2022 37 328 0.11 [0.08; 0.15]
Wetselaar, 2019 17 1209 0.01 [0.01; 0.02]
Wetselaar, 2020 7 562 0.01 [0.01; 0.03]
Random effects model 14668 0.05 [0.03; 0.08]
Heterogeneity: I^2 = 94%, τ^2 = 0.5876, p < 0.01
continent = South America
Câmara-Souza, 2023 5 69 0.07 [0.03; 0.16]
Dantas-Neta, 2014 30 306 0.10 [0.07; 0.14]
Prado, 2018 25 231 0.11 [0.07; 0.16]
Juliatte, 2022 37 384 0.10 [0.07; 0.13]
Hilgenberg-Sydney, 2022 4 50 0.08 [0.03; 0.19]
Random effects model 1040 0.10 [0.08; 0.12]
Heterogeneity: I^2 = 0%, τ^2 = 0, p = 0.91
continent = Asia
Almutairi, 2021 240 1403 0.17 [0.15; 0.19]
Nekora-Azak, 2009 199 795 0.25 [0.22; 0.28]
Perlman, 2016 37 685 0.05 [0.04; 0.07]
Shalev-Antsel, 2023 102 587 0.17 [0.15; 0.21]
Uma, 2021 50 464 0.11 [0.08; 0.14]
Winocur, 2019 325 2347 0.14 [0.13; 0.15]
Winocur-Arias, 2023 43 311 0.14 [0.10; 0.18]
Random effects model 6592 0.14 [0.10; 0.19]
Heterogeneity: I^2 = 95%, τ^2 = 0.2392, p < 0.01
Random effects model 22300 0.08 [0.06; 0.11]
Prediction interval [0.02; 0.30]
Heterogeneity: I^2 = 97%, τ^2 = 0.5614, p < 0.01
Test for subgroup differences: χ^2_2 = 11.55, df = 2 (p < 0.01)
0.05 0.1 0.15 0.2 0.25 0.3

Figure S22. Global prevalence of awake bruxism by continent and age.

Study	Events	Total	Proportion	95%-CI
**continent = Europe, age = minors**				
Selms, 2012	366	4205	0.09	[0.08; 0.10]
Wetselaar, 2020	23	562	0.04	[0.03; 0.06]
**Random effects model**		**4767**	**0.06**	**[0.04; 0.10]**
Heterogeneity: $I^2 = 93\%$, $\tau^2 = 0.1407$, $p < 0.01$				
**continent = Europe, age = adults**				
Berger, 2017	57	508	0.11	[0.09; 0.14]
Cavallo, 2016	106	278	0.38	[0.33; 0.44]
Ekman, 2020	193	1962	0.10	[0.09; 0.11]
Emodi-Perlman, 2020	372	1092	0.34	[0.31; 0.37]
Flueraşu, 2022	47	308	0.15	[0.12; 0.20]
Huhtela, 2021	2038	7253	0.28	[0.27; 0.29]
Jokubauskas, 2019	132	228	0.58	[0.51; 0.64]
Macfarlane, 2003	193	2137	0.09	[0.08; 0.10]
Manfredini , 2012	25	219	0.11	[0.08; 0.16]
Melis, 2003	95	1014	0.09	[0.08; 0.11]
Nykinen, 2023	11	46	0.24	[0.14; 0.38]
Osses-Anguita, 2023	85	274	0.31	[0.26; 0.37]
Panek , 2012	24	303	0.08	[0.05; 0.12]
Rauch , 2023	20	166	0.12	[0.08; 0.18]
Saczuk, 2022	480	1018	0.47	[0.44; 0.50]
Serra-Negra, 2021	48	136	0.35	[0.28; 0.44]
Sierwald, 2015	100	890	0.11	[0.09; 0.13]
Vlăduțu, 2022	108	328	0.33	[0.28; 0.38]
Wetselaar, 2019	60	1209	0.05	[0.04; 0.06]
Zani, 2019	11	30	0.37	[0.22; 0.55]
**Random effects model**		**19399**	**0.20**	**[0.14; 0.27]**
Heterogeneity: $I^2 = 99\%$, $\tau^2 = 0.7878$, $p < 0.01$				
**continent = South America, age = minors**				
Alonso, 2021	190	434	0.44	[0.39; 0.48]
Silva, 2023	110	739	0.15	[0.12; 0.18]
Junqueira , 2013	19	937	0.02	[0.01; 0.03]
Souza, 2020	198	403	0.49	[0.44; 0.54]
Prado, 2020	208	403	0.52	[0.47; 0.56]
**Random effects model**		**2916**	**0.24**	**[0.08; 0.54]**
Heterogeneity: $I^2 = 99\%$, $\tau^2 = 2.2343$, $p < 0.01$				
**continent = South America, age = adults**				
Aguiar, 2018	56	152	0.37	[0.30; 0.45]
Câmara-Souza, 2023	26	69	0.38	[0.27; 0.49]
Dantas-Neta, 2014	85	306	0.28	[0.23; 0.33]
Goulart, 2021	12	366	0.03	[0.02; 0.06]
Peixoto, 2021	345	641	0.54	[0.50; 0.58]
Pereira, 2020	22	38	0.58	[0.42; 0.72]
Prado, 2018	55	231	0.24	[0.19; 0.30]
Juliatte, 2022	194	384	0.51	[0.46; 0.55]
Serra-Negra, 2021	21	69	0.30	[0.21; 0.42]
Hilgenberg-Sydney, 2022	24	50	0.48	[0.35; 0.61]
**Random effects model**		**2306**	**0.33**	**[0.21; 0.48]**
Heterogeneity: $I^2 = 96\%$, $\tau^2 = 0.9483$, $p < 0.01$				
**continent = Asia, age = minors**				
Emodi-Perlman, 2016	192	1000	0.19	[0.17; 0.22]
Perlman, 2016	131	685	0.19	[0.16; 0.22]
Winocur, 2019	678	2347	0.29	[0.27; 0.31]
**Random effects model**		**4032**	**0.22**	**[0.18; 0.28]**
Heterogeneity: $I^2 = 96\%$, $\tau^2 = 0.0626$, $p < 0.01$				
**continent = Asia, age = adults**				
Almutairi, 2021	632	1403	0.45	[0.42; 0.48]
Başpınar, 2023	54	249	0.22	[0.17; 0.27]
Eli, 2022	31	304	0.10	[0.07; 0.14]
Emodi-Perlman, 2020	111	700	0.16	[0.13; 0.19]
Hermesh, 2014	1	33	0.03	[0.00; 0.17]
Khayat, 2019	22	149	0.15	[0.10; 0.21]
Levartovsky, 2022	90	387	0.23	[0.19; 0.28]
Manfredini , 2012	43	397	0.11	[0.08; 0.14]
Nekora-Azak, 2009	535	795	0.67	[0.64; 0.70]
Rao, 2011	87	147	0.59	[0.51; 0.67]
Shalev-Antsel, 2023	241	587	0.41	[0.37; 0.45]
Phuong, 2020	133	568	0.23	[0.20; 0.27]
Tinastepe, 2021	97	167	0.58	[0.50; 0.65]
Uma, 2021	166	464	0.36	[0.32; 0.40]
Winocur-Arias, 2023	96	311	0.31	[0.26; 0.36]
Yoshinaka , 2010	42	664	0.06	[0.05; 0.08]
**Random effects model**		**7325**	**0.25**	**[0.17; 0.36]**
Heterogeneity: $I^2 = 98\%$, $\tau^2 = 1.0854$, $p < 0.01$				
**Random effects model**		**40745**	**0.23**	**[0.18; 0.28]**
**Prediction interval**				**[0.03; 0.72]**

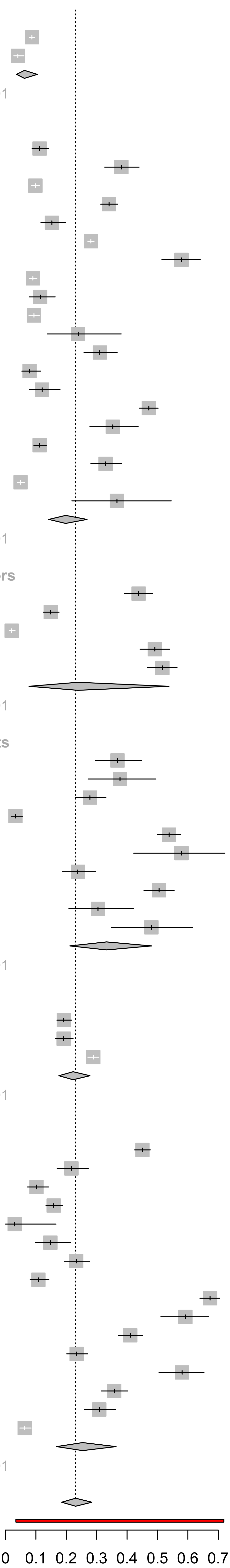

Heterogeneity: $I^2 = 99\%$, $\tau^2 = 1.1186$, $p = 0$
Test for subgroup differences: $\chi^2_5 = 28.13$, df = 5 ($p < 0.01$)

Figure S23. Global prevalence of female awake bruxism by continent and age.

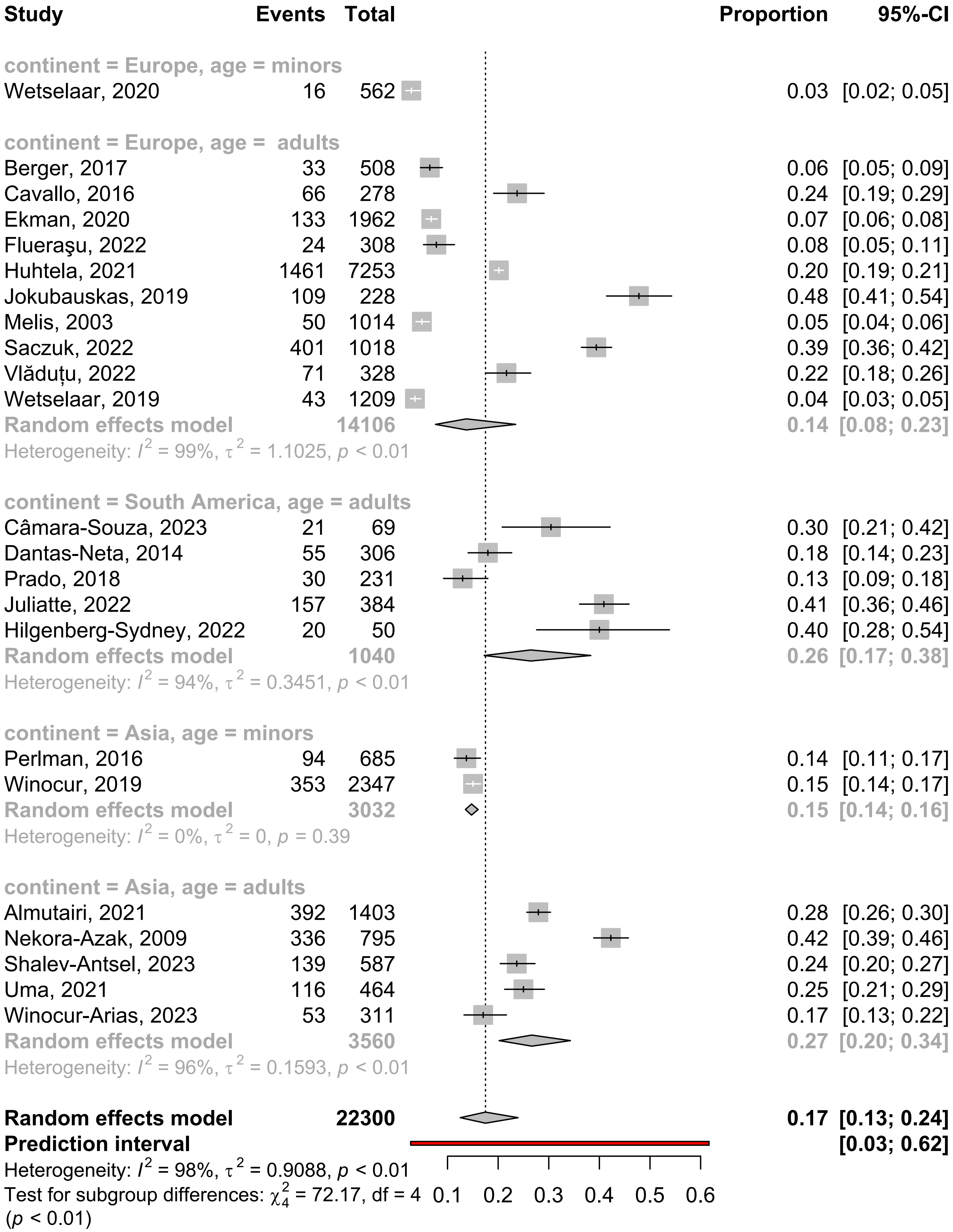
Study Events Total Proportion 95%-CI
continent = Europe, age = minors
Wetselaar, 2020 16 562 0.03 [0.02; 0.05]
continent = Europe, age = adults
Berger, 2017 33 508 0.06 [0.05; 0.09]
Cavallo, 2016 66 278 0.24 [0.19; 0.29]
Ekman, 2020 133 1962 0.07 [0.06; 0.08]
Flueraşu, 2022 24 308 0.08 [0.05; 0.11]
Huhtela, 2021 1461 7253 0.20 [0.19; 0.21]
Jokubauskas, 2019 109 228 0.48 [0.41; 0.54]
Melis, 2003 50 1014 0.05 [0.04; 0.06]
Saczuk, 2022 401 1018 0.39 [0.36; 0.42]
Vlăduțu, 2022 71 328 0.22 [0.18; 0.26]
Wetselaar, 2019 43 1209 0.04 [0.03; 0.05]
Random effects model 14106 0.14 [0.08; 0.23]
Heterogeneity: I^2 = 99%, τ^2 = 1.1025, p < 0.01
continent = South America, age = adults
Câmara-Souza, 2023 21 69 0.30 [0.21; 0.42]
Dantas-Neta, 2014 55 306 0.18 [0.14; 0.23]
Prado, 2018 30 231 0.13 [0.09; 0.18]
Juliatte, 2022 157 384 0.41 [0.36; 0.46]
Hilgenberg-Sydney, 2022 20 50 0.40 [0.28; 0.54]
Random effects model 1040 0.26 [0.17; 0.38]
Heterogeneity: I^2 = 94%, τ^2 = 0.3451, p < 0.01
continent = Asia, age = minors
Perlman, 2016 94 685 0.14 [0.11; 0.17]
Winocur, 2019 353 2347 0.15 [0.14; 0.17]
Random effects model 3032 0.15 [0.14; 0.16]
Heterogeneity: I^2 = 0%, τ^2 = 0, p = 0.39
continent = Asia, age = adults
Almutairi, 2021 392 1403 0.28 [0.26; 0.30]
Nekora-Azak, 2009 336 795 0.42 [0.39; 0.46]
Shalev-Antsel, 2023 139 587 0.24 [0.20; 0.27]
Uma, 2021 116 464 0.25 [0.21; 0.29]
Winocur-Arias, 2023 53 311 0.17 [0.13; 0.22]
Random effects model 3560 0.27 [0.20; 0.34]
Heterogeneity: I^2 = 96%, τ^2 = 0.1593, p < 0.01
Random effects model 22300 0.17 [0.13; 0.24]
Prediction interval [0.03; 0.62]
Heterogeneity: I^2 = 98%, τ^2 = 0.9088, p < 0.01
Test for subgroup differences: χ^2_4 = 72.17, df = 4 (p < 0.01)
0.1 0.2 0.3 0.4 0.5 0.6

Figure S24. Global prevalence of male awake bruxism by continent and age.

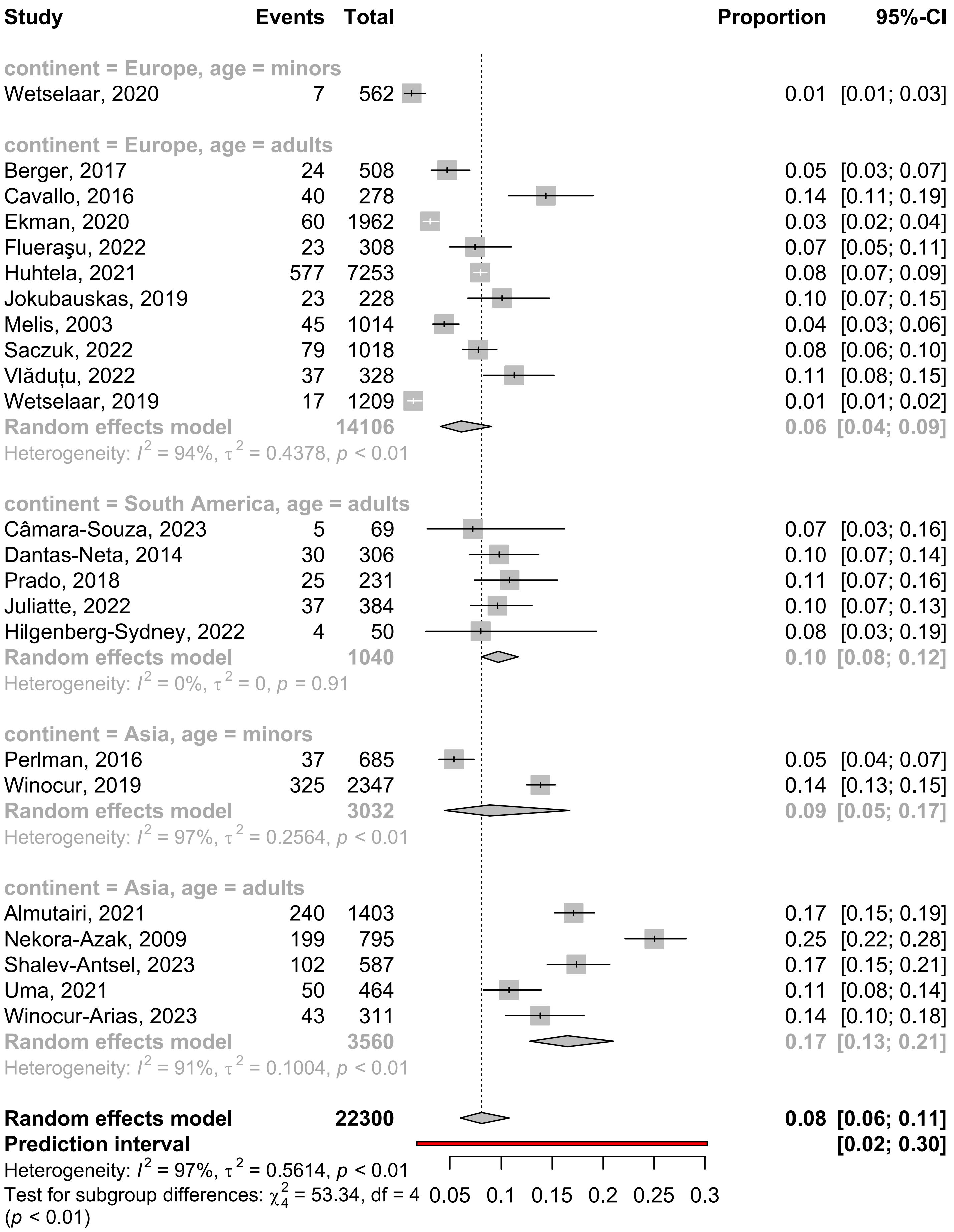
Study Events Total Proportion 95%-CI
continent = Europe, age = minors
Wetselaar, 2020 7 562 0.01 [0.01; 0.03]
continent = Europe, age = adults
Berger, 2017 24 508 0.05 [0.03; 0.07]
Cavallo, 2016 40 278 0.14 [0.11; 0.19]
Ekman, 2020 60 1962 0.03 [0.02; 0.04]
Flueraşu, 2022 23 308 0.07 [0.05; 0.11]
Huhtela, 2021 577 7253 0.08 [0.07; 0.09]
Jokubauskas, 2019 23 228 0.10 [0.07; 0.15]
Melis, 2003 45 1014 0.04 [0.03; 0.06]
Saczuk, 2022 79 1018 0.08 [0.06; 0.10]
Vlăduțu, 2022 37 328 0.11 [0.08; 0.15]
Wetselaar, 2019 17 1209 0.01 [0.01; 0.02]
Random effects model 14106 0.06 [0.04; 0.09]
Heterogeneity: I^2 = 94%, τ^2 = 0.4378, p < 0.01
continent = South America, age = adults
Câmara-Souza, 2023 5 69 0.07 [0.03; 0.16]
Dantas-Neta, 2014 30 306 0.10 [0.07; 0.14]
Prado, 2018 25 231 0.11 [0.07; 0.16]
Juliatte, 2022 37 384 0.10 [0.07; 0.13]
Hilgenberg-Sydney, 2022 4 50 0.08 [0.03; 0.19]
Random effects model 1040 0.10 [0.08; 0.12]
Heterogeneity: I^2 = 0%, τ^2 = 0, p = 0.91
continent = Asia, age = minors
Perlman, 2016 37 685 0.05 [0.04; 0.07]
Winocur, 2019 325 2347 0.14 [0.13; 0.15]
Random effects model 3032 0.09 [0.05; 0.17]
Heterogeneity: I^2 = 97%, τ^2 = 0.2564, p < 0.01
continent = Asia, age = adults
Almutairi, 2021 240 1403 0.17 [0.15; 0.19]
Nekora-Azak, 2009 199 795 0.25 [0.22; 0.28]
Shalev-Antsel, 2023 102 587 0.17 [0.15; 0.21]
Uma, 2021 50 464 0.11 [0.08; 0.14]
Winocur-Arias, 2023 43 311 0.14 [0.10; 0.18]
Random effects model 3560 0.17 [0.13; 0.21]
Heterogeneity: I^2 = 91%, τ^2 = 0.1004, p < 0.01
Random effects model 22300 0.08 [0.06; 0.11]
Prediction interval [0.02; 0.30]
Heterogeneity: I^2 = 97%, τ^2 = 0.5614, p < 0.01
Test for subgroup differences: χ^2_4 = 53.34, df = 4 (p < 0.01)
0.05 0.1 0.15 0.2 0.25 0.3

Figure S24. Global prevalence of sleep bruxism by polysomnography.

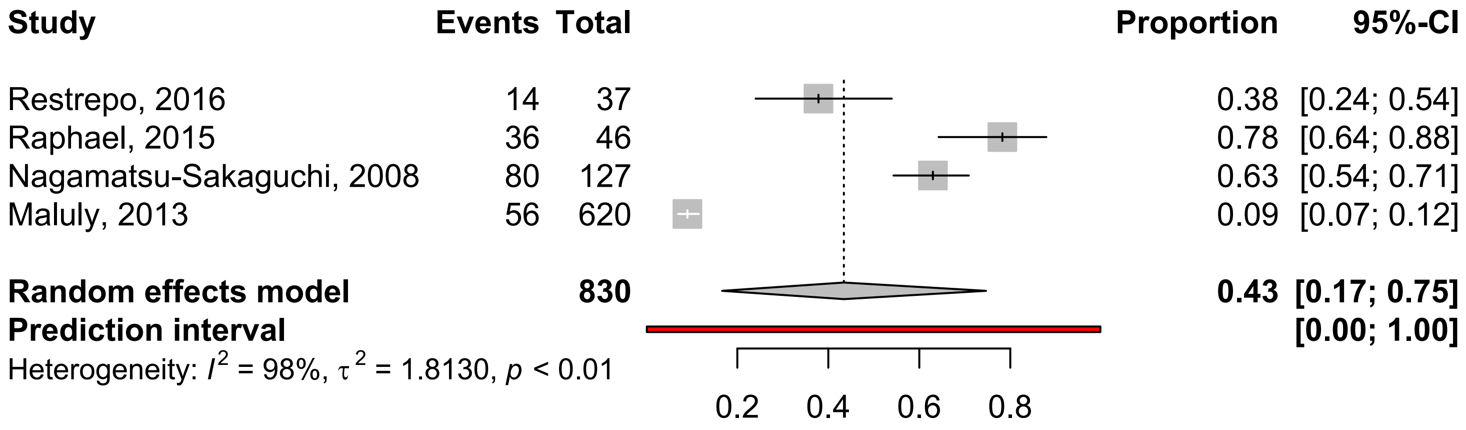

Study
Events
Total
Proportion
95%-CI
Restrepo, 2016
14
37
0.38
[0.24; 0.54]
Raphael, 2015
36
46
0.78
[0.64; 0.88]
Nagamatsu-Sakaguchi, 2008
80
127
0.63
[0.54; 0.71]
Maluly, 2013
56
620
0.09
[0.07; 0.12]
Random effects model
830
0.43
[0.17; 0.75]
Prediction interval
[0.00; 1.00]
Heterogeneity: I^2 = 98%, τ^2 = 1.8130, p < 0.01
0.2
0.4
0.6
0.8
