# Supplementary material for: Global Prevalence of Sleep Bruxism and Awake Bruxism in Pediatric and Adult Populations: A Systematic Review and Meta-Analysis
Source: J Clin Med. 2024 Jul 22;13(14):4259. doi: 10.3390/jcm13144259 (PMC11278015; doi:10.3390/jcm13144259)
Supplement: Supplementary file 1 [file jcm-13-04259-s001.zip › Supplementary Material S11 Presentation of funnel plots..pdf]

Global Prevalence of Sleep Bruxism and Awake Bruxism in Pediatric and Adult Populations: A Systematic Review and Meta-Analysis

Grzegorz Zieliński [1,*], Agnieszka Pająk [2], Marcin Wójcicki [3]

[1] Department of Sports Medicine, Medical University of Lublin, 20-093 Lublin, Poland
[2] Clinic of Anaesthesiology and Paediatric Intensive Care, Medical University of Lublin, Gebali Str. 6, 20-093 Lublin, Poland
[3] Independent Unit of Functional Masticatory Disorder, Medical University of Lublin, 20-093 Lublin, Poland

***** Correspondence: grzegorz.zielinski@umlub.pl

Figure S1. Global prevalence of bruxism regardless of type – funnel plots.

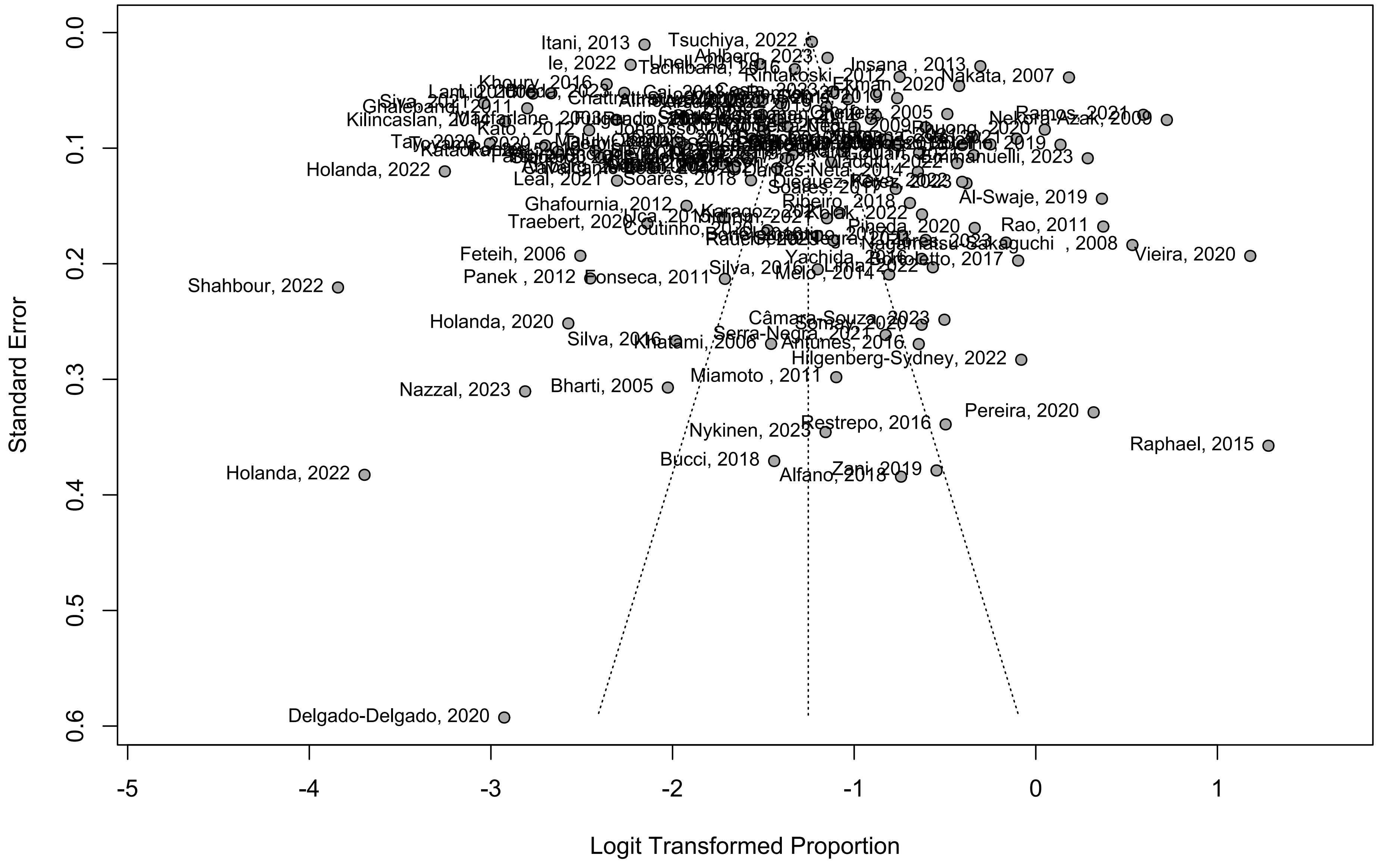
Standard Error
Logit Transformed Proportion
0.0
0.1
0.2
0.3
0.4
0.5
0.6
-5
-4
-3
-2
-1
0
1
Itani, 2013
Tsuchiya, 2022
Ie, 2022
Insana , 2013
Nakata, 2007
Khoury, 2016
Ekman, 2020
Ramos, 2021
Kilincaslan, 2014
Holanda, 2022
Leal, 2021
Soares, 2018
Al-Swaje, 2019
Ghafournia, 2012
Traebert, 2020
Rao, 2011
Feteih, 2006
Vieira, 2020
Panek , 2012
Fonseca, 2011
Shahbour, 2022
Holanda, 2020
Hilgenberg-Sydney, 2022
Miamoto , 2011
Nazzal, 2023
Bharti, 2005
Pereira, 2020
Restrepo, 2016
Nykinen, 2023
Raphael, 2015
Bucci, 2018
Holanda, 2022
Delgado-Delgado, 2020

Figure S2. Global prevalence of sleep bruxism – funnel plots.

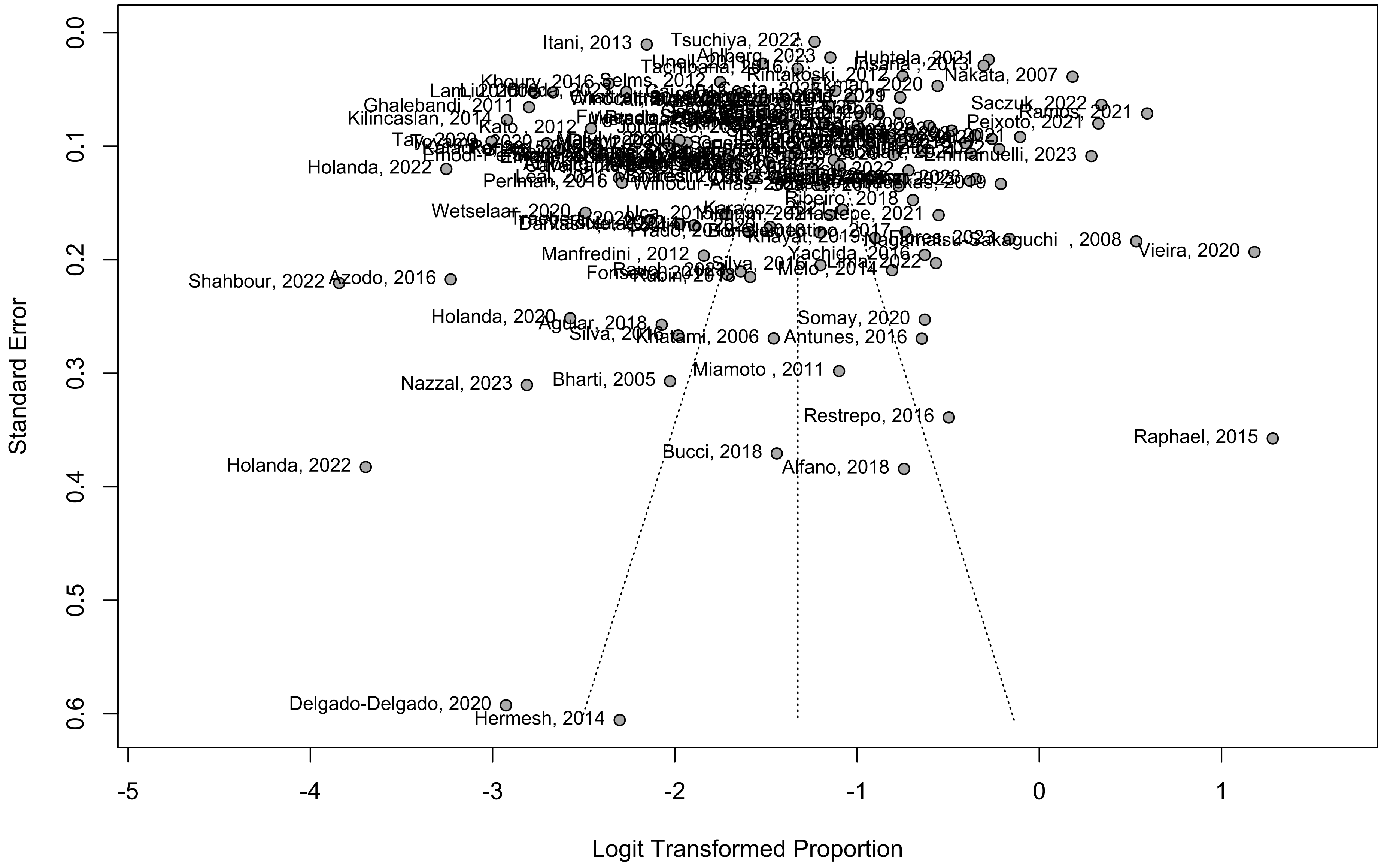
Standard Error
Logit Transformed Proportion
0.0
0.1
0.2
0.3
0.4
0.5
0.6
-5
-4
-3
-2
-1
0
1
Itani, 2013
Tsuchiya, 2022
Ahlberg, 2023
Huhtela, 2021
Nakata, 2007
Rintakoski, 2012
Ekman, 2020
Ghalebandi, 2011
Kilincaslan, 2014
Kato , 2012
Saczuk, 2022
Ramos, 2021
Peixoto, 2021
Emmanuelli, 2023
Holanda, 2022
Perlman, 2016
Ribeiro, 2018
Wetselaar, 2020
Nagamatsu-Sakaguchi , 2008
Vieira, 2020
Manfredini , 2012
Yachida, 2016
Melo , 2014
Shahbour, 2022
Azodo, 2016
Holanda, 2020
Aguiar, 2018
Khatami, 2006
Antunes, 2016
Somay, 2020
Miamoto , 2011
Nazzal, 2023
Bharti, 2005
Restrepo, 2016
Raphael, 2015
Holanda, 2022
Bucci, 2018
Alfano, 2018
Delgado-Delgado, 2020
Hermesh, 2014

Figure S3. Global prevalence of female sleep bruxism – funnel plots.

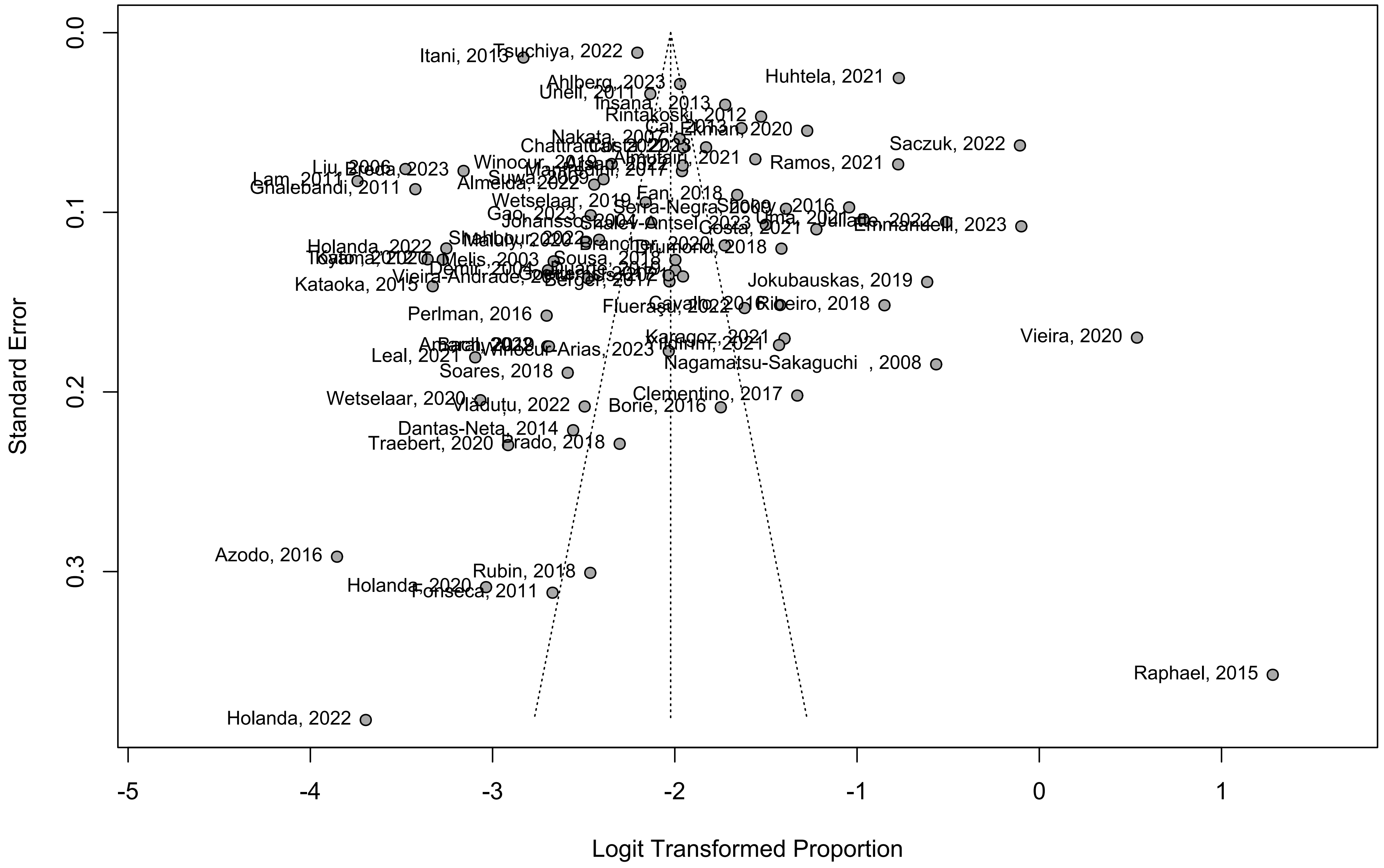

Standard Error
Logit Transformed Proportion
0.0
0.1
0.2
0.3
-5
-4
-3
-2
-1
0
1
Itani, 2013
Tsuchiya, 2022
Huhtela, 2021
Saczuk, 2022
Ramos, 2021
Gnalebandi, 2011
Almeida, 2022
Fan, 2018
Costa, 2021
Holanda, 2022
Jokubauskas, 2019
Kataoka, 2015
Berger, 2017
Ribeiro, 2018
Perlman, 2016
Vieira, 2020
Karagoz, 2021
Nagamatsu-Sakaguchi , 2008
Soares, 2018
Clementino, 2017
Wetselaar, 2020
Borie, 2016
Dantas-Neta, 2014
Traebert, 2020
Prado, 2018
Azodo, 2016
Rubin, 2018
Fonseca, 2011
Raphael, 2015
Holanda, 2022

Figure S4. Global prevalence of male sleep bruxism – funnel plots.

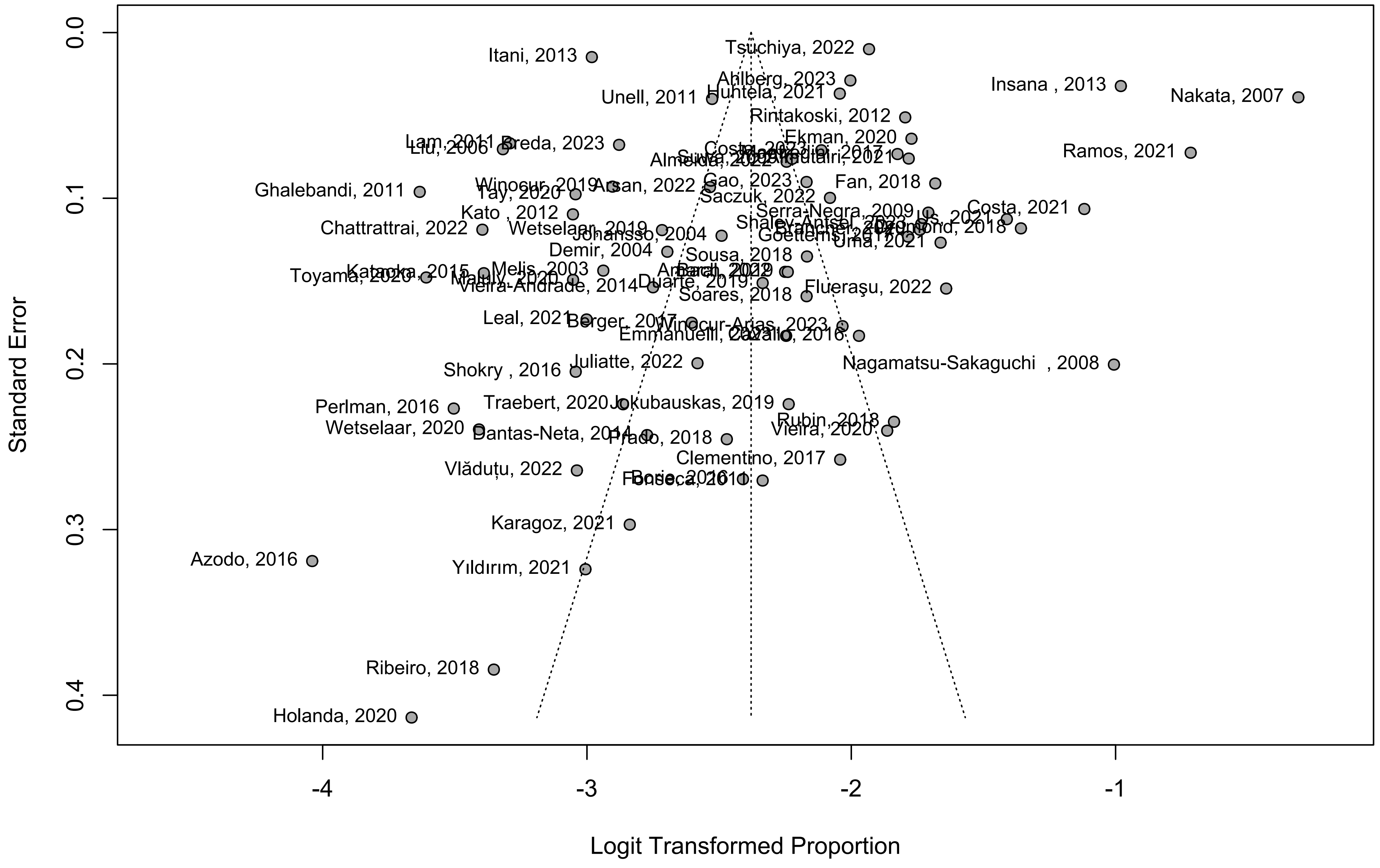
Standard Error
Logit Transformed Proportion
0.0
0.1
0.2
0.3
0.4
-4
-3
-2
-1
Itani, 2013
Tsuchiya, 2022
Ahlberg, 2023
Unell, 2011
Huhtela, 2021
Insana , 2013
Nakata, 2007
Rintakoski, 2012
Ekman, 2020
Breda, 2023
Liu, 2006
Ramos, 2021
Almeida, 2022
Ghalebandi, 2011
Winocur, 2019
Tay, 2020
Gao, 2023
Fan, 2018
Saczuk, 2022
Kato , 2012
Serra-Negra, 2009
Costa, 2021
Chattrattrai, 2022
Wetselaar, 2019
Johansso, 2004
Goettems, 2017
Uma, 2021
Demir, 2004
Sousa, 2018
Toyama, 2020
Melis, 2003
Vieira-Andrade, 2014
Duarte, 2019
Soares, 2018
Flueraşu, 2022
Leal, 2021
Berger, 2017
Winocur-Arias, 2023
Juliatte, 2022
Shokry , 2016
Nagamatsu-Sakaguchi , 2008
Perlman, 2016
Traebert, 2020
Jokubauskas, 2019
Rubin, 2018
Wetselaar, 2020
Dantas-Neta, 2014
Prado, 2018
Vieira, 2020
Clementino, 2017
Vlăduțu, 2022
Fonseca, 2011
Karagoz, 2021
Azodo, 2016
Yıldırım, 2021
Ribeiro, 2018
Holanda, 2020

Figure S5. Global prevalence of awake bruxism – funnel plots.

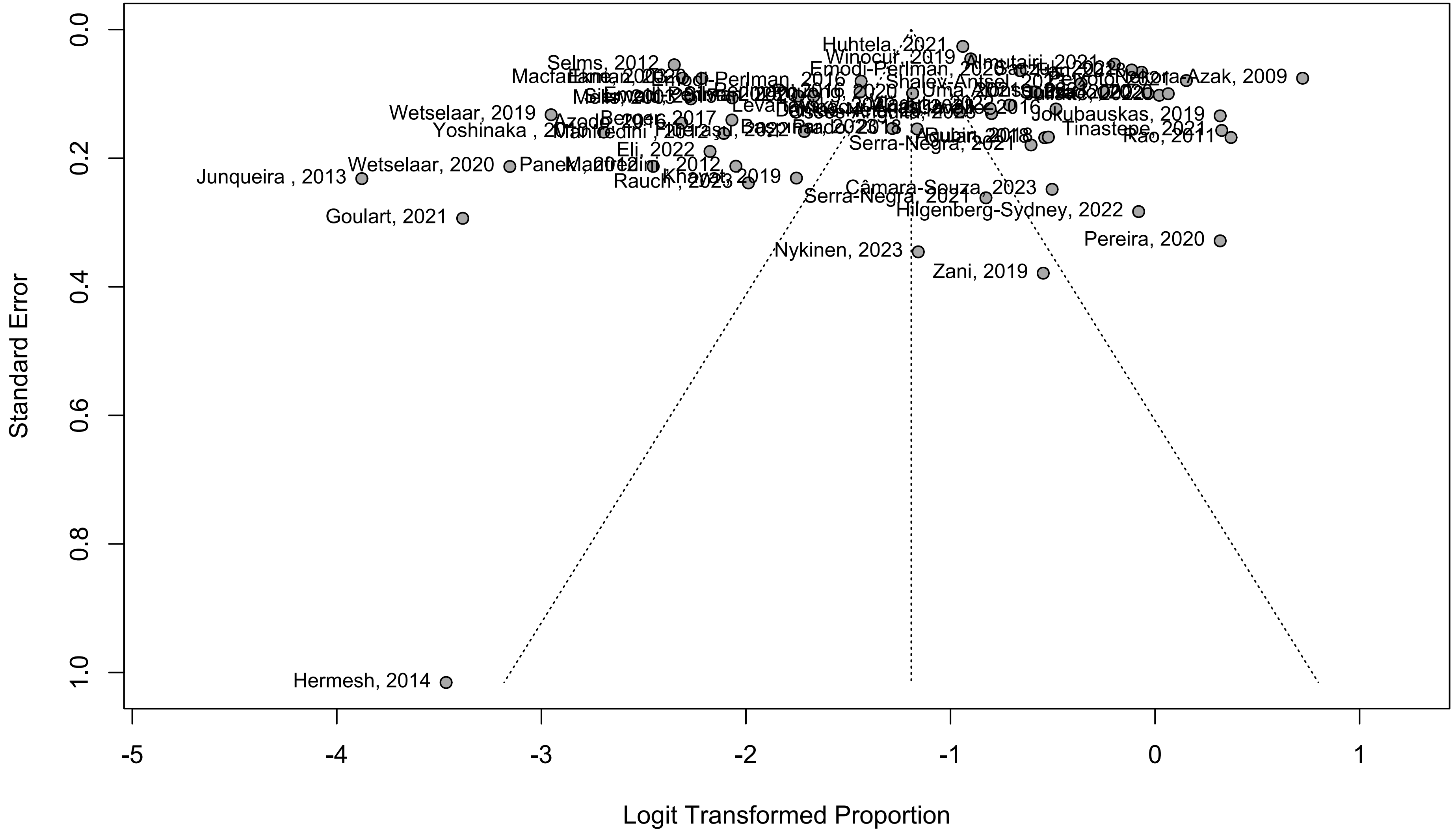
Huhtela, 2021
Winocur, 2019
Selms, 2012
Wetselaar, 2019
Jokubauskas, 2019
Tinastepe, 2021
Rao, 2011
Eli, 2022
Serra-Negra, 2021
Wetselaar, 2020
Junqueira , 2013
Khayat, 2019
Câmara-Souza, 2023
Serra-Negra, 2021
Hilgenberg-Sydney, 2022
Goulart, 2021
Pereira, 2020
Nykinen, 2023
Zani, 2019
Hermesh, 2014
Standard Error
0.0
0.2
0.4
0.6
0.8
1.0
-5
-4
-3
-2
-1
0
1
Logit Transformed Proportion

Figure S6. Global prevalence of female awake bruxism – funnel plots.

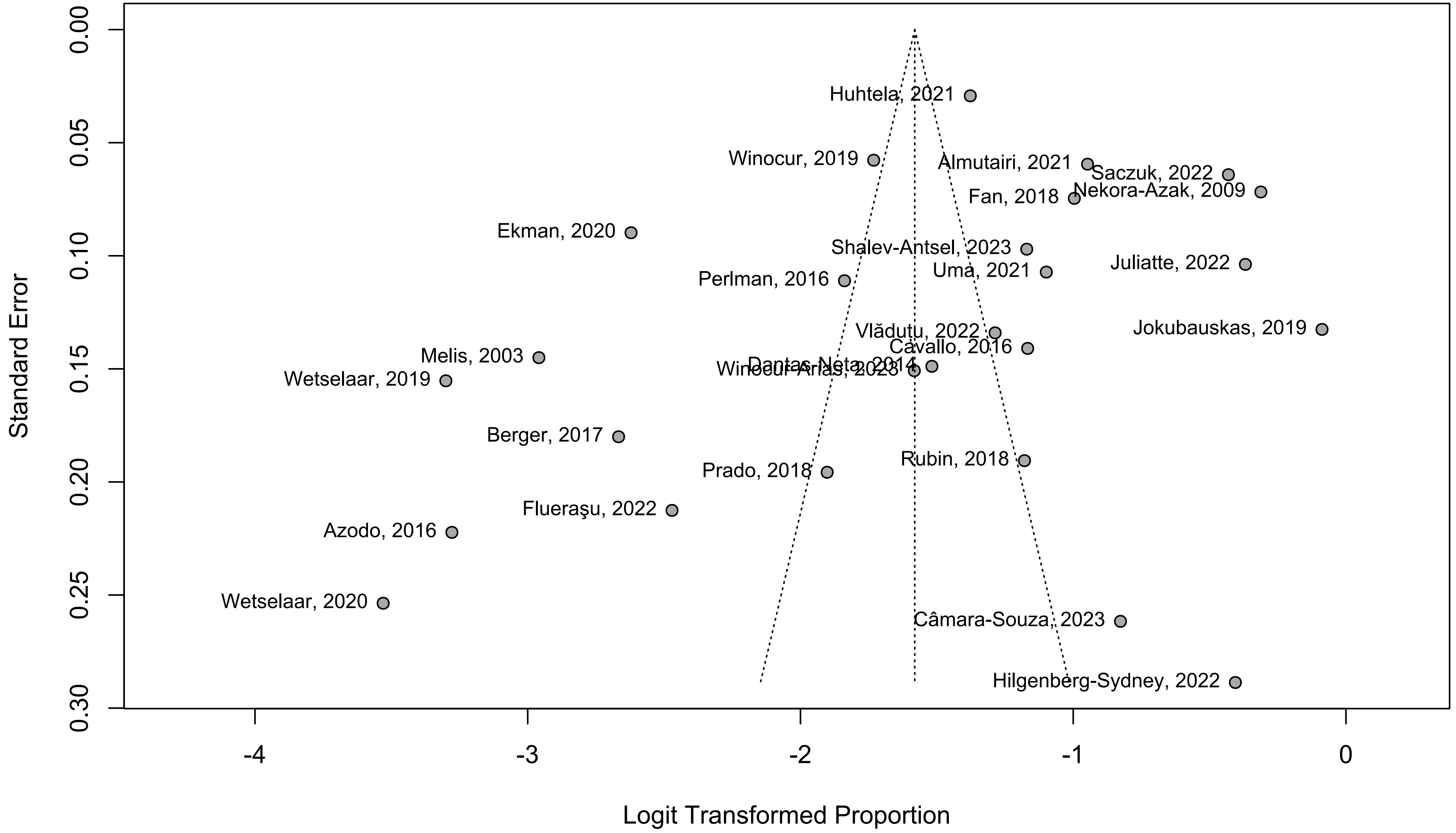

Standard Error
Logit Transformed Proportion
0.00
0.05
0.10
0.15
0.20
0.25
0.30
-4
-3
-2
-1
0
Huhtela, 2021
Winocur, 2019
Almutairi, 2021
Saczuk, 2022
Fan, 2018
Nekora-Azak, 2009
Ekman, 2020
Shalev-Antsel, 2023
Juliatte, 2022
Uma, 2021
Perlman, 2016
Jokubauskas, 2019
Vlădutu, 2022
Cavallo, 2016
Melis, 2003
Dantas-Neta, 2014
Winocur-Arias, 2023
Wetselaar, 2019
Berger, 2017
Rubin, 2018
Prado, 2018
Flueraşu, 2022
Azodo, 2016
Wetselaar, 2020
Câmara-Souza, 2023
Hilgenberg-Sydney, 2022

Figure S7. Global prevalence of male awake bruxism – funnel plots.

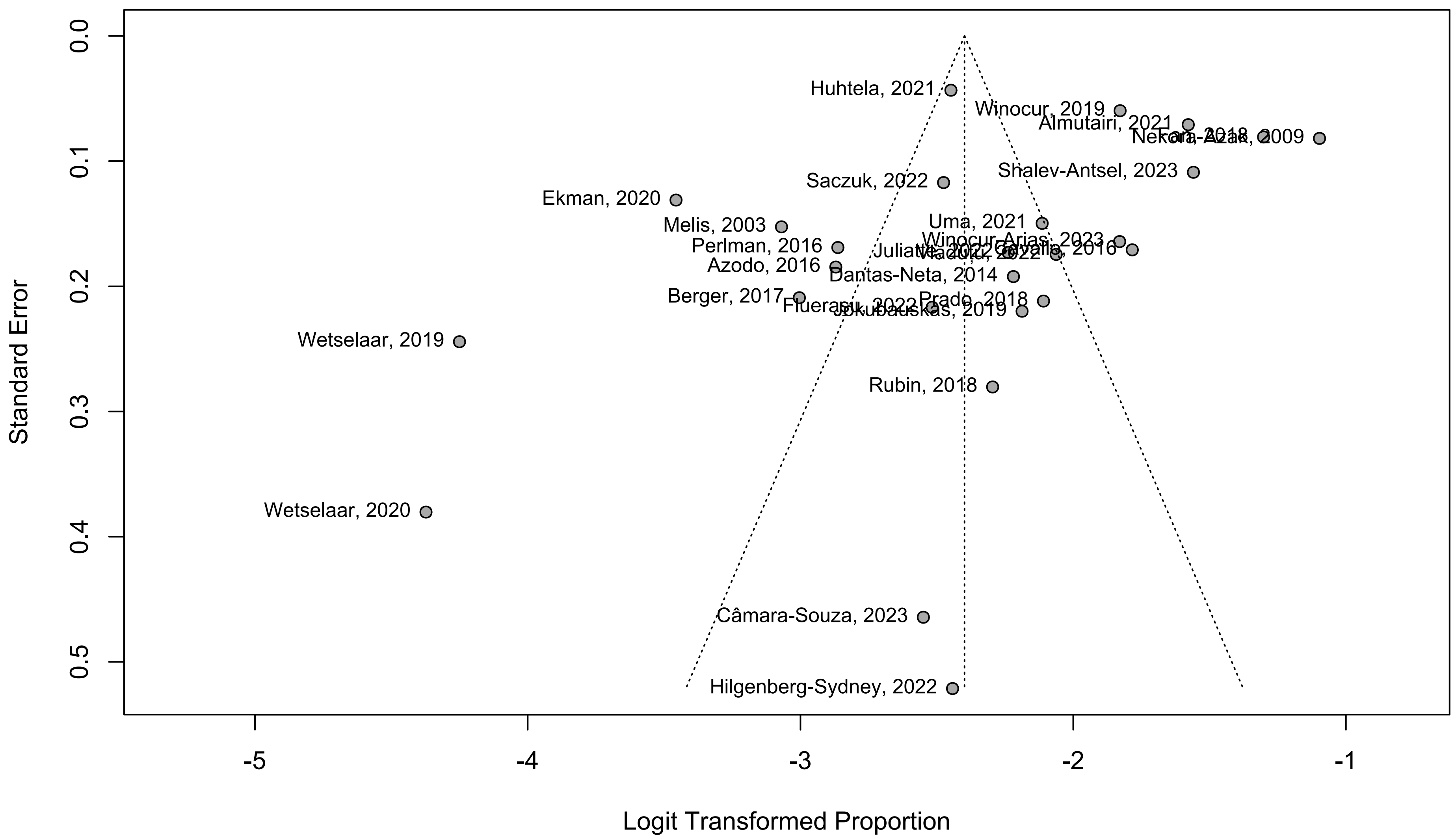

Huhtela, 2021
Winocur, 2019
Almutairi, 2021
Shalev-Antsel, 2023
Saczuk, 2022
Ekman, 2020
Melis, 2003
Uma, 2021
Perlman, 2016
Azodo, 2016
Dantas-Neta, 2014
Berger, 2017
Prado, 2018
Wetselaar, 2019
Rubin, 2018
Wetselaar, 2020
Câmara-Souza, 2023
Hilgenberg-Sydney, 2022
0.0
0.1
0.2
0.3
0.4
0.5
Standard Error
-5
-4
-3
-2
-1
Logit Transformed Proportion
