# Supplementary material for: Global Prevalence of Sleep Bruxism and Awake Bruxism in Pediatric and Adult Populations: A Systematic Review and Meta-Analysis
Source: J Clin Med. 2024 Jul 22;13(14):4259. doi: 10.3390/jcm13144259 (PMC11278015; doi:10.3390/jcm13144259)
Supplement: Supplementary file 1 [file jcm-13-04259-s001.zip › Supplementary Material S3 Adapted questionnaire for initial selection..pdf]

Systematic Review

Global Prevalence of Sleep Bruxism and Awake Bruxism in Pediatric and Adult Populations: A Systematic Review and Meta-Analysis

Grzegorz Zieliński [1,*], Agnieszka Pająk [2], Marcin Wójcicki [3]

[1] Department of Sports Medicine, Medical University of Lublin, 20-093 Lublin, Poland
[2] Clinic of Anaesthesiology and Paediatric Intensive Care, Medical University of Lublin, Gebali Str. 6, 20-093 Lublin, Poland
[3] Independent Unit of Functional Masticatory Disorder, Medical University of Lublin, 20-093 Lublin, Poland
* Correspondence: grzegorz.zielinski@umlub.pl

The quality assessment was conducted based on the adaptation of the questionnaire used by Berger et al. [1]. The questionnaire was expanded with questions related to the tools proposed by Giannakopoulos et al. [2] and Stang et al. [3]. Each question in the questionnaire was supported by relevant literature. Question Q6 was constructed to reflect the spectrum and diagnostic value of research on bruxism. It was created based on the literature [2,1,4–6] and the clinical experience of the authors. A threshold of 9 points was established based on consensus among all authors. This is because even the maximum score for questions Q1-Q4 and Q7 gives a value of 8 points. The study must obtain an additional 9th point for the significant value of the number of subjects in question Q5 (above 100 individuals [1]) or for the diagnostic value of bruxism Q6 [2,1,4–6]. The questionnaire was registered in the protocol in the Open Science Framework (OSF) under the number identifier: DOI 10.17605/OSF.IO/ZE786 [7].

Table S1. Adapted initial selection questionnaire.

Id.	Basis Questions		Points	Max. Pkt.
**Q1**	[2,3,8]	**Consent of the bioethics committee**		
		Lack of information	0	1
		Presented information on consent of the committee	1	
**Q2**	[2,3,8]	**Clearly defined populations:**		
		Provision of age information	1	3
		No information on age	0	
		Specifying sex	1	
		No information on sex	0	
		Provision of information on nationality or other information to help identify the test site	1	
		No information on nationality or other information to help identify the test site	0	
**Q3**	[1–3,8]	**Definition of inclusion criteria**	1	1
		No information on inclusion criterion	0	
**Q4**	[1–3,8]	**Definition of criteria exclusion**	1	1
		No information on the exclusion criterion for the study	0	
**Q5**	[1–3,8,9]	**Sample size**		
		≤99	0	3
		≥100	1	
		≥400	2	
		≥1000	3	
**Q6**	[2,1,4–6]	**Investigation of bruxism**		
		Lack of information or questionable diagnosis - e.g. single question	0	5
		Questionnaire, BruxApp	1	
		Clinical examination of the oral cavity, study based on American	2	

		Academy of Sleep Medicine (AASM), used BruxChecker		
		Polysomnography/Electromyography examination	5	
**Q7**	[1,8]	**Conflict of interest**		
		Current	0	2
		No information	1	
		Declared no conflict	2	
	**Max. pkt.**			16
		**Test acceptance threshold minimum 9 points**		

References

1. Berger, M.; Szalewski, L.; Bakalczuk, M.; Bakalczuk, G.; Bakalczuk, S.; Szkutnik, J. Association between Estrogen Levels and Temporomandibular Disorders: A Systematic Literature Review. *Menopause Rev. Menopauzalny* **2015**, *14*, 260–270, doi:10.5114/pm.2015.56538.
2. Giannakopoulos, N.N.; Rammelsberg, P.; Eberhard, L.; Schmitter, M. A New Instrument for Assessing the Quality of Studies on Prevalence. *Clin. Oral Investig.* **2012**, *16*, 781–788, doi:10.1007/s00784-011-0557-4.
3. Stang, A. Critical Evaluation of the Newcastle-Ottawa Scale for the Assessment of the Quality of Nonrandomized Studies in Meta-Analyses. *Eur. J. Epidemiol.* **2010**, *25*, 603–605, doi:10.1007/s10654-010-9491-z.
4. Bulanda, S.; Ilczuk-Rypuła, D.; Nitecka-Buchta, A.; Nowak, Z.; Baron, S.; Postek-Stefańska, L. Sleep Bruxism in Children: Etiology, Diagnosis, and Treatment-A Literature Review. *Int. J. Environ. Res. Public. Health* **2021**, *18*, 9544, doi:10.3390/ijerph18189544.
5. Bartolucci, M.L.; Incerti Parenti, S.; Bortolotti, F.; Della Godenza, V.; Vandi, S.; Pizza, F.; Plazzi, G.; Alessandri-Bonetti, G. Sleep Bruxism and Orofacial Pain in Patients with Sleep Disorders: A Controlled Cohort Study. *J. Clin. Med.* **2023**, *12*, 2997, doi:10.3390/jcm12082997.
6. Palinkas, M.; De, L.C.G.; Rodrigues, L.A.M.; Bataglion, C.; Si, éssere S.; Semprini, M.; Regalo, S.C.H. Comparative Capabilities of Clinical Assessment, Diagnostic Criteria, and Polysomnography in Detecting Sleep Bruxism. *J. Clin. Sleep Med. 11*, 1319–1325, doi:10.5664/jcsm.5196.
7. Zieliński, G.; Pająk, A.; Wójcicki, M. A Meta-Analysis of the Global Prevalence of Sleep Bruxism and Awake Bruxism in Pediatric and Adult Populations. **2024**, doi:10.17605/OSF.IO/ZE786.
8. Zieliński, G.; Byś, A. The Incidence, the Most Common Symptoms and Risk Factors of Altitude Sickness in Children. *Pediatr. Med. Rodz.* **2020**, *16*, 275–279, doi:10.15557/PiMR.2020.0050.
9. Kyriazos, T. Applied Psychometrics: Sample Size and Sample Power Considerations in Factor Analysis (EFA, CFA) and SEM in General. *Psychology* **2018**, *09*, 2207–2230, doi:10.4236/psych.2018.98126.
