# Supplementary material for: Global Prevalence of Sleep Bruxism and Awake Bruxism in Pediatric and Adult Populations: A Systematic Review and Meta-Analysis
Source: J Clin Med. 2024 Jul 22;13(14):4259. doi: 10.3390/jcm13144259 (PMC11278015; doi:10.3390/jcm13144259)
Supplement: Supplementary file 1 [file jcm-13-04259-s001.zip › Supplementary Material S4 The results of the evaluation of studies using an adapted questionnaire..pdf]

Systematic Review

Global Prevalence of Sleep Bruxism and Awake Bruxism in Pediatric and Adult Populations: A Systematic Review and Meta-Analysis

Grzegorz Zieliński [1,*], Agnieszka Pająk [2], Marcin Wójcicki [3]

[1] Department of Sports Medicine, Medical University of Lublin, 20-093 Lublin, Poland
[2] Clinic of Anaesthesiology and Paediatric Intensive Care, Medical University of Lublin, Gebali Str. 6, 20-093 Lublin, Poland
[3] Independent Unit of Functional Masticatory Disorder, Medical University of Lublin, 20-093 Lublin, Poland
* Correspondence: grzegorz.zielinski@umlub.pl

Table S1. The results of the evaluation of studies using an adapted questionnaire.

	autor	year	Q1	Q2	Q3	Q4	Q5	Q6	Q7	score
**[1]**	Aguiar	2018	1	3	1	1	2	0	1	9
**[2]**	Ahlberg	2023	1	3	1	1	3	0	1	10
**[3]**	Ahlberg	2008	1	3	1	1	1	1	1	9
**[4]**	Alfano	2018	1	3	1	1	1	1	2	10
**[5]**	Almeida	2022	1	3	1	1	0	1	2	9
**[6]**	Almutairi	2021	1	3	1	1	1	1	2	10
**[7]**	Alonso	2021	1	3	1	1	0	5	2	13
**[8]**	Al-Swaje	2019	1	2	1	1	1	1	2	9
**[9]**	Amaral	2022	1	3	1	1	3	0	2	11
**[10]**	Antunes	2016	1	3	1	1	0	2	1	9
**[11]**	Arsan	2022	1	3	1	1	2	0	1	9
**[12]**	Azodo	2016	1	3	1	1	3	0	2	11
**[13]**	Bach	2019	1	3	1	1	2	0	2	10
**[14]**	Başpınar	2023	1	3	1	1	0	1	2	9
**[15]**	Berger	2017	1	3	1	1	3	0	2	11
**[16]**	Bharti	2005	1	3	1	1	1	1	1	9
**[17]**	Bolsson	2023	1	2	1	1	2	1	2	10
**[18]**	Borie	2016	1	3	1	1	1	0	2	9
**[19]**	Bortoletto	2017	1	3	1	1	3	1	2	12
**[20]**	Botelho	2019	1	2	1	1	1	1	2	9
**[21]**	Bracci	2018	1	3	1	1	0	1	0	7
**[22]**	Brancher	2020	1	3	1	1	3	0	2	11
**[23]**	Breda	2023	1	3	1	1	2	2	1	11
**[24]**	Bucci	2018	1	3	1	1	3	2	2	13
**[25]**	Bucci	2022	1	3	1	1	1	1	0	8
**[26]**	Cai	2013	1	3	1	1	1	1	2	10
**[27]**	Câmara-Souza	2023	1	3	1	1	3	0	2	11
**[28]**	Carra	2011	1	2	1	1	3	0	2	10
**[29]**	Cavalcante-Leão	2017	1	3	1	1	2	0	2	10

**[30]**	Cavallo	2016	1	3	1	1	1	5	1	13
**[31]**	Chattrattrai	2022	1	3	1	1	1	2	2	11
**[32]**	Cheifetz	2005	1	3	1	1	3	0	2	11
**[33]**	Ciancaglini	2008	1	3	1	1	2	1	2	11
**[34]**	Clementino	2017	1	3	1	1	1	2	1	10
**[35]**	Colonna	2021	1	2	1	1	1	1	2	9
**[36]**	Costa	2021	1	3	1	1	2	0	2	10
**[37]**	Costa	2023	1	3	1	1	1	2	1	10
**[38]**	Coutinho	2020	1	3	1	1	1	1	1	9
**[39]**	Dantas-Neta	2014	1	3	1	1	2	0	2	10
**[40]**	Delgado-Delgado	2020	1	3	1	1	2	0	2	10
**[41]**	Demir	2004	1	3	2	1	1	0	1	9
**[42]**	Dias	2021	1	3	1	1	0	1	0	7
**[43]**	Diéguez-Pérez	2023	1	3	1	1	3	2	2	13
**[44]**	Drumond	2018	1	3	1	1	2	0	2	10
**[45]**	Duarte	2019	1	3	1	1	1	1	1	9
**[46]**	Ekman	2020	1	3	1	1	1	1	2	10
**[47]**	Eli	2022	1	3	1	1	0	5	2	13
**[48]**	Emmanuelli	2023	1	3	1	1	2	0	1	9
**[49]**	Emodi-Perlman	2020	1	3	1	1	1	0	2	9
**[50]**	Emodi-Perlman	2016	1	2	1	1	0	5	2	12
**[51]**	Fan	2018	1	3	1	1	3	0	1	10
**[52]**	Farsi	2003	1	3	1	1	2	1	1	10
**[53]**	Ferreira	2016	1	3	1	1	2	1	1	10
**[54]**	Feteih	2006	1	3	1	1	2	1	2	11
**[55]**	Flores	2023	1	3	1	1	3	0	2	11
**[56]**	Flueraşu	2022	1	3	1	1	0	2	2	10
**[57]**	Fonseca	2011	1	3	1	1	2	0	2	10
**[58]**	Fulgencio	2016	1	2	1	1	1	1	2	9
**[59]**	Gao	2023	1	3	1	1	2	0	2	10
**[60]**	Garde	2014	1	3	1	1	2	1	2	11
**[61]**	Ghafournia	2012	1	3	1	1	2	1	2	11
**[62]**	Ghalebandi	2011	1	3	1	1	3	1	1	11
**[63]**	Goettems	2017	1	3	1	1	1	2	2	11
**[64]**	Gomes	2018	1	2	1	1	2	1	2	10
**[65]**	Goulart	2021	1	3	1	1	1	1	1	9
**[66]**	Hermesh	2014	1	3	1	1	1	2	2	11
**[67]**	Hilgenberg-Sydney	2022	1	3	1	1	0	2	1	9
**[68]**	Holanda	2022	1	3	1	1	2	0	1	9
**[69]**	Holanda	2020	1	3	1	1	2	0	2	10
**[70]**	Huhtela	2021	1	3	1	1	2	1	1	10
**[71]**	Insana	2013	1	3	1	1	2	0	1	9
**[72]**	Itani	2013	1	3	1	1	3	0	2	11
**[73]**	Johansso	2004	1	3	1	1	3	2	1	12
**[74]**	Jokubauskas	2019	1	3	1	1	2	1	1	10
**[75]**	Juliatte	2022	1	3	1	1	2	0	2	10
**[76]**	Junqueira	2013	1	3	1	1	3	0	1	10

**[77]**	Karagoz	2021	1	3	1	1	1	1	2	10
**[78]**	Kataoka	2015	1	3	1	1	3	2	1	12
**[79]**	Kato	2012	1	3	1	1	3	0	2	11
**[80]**	Kaya	2022	1	3	1	1	1	1	1	9
**[81]**	Khatami	2006	1	3	1	1	3	2	1	12
**[82]**	Khayat	2019	1	3	1	1	2	1	2	11
**[83]**	Khoury	2016	1	3	1	1	3	0	0	9
**[84]**	Kilincaslan	2014	1	3	1	1	0	5	2	13
**[85]**	Kim	2017	1	3	1	1	1	1	2	10
**[86]**	Kolak	2022	1	3	1	1	1	2	2	11
**[87]**	Lam	2011	1	2	1	1	1	1	2	9
**[88]**	Leal	2021	1	3	1	1	2	0	1	9
**[89]**	Le	2022	1	2	1	1	2	1	1	9
**[90]**	Levartovsky	2022	1	3	1	1	1	2	2	11
**[91]**	Lima	2022	1	3	1	1	3	0	1	10
**[92]**	Liu	2006	1	3	1	1	0	1	2	9
**[93]**	Macfarlane	2003	1	3	1	1	1	2	1	10
**[94]**	Maluly	2020	1	3	1	1	3	1	2	12
**[95]**	Manfredini	2017	1	3	1	1	3	0	2	11
**[96]**	Manfredini	2012	1	3	1	1	3	0	0	9
**[97]**	Martynowicz	2019	1	3	1	1	1	5	0	12
**[98]**	Massignan	2019	1	3	1	1	2	1	2	11
**[99]**	Melis	2003	1	3	1	1	2	0	2	10
**[100]**	Melo	2014	1	3	1	1	2	2	1	11
**[101]**	Miamoto	2011	1	3	1	1	2	1	1	10
**[102]**	Montero	2017	1	3	1	1	3	1	2	12
**[103]**	Nagamatsu-Sakaguchi	2008	1	3	1	1	2	0	1	9
**[104]**	Nahás-Scocate	2014	1	3	1	1	2	0	2	10
**[105]**	Nakata	2007	1	3	1	1	1	1	2	10
**[106]**	Nazzal	2023	1	3	1	1	2	0	1	9
**[107]**	Nekora-Azak	2009	1	3	1	1	1	1	1	9
**[108]**	Ng	2009	1	3	1	1	0	0	1	7
**[109]**	Nykinen	2023	1	3	1	1	2	0	2	10
**[110]**	Okawara	2022	1	3	1	1	1	1	2	10
**[111]**	Osses-Anguita	2023	1	3	1	1	2	1	2	11
**[112]**	Panek	2012	1	3	1	1	2	0	2	10
**[113]**	Peixoto	2021	1	3	1	1	3	0	2	11
**[114]**	Pereira	2020	1	3	1	1	0	2	2	10
**[115]**	Perlman	2016	1	3	1	1	3	1	1	11
**[116]**	Pineda	2020	1	3	1	1	3	1	2	12
**[117]**	Pontes	2019	1	3	1	1	2	2	2	12
**[118]**	Prado	2018	1	3	1	1	2	2	1	11
**[119]**	Prado	2020	1	3	1	1	1	2	2	11
**[120]**	Prado	2019	1	3	1	1	1	1	2	10
**[121]**	Quadri	2015	1	3	1	1	2	0	2	10
**[122]**	Ramos	2021	1	3	1	1	3	1	1	11
**[123]**	Rana	2017	1	3	1	1	0	1	2	9
**[124]**	Rao	2011	1	3	1	1	0	1	2	9

**[125]**	Raphael	2015	1	3	1	1	1	1	2	10
**[126]**	Rauch	2023	1	3	1	1	2	2	1	11
**[127]**	Renner	2011	1	3	1	1	2	1	2	11
**[128]**	Restrepo	2016	1	3	1	1	1	0	2	9
**[129]**	Ribeiro	2018	1	2	1	1	2	1	2	10
**[130]**	Rintakoski	2012	1	3	1	1	2	0	2	10
**[131]**	Rossi	2013	1	3	1	1	0	0	1	7
**[132]**	Rubin	2018	1	3	1	1	2	0	1	9
**[133]**	Saczuk	2022	1	3	1	1	2	0	1	9
**[134]**	Selms	2019	1	3	1	1	1	2	2	11
**[135]**	Seraj	2010	1	3	1	1	3	0	2	11
**[136]**	Serra-Negra	2021	1	3	1	1	3	0	2	11
**[137]**	Serra-Negra	2009	1	3	1	1	2	1	0	9
**[138]**	Shalev-Antsel	2023	1	3	1	1	1	1	1	9
**[139]**	Prakash	2022	1	3	1	1	2	0	2	10
**[140]**	Shahbour	2022	1	3	1	1	1	1	2	10
**[141]**	Shokry	2016	1	2	1	1	0	2	2	9
**[142]**	Sierwald	2015	1	2	1	1	2	1	1	9
**[143]**	Silva	2023	1	3	1	1	1	2	1	10
**[144]**	Silva	2016	1	3	1	1	3	1	1	11
**[145]**	Simões-Zenari	2010	1	3	1	1	1	0	1	8
**[146]**	Siqueira	2013	1	3	1	1	1	2	2	11
**[147]**	Siva	2021	1	3	1	1	1	1	1	9
**[148]**	Soares	2017	1	2	1	1	0	2	2	9
**[149]**	Soares	2018	1	3	1	1	3	0	2	11
**[150]**	Sousa	2018	1	3	1	1	2	2	2	12
**[151]**	Soares	2020	1	3	1	1	3	1	0	10
**[152]**	Somay	2020	1	2	1	1	2	1	1	9
**[153]**	Souza	2020	1	3	1	1	2	2	1	11
**[154]**	Strausz	2010	1	3	1	1	1	0	1	8
**[155]**	Suwa	2009	1	3	1	1	1	1	1	9
**[156]**	Tachibana	2016	1	3	1	1	3	0	2	11
**[157]**	Tay	2020	1	3	1	1	3	1	1	11
**[158]**	Phuong	2020	1	3	1	1	3	0	1	10
**[159]**	Tinastepe	2021	1	3	1	1	2	1	2	11
**[160]**	Toyama	2020	1	3	1	1	3	0	1	10
**[161]**	Traebert	2020	1	2	1	1	2	2	2	11
**[162]**	Tsuchiya	2022	1	3	1	1	2	5	2	15
**[163]**	Uca	2015	1	3	1	1	3	0	1	10
**[164]**	Uma	2021	1	3	1	1	3	1	2	12
**[165]**	Unell	2011	1	3	1	1	3	0	2	11
**[166]**	Us	2021	1	3	1	1	1	1	1	9
**[167]**	Selms	2012	1	3	1	1	2	1	2	11
**[168]**	Vieira	2020	1	3	1	1	1	1	1	9
**[169]**	Vieira-Andrade	2014	1	3	1	1	2	2	1	11
**[170]**	Vlăduțu	2022	1	3	1	1	3	2	2	13
**[171]**	Wetselaar	2019	1	3	1	1	2	1	2	11
**[172]**	Wetselaar	2020	1	3	1	1	3	0	1	10

**[173]**	Winocur	2019	1	3	1	1	1	2	1	10
**[174]**	Winocur-Arias	2023	1	3	1	1	3	1	2	12
**[175]**	Yachida	2016	1	2	1	1	2	1	2	10
**[176]**	Yeler	2016	1	3	1	1	0	2	1	9
**[177]**	Yıldırım	2021	1	3	1	1	2	1	2	11
**[178]**	Yoshinaka	2010	1	3	1	1	3	0	2	11
**[179]**	Zani	2019	1	3	1	1	3	1	1	11
**[180]**	Zani	2021	1	3	1	1	1	1	0	8

References

1. Aguiar, S.O.; Prado, I.M.; Silveira, K.S.R.; Abreu, L.G.; Auad, S.M.; Paiva, S.M.; Serra-Negra, J.M.C. Possible Sleep Bruxism, Circadian Preference, and Sleep-Related Characteristics and Behaviors among Dental Students. *CRANIO®* **2019**, *37*, 389–394, doi:10.1080/08869634.2018.1471113.
2. Ahlberg, J.; Lobbezoo, F.; Manfredini, D.; Piirtola, M.; Hublin, C.; Kaprio, J. Self-Reported Sleep Bruxism and Mortality in 1990–2020 in a Nationwide Twin Cohort. *J. Oral Rehabil.* **2024**, *51*, 125–130, doi:10.1111/joor.13441.
3. Ahlberg, K.; Jahkola, A.; Savolainen, A.; Könönen, M.; Partinen, M.; Hublin, C.; Sinisalo, J.; Lindholm, H.; Sarna, S.; Ahlberg, J. Associations of Reported Bruxism with Insomnia and Insufficient Sleep Symptoms among Media Personnel with or without Irregular Shift Work. *Head Face Med.* **2008**, *4*, 4, doi:10.1186/1746-160X-4-4.
4. Alfano, C.A.; Bower, J.L.; Meers, J.M. Polysomnography-Detected Bruxism in Children Is Associated With Somatic Complaints But Not Anxiety. *J. Clin. Sleep Med.* **2018**, *14*, 23–29, doi:10.5664/jcsm.6872.
5. Brandão de Almeida, A.; Rodrigues, R.S.; Simão, C.; de Araújo, R.P.; Figueiredo, J. Prevalence of Sleep Bruxism Reported by Parents/Caregivers in a Portuguese Pediatric Dentistry Service: A Retrospective Study. *Int. J. Environ. Res. Public. Health* **2022**, *19*, 7823, doi:10.3390/ijerph19137823.
6. Almutairi, A.F.; Albesher, N.; Aljohani, M.; Alsinanni, M.; Turkistani, O.; Salam, M. Association of Oral Parafunctional Habits with Anxiety and the Big-Five Personality Traits in the Saudi Adult Population. *Saudi Dent. J.* **2021**, *33*, 90–98, doi:10.1016/j.sdentj.2020.01.003.
7. Alonso, L.S.; Serra-Negra, J.M.; Abreu, L.G.; Martins, I.M.; Tourino, L.F.P.G.; Vale, M.P. Association between Possible Awake Bruxism and Bullying among 8- to 11-Year-Old Children/Adolescents. *Int. J. Paediatr. Dent.* **2022**, *32*, 41–48, doi:10.1111/ipd.12789.
8. Noor Al-Swaje*, A.-A.S., Shatha Al-Khalifah S, Prof. Sana Shafshaks THE PREVALENCE OF BRUXISM AND DENTAL WEAR IN CHILDREN IN RELATION TO SMART DEVICES AND VIDEO GAMES. *INDO Am. J. Pharm. Sci.* **2019**, *o6*, 4560–4565, doi:10.5281/zenodo.2579294.
9. Amaral, C.C.; Fernandez, M. dos S.; Jansen, K.; da Silva, R.A.; Boscato, N.; Goettems, M.L. Daily Screen Time, Sleep Pattern, and Probable Sleep Bruxism in Children: A Cross-Sectional Study. *Oral Dis.* **2023**, *29*, 2888–2894, doi:10.1111/odi.14395.
10. Antunes, L.A.A.; Castilho, T.; Marinho, M.; Fraga, R.S.; Antunes, L.S. Childhood Bruxism: Related Factors and Impact on Oral Health-Related Quality of Life. *Spec. Care Dentist.* **2016**, *36*, 7–12, doi:10.1111/scd.12140.
11. Arısan, V.; Bedeloğlu, E.; Pişkin, B. Prevalence and Predictors of Bruxism in Two University Clinic Patient Populations with Dental Implants: A Cross-Sectional Analysis. *Cranio J. Craniomandib. Pract.* **2022**, 1–12, doi:10.1080/08869634.2022.2071794.
12. Azodo, C.; Ojehanon, P. Bruxism Experience among Undergraduates of a Nigerian University. *Indian J. Multidiscip. Dent.* **2016**, *6*, 14, doi:10.4103/2229-6360.188219.
13. Bach, S. de L.; Moreira, F.P.; Goettems, M.L.; Brancher, L.C.; Oses, J.P.; da Silva, R.A.; Jansen, K. Salivary Cortisol Levels and Biological Rhythm in Schoolchildren with Sleep Bruxism. *Sleep Med.* **2019**, *54*, 48–52, doi:10.1016/j.sleep.2018.09.031.
14. Mercan Başpınar, M.; Mercan, Ç.; Mercan, M.; Arslan Aras, M. Comparison of the Oral Health-Related Quality of Life, Sleep Quality, and Oral Health Literacy in Sleep and Awake Bruxism: Results from Family Medicine Practice. *Int. J. Clin. Pract.* **2023**, *2023*, 1186278, doi:10.1155/2023/1186278.
15. Berger, M.; Szalewski, L.; Szkutnik, J.; Ginszt, M.; Ginszt, A. Different Association between Specific Manifestations of Bruxism and Temporomandibular Disorder Pain. *Neurol. Neurochir. Pol.* **2017**, *51*, 7–11, doi:10.1016/j.pjnns.2016.08.008.
16. Bharti, B.; Malhi, P.; Kashyap, S. Patterns and Problems of Sleep in School Going Children. *Indian Pediatr.* **2006**, *43*, 35–38.
17. Bolsson, G.B.; Knorst, J.K.; Menegazzo, G.R.; Ardenghi, T.M. Impact of Dental Bullying on Bruxism Associated with Poor Sleep Quality among Adolescents. *Braz. Oral Res.* **2023**, *37*, e36, doi:10.1590/1807-3107BOR-2023.vol37.0036.
18. Borie, L.; Langbour, N.; Guehl, D.; Burbaud, P.; Ella, B. Bruxism in Craniocervical Dystonia: A Prospective Study. *Cranio J. Craniomandib. Pract.* **2016**, *34*, 291–295, doi:10.1080/08869634.2015.1120473.
19. Bortoletto, C.C.; Salgueiro, M. da C.C.; Valio, R.; Fragoso, Y.D.; Motta, P. de B.; Motta, L.J.; Kobayashi, F.Y.; Fernandes, K.P.S.; Mesquita-Ferrari, R.A.; Deana, A.; et al. The Relationship between Bruxism, Sleep Quality, and Headaches in Schoolchildren. *J. Phys. Ther. Sci.* **2017**, *29*, 1889–1892, doi:10.1589/jpts.29.1889.

20. Botelho, J.; Machado, V.; Proença, L.; Rua, J.; Martins, L.; Alves, R.; Cavacas, M.A.; Manfredini, D.; Mendes, J.J. Relationship between Self-Reported Bruxism and Periodontal Status: Findings from a Cross-Sectional Study. *J. Periodontol.* **2020**, *91*, 1049–1056, doi:10.1002/JPER.19-0364.
21. Bracci, A.; Djukic, G.; Favero, L.; Salmaso, L.; Guarda-Nardini, L.; Manfredini, D. Frequency of Awake Bruxism Behaviours in the Natural Environment. A 7-Day, Multiple-Point Observation of Real-Time Report in Healthy Young Adults. *J. Oral Rehabil.* **2018**, *45*, 423–429, doi:10.1111/joor.12627.
22. Brancher, L.C.; Cademartori, M.G.; Jansen, K.; da Silva, R.A.; Bach, S.; Reyes, A.; Boscato, N.; Goettems, M.L. Social, Emotional, and Behavioral Problems and Parent-Reported Sleep Bruxism in Schoolchildren. *J. Am. Dent. Assoc.* **2020**, *151*, 327–333, doi:10.1016/j.adaj.2020.01.025.
23. Breda, M.; Belli, A.; Esposito, D.; Di Pilla, A.; Melegari, M.G.; DelRosso, L.; Malorgio, E.; Doria, M.; Ferri, R.; Bruni, O. Sleep Habits and Sleep Disorders in Italian Children and Adolescents: A Cross-Sectional Survey. *J. Clin. Sleep Med. JCSM Off. Publ. Am. Acad. Sleep Med.* **2023**, *19*, 659–672, doi:10.5664/jcsm.10400.
24. Bucci, C.; Amato, M.; Zingone, F.; Caggiano, M.; Iovino, P.; Ciacci, C. Prevalence of Sleep Bruxism in IBD Patients and Its Correlation to Other Dental Disorders and Quality of Life. *Gastroenterol. Res. Pract.* **2018**, *2018*, 7274318, doi:10.1155/2018/7274318.
25. Bucci, R.; Manfredini, D.; Lenci, F.; Simeon, V.; Bracci, A.; Michelotti, A. Comparison between Ecological Momentary Assessment and Questionnaire for Assessing the Frequency of Waking-Time Non-Functional Oral Behaviours. *J. Clin. Med.* **2022**, *11*, 5880, doi:10.3390/jcm11195880.
26. Cai, X.-H.; Xie, Y.-P.; Li, X.-C.; Qu, W.-L.; Li, T.; Wang, H.-X.; Lv, J.-Q.; Wang, L.-X. The Prevalence and Associated Risk Factors of Sleep Disorder-Related Symptoms in Pregnant Women in China. *Sleep Breath. Schlaf Atm.* **2013**, *17*, 951–956, doi:10.1007/s11325-012-0783-2.
27. Câmara-Souza, M.B.; Carvalho, A.G.; Figueredo, O.M.C.; Bracci, A.; Manfredini, D.; Rodrigues Garcia, R.C.M. Awake Bruxism Frequency and Psychosocial Factors in College Preparatory Students. *Cranio J. Craniomandib. Pract.* **2023**, *41*, 178–184, doi:10.1080/08869634.2020.1829289.
28. Carra, M.C.; Huynh, N.; Morton, P.; Rompré, P.H.; Papadakis, A.; Remise, C.; Lavigne, G.J. Prevalence and Risk Factors of Sleep Bruxism and Wake-Time Tooth Clenching in a 7- to 17-Yr-Old Population. *Eur. J. Oral Sci.* **2011**, *119*, 386–394, doi:10.1111/j.1600-0722.2011.00846.x.
29. Cavalcante-Leão, B.L.; Todero, S.R.B.; Ferreira, F.M.; Gavião, M.B.D.; Fraiz, F.C. Profile of Orofacial Dysfunction in Brazilian Children Using the Nordic Orofacial Test-Screening. *Acta Odontol. Scand.* **2017**, *75*, 262–267, doi:10.1080/00016357.2017.1290823.
30. Cavallo, P.; Carpinelli, L.; Savarese, G. Perceived Stress and Bruxism in University Students. *BMC Res. Notes* **2016**, *9*, 514, doi:10.1186/s13104-016-2311-0.
31. Chattrattrai, T.; Blanken, T.F.; Lobbezoo, F.; Su, N.; Aarab, G.; Van Someren, E.J.W. A Network Analysis of Self-Reported Sleep Bruxism in the Netherlands Sleep Registry: Its Associations with Insomnia and Several Demographic, Psychological, and Life-Style Factors. *Sleep Med.* **2022**, *93*, 63–70, doi:10.1016/j.sleep.2022.03.018.
32. Cheifetz, A.T.; Osganian, S.K.; Allred, E.N.; Needleman, H.L. Prevalence of Bruxism and Associated Correlates in Children as Reported by Parents. *J. Dent. Child. Chic. Ill* **2005**, *72*, 67–73.
33. Ciancaglini, R.; Gherlone, E.F.; Radaelli, G. The Relationship of Bruxism with Craniofacial Pain and Symptoms from the Masticatory System in the Adult Population. *J. Oral Rehabil.* **2001**, *28*, 842–848, doi:10.1111/j.1365-2842.2001.00753.x.
34. Clementino, M.A.; Siqueira, M.B.; Serra-Negra, J.M.; Paiva, S.M.; Granville-Garcia, A.F. The Prevalence of Sleep Bruxism and Associated Factors in Children: A Report by Parents. *Eur. Arch. Paediatr. Dent. Off. J. Eur. Acad. Paediatr. Dent.* **2017**, *18*, 399–404, doi:10.1007/s40368-017-0312-x.
35. Colonna, A.; Guarda-Nardini, L.; Ferrari, M.; Manfredini, D. COVID-19 Pandemic and the Psyche, Bruxism, Temporomandibular Disorders Triangle. *CRANIO®* **2021**, *0*, 1–6, doi:10.1080/08869634.2021.1989768.
36. da Costa, S.V.; de Souza, B.K.; Cruvinel, T.; Oliveira, T.M.; Lourenço Neto, N.; Machado, M.A.A.M. Factors Associated with Preschool Children’s Sleep Bruxism. *Cranio J. Craniomandib. Pract.* **2024**, *42*, 48–54, doi:10.1080/08869634.2021.1903663.
37. Costa, F.D.S.; Fernandez, M.D.S.; Silva-Junior, I.F. da; Karam, S.A.; Chisini, L.A.; Goettems, M.L. Association Involving Possible Sleep Bruxism, Stress, and Depressive Symptoms in Brazilian University Students: A Cross-Sectional Study. *Sleep Sci. Sao Paulo Braz.* **2023**, *16*, e317–e322, doi:10.1055/s-0043-1772808.
38. Nogueira Coutinho MPH, E.; Pereira Rodrigues dos Santos MPH, K.; Henrique Barros Ferreira MPH, E.; Grailea Silva Pinto BHS, R.; de Oliveira Sanchez DPH, M. Association between Self-Reported Sleep Bruxism and Temporomandibular Disorder in Undergraduate Students from Brazil. *CRANIO®* **2020**, *38*, 91–98, doi:10.1080/08869634.2018.1495874.
39. Dantas-Neta, N.B.; Laurentino, J.B.; Souza, C.H. de C. e; Nunes-Dos-Santos, D.L.; Mendes, R.F.; Prado-Júnior, R.R. Prevalence and Potential Factors Associated with Probable Sleep or Awake Bruxism and Dentin Hypersensitivity in Undergraduate Students. *Rev. Odontol. UNESP* **2014**, *43*, 245–251, doi:10.1590/rou.2014.040.
40. Delgado-Delgado, R.; Iriarte-Álvarez, N.; Valera-Calero, J.A.; Centenera-Centenera, M.B.; Garnacho-Garnacho, V.E.; Gallego-Sendarrubias, G.M. Association between Temporomandibular Disorders with Clinical and Sociodemographic Features: An Observational Study. *Int. J. Clin. Pract.* **2021**, *75*, e13961, doi:10.1111/ijcp.13961.
41. Demir, A.; Uysal, T.; Guray, E.; Basciftci, F.A. The Relationship between Bruxism and Occlusal Factors among Seven- to 19-Year-Old Turkish Children. *Angle Orthod.* **2004**, *74*, 672–676, doi:10.1043/0003-3219(2004)074<0672:TRBBAO>2.0.CO;2.
42. Dias, R.; Vaz, R.; Rodrigues, M.J.; Serra-Negra, J.M.; Bracci, A.; Manfredini, D. Utility of Smartphone-Based Real-Time Report (Ecological Momentary Assessment) in the Assessment and Monitoring of Awake Bruxism: A Multiple-

Week Interval Study in a Portuguese Population of University Students. *J. Oral Rehabil.* **2021**, *48*, 1307–1313, doi:10.1111/joor.13259.
43. Diéguez-Pérez, M.; Ticona-Flores, J.M.; Prieto-Regueiro, B. Prevalence of Possible Sleep Bruxism and Its Association with Social and Orofacial Factors in Preschool Population. *Healthcare* **2023**, *11*, 1450, doi:10.3390/healthcare11101450.
44. Drumond, C.L.; Ramos-Jorge, J.; Vieira-Andrade, R.G.; Paiva, S.M.; Serra-Negra, J.M.C.; Ramos-Jorge, M.L. Prevalence of Probable Sleep Bruxism and Associated Factors in Brazilian Schoolchildren. *Int. J. Paediatr. Dent.* **2018**, doi:10.1111/ipd.12443.
45. Duarte, J.; Souza, J.F. de; Cavalcante-Leão, B.; Todero, S.R.B.; Ferreira, F.M.; Fraiz, F.C. Association of Possible Sleep Bruxism with Daytime Oral Habits and Sleep Behavior in Schoolchildren. *Cranio J. Craniomandib. Pract.* **2021**, *39*, 372–378, doi:10.1080/08869634.2019.1661113.
46. Ekman, A.; Rousu, J.; Näpänkangas, R.; Kuoppala, R.; Raustia, A.; Sipilä, K. Association of Self-Reported Bruxism with Temporomandibular Disorders – Northern Finland Birth Cohort (NFBC) 1966 Study. *CRANIO®* **2023**, *41*, 212–217, doi:10.1080/08869634.2020.1853306.
47. Eli, I.; Zigler-Garburg, A.; Winocur, E.; Friedman-Rubin, P.; Shalev-Antsel, T.; Levartovsky, S.; Emodi-Perlman, A. Temporomandibular Disorders and Bruxism among Sex Workers-A Cross Sectional Study. *J. Clin. Med.* **2022**, *11*, 6622, doi:10.3390/jcm11226622.
48. Emmanuelli, B.; Araujo, G. de; Knorst, J.K.; Tagliari, C.V. da C.; Baldissera, B.S.; Tuchtenhagen, S. Social Capital and Possible Bruxism during the COVID-19 Pandemic among Brazilian Undergraduates. *Braz. Oral Res.* **2023**, *37*, e108, doi:10.1590/1807-3107bor-2023.vol37.0108.
49. Emodi-Perlman, A.; Eli, I.; Smardz, J.; Uziel, N.; Wieckiewicz, G.; Gilon, E.; Grychowska, N.; Wieckiewicz, M. Temporomandibular Disorders and Bruxism Outbreak as a Possible Factor of Orofacial Pain Worsening during the COVID-19 Pandemic-Concomitant Research in Two Countries. *J. Clin. Med.* **2020**, *9*, 3250, doi:10.3390/jcm9103250.
50. Emodi Perlman, A.; Lobbezoo, F.; Zar, A.; Friedman Rubin, P.; van Selms, M.K.A.; Winocur, E. Self-Reported Bruxism and Associated Factors in Israeli Adolescents. *J. Oral Rehabil.* **2016**, *43*, 443–450, doi:10.1111/joor.12391.
51. Fan, W.-Y.; Tiang, N.; Broadbent, J.M.; Thomson, W.M. Occurrence, Associations, and Impacts of Nocturnal Parafunction, Daytime Parafunction, and Temporomandibular Symptoms in 38-Year-Old Individuals. *J. Oral Facial Pain Headache* **2019**, *33*, 254–259, doi:10.11607/ofph.2221.
52. Farsi, N.M.A. Symptoms and Signs of Temporomandibular Disorders and Oral Parafunctions among Saudi Children. *J. Oral Rehabil.* **2003**, *30*, 1200–1208, doi:10.1111/j.1365-2842.2003.01187.x.
53. Ferreira, N.M.R.; dos Santos, J.F.F.; dos Santos, M.B.F.; Marchini, L. Sleep Bruxism Associated with Obstructive Sleep Apnea Syndrome in Children. *CRANIO®* **2015**, *33*, 251–255, doi:10.1179/2151090314Y.0000000025.
54. Feteih, R.M. Signs and Symptoms of Temporomandibular Disorders and Oral Parafunctions in Urban Saudi Arabian Adolescents: A Research Report. *Head Face Med.* **2006**, *2*, 25, doi:10.1186/1746-160X-2-25.
55. Medina Flores, D.; Barragán Nuñez, M.I.; Müller de Quevedo, H.; Bonjardim, L.R.; Rodrigues Conti, P.C. Real Time Evaluation of Awake Bruxism Behaviors in Young Asymptomatic Students and Its Impact on the Masticatory Muscles. *J. Prosthet. Dent.* **2023**, S0022-3913(23)00174-9, doi:10.1016/j.prosdent.2023.03.009.
56. Flueraşu, M.I.; Bocşan, I.C.; Ţig, I.-A.; Iacob, S.M.; Popa, D.; Buduru, S. The Epidemiology of Bruxism in Relation to Psychological Factors. *Int. J. Environ. Res. Public. Health* **2022**, *19*, 691, doi:10.3390/ijerph19020691.
57. Fonseca, C.M.E.; dos Santos, M.B.F.; Consani, R.L.X.; dos Santos, J.F.F.; Marchini, L. Incidence of Sleep Bruxism among Children in Itanhandu, Brazil. *Sleep Breath.* **2011**, *15*, 215–220, doi:10.1007/s11325-010-0427-3.
58. Fulgencio, L.B.; Corrêa-Faria, P.; Lage, C.F.; Paiva, S.M.; Pordeus, I.A.; Serra-Negra, J.M. Diagnosis of Sleep Bruxism Can Assist in the Detection of Cases of Verbal School Bullying and Measure the Life Satisfaction of Adolescents. *Int. J. Paediatr. Dent.* **2017**, *27*, 293–301, doi:10.1111/ipd.12264.
59. Gao, Y.; Xu, P.; Aizetiguli, M.; Surong, S.; Zhu, Z.; Zhang, J. Prevalence and Influencing Factors of Sleep Disorders among Preschool Children in Urumqi City: A Cross-Sectional Survey. *Ital. J. Pediatr.* **2023**, *49*, 68, doi:10.1186/s13052-023-01477-w.
60. Garde, J.B.; Suryavanshi, R.K.; Jawale, B.A.; Deshmukh, V.; Dadhe, D.P.; Suryavanshi, M.K. An Epidemiological Study to Know the Prevalence of Deleterious Oral Habits among 6 to 12 Year Old Children. *J. Int. Oral Health JIOH* **2014**, *6*, 39–43.
61. Ghafournia, M.; Hajenourozali Tehrani, M. Relationship between Bruxism and Malocclusion among Preschool Children in Isfahan. *J. Dent. Res. Dent. Clin. Dent. Prospects* **2012**, *6*, 138–142, doi:10.5681/joddd.2012.028.
62. Ghalebandi, M.; Salehi, M.; Rasoulain, M.; Shooshtari, M.H.; Naserbakht, M.; Salarifar, M.H. Prevalence of Parasomnia in School Aged Children in Tehran. *Iran. J. Psychiatry* **2011**, *6*, 75–79.
63. Goettems, M.L.; Poletto-Neto, V.; Shqair, A.Q.; Pinheiro, R.T.; Demarco, F.F. Influence of Maternal Psychological Traits on Sleep Bruxism in Children. *Int. J. Paediatr. Dent.* **2017**, *27*, 469–475, doi:10.1111/ipd.12285.
64. Gomes, M.C.; Neves, É.T.; Perazzo, M.F.; Souza, E.G.C. de; Serra-Negra, J.M.; Paiva, S.M.; Granville-Garcia, A.F. Evaluation of the Association of Bruxism, Psychosocial and Sociodemographic Factors in Preschoolers. *Braz. Oral Res.* **2018**, *32*, e009, doi:10.1590/1807-3107bor-2018.vol32.0009.
65. Goulart, A.C.; Arap, A.M.; Bufarah, H.B.; Bismarchi, D.; Rienzo, M.; Syllos, D.H.; Wang, Y.-P. Anxiety, Depression, and Anger in Bruxism: A Cross-Sectional Study among Adult Attendees of a Preventive Center. *Psychiatry Res.* **2021**, *299*, 113844, doi:10.1016/j.psychres.2021.113844.
66. Hermesh, H.; Schapir, L.; Marom, S.; Skopski, R.; Barnea, E.; Weizman, A.; Winocur, E. Bruxism and Oral Parafunctional Hyperactivity in Social Phobia Outpatients. *J. Oral Rehabil.* **2015**, *42*, 90–97, doi:10.1111/joor.12235.
67. Hilgenberg-Sydney, P.B.; Lorenzon, A.L.; Pimentel, G.; Petterle, R.R.; Bonotto, D. Probable Awake Bruxism - Prevalence and Associated Factors: A Cross-Sectional Study. *Dent. Press J. Orthod.* **2022**, *27*, e2220298, doi:10.1590/2177-6709.27.4.e2220298.oar.

68. de Holanda, T.A.; Marmitt, L.P.; Cesar, J.A.; Svensson, P.; Boscato, N. Sleep Bruxism in Puerperal Women: Data from a Population-Based Survey. *Matern. Child Health J.* **2023**, *27*, 262–271, doi:10.1007/s10995-022-03576-2.
69. Azario de Holanda, T.; Castagno, C.D.; Barbon, F.J.; Mota Freitas, M.P.; Goettems, M.L.; Boscato, N. Influence of Respiratory Allergy and Restless Sleep on Definite Sleep Bruxism: A Cross-Sectional Clinical Study. *Sleep Med.* **2020**, *70*, 43–49, doi:10.1016/j.sleep.2020.02.010.
70. Huhtela, O.S.; Näpänkangas, R.; Suominen, A.L.; Karppinen, J.; Kunttu, K.; Sipilä, K. Association of Psychological Distress and Widespread Pain with Sympatoms of Temporomandibular Disorders and Self-Reported Bruxism in Students. *Clin. Exp. Dent. Res.* **2021**, *7*, 1154–1166, doi:10.1002/cre2.472.
71. Insana, S.P.; Gozal, D.; McNeil, D.W.; Montgomery-Downs, H.E. Community Based Study of Sleep Bruxism during Early Childhood. *Sleep Med.* **2013**, *14*, 183–188, doi:10.1016/j.sleep.2012.09.027.
72. Itani, O.; Kaneita, Y.; Ikeda, M.; Kondo, S.; Yamamoto, R.; Osaki, Y.; Kanda, H.; Suzuki, K.; Higuchi, S.; Ohida, T. Disorders of Arousal and Sleep-Related Bruxism among Japanese Adolescents: A Nationwide Representative Survey. *Sleep Med.* **2013**, *14*, 532–541, doi:10.1016/j.sleep.2013.03.005.
73. Johansson, A.; Unell, L.; Carlsson, G.E.; Söderfeldt, B.; Halling, A.; Widar, F. Associations between Social and General Health Factors and Symptoms Related to Temporomandibular Disorders and Bruxism in a Population of 50-year-old Subjects. *Acta Odontol. Scand.* **2004**, doi:10.1080/00016350410001649.
74. Jokubauskas, L.; Baltrušaitytė, A.; Pileičikienė, G.; Žekonis, G. Interrelationships between Distinct Circadian Manifestations of Possible Bruxism, Perceived Stress, Chronotype and Social Jetlag in a Population of Undergraduate Students. *Chronobiol. Int.* **2019**, *36*, 1558–1569, doi:10.1080/07420528.2019.1660356.
75. Juliatte, T. de P.R.; Costa, P.D.; Canaan, J.D.R.; Fonseca, D.C.; Serra-Negra, J.M.; Andrade, E.F.; Castelo, P.M.; Pereira, L.J. Circadian Preference and Its Relationship with Possible Sleep and Awake Bruxism in Adults Assisted by the Public Health System. *Chronobiol. Int.* **2022**, *39*, 68–76, doi:10.1080/07420528.2021.1973487.
76. Junqueira, T.H.; Nahás-Scocate, A.C.R.; Valle-Corotti, K.M. do; Conti, A.C. de C.F.; Trevisan, S. Association of Infantile Bruxism and the Terminal Relationships of the Primary Second Molars. *Braz. Oral Res.* **2013**, *27*, 42–47, doi:10.1590/s1806-83242013000100008.
77. Kirarslan Karagoz, O.; Yildirim, B.; Tekeli Simsek, A.; Koca, C.G.; Igneci, M. Possible Sleep and Awake Bruxism, Chronotype Profile and TMD Symptoms among Turkish Dental Students. *Chronobiol. Int.* **2021**, *38*, 1367–1374, doi:10.1080/07420528.2021.1931279.
78. Kataoka, K.; Ekuni, D.; Mizutani, S.; Tomofuji, T.; Azuma, T.; Yamane, M.; Kawabata, Y.; Iwasaki, Y.; Morita, M. Association Between Self-Reported Bruxism and Malocclusion in University Students: A Cross-Sectional Study. *J. Epidemiol.* **2015**, *25*, 423–430, doi:10.2188/jea.JE20140180.
79. Kato, T.; Velly, A.M.; Nakane, T.; Masuda, Y.; Maki, S. Age Is Associated with Self-Reported Sleep Bruxism, Independently of Tooth Loss. *Sleep Breath.* **2012**, *16*, 1159–1165, doi:10.1007/s11325-011-0625-7.
80. Kaya, M.; Koroglu, A.; Sahin, O. The Relationship of Psychological Status and Sociodemographic Factors with Bruxism among Undergraduate Dental Students: A National Survey. *Niger. J. Clin. Pract.* **2022**, *25*, 944–950, doi:10.4103/njcp.njcp_1980_21.
81. Khatami, R.; Zutter, D.; Siegel, A.; Mathis, J.; Donati, F.; Bassetti, C.L. Sleep-Wake Habits and Disorders in a Series of 100 Adult Epilepsy Patients--a Prospective Study. *Seizure* **2006**, *15*, 299–306, doi:10.1016/j.seizure.2006.02.018.
82. Khayat, N.; Winocur, E.; Emodi Perelman, A.; Friedman-Rubin, P.; Gafni, Y.; Shpack, N. The Prevalence of Posterior Crossbite, Deep Bite, and Sleep or Awake Bruxism in Temporomandibular Disorder (TMD) Patients Compared to a Non-TMD Population: A Retrospective Study. *Cranio J. Craniomandib. Pract.* **2021**, *39*, 398–404, doi:10.1080/08869634.2019.1650217.
83. Khoury, S.; Carra, M.C.; Huynh, N.; Montplaisir, J.; Lavigne, G.J. Sleep Bruxism-Tooth Grinding Prevalence, Characteristics and Familial Aggregation: A Large Cross-Sectional Survey and Polysomnographic Validation. *Sleep* **2016**, *39*, 2049–2056, doi:10.5665/sleep.6242.
84. Kilincaslan, A.; Yilmaz, K.; Oflaz, S.B.; Aydin, N. Epidemiological Study of Self-Reported Sleep Problems in Turkish High School Adolescents. *Pediatr. Int. Off. J. Jpn. Pediatr. Soc.* **2014**, *56*, 594–600, doi:10.1111/ped.12287.
85. Kim, D.S.; Lee, C.L.; Ahn, Y.M. Sleep Problems in Children and Adolescents at Pediatric Clinics. *Korean J. Pediatr.* **2017**, *60*, 158–165, doi:10.3345/kjp.2017.60.5.158.
86. Kolak, V.; Pavlovic, M.; Aleksic, E.; Biocanin, V.; Gajic, M.; Nikitovic, A.; Lalovic, M.; Melih, I.; Pesic, D. Probable Bruxism and Psychological Issues among Dental Students in Serbia during the COVID-19 Pandemic. *Int. J. Environ. Res. Public. Health* **2022**, *19*, 7729, doi:10.3390/ijerph19137729.
87. Lam, M.H.B.; Zhang, J.; Li, A.M.; Wing, Y.K. A Community Study of Sleep Bruxism in Hong Kong Children: Association with Comorbid Sleep Disorders and Neurobehavioral Consequences. *Sleep Med.* **2011**, *12*, 641–645, doi:10.1016/j.sleep.2010.11.013.
88. Leal, T.R.; de Lima, L.C.M.; Perazzo, M.F.; Neves, É.T.B.; Paiva, S.M.; Serra-Negra, J.M.C.; Ferreira, F.M.; Granville-Garcia, A.F. Influence of the Practice of Sports, Sleep Disorders, and Habits on Probable Sleep Bruxism in Children with Mixed Dentition. *Oral Dis.* **2023**, *29*, 211–219, doi:10.1111/odi.13917.
89. Le, A.; Khoo, E.; Palamar, J.J. Associations between Oral Health and Cannabis Use among Adolescents and Young Adults: Implications for Orthodontists. *Int. J. Environ. Res. Public. Health* **2022**, *19*, 15261, doi:10.3390/ijerph192215261.
90. Levartovsky, S.; Msarwa, S.; Reiter, S.; Eli, I.; Winocur, E.; Sarig, R. The Association between Emotional Stress, Sleep, and Awake Bruxism among Dental Students: A Sex Comparison. *J. Clin. Med.* **2021**, *11*, 10, doi:10.3390/jcm11010010.
91. Lima, L.C.M. de; Leal, T.R.; AraúJo, L.J.S. de; Sousa, M.L.C.; Silva, S.E. da; Serra-Negra, J.M.C.; Ferreira, F. de M.; Paiva, S.M.; Granville-Garcia, A.F. Impact of the COVID-19 Pandemic on Sleep Quality and Sleep Bruxism in Children Eight to Ten Years of Age. *Braz. Oral Res.* **2022**, *36*, e046, doi:10.1590/1807-3107bor-2022.vol36.0046.

92. Liu, X.; Ma, Y.; Wang, Y.; Jiang, Q.; Rao, X.; Lu, X.; Teng, H. Brief Report: An Epidemiologic Survey of the Prevalence of Sleep Disorders among Children 2 to 12 Years Old in Beijing, China. *Pediatrics* **2005**, *115*, 266–268, doi:10.1542/peds.2004-0815I.
93. Macfarlane, T.V.; Blinkhorn, A.S.; Davies, R.M.; Worthington, H.V. Association between Local Mechanical Factors and Orofacial Pain: Survey in the Community. *J. Dent.* **2003**, *31*, 535–542, doi:10.1016/S0300-5712(03)00108-8.
94. Maluly, M.; Dal Fabbro, C.; Andersen, M.L.; Herrero Babiloni, A.; Lavigne, G.J.; Tufik, S. Sleep Bruxism and Its Associations with Insomnia and OSA in the General Population of Sao Paulo. *Sleep Med.* **2020**, *75*, 141–148, doi:10.1016/j.sleep.2020.06.016.
95. Manfredini, D.; Lobbezoo, F.; Giancristofaro, R.A.; Restrepo, C. Association between Proxy-Reported Sleep Bruxism and Quality of Life Aspects in Colombian Children of Different Social Layers. *Clin. Oral Investig.* **2017**, *21*, 1351–1358, doi:10.1007/s00784-016-1901-5.
96. Manfredini, D.; Winocur, E.; Guarda-Nardini, L.; Lobbezoo, F. Self-Reported Bruxism and Temporomandibular Disorders: Findings from Two Specialised Centres. *J. Oral Rehabil.* **2012**, *39*, 319–325, doi:10.1111/j.1365-2842.2011.02281.x.
97. Martynowicz, H.; Wieckiewicz, M.; Poreba, R.; Wojakowska, A.; Smardz, J.; Januszewska, L.; Markiewicz-Gorka, I.; Mazur, G.; Pawlas, K.; Gac, P. The Relationship between Sleep Bruxism Intensity and Renalase Concentration—An Enzyme Involved in Hypertension Development. *J. Clin. Med.* **2020**, *9*, 16, doi:10.3390/jcm9010016.
98. Massignan, C.; de Alencar, N.A.; Soares, J.P.; Santana, C.M.; Serra-Negra, J.; Bolan, M.; Cardoso, M. Poor Sleep Quality and Prevalence of Probable Sleep Bruxism in Primary and Mixed Dentitions: A Cross-Sectional Study. *Sleep Breath.* **2019**, *23*, 935–941, doi:10.1007/s11325-018-1771-y.
99. Melis, M.; Abou-Atme, Y.S. Prevalence of Bruxism Awareness in a Sardinian Population. *CRANIO®* **2003**, *21*, 144–151, doi:10.1080/08869634.2003.11746243.
100. Melo, P.E.D.; Pontes, J.R.D.S. Deleterious Oral Habits in a Group of Children from a Public School in Sao Paulo City. *Rev. CEFAC* **2014**, *16*, 1945–1952, doi:10.1590/1982-0216201418213.
101. Miamoto, C.B.; Pereira, L.J.; Ramos-Jorge, M.L.; Marques, L.S. Prevalence and Predictive Factors of Sleep Bruxism in Children with and without Cognitive Impairment. *Braz. Oral Res.* **2011**, *25*, 439–445, doi:10.1590/s1806-83242011000500011.
102. Montero, J.; Gómez-Polo, C. Personality Traits and Dental Anxiety in Self-Reported Bruxism. A Cross-Sectional Study. *J. Dent.* **2017**, *65*, 45–50, doi:10.1016/j.jdent.2017.07.002.
103. Nagamatsu-Sakaguchi, C.; Minakuchi, H.; Clark, G.T.; Kuboki, T. Relationship between the Frequency of Sleep Bruxism and the Prevalence of Signs and Symptoms of Temporomandibular Disorders in an Adolescent Population. *Int. J. Prosthodont.* **2008**, *21*, 292–298.
104. Nahás-Scocate, A.C.R.; Coelho, F.V.; Almeida, V.C. de Bruxism in Children and Transverse Plane of Occlusion: Is There a Relationship or Not? *Dent. Press J. Orthod.* **2014**, *19*, 67–73, doi:10.1590/2176-9451.19.5.067-073.oar.
105. Nakata, A.; Takahashi, M.; Ikeda, T.; Hojou, M.; Araki, S. Perceived Psychosocial Job Stress and Sleep Bruxism among Male and Female Workers. *Community Dent. Oral Epidemiol.* **2008**, *36*, 201–209, doi:10.1111/j.1600-0528.2007.00388.x.
106. Nazzal, H.; Baccar, M.; Ziad, T.; Al-Musfir, T.; Al Emadi, B.; Matoug-Elwerfelli, M.; Narasimhan, S.; Khan, Y.; Reagu, S. Prevalence of Anxiety, Sleep Bruxism and Temporomandibular Disorders during COVID-19 in Qatari Children and Adolescents: A Cross-Sectional Study. *Eur. Arch. Paediatr. Dent. Off. J. Eur. Acad. Paediatr. Dent.* **2023**, *24*, 787–795, doi:10.1007/s40368-023-00847-6.
107. Nekora-Azak, A.; Yengin, E.; Evlioglu, G.; Ceyhan, A.; Ocak, O.; Issever, H. Prevalence of Bruxism Awareness in Istanbul, Turkey. *Cranio J. Craniomandib. Pract.* **2010**, *28*, 122–127, doi:10.1179/crn.2010.017.
108. Ng, E.P.; Ng, D.K.; Chan, C.H. Sleep Duration, Wake/Sleep Symptoms, and Academic Performance in Hong Kong Secondary School Children. *Sleep Breath.* **2009**, *13*, 357–367, doi:10.1007/s11325-009-0255-5.
109. Nykänen, L.; Manfredini, D.; Lobbezoo, F.; Kämppi, A.; Bracci, A.; Ahlberg, J. Assessment of Awake Bruxism by a Novel Bruxism Screener and Ecological Momentary Assessment among Patients with Masticatory Muscle Myalgia and Healthy Controls. *J. Oral Rehabil.* **2024**, *51*, 162–169, doi:10.1111/joor.13462.
110. Okawara, A.; Matsuyama, Y.; Yoshizawa Araki, M.; Unnai Yasuda, Y.; Ogawa, T.; Tumurkhuu, T.; Ganburged, G.; Bazar, A.; Fujiwara, T.; Moriyama, K. Association between Child Abuse and Poor Oral Habits in Mongolian Adolescents. *Int. J. Environ. Res. Public. Health* **2022**, *19*, 10667, doi:10.3390/ijerph191710667.
111. Osses-Anguita, Á.E.; Sánchez-Sánchez, T.; Soto-Goñi, X.A.; García-González, M.; Alén Fariñas, F.; Cid-Verdejo, R.; Sánchez Romero, E.A.; Jiménez-Ortega, L. Awake and Sleep Bruxism Prevalence and Their Associated Psychological Factors in First-Year University Students: A Pre-Mid-Post COVID-19 Pandemic Comparison. *Int. J. Environ. Res. Public. Health* **2023**, *20*, 2452, doi:10.3390/ijerph20032452.
112. Panek, H.; Nawrot, P.; Mazan, M.; Bielicka, B.; Sumisławska, M.; Pomianowski, R. Coincidence and Awareness of Oral Parafunctions in College Students. *Community Dent. Health* **2012**, 74–77, doi:10.1922/CDH_2684Panek04.
113. Peixoto, K.O.; Resende, C.M.B.M. de; Almeida, E.O. de; Almeida-Leite, C.M.; Conti, P.C.R.; Barbosa, G.A.S.; Barbosa, J.S. Association of Sleep Quality and Psychological Aspects with Reports of Bruxism and TMD in Brazilian Dentists during the COVID-19 Pandemic. *J. Appl. Oral Sci. Rev. FOB* **2021**, *29*, e20201089, doi:10.1590/1678-7757-2020-1089.
114. Pereira, N.C.; Oltramari, P.V.P.; Conti, P.C.R.; Bonjardim, L.R.; de Almeida-Pedrin, R.R.; Fernandes, T.M.F.; de Almeida, M.R.; Conti, A.C.C.F. Frequency of Awake Bruxism Behaviour in Orthodontic Patients: Randomised Clinical Trial: Awake Bruxism Behaviour in Orthodontic Patients. *J. Oral Rehabil.* **2021**, *48*, 422–429, doi:10.1111/joor.13130.
115. Emodi Perlman, A.; Lobbezoo, F.; Zar, A.; Friedman Rubin, P.; van Selms, M.K.A.; Winocur, E. Self-Reported Bruxism and Associated Factors in Israeli Adolescents. *J. Oral Rehabil.* **2016**, *43*, 443–450, doi:10.1111/joor.12391.

116. González-Aragón Pineda, Á.E.; García Pérez, A.; Rosales-Ibáñez, R.; Stein-Gemora, E. Relationship between the Normative Need for Orthodontic Treatment and Oral Health in Mexican Adolescents Aged 13–15 Years Old. *Int. J. Environ. Res. Public. Health* **2020**, *17*, 8107, doi:10.3390/ijerph17218107.
117. Pontes, L. da S.; Prietsch, S.O.M. Sleep bruxism: population based study in people with 18 years or more in the city of Rio Grande, Brazil. *Rev. Bras. Epidemiol. Braz. J. Epidemiol.* **2019**, *22*, e190038, doi:10.1590/1980-549720190038.
118. Prado, I.M.; Abreu, L.G.; Silveira, K.S.; Auad, S.M.; Paiva, S.M.; Manfredini, D.; Serra, -Negra Júnia Maria Study of Associated Factors With Probable Sleep Bruxism Among Adolescents. *J. Clin. Sleep Med. 14*, 1369–1376, doi:10.5664/jcsm.7276.
119. Prado, I.M.; Abreu, L.G.; Pordeus, I.A.; Amin, M.; Paiva, S.M.; Serra-Negra, J.M. Diagnosis and Prevalence of Probable Awake and Sleep Bruxism in Adolescents: An Exploratory Analysis. *Braz. Dent. J.* **2023**, *34*, 9–24, doi:10.1590/0103-6440202305202.
120. Prado, I.M.; Paiva, S.M.; Fonseca-Gonçalves, A.; Maia, L.C.; Tavares-Silva, C.; Fraiz, F.C.; Ferreira, F.M.; Duarte, J.; Granville-Garcia, A.F.; Costa, E.M.M.B.; et al. Knowledge of Parents/Caregivers about the Sleep Bruxism of Their Children from All Five Brazilian Regions: A Multicenter Study. *Int. J. Paediatr. Dent.* **2019**, *29*, 507–523, doi:10.1111/ipd.12486.
121. Quadri, M.F.A.; Mahnashi, A.; Al Almutahhir, A.; Tubayqi, H.; Hakami, A.; Arishi, M.; Alamir, A. Association of Awake Bruxism with Khat, Coffee, Tobacco, and Stress among Jazan University Students. *Int. J. Dent.* **2015**, *2015*, 842096, doi:10.1155/2015/842096.
122. Ramos, P.F.C.; de Lima, M. de D.M.; de Moura, M.S.; Bendo, C.B.; Moura, L. de F.A. de D.; Lima, C.C.B. Breathing Problems, Being an Only Child and Having Parents with Possible Sleep Bruxism Are Associated with Probable Sleep Bruxism in Preschoolers: A Population-Based Study. *Sleep Breath.* **2021**, *25*, 1677–1684, doi:10.1007/s11325-020-02281-0.
123. Alouda, R.; Alshehri, M.; Alnaghmoosh, S.; Shafique, M.; Al-Khudhairy, M.W. Mother's Work Status on Children's Bruxism in a Subset of Saudi Population. *J. Int. Soc. Prev. Community Dent.* **2017**, *7*, S170–S178, doi:10.4103/jispcd.JISPCD_384_17.
124. Rao, S.K.; Bhat, M.; David, J. Work, Stress, and Diurnal Bruxism: A Pilot Study among Information Technology Professionals in Bangalore City, India. *Int. J. Dent.* **2011**, *2011*, 650489, doi:10.1155/2011/650489.
125. Raphael, K.G.; Janal, M.N.; Sirois, D.A.; Dubrovsky, B.; Klausner, J.J.; Krieger, A.C.; Lavigne, G.J. Validity of Self-Reported Sleep Bruxism among Myofascial Temporomandibular Disorder Patients and Controls. *J. Oral Rehabil.* **2015**, *42*, 751–758, doi:10.1111/joor.12310.
126. Rauch, A.; Nitschke, I.; Hahnel, S.; Weber, S.; Zenthöfer, A.; Schierz, O. Prevalence of Temporomandibular Disorders and Bruxism in Seniors. *J. Oral Rehabil.* **2023**, *50*, 531–536, doi:10.1111/joor.13450.
127. Renner, A.C.; da Silva, A.A.M.; Rodriguez, J.D.M.; Simões, V.M.F.; Barbieri, M.A.; Bettiol, H.; Thomaz, E.B.A.F.; da Conceição Saraiva, M. Are Mental Health Problems and Depression Associated with Bruxism in Children? *Community Dent. Oral Epidemiol.* **2012**, *40*, 277–287, doi:10.1111/j.1600-0528.2011.00644.x.
128. Restrepo, C.; Manfredini, D.; Castrillon, E.; Svensson, P.; Santamaria, A.; Alvarez, C.; Manrique, R.; Lobbezoo, F. Diagnostic Accuracy of the Use of Parental-Reported Sleep Bruxism in a Polysomnographic Study in Children. *Int. J. Paediatr. Dent.* **2017**, *27*, 318–325, doi:10.1111/ipd.12262.
129. Ribeiro, M.B.; Manfredini, D.; Tavares-Silva, C.; Costa, L.; Luiz, R.R.; Paiva, S.; Serra-Negra, J.M.; Fonseca-Gonçalves, A.; Maia, L.C. Association of Possible Sleep Bruxism in Children with Different Chronotype Profiles and Sleep Characteristics. *Chronobiol. Int.* **2018**, *35*, 633–642, doi:10.1080/07420528.2018.1424176.
130. Rintakoski, K.; Hublin, C.; Lobbezoo, F.; Rose, R.J.; Kaprio, J. Genetic Factors Account for Half of the Phenotypic Variance in Liability to Sleep-Related Bruxism in Young Adults: A Nationwide Finnish Twin Cohort Study. *Twin Res. Hum. Genet.* **2012**, *15*, 714–719, doi:10.1017/thg.2012.54.
131. Rossi, D.; Manfredini, D. Family and School Environmental Predictors of Sleep Bruxism in Children. *J. Orofac. Pain* **2013**, *27*, 135–141, doi:10.11607/jop.1057.
132. Friedman Rubin, P.; Erez, A.; Peretz, B.; Birenboim-Wilensky, R.; Winocur, E. Prevalence of Bruxism and Temporomandibular Disorders among Orphans in Southeast Uganda: A Gender and Age Comparison. *CRANIO®* **2018**, *36*, 243–249, doi:10.1080/08869634.2017.1331784.
133. Saczuk, K.; Lapinska, B.; Wawrzynkiewicz, A.; Witkowska, A.; Arbildo-Vega, H.I.; Domarecka, M.; Lukomska-Szymanska, M. Temporomandibular Disorders, Bruxism, Perceived Stress, and Coping Strategies among Medical University Students in Times of Social Isolation during Outbreak of COVID-19 Pandemic. *Healthc. Basel Switz.* **2022**, *10*, 740, doi:10.3390/healthcare10040740.
134. van Selms, M.K.A.; Marpaung, C.; Pogosian, A.; Lobbezoo, F. Geographical Variation of Parental-Reported Sleep Bruxism among Children: Comparison between the Netherlands, Armenia and Indonesia. *Int. Dent. J.* **2019**, *69*, 237–243, doi:10.1111/idj.12450.
135. Seraj, B.; Shahrabi, M.; Ghadimi, S.; Ahmadi, R.; Nikfarjam, J.; Zayeri, F.; Taghi, F.P.; Zare, H. The Prevalence of Bruxism and Correlated Factors in Children Referred to Dental Schools of Tehran, Based on Parent's Report. *Iran. J. Pediatr.* **2010**, *20*, 174–180.
136. Serra-Negra, J.M.; Dias, R.B.; Rodrigues, M.J.; Aguiar, S.O.; Auad, S.M.; Pordeus, I.A.; Lombardo, L.; Manfredini, D. Self-Reported Awake Bruxism and Chronotype Profile: A Multicenter Study on Brazilian, Portuguese and Italian Dental Students. *CRANIO®* **2021**, *39*, 113–118, doi:10.1080/08869634.2019.1587854.
137. Serra-Negra, J.M.; Ramos-Jorge, M.L.; Flores-Mendoza, C.E.; Paiva, S.M.; Pordeus, I.A. Influence of Psychosocial Factors on the Development of Sleep Bruxism among Children. *Int. J. Paediatr. Dent.* **2009**, *19*, 309–317, doi:10.1111/j.1365-263X.2009.00973.x.

138. Shalev-Antsel, T.; Winocur-Arias, O.; Friedman-Rubin, P.; Naim, G.; Keren, L.; Eli, I.; Emodi-Perlman, A. The Continuous Adverse Impact of COVID-19 on Temporomandibular Disorders and Bruxism: Comparison of Pre- during- and Post-Pandemic Time Periods. *BMC Oral Health* **2023**, *23*, 716, doi:10.1186/s12903-023-03447-4.
139. Prakash, J.; Ranvijay, K.; Devi, L.S.; Shenoy, M.; Abdul, N.S.; Shivakumar, G.C.; Gupta, P. Assessment of Symptoms Associated with Temporomandibular Dysfunction and Bruxism among Elderly Population: An Epidemiological Survey. *J. Contemp. Dent. Pract.* **2022**, *23*, 393–398.
140. Shahbour, S.A.; Abohamila, N.; EL-Bayoumi, M.H. Prevalence of Sleep Bruxism and Associated Factors in Tanta Preschool Children. *Alex. Dent. J.* **2022**, *47*, 155–162, doi:10.21608/adjalexu.2022.72061.1187.
141. Shokry, S.M.; El Wakeel, E.E.; Al-Maflehi, N.; RasRas, Z.; Fataftah, N.; Abdul Kareem, E. Association between Self-Reported Bruxism and Sleeping Patterns among Dental Students in Saudi Arabia: A Cross-Sectional Study. *Int. J. Dent.* **2016**, *2016*, e4327081, doi:10.1155/2016/4327081.
142. Sierwald, I.; John, M.T.; Schierz, O.; Jost-Brinkmann, P.-G.; Reissmann, D.R. Association of Overjet and Overbite with Esthetic Impairments of Oral Health-Related Quality of Life. *J. Orofac. Orthop. Fortschritte Kieferorthopädie* **2015**, *76*, 405–420, doi:10.1007/s00056-015-0300-x.
143. Silva, S.E. da; Lima, L.C.M. de; Leal, T.R.; Firmino, R.T.; Granville-Garcia, A.F. Use of Electronic Devices, Practice of Sports, and Awake Bruxism in Schoolchildren Aged Eight to Ten Years. *Braz. Oral Res.* **2022**, *36*, e137, doi:10.1590/1807-3107bor-2022.vol36.0137.
144. Tavares Silva, C.; Calabrio, I.R.; Serra-Negra, J.M.; Fonseca-Gonçalves, A.; Maia, L.C. Knowledge of Parents/Guardians about Nocturnal Bruxism in Children and Adolescents. *CRANIO®* **2017**, *35*, 223–227, doi:10.1080/08869634.2016.1201633.
145. Simões-Zenari, M.; Bitar, M.L. Factors Associated to Bruxism in Children from 4 - 6 Years. *Pró-Fono Rev. Atualização Científica* **2010**, *22*, 465–472, doi:10.1590/S0104-56872010000400018.
146. de Siqueira, S.R.D.T.; Vilela, T.T.; Florindo, A.A. Prevalence of Headache and Orofacial Pain in Adults and Elders in a Brazilian Community: An Epidemiological Study. *Gerodontology* **2015**, *32*, 123–131, doi:10.1111/ger.12063.
147. Siva, L.; Krishnamoorthy, V.; Durai, K.S.; Shaheed Ahamed, S.S.; Rajakumari, S.; Catherine, N.C. Comparative Evaluation of Body Mass Index among School Children with and without Bruxism of Age Group of 6-12 Years in Kanchipuram District: A Cross-Sectional Study. *J. Indian Soc. Pedod. Prev. Dent.* **2021**, *39*, 42–46, doi:10.4103/jisppd.jisppd_523_20.
148. Soares, L.G.; Costa, I.R.; Brum Júnior, J.D.S.; Cerqueira, W.S.B.; Oliveira, E.S. de; Douglas de Oliveira, D.W.; Gonçalves, P.F.; Glória, J.C.R.; Tavano, K.T.A.; Flecha, O.D. Prevalence of Bruxism in Undergraduate Students. *Cranio J. Craniomandib. Pract.* **2017**, *35*, 298–303, doi:10.1080/08869634.2016.1218671.
149. Pezzini Soares, J.; Klein, D.; Ximenes, M.; Pereira, C.; Antunes, E.; Dias, L.; Borgatto, A.; Cardoso, M.; Bolan, M. Mouth Breathing and Prevalence of Sleep Bruxism among Preschoolers Aged 2 to 5 Years. *Pesqui. Bras. Em Odontopediatria E Clínica Integrada* **2018**, *18*, 3490–3492, doi:10.4034/PBOCI.2018.181.46.
150. Sousa, H.C.S.; Lima, M. de D.M. de; Dantas Neta, N.B.; Tobias, R.Q.; Moura, M.S. de; Moura, L. de F.A. de D. Prevalence and Associated Factors to Sleep Bruxism in Adolescents from Teresina, Piauí. *Rev. Bras. Epidemiol. Braz. J. Epidemiol.* **2018**, *21*, e180002, doi:10.1590/1980-549720180002.
151. Soares, J.P.; Giacomin, A.; Cardoso, M.; Serra-Negra, J.M.; Bolan, M. Association of Gender, Oral Habits, and Poor Sleep Quality with Possible Sleep Bruxism in Schoolchildren. *Braz. Oral Res.* **2020**, *34*, e019, doi:10.1590/1807-3107bor-2020.vol34.0019.
152. Somay, E.; Tekkarismaz, N. Evaluation of Sleep Bruxism and Temporomandibular Disorders in Patients Undergoing Hemodialysis. *Niger. J. Clin. Pract.* **2020**, *23*, 1375–1380, doi:10.4103/njcp.njcp_630_19.
153. Souza, G.L.N.; Serra-Negra, J.M.; Prado, I.M.; Aguiar, S.O.; Hoffmam, G. de F.E.B.; Pordeus, I.A.; Auad, S.M.; Abreu, L.G. Association of Facial Type with Possible Bruxism and Its Related Clinical Features in Adolescents: A Cross-Sectional Study. *Int. Orthod.* **2020**, *18*, 758–769, doi:10.1016/j.ortho.2020.08.004.
154. Strausz, T.; Ahlberg, J.; Lobbezoo, F.; Restrepo, C.C.; Hublin, C.; Ahlberg, K.; Könönen, M. Awareness of Tooth Grinding and Clenching from Adolescence to Young Adulthood: A Nine-Year Follow-Up. *J. Oral Rehabil.* **2010**, *37*, 497–500, doi:10.1111/j.1365-2842.2010.02071.x.
155. Suwa, S.; Takahara, M.; Shirakawa, S.; Komada, Y.; Sasaguri, K.; Onozuka, M.; Sato, S. Sleep Bruxism and Its Relationship to Sleep Habits and Lifestyle of Elementary School Children in Japan. *Sleep Biol. Rhythms* **2009**, *7*, 93–102, doi:10.1111/j.1479-8425.2009.00394.x.
156. Tachibana, M.; Kato, T.; Kato-Nishimura, K.; Matsuzawa, S.; Mohri, I.; Taniike, M. Associations of Sleep Bruxism with Age, Sleep Apnea, and Daytime Problematic Behaviors in Children. *Oral Dis.* **2016**, *22*, 557–565, doi:10.1111/odi.12492.
157. Tay, K.J.; Ujin, Y.A.; Allen, P.F. Impact of Sleep Bruxism on Oral Health-Related Quality of Life. *Int. J. Prosthodont.* **2020**, *33*, 285–291, doi:10.11607/ijp.6782.
158. Phuong, N.T.T.; Ngoc, V.T.N.; Linh, L.M.; Duc, N.M.; Tra, N.T.; Anh, L.Q. Bruxism, Related Factors and Oral Health-Related Quality of Life Among Vietnamese Medical Students. *Int. J. Environ. Res. Public. Health* **2020**, *17*, E7408, doi:10.3390/ijerph17207408.
159. Tinastepe, N.; Iscan, I. Relationship between Bruxism and Smartphone Overuse in Young Adults. *CRANIO®* **2024**, *42*, 55–62, doi:10.1080/08869634.2021.1909456.
160. Toyama, N.; Ekuni, D.; Taniguchi-Tabata, A.; Yoneda, T.; Kataoka, K.; Yokoi, A.; Uchida, Y.; Fukuhara, D.; Saho, H.; Monirul, I.M.; et al. Associations between Sleep Bruxism, Sleep Quality, and Exposure to Secondhand Smoke in Japanese Young Adults: A Cross-Sectional Study. *Sleep Med.* **2020**, *68*, 57–62, doi:10.1016/j.sleep.2019.09.003.
161. Traebert, E.; Nazário, A.; Nunes, R.; Margreiter, S.; Pereira, K.; Costa, S.; Traebert, J. Prevalence of Sleep Bruxism and Association with Oral Health Conditions in Schoolchildren in a Municipality in Southern Brazil. *Pesqui. Bras. Em Odontopediatria E Clínica Integrada* **2020**, *20*, doi:10.1590/pboci.2020.125.

162. Tsuchiya, M.; Tsuchiya, S.; Momma, H.; Mizuno, K.; Nagatomi, R.; Yaegashi, N.; Arima, T.; Japan Environment and Children's Study Group Prospective Association of Short Sleep Duration in Newborns with Bruxism Behavior in Children: The Japan Environment and Children's Study (JECS). *Sleep Med.* **2022**, *100*, 71–78, doi:10.1016/j.sleep.2022.07.018.
163. Uca, A.U.; Uğuz, F.; Kozak, H.H.; Gümüş, H.; Aksoy, F.; Seyithanoğlu, A.; Kurt, H.G. Antidepressant-Induced Sleep Bruxism: Prevalence, Incidence, and Related Factors. *Clin. Neuropharmacol.* **2015**, *38*, 227–230, doi:10.1097/WNF.0000000000000108.
164. Uma, U.; Fongpisuttikul, P.; Padungpipatbawon, P.; Luyapan, P. Prevalence, Awareness, and Management of Bruxism in Thai Dental Students: A Cross-Sectional Study. *Cranio J. Craniomandib. Pract.* **2021**, 1–7, doi:10.1080/08869634.2021.2015557.
165. Unell, L.; Johansson, A.; Ekbäck, G.; Ordell, S.; Carlsson, G.E. Prevalence of Troublesome Symptoms Related to Temporomandibular Disorders and Awareness of Bruxism in 65- and 75-Year-Old Subjects. *Gerodontology* **2012**, *29*, e772–e779, doi:10.1111/j.1741-2358.2011.00558.x.
166. Us, M.C.; Us, Y.O. Evaluation of the Relationship between Sleep Bruxism and Sleeping Habits in School-Aged Children. *Cranio J. Craniomandib. Pract.* **2023**, *41*, 569–577, doi:10.1080/08869634.2021.1890454.
167. van Selms, M.K.A.; Visscher, C.M.; Naeije, M.; Lobbezoo, F. Bruxism and Associated Factors among Dutch Adolescents. *Community Dent. Oral Epidemiol.* **2013**, *41*, 353–363, doi:10.1111/cdoe.12017.
168. Vieira, K.R.M.; Folchini, C.M.; Heyde, M.D.V.D.; Stuginski-Barbosa, J.; Kowacs, P.A.; Piovesan, E.J. Wake-Up Headache Is Associated With Sleep Bruxism. *Headache* **2020**, *60*, 974–980, doi:10.1111/head.13816.
169. Vieira-Andrade, R.G.; Drumond, C.L.; Martins-Júnior, P.A.; Corrêa-Faria, P.; Gonzaga, G.C.; Marques, L.S.; Ramos-Jorge, M.L. Prevalence of Sleep Bruxism and Associated Factors in Preschool Children. *Pediatr. Dent.* **2014**, *36*, 46–50.
170. Vlăduțu, D.; Popescu, S.M.; Mercuț, R.; Ionescu, M.; Scrieciu, M.; Glodeanu, A.D.; Stănuși, A.; Rîcă, A.M.; Mercuț, V. Associations between Bruxism, Stress, and Manifestations of Temporomandibular Disorder in Young Students. *Int. J. Environ. Res. Public. Health* **2022**, *19*, 5415, doi:10.3390/ijerph19095415.
171. Wetselaar, P.; Vermaire, E. (J. H.); Lobbezoo, F.; Schuller, A.A. The Prevalence of Awake Bruxism and Sleep Bruxism in the Dutch Adult Population. *J. Oral Rehabil.* **2019**, *46*, 617–623, doi:10.1111/joor.12787.
172. Wetselaar, P.; Vermaire, E.J.H.; Lobbezoo, F.; Schuller, A.A. The Prevalence of Awake Bruxism and Sleep Bruxism in the Dutch Adolescent Population. *J. Oral Rehabil.* **2021**, *48*, 143–149, doi:10.1111/joor.13117.
173. Winocur, E.; Messer, T.; Eli, I.; Emodi-Perlman, A.; Kedem, R.; Reiter, S.; Friedman-Rubin, P. Awake and Sleep Bruxism Among Israeli Adolescents. *Front. Neurol.* **2019**, *10*, doi:10.3389/fneur.2019.00443.
174. Winocur-Arias, O.; Winocur, E.; Shalev-Antsel, T.; Reiter, S.; Shifra, L.; Emodi-Perlman, A.; Friedman-Rubin, P. Painful Temporomandibular Disorders, Bruxism and Oral Parafunctions before and during the COVID-19 Pandemic Era: A Sex Comparison among Dental Patients. *J. Clin. Med.* **2022**, *11*, 589, doi:10.3390/jcm11030589.
175. Yachida, W.; Arima, T.; Castrillon, E.E.; Baad-Hansen, L.; Ohata, N.; Svensson, P. Diagnostic Validity of Self-Reported Measures of Sleep Bruxism Using an Ambulatory Single-Channel EMG Device. *J. Prosthodont. Res.* **2016**, *60*, 250–257, doi:10.1016/j.jpor.2016.01.001.
176. Yalçın Yeler, D.; Yılmaz, N.; Koraltan, M.; Aydın, E. A Survey on the Potential Relationships between TMD, Possible Sleep Bruxism, Unilateral Chewing, and Occlusal Factors in Turkish University Students. *CRANIO®* **2017**, *35*, 308–314, doi:10.1080/08869634.2016.1239851.
177. Yıldırım, B.; Kırarslan Karagoz, O.; Tekeli Simsek, A.; Koca, C.; Cicek, M.F. Associations between Self-Reported Bruxism, Sleep Quality, and Psychological Status among Dental Students in Turkey. *Cranio J. Craniomandib. Pract.* **2024**, *42*, 63–68, doi:10.1080/08869634.2021.1909458.
178. Yoshinaka, M.; Ikebe, K.; Furuya-Yoshinaka, M.; Hazeyama, T.; Maeda, Y. Prevalence of Torus Palatinus among a Group of Japanese Elderly. *J. Oral Rehabil.* **2010**, *37*, 848–853, doi:10.1111/j.1365-2842.2010.02100.x.
179. Zani, A.; Lobbezoo, F.; Bracci, A.; Ahlberg, J.; Manfredini, D. Ecological Momentary Assessment and Intervention Principles for the Study of Awake Bruxism Behaviors, Part 1: General Principles and Preliminary Data on Healthy Young Italian Adults. *Front. Neurol.* **2019**, *10*, doi:10.3389/fneur.2019.00169.
180. Zani, A.; Lobbezoo, F.; Bracci, A.; Djukic, G.; Guarda-Nardini, L.; Favero, R.; Ferrari, M.; Aarab, G.; Manfredini, D. Smartphone-Based Evaluation of Awake Bruxism Behaviours in a Sample of Healthy Young Adults: Findings from Two University Centres. *J. Oral Rehabil.* **2021**, *48*, 989–995, doi:10.1111/joor.13212.
