# Supplementary material for: Global Prevalence of Sleep Bruxism and Awake Bruxism in Pediatric and Adult Populations: A Systematic Review and Meta-Analysis
Source: J Clin Med. 2024 Jul 22;13(14):4259. doi: 10.3390/jcm13144259 (PMC11278015; doi:10.3390/jcm13144259)
Supplement: Supplementary file 1 [file jcm-13-04259-s001.zip › Supplementary Material S5 Presentation of the studies included the meta-analysis..pdf]

Systematic Review

Global Prevalence of Sleep Bruxism and Awake Bruxism in Pediatric and Adult Populations: A Systematic Review and Meta-Analysis

Grzegorz Zieliński [1,*], Agnieszka Pająk [2], Marcin Wójcicki [3]

[1] Department of Sports Medicine, Medical University of Lublin, 20-093 Lublin, Poland
[2] Clinic of Anaesthesiology and Paediatric Intensive Care, Medical University of Lublin, Gebali Str. 6, 20-093 Lublin, Poland
[3] Independent Unit of Functional Masticatory Disorder, Medical University of Lublin, 20-093 Lublin, Poland

***** Correspondence: grzegorz.zielinski@umlub.pl

Table S1. Studies Qualified for Analysis.

author	continent	sample_n	age	female_n	male_n	sleep_bruxism_total_n	awake_bruxism_total_n	bruxism_total_n	sleep_bruxism_female_n	awake_bruxism_female_n	sleep_bruxism_male_n	awake_bruxism_male_n	sleep_bruxism_total_prc	awake_bruxism_total_prc	bruxism_total_prc	sleep_bruxism_female_prc	awake_bruxism_female_prc	sleep_bruxism_male_prc	awake_bruxism_male_prc
Aguiar, 2018	South America	152	adults			17	56						11,30	36,80					
Ahlberg, 2023	Europe	11 427	adults	6191	5236	2755			1397		1358		24,11			22,57		25,93	
Ahlberg, 2008	Europe	750	adults			82							10,60						
Alfano, 2018	Noth America	31	minors			10							32,30						
Almutairi, 2021	Asia	1403	adults	912	491	446	632		244	392	202	240	31,79	15,05		24,60	39,50	38,30	45,50
Alonso, 2021	South America	434	minors				190							43,78					
Al-Swaje, 2019	Asia	200	minors					118							59,20				
Amaral, 2022	South America	556	minors	288	268	88			35		53		15,83			12,15		19,78	
Azodo, 2016	Africa	578	adults	321	0	22	52		12	21	10	31	15,40	36,40		20,70	36,20	11,00	36,40
Başpınar, 2023	Asia	249	adults			103	54						58,60	21,70					
Berger, 2017	Europe	508	adults	296	212	94	57		59	33	35	24	18,50	11,20		19,90	11,10	16,50	11,30
Bharti, 2005	Asia	103	minors			12							11,60						
Bolsson, 2023	South America	429	minors			105							23,70						
Borie, 2016	Europe	182	adults	98	84	42			27		15		23,08			27,55		17,86	
Bortoletto, 2017	South America	103	minors					49							47,60				
Botelho, 2019	Europe	427	adults					228							53,40				
Brancher, 2020	South America	556	minors	277	306	163			84		83		30,04			31,70		30,32	
Breda, 2023	Europe	4321	minors	2100	2221	406			176		230		9,40			8,60		10,60	
Bucci, 2018	Europe	47	adults		24	9							19,10						
Cai, 2013	Asia	2591	adults	2591	0	423			423				16,33			16,33			

Câmara-Souza, 2023	South America	69	adults	50	19		26			21		5		37,68			42,79		27,00
Carra, 2011	Noth America	604	minors			91							15,00						
Cavalcante-Leão, 2017	South America	531	minors			85							16,00						
Cavallo, 2016	Europe	278	adults	161	117	88	106		54	66	34	40	31,80	37,90		33,30	40,80	29,10	34,20
Chattrattrai, 2022	Europe	2251	adults	1699	552	352			279		73		15,64			16,40		13,20	
Cheifetz, 2005	Noth America	854	minors					325							38,00				
Ciancaglini, 2008	Europe	489	adults	300	189			155							31,40				
Clementino, 2017	South America	148	minors	76	72	48			31		17		32,43			40,79		23,61	
Colonna, 2021	Europe	506	adults			209							41,30	46,80					
Costa, 2021	South America	475	minors	235	240	225			115		127		47,40			48,94		52,92	
Coutinho, 2020	South America	226	adults			42							18,58						
Silva, 2023	South America	739	minors				110							14,90					
Pontes, 2019	South America	1280	adults			104							8,10						
Dantas-Neta, 2014	South America	306	adults	157	149	40	85	105	22	55	18	30	13,07	57,05	34,30	14,00	22,50	12,10	20,10
Almeida, 2022	Europe	1900	minors	939	961	334			152		152		17,60			16,20		16,20	
Holanda, 2022	South America	1936	adults	1936	0	72			72				3,72			3,72			
Holanda, 2022	South America	289	minors	289	0	7			7				2,42			2,42			
Holanda, 2020	South America	240	adults	108	132	17			11		6		7,08			10,19		4,55	
Bach, 2019	South America	551	minors	260	291	88			35		53		16,00			13,50		18,20	
Siqueira, 2013	South America	890	adults			94							10,56						
Delgado-Delgado, 2020	Europe	59	adults			3							5,00						
Demir, 2004	Asia	965	minors	493	472	122			61		61		12,64			12,40		12,90	
Diéguez-Pérez, 2023	Europe	244	minors			99							40,57						
Costa, 2023	South America	2059	adults	1067	983	507			285		222		24,62			26,71		22,58	
Drumond, 2018	South America	440	minors	241	199	176			86		90		40,00			48,90		51,10	
Duarte, 2019	South America	544	minors	295	249	113			65		48		20,77			22,00		19,20	
Ekman, 2020	Europe	1962	adults			736	198	777	429	133	285	60	37,50	10,10	39,60	42,20	13,10	32,10	6,70
Eli, 2022	Asia	304	adults			87	31						28,60	31,40					
Emmanuelli, 2023	South America	345	adults	77	268	197			164		33		57,10			61,20		42,90	
Emodi-Perlman, 2020	Asia	700	adults			94	111						16,00	13,00					

Emodi-Perlman, 2020	Europe	1092	adults			311	372						28,00	34,00					
Emodi-Perlman, 2016	Asia	1000	minors			92	192						9,20	19,20					
Fonseca, 2011	South America	170	minors	88	82	26			11		15		15,29			12,50		18,29	
Fan, 2018	Australia	912	adults	453	459	289	441		146	246	143	195	31,60	48,30		32,10	54,20	31,10	42,50
Farsi, 2003	Asia	1113	minors					93							8,40				
Ferreira, 2016	South America	496	minors			127							25,60						
Feteih, 2006	Asia	385	minors	230	115			29							7,40				
Flores, 2023	South America	122	adults			56								45,50					
Flueraşu, 2022	Europe	308	adults	133	175	101	47		51	24	50	23	32,79	15,26		38,35	18,05	28,57	13,14
Fulgencio, 2016	South America	1344	minors			205							15,30						
Gao, 2023	Asia	1336	minors	645	691	242			105		137		18,11			16,28		19,83	
Garde, 2014	Asia	832	minors					144							17,30				
Ghafournia, 2012	Asia	400	minors					51							12,75				
Ghalebandi, 2011	Asia	4309	minors	2665	1644	247			136		111		5,73			5,10		6,80	
Goettems, 2017	South America	536	adults	270	266	136			62		77		25,93			22,96		28,95	
Gomes, 2018	South America	761	minors	399	362	205							26,90						
Pineda, 2020	Noth America	144	minors					60							42,00				
Goulart, 2021	South America	366	adults			149	12	152					40,71	3,28	41,53				
Hermesh, 2014	Asia	33	adults			3	1						9,10	3,00					
Huhtela, 2021	Europe	7253	adults	4709	2664	3126	2038		2294	1461	832	577	43,10	28,10		48,72	31,03	31,23	21,66
Insana , 2013	Noth America	4841	minors	2206	2490	2054			733		1321		42,43			33,23		53,05	
Itani, 2013	Asia	99416	minors	49418	47981	10333			5535		4798		10,39			11,20		10,00	
Johansso, 2004	Europe	941	adults	484	457	172			100		72		18,28			20,60		15,80	
Jokubauskas, 2019	Europe	228	adults	188	40	102	132		80	109	22	23	44,70	57,90		42,60	58,00	55,00	57,50
Junqueira , 2013	South America	937	minors			256	19						27,30	2,00					
Karagoz, 2021	Asia	217	adults	132	85	55			43		12		25,35			32,60		14,10	
Kataoka, 2015	Europe	1503	adults	607	896	101			52		49		6,72			8,60		5,50	
Kato , 2012	Asia	1930	adults	911	867	152			65		87		7,90			6,70		9,10	
Kaya, 2022	Asia	250	adults	165	85			100							40,00				
Khatami, 2006	Europe	90	adults			171							19,00						

Khayat, 2019	Asia	149	adults	72	77	43	22						28,90	14,80					
Khoury, 2016	Noth America	6357	adults			547							8,60						
Kilincaslan, 2014	Asia	3485	minors			178							5,10						
Kim, 2017	Asia	936	minors			190							21,10						
Kolak, 2022	Europe	178	adults					62							34,83				
Lam, 2011	Asia	6471	minors	3197	3274	382			150		232		5,90			4,70		7,70	
Leal, 2021	South America	739	minors	372	367	67			32		35		9,07			8,60		9,50	
le, 2022	Noth America	14657	adults					1422							9,70				
Levartovsky, 2022	Asia	387	adults	207	180	97	90						25,00	23,70					
Lima, 2022	South America	105	minors			38							36,20						
Liu, 2006	Asia	5979	minors	3065	2914	389			179		209		6,51			6,10		6,80	
Macfarlane, 2003	Europe	2137	adults				193							0,09					
Maluly, 2020	South America	1042	adults	495	420	127			80		47		12,19			13,90		10,30	
Manfredini, 2017	South America	1556	minors	752	804	408			192		216		26,22			25,53		26,87	
Manfredini , 2012	Asia	397	adults			76	43						19,10	10,80					
Manfredini , 2012	Europe	219	adults			30	25						13,70	11,40					
Massignan, 2019	South America	935	minors			274							29,31						
Melis, 2003	Europe	1014	adults	542	472	117	95		66	50	51	45	11,50	9,40		12,20	9,20	10,80	9,50
Melo , 2014	South America	107	minors	52	55	33							30,80						
Miamoto , 2011	South America	60	minors			15							25,00						
Montero , 2017	Europe	526	adults			106							20,20						
Nahás-Scocate, 2014	South America	873	minors	434	439			251							28,75				
Nakata, 2007	Asia	2680	adults	736	1944	1462			327		1135		54,55			44,40		58,40	
Nazzal, 2023	Asia	194	minors			11							5,67						
Nekora-Azak, 2009	Asia	795	adults	468	327		535			336		199		67,30					
Souza, 2020	South America	403	minors			90	198						22,33	47,89					
Nykinen, 2023	Europe	46	adults				11							23,91					
Okawara, 2022	Asia	770	minors	437	333			135							17,50				
Osses-Anguita, 2023	Europe	274	adults			88	85						32,12	31,02					
Panek , 2012	Europe	303	adults				24							7,92					

Peixoto, 2021	South America	641	adults			372	345						58,03	53,82					
Pereira, 2020	South America	38	adults				22							58,60					
Perlman, 2016	Asia	685	minors	490	195	63	131		43	94	20	37	9,20	19,12		8,70	19,20	10,60	19,20
Prado, 2018	South America	231	adults	125	106	39	55		21		18		16,90	23,90		53,80		46,20	
Ramos, 2021	South America	862	minors	425	437	555			272		283		64,39			64,00		64,76	
Rana, 2017	Asia	409	minors			141							34,50						
Rao, 2011	Asia	147	adults				87							59,20					
Raphael, 2015	Noth America	46	adults	46	0	36			36				78,30			78,30			
Rauch , 2023	Europe	166	adults			27	20	41					16,20	11,90	24,70				
Renner, 2011	South America	1674	minors					491							29,33				
Restrepo, 2016	South America	37	minors			14							37,80						
Ribeiro, 2018	South America	207	minors	180	27	69			62		7		33,33			34,44		25,93	
Rintakoski, 2012	Europe	3126	adults	1445	1681	1003			558		445		32,09			33,20		30,80	
Juliatte, 2022	South America	384	adults	304	80	171	194		144	157	27	37	44,53	50,52		47,40	51,60	33,80	46,30
Rubin, 2018	Africa	153	minors	90	63	26	57		12	36	21	14	16,90	37,30		13,30	40,00	33,30	22,20
Saczuk, 2022	Europe	1018	adults	790	219	595	480		482	401	113	79	58,45	47,15		61,00	50,80	51,60	36,10
Sousa, 2018	South America	594	minors	375	219	132			71		61		22,20			18,90		27,90	
Selms, 2019	Asia	1431	minors			455							31,80						
Selms, 2019	Europe	1131	minors			220							19,50						
Seraj, 2010	Asia	600	minors	286	314			157							26,20				
Serra-Negra, 2021	South America	69	adults				21							30,40					
Serra-Negra, 2021	Europe	136	adults				48							35,29					
Serra-Negra, 2009	South America	652	minors	340	312	230			130		100		35,30			56,50		43,50	
Shahbour, 2022	Africa	1000	minors	483	517	21			82		4		2,10			17,00		0,80	
Shalev-Antsel, 2023	Asia	587	adults	281	306	195	241		107	139	88	102	33,22	41,06		38,00	49,40	28,90	33,20
Prakash, 2022	Asia	600	adults					102							17,00				
Shokry , 2016	Asia	549	adults	482	67	168			143		25		30,60			30,30		37,90	
Sierwald, 2015	Europe	890	adults			209	100						23,50	11,20					
Silva, 2016	South America	134	adults			31							23,10						
Silva, 2016	South America	132	minors			16							11,90						

Siva, 2021	Asia	6122	minors					280							4,57				
Soares, 2017	South America	253	adults			80							31,60						
Soares, 2018	South America	429	minors	202	227	74			30		44		17,25			14,90		19,40	
Somay, 2020	Asia	69	adults			24							35,30						
Suwa, 2009	Asia	1956	minors	983	973	355			164		191		18,45			16,68		19,63	
Tachibana, 2016	Asia	6023	minors	2405	2355	1263							21,00						
Tay, 2020	Asia	2417	adults	2360	57	114			4		110		4,71			0,17		4,55	
Phuong, 2020	Asia	568	adults	305	263	217	133	291					38,20	23,40	51,20				
Tinastepe, 2021	Asia	167	adults			61	97						36,50	58,70					
Toyama, 2020	Asia	1781	adults	811	970	112			65		47		6,29			8,00		4,80	
Traebert, 2020	South America	389	minors	157	232	41			20		21		10,54			8,60		13,40	
Tsuchiya, 2022	Asia	90148	adults	44182	45966	20344			8946		11398		22,60			20,25		24,80	
Uca, 2015	Asia	301	adults			46							15,30						
Uma, 2021	Asia	464	adults	338	126	202	166	90	128	116	74	50	43,53	35,78	19,40	37,87	34,32	35,71	39,68
Unell, 2011	Europe	9093	adults	4632	4461	1635			962		673		17,98			20,77		15,09	
Us, 2021	Asia	500	minors	267	233	160			62		98		32,00			38,70		61,20	
Selms, 2012	Europe	4205	minors			622	366						14,80	8,70					
Vieira, 2020	South America	149	adults	120	29	114			94		20		76,51			78,33		68,97	
Vieira-Andrade, 2014	South America	749	minors	395	354	103			58		45		14,00			15,00		13,00	
Vlăduțu, 2022	Europe	328	adults	212	116	40	108	129	25	71	15	37	12,20	32,93	39,33	11,79	33,49	12,93	31,90
Wetselaar, 2019	Europe	1209	adults	671	538	200	60		125	43	75	17	16,54	4,96		18,60	6,40	13,90	3,20
Wetselaar, 2020	Europe	562	minors	326	236	43	23		25	16	18	7	7,60	4,10		7,80	5,00	7,50	3,20
Prado, 2020	South America	403	minors			125	208						31,00	51,60					
Winocur, 2019	Asia	2347	minors	1344	1255	980	519		324	431	193	378	41,76	22,11		24,10	32,10	15,40	30,10
Winocur-Arias, 2023	Asia	311	adults	133	155	72	96		36	53	36	43	23,15	30,87		27,50	40,50	23,40	27,90
Yachida, 2016	Europe	115	adults			40							35,10						
Yeler, 2016	Asia	519	adults			147							28,32						
Yıldırım, 2021	Asia	212	adults	124	88	51			41		10		24,07			33,06		12,35	
Zani, 2019	Europe	30	adults				11							38,00					
Arsan, 2022	Asia	1688	adults	911	777	333			209		124		19,73			22,94		15,96	

Prado , 2019	South America	1325	adults			204						15,40					
Prado , 2019	South America	1325	minors			318						24,00					
Yoshinaka , 2010	Asia	664	adults	370	294	102	42					15,40	6,30				
Nagamatsu-Sakaguchi , 2008	Asia	127	minors	78	49	80		46		34		63,00		59,00		69,00	
Antunes, 2016	South America	61	minors			21						34,43					
Hilgenberg-Sydney, 2022	South America	50	adults	37	13		24		20		4		48,00		54,05		30,77

References

1. Aguiar, S.O.; Prado, I.M.; Silveira, K.S.R.; Abreu, L.G.; Auad, S.M.; Paiva, S.M.; Serra-Negra, J.M.C. Possible Sleep Bruxism, Circadian Preference, and Sleep-Related Characteristics and Behaviors among Dental Students. *CRANIO®* **2019**, *37*, 389–394, doi:10.1080/08869634.2018.1471113.

2. Ahlberg, J.; Lobbezoo, F.; Manfredini, D.; Piirtola, M.; Hublin, C.; Kaprio, J. Self-Reported Sleep Bruxism and Mortality in 1990–2020 in a Nationwide Twin Cohort. *Journal of Oral Rehabilitation* **2024**, *51*, 125–130, doi:10.1111/joor.13441.

3. Ahlberg, K.; Jahkola, A.; Savolainen, A.; Könönen, M.; Partinen, M.; Hublin, C.; Sinisalo, J.; Lindholm, H.; Sarna, S.; Ahlberg, J. Associations of Reported Bruxism with Insomnia and Insufficient Sleep Symptoms among Media Personnel with or without Irregular Shift Work. *Head & Face Medicine* **2008**, *4*, 4, doi:10.1186/1746-160X-4-4.

4. Alfano, C.A.; Bower, J.L.; Meers, J.M. Polysomnography-Detected Bruxism in Children Is Associated With Somatic Complaints But Not Anxiety. *Journal of Clinical Sleep Medicine* **2018**, *14*, 23–29, doi:10.5664/jcsm.6872.

5. Brandão de Almeida, A.; Rodrigues, R.S.; Simão, C.; de Araújo, R.P.; Figueiredo, J. Prevalence of Sleep Bruxism Reported by Parents/Caregivers in a Portuguese Pediatric Dentistry Service: A Retrospective Study. *International Journal of Environmental Research and Public Health* **2022**, *19*, 7823, doi:10.3390/ijerph19137823.

6. Almutairi, A.F.; Albesher, N.; Aljohani, M.; Alsinanni, M.; Turkistani, O.; Salam, M. Association of Oral Parafunctional Habits with Anxiety and the Big-Five Personality Traits in the Saudi Adult Population. *Saudi Dent J* **2021**, *33*, 90–98, doi:10.1016/j.sdentj.2020.01.003.

7. Alonso, L.S.; Serra-Negra, J.M.; Abreu, L.G.; Martins, I.M.; Tourino, L.F.P.G.; Vale, M.P. Association between Possible Awake Bruxism and Bullying among 8- to 11-Year-Old Children/Adolescents. *International Journal of Paediatric Dentistry* **2022**, *32*, 41–48, doi:10.1111/ipd.12789.

8. Noor Al-Swaje*, A.-A.S., Shatha Al-Khalifah S, Prof. Sana Shafshaks THE PREVALENCE OF BRUXISM AND DENTAL WEAR IN CHILDREN IN RELATION TO SMART DEVICES AND VIDEO GAMES. *INDO AMERICAN JOURNAL OF PHARMACEUTICAL SCIENCES* **2019**, *o6*, 4560–4565, doi:10.5281/zenodo.2579294.

9. Amaral, C.C.; Fernandez, M. dos S.; Jansen, K.; da Silva, R.A.; Boscato, N.; Goettems, M.L. Daily Screen Time, Sleep Pattern, and Probable Sleep Bruxism in Children: A Cross-Sectional Study. *Oral Diseases* **2023**, *29*, 2888–2894, doi:10.1111/odi.14395.

10. Antunes, L.A.A.; Castilho, T.; Marinho, M.; Fraga, R.S.; Antunes, L.S. Childhood Bruxism: Related Factors and Impact on Oral Health-Related Quality of Life. *Special Care in Dentistry* **2016**, *36*, 7–12, doi:10.1111/scd.12140.

11. Arısan, V.; Bedeloğlu, E.; Pişkin, B. Prevalence and Predictors of Bruxism in Two University Clinic Patient Populations with Dental Implants: A Cross-Sectional Analysis. *Cranio* **2022**, 1–12, doi:10.1080/08869634.2022.2071794.

12. Azodo, C.; Ojehanon, P. Bruxism Experience among Undergraduates of a Nigerian University. *Indian J Multidiscip Dent* **2016**, *6*, 14, doi:10.4103/2229-6360.188219.

13. Bach, S. de L.; Moreira, F.P.; Goettems, M.L.; Brancher, L.C.; Oses, J.P.; da Silva, R.A.; Jansen, K. Salivary Cortisol Levels and Biological Rhythm in Schoolchildren with Sleep Bruxism. *Sleep Med* **2019**, *54*, 48–52, doi:10.1016/j.sleep.2018.09.031.

14. Mercan Başpınar, M.; Mercan, Ç.; Mercan, M.; Arslan Aras, M. Comparison of the Oral Health-Related Quality of Life, Sleep Quality, and Oral Health Literacy in Sleep and Awake Bruxism: Results from Family Medicine Practice. *Int J Clin Pract* **2023**, *2023*, 1186278, doi:10.1155/2023/1186278.

15. Berger, M.; Szalewski, L.; Szkutnik, J.; Ginszt, M.; Ginszt, A. Different Association between Specific Manifestations of Bruxism and Temporomandibular Disorder Pain. *Neurol Neurochir Pol* **2017**, *51*, 7–11, doi:10.1016/j.pjnns.2016.08.008.

16. Bharti, B.; Malhi, P.; Kashyap, S. Patterns and Problems of Sleep in School Going Children. *Indian Pediatr* **2006**, *43*, 35–38.

17. Bolsson, G.B.; Knorst, J.K.; Menegazzo, G.R.; Ardenghi, T.M. Impact of Dental Bullying on Bruxism Associated with Poor Sleep Quality among Adolescents. *Braz Oral Res* **2023**, *37*, e36, doi:10.1590/1807-3107BOR-2023.vol37.0036.

18. Borie, L.; Langbour, N.; Guehl, D.; Burbaud, P.; Ella, B. Bruxism in Craniocervical Dystonia: A Prospective Study. *Cranio* **2016**, *34*, 291–295, doi:10.1080/08869634.2015.1120473.

19. Bortoletto, C.C.; Salgueiro, M. da C.C.; Valio, R.; Fragoso, Y.D.; Motta, P. de B.; Motta, L.J.; Kobayashi, F.Y.; Fernandes, K.P.S.; Mesquita-Ferrari, R.A.; Deana, A.; et al. The Relationship between Bruxism, Sleep Quality, and Headaches in Schoolchildren. *J Phys Ther Sci* **2017**, *29*, 1889–1892, doi:10.1589/jpts.29.1889.

20. Botelho, J.; Machado, V.; Proença, L.; Rua, J.; Martins, L.; Alves, R.; Cavacas, M.A.; Manfredini, D.; Mendes, J.J. Relationship between Self-Reported Bruxism and Periodontal Status: Findings from a Cross-Sectional Study. *J Periodontol* **2020**, *91*, 1049–1056, doi:10.1002/JPER.19-0364.

21. Brancher, L.C.; Cademartori, M.G.; Jansen, K.; da Silva, R.A.; Bach, S.; Reyes, A.; Boscato, N.; Goettems, M.L. Social, Emotional, and Behavioral Problems and Parent-Reported Sleep Bruxism in Schoolchildren. *The Journal of the American Dental Association* **2020**, *151*, 327–333, doi:10.1016/j.adaj.2020.01.025.

22. Breda, M.; Belli, A.; Esposito, D.; Di Pilla, A.; Melegari, M.G.; DelRosso, L.; Malorgio, E.; Doria, M.; Ferri, R.; Bruni, O. Sleep Habits and Sleep Disorders in Italian Children and Adolescents: A Cross-Sectional Survey. *J Clin Sleep Med* **2023**, *19*, 659–672, doi:10.5664/jcsm.10400.

23. Bucci, C.; Amato, M.; Zingone, F.; Caggiano, M.; Iovino, P.; Ciacci, C. Prevalence of Sleep Bruxism in IBD Patients and Its Correlation to Other Dental Disorders and Quality of Life. *Gastroenterol Res Pract* **2018**, *2018*, 7274318, doi:10.1155/2018/7274318.

24. Cai, X.-H.; Xie, Y.-P.; Li, X.-C.; Qu, W.-L.; Li, T.; Wang, H.-X.; Lv, J.-Q.; Wang, L.-X. The Prevalence and Associated Risk Factors of Sleep Disorder-Related Symptoms in Pregnant Women in China. *Sleep Breath* **2013**, *17*, 951–956, doi:10.1007/s11325-012-0783-2.

25. Câmara-Souza, M.B.; Carvalho, A.G.; Figueredo, O.M.C.; Bracci, A.; Manfredini, D.; Rodrigues Garcia, R.C.M. Awake Bruxism Frequency and Psychosocial Factors in College Preparatory Students. *Cranio* **2023**, *41*, 178–184, doi:10.1080/08869634.2020.1829289.

26. Carra, M.C.; Huynh, N.; Morton, P.; Rompré, P.H.; Papadakis, A.; Remise, C.; Lavigne, G.J. Prevalence and Risk Factors of Sleep Bruxism and Wake-Time Tooth Clenching in a 7- to 17-Yr-Old Population. *European Journal of Oral Sciences* **2011**, *119*, 386–394, doi:10.1111/j.1600-0722.2011.00846.x.

27. Cavalcante-Leão, B.L.; Todero, S.R.B.; Ferreira, F.M.; Gavião, M.B.D.; Fraiz, F.C. Profile of Orofacial Dysfunction in Brazilian Children Using the Nordic Orofacial Test-Screening. *Acta Odontologica Scandinavica* **2017**, *75*, 262–267, doi:10.1080/00016357.2017.1290823.

28. Cavallo, P.; Carpinelli, L.; Savarese, G. Perceived Stress and Bruxism in University Students. *BMC Research Notes* **2016**, *9*, 514, doi:10.1186/s13104-016-2311-0.

29. Chattrattrai, T.; Blanken, T.F.; Lobbezoo, F.; Su, N.; Aarab, G.; Van Someren, E.J.W. A Network Analysis of Self-Reported Sleep Bruxism in the Netherlands Sleep Registry: Its Associations with Insomnia and Several Demographic, Psychological, and Life-Style Factors. *Sleep Med* **2022**, *93*, 63–70, doi:10.1016/j.sleep.2022.03.018.

30. Cheifetz, A.T.; Osganian, S.K.; Allred, E.N.; Needleman, H.L. Prevalence of Bruxism and Associated Correlates in Children as Reported by Parents. *J Dent Child (Chic)* **2005**, *72*, 67–73.

31. Ciancaglini, R.; Gherlone, E.F.; Radaelli, G. The Relationship of Bruxism with Craniofacial Pain and Symptoms from the Masticatory System in the Adult Population. *Journal of Oral Rehabilitation* **2001**, *28*, 842–848, doi:10.1111/j.1365-2842.2001.00753.x.

32. Clementino, M.A.; Siqueira, M.B.; Serra-Negra, J.M.; Paiva, S.M.; Granville-Garcia, A.F. The Prevalence of Sleep Bruxism and Associated Factors in Children: A Report by Parents. *Eur Arch Paediatr Dent* **2017**, *18*, 399–404, doi:10.1007/s40368-017-0312-x.

33. Colonna, A.; Guarda-Nardini, L.; Ferrari, M.; Manfredini, D. COVID-19 Pandemic and the Psyche, Bruxism, Temporomandibular Disorders Triangle. *CRANIO®* **2021**, *0*, 1–6, doi:10.1080/08869634.2021.1989768.

34. da Costa, S.V.; de Souza, B.K.; Cruvinel, T.; Oliveira, T.M.; Lourenço Neto, N.; Machado, M.A.A.M. Factors Associated with Preschool Children's Sleep Bruxism. *Cranio* **2024**, *42*, 48–54, doi:10.1080/08869634.2021.1903663.

35. Costa, F.D.S.; Fernandez, M.D.S.; Silva-Junior, I.F. da; Karam, S.A.; Chisini, L.A.; Goettems, M.L. Association Involving Possible Sleep Bruxism, Stress, and Depressive Symptoms in Brazilian University Students: A Cross-Sectional Study. *Sleep Sci* **2023**, *16*, e317–e322, doi:10.1055/s-0043-1772808.

36. Nogueira Coutinho MPH, E.; Pereira Rodrigues dos Santos MPH, K.; Henrique Barros Ferreira MPH, E.; Grailea Silva Pinto BHS, R.; de Oliveira Sanchez DPH, M. Association between Self-Reported Sleep Bruxism and Temporomandibular Disorder in Undergraduate Students from Brazil. *CRANIO®* **2020**, *38*, 91–98, doi:10.1080/08869634.2018.1495874.

37. Dantas-Neta, N.B.; Laurentino, J.B.; Souza, C.H. de C. e; Nunes-Dos-Santos, D.L.; Mendes, R.F.; Prado-Júnior, R.R. Prevalence and Potential Factors Associated with Probable Sleep or Awake Bruxism and Dentin Hypersensitivity in Undergraduate Students. *Rev. odontol. UNESP* **2014**, *43*, 245–251, doi:10.1590/rou.2014.040.

38. Delgado-Delgado, R.; Iriarte-Álvarez, N.; Valera-Calero, J.A.; Centenera-Centenera, M.B.; Garnacho-Garnacho, V.E.; Gallego-Sendarrubias, G.M. Association between Temporomandibular Disorders with Clinical and Sociodemographic Features: An Observational Study. *Int J Clin Pract* **2021**, *75*, e13961, doi:10.1111/ijcp.13961.

39. Demir, A.; Uysal, T.; Guray, E.; Basciftci, F.A. The Relationship between Bruxism and Occlusal Factors among Seven- to 19-Year-Old Turkish Children. *Angle Orthod* **2004**, *74*, 672–676, doi:10.1043/0003-3219(2004)074<0672:TRBBAO>2.0.CO;2.

40. Diéguez-Pérez, M.; Ticona-Flores, J.M.; Prieto-Regueiro, B. Prevalence of Possible Sleep Bruxism and Its Association with Social and Orofacial Factors in Preschool Population. *Healthcare* **2023**, *11*, 1450, doi:10.3390/healthcare11101450.

41. Drumond, C.L.; Ramos-Jorge, J.; Vieira-Andrade, R.G.; Paiva, S.M.; Serra-Negra, J.M.C.; Ramos-Jorge, M.L. Prevalence of Probable Sleep Bruxism and Associated Factors in Brazilian Schoolchildren. *Int J Paediatr Dent* **2018**, doi:10.1111/ipd.12443.

42. Duarte, J.; Souza, J.F. de; Cavalcante-Leão, B.; Todero, S.R.B.; Ferreira, F.M.; Fraiz, F.C. Association of Possible Sleep Bruxism with Daytime Oral Habits and Sleep Behavior in Schoolchildren. *Cranio* **2021**, *39*, 372–378, doi:10.1080/08869634.2019.1661113.

43. Ekman, A.; Rousu, J.; Näpänkangas, R.; Kuoppala, R.; Raustia, A.; Sipilä, K. Association of Self-Reported Bruxism with Temporomandibular Disorders – Northern Finland Birth Cohort (NFBC) 1966 Study. *CRANIO®* **2023**, *41*, 212–217, doi:10.1080/08869634.2020.1853306.

44. Eli, I.; Zigler-Garburg, A.; Winocur, E.; Friedman-Rubin, P.; Shalev-Antsel, T.; Levartovsky, S.; Emodi-Perlman, A. Temporomandibular Disorders and Bruxism among Sex Workers-A Cross Sectional Study. *J Clin Med* **2022**, *11*, 6622, doi:10.3390/jcm11226622.

45. Emmanuelli, B.; Araujo, G. de; Knorst, J.K.; Tagliari, C.V. da C.; Baldissera, B.S.; Tuchtenhagen, S. Social Capital and Possible Bruxism during the COVID-19 Pandemic among Brazilian Undergraduates. *Braz. oral res.* **2023**, *37*, e108, doi:10.1590/1807-3107bor-2023.vol37.0108.

46. Emodi-Perlman, A.; Eli, I.; Smardz, J.; Uziel, N.; Wieckiewicz, G.; Gilon, E.; Grychowska, N.; Wieckiewicz, M. Temporomandibular Disorders and Bruxism Outbreak as a Possible Factor of Orofacial Pain Worsening during the COVID-19 Pandemic-Concomitant Research in Two Countries. *J Clin Med* **2020**, *9*, 3250, doi:10.3390/jcm9103250.

47. Emodi Perlman, A.; Lobbezoo, F.; Zar, A.; Friedman Rubin, P.; van Selms, M.K.A.; Winocur, E. Self-Reported Bruxism and Associated Factors in Israeli Adolescents. *Journal of Oral Rehabilitation* **2016**, *43*, 443–450, doi:10.1111/joor.12391.

48. Fan, W.-Y.; Tiang, N.; Broadbent, J.M.; Thomson, W.M. Occurrence, Associations, and Impacts of Nocturnal Parafunction, Daytime Parafunction, and Temporomandibular Symptoms in 38-Year-Old Individuals. *J Oral Facial Pain Headache* **2019**, *33*, 254–259, doi:10.11607/ofph.2221.

49. Farsi, N.M.A. Symptoms and Signs of Temporomandibular Disorders and Oral Parafunctions among Saudi Children. *Journal of Oral Rehabilitation* **2003**, *30*, 1200–1208, doi:10.1111/j.1365-2842.2003.01187.x.

50. Ferreira, N.M.R.; dos Santos, J.F.F.; dos Santos, M.B.F.; Marchini, L. Sleep Bruxism Associated with Obstructive Sleep Apnea Syndrome in Children. *CRANIO®* **2015**, *33*, 251–255, doi:10.1179/2151090314Y.0000000025.

51. Feteih, R.M. Signs and Symptoms of Temporomandibular Disorders and Oral Parafunctions in Urban Saudi Arabian Adolescents: A Research Report. *Head & Face Medicine* **2006**, *2*, 25, doi:10.1186/1746-160X-2-25.

52. Medina Flores, D.; Barragán Nuñez, M.I.; Müller de Quevedo, H.; Bonjardim, L.R.; Rodrigues Conti, P.C. Real Time Evaluation of Awake Bruxism Behaviors in Young Asymptomatic Students and Its Impact on the Masticatory Muscles. *J Prosthet Dent* **2023**, S0022-3913(23)00174-9, doi:10.1016/j.prosdent.2023.03.009.

53. Flueraşu, M.I.; Bocşan, I.C.; Ţig, I.-A.; Iacob, S.M.; Popa, D.; Buduru, S. The Epidemiology of Bruxism in Relation to Psychological Factors. *Int J Environ Res Public Health* **2022**, *19*, 691, doi:10.3390/ijerph19020691.

54. Fonseca, C.M.E.; dos Santos, M.B.F.; Consani, R.L.X.; dos Santos, J.F.F.; Marchini, L. Incidence of Sleep Bruxism among Children in Itanhandu, Brazil. *Sleep Breath* **2011**, *15*, 215–220, doi:10.1007/s11325-010-0427-3.

55. Fulgencio, L.B.; Corrêa-Faria, P.; Lage, C.F.; Paiva, S.M.; Pordeus, I.A.; Serra-Negra, J.M. Diagnosis of Sleep Bruxism Can Assist in the Detection of Cases of Verbal School Bullying and Measure the Life Satisfaction of Adolescents. *Int J Paediatr Dent* **2017**, *27*, 293–301, doi:10.1111/ipd.12264.

56. Gao, Y.; Xu, P.; Aizetiguli, M.; Surong, S.; Zhu, Z.; Zhang, J. Prevalence and Influencing Factors of Sleep Disorders among Preschool Children in Urumqi City: A Cross-Sectional Survey. *Italian Journal of Pediatrics* **2023**, *49*, 68, doi:10.1186/s13052-023-01477-w.

57. Garde, J.B.; Suryavanshi, R.K.; Jawale, B.A.; Deshmukh, V.; Dadhe, D.P.; Suryavanshi, M.K. An Epidemiological Study to Know the Prevalence of Deleterious Oral Habits among 6 to 12 Year Old Children. *J Int Oral Health* **2014**, *6*, 39–43.

58. Ghafournia, M.; Hajenourozali Tehrani, M. Relationship between Bruxism and Malocclusion among Preschool Children in Isfahan. *J Dent Res Dent Clin Dent Prospects* **2012**, *6*, 138–142, doi:10.5681/joddd.2012.028.

59. Ghalebandi, M.; Salehi, M.; Rasoulain, M.; Shooshtari, M.H.; Naserbakht, M.; Salarifar, M.H. Prevalence of Parasomnia in School Aged Children in Tehran. *Iran J Psychiatry* **2011**, *6*, 75–79.

60. Goettems, M.L.; Poletto-Neto, V.; Shqair, A.Q.; Pinheiro, R.T.; Demarco, F.F. Influence of Maternal Psychological Traits on Sleep Bruxism in Children. *Int J Paediatr Dent* **2017**, *27*, 469–475, doi:10.1111/ipd.12285.

61. Gomes, M.C.; Neves, É.T.; Perazzo, M.F.; Souza, E.G.C. de; Serra-Negra, J.M.; Paiva, S.M.; Granville-Garcia, A.F. Evaluation of the Association of Bruxism, Psychosocial and Sociodemographic Factors in Preschoolers. *Braz Oral Res* **2018**, *32*, e009, doi:10.1590/1807-3107bor-2018.vol32.0009.

62. Goulart, A.C.; Arap, A.M.; Bufarah, H.B.; Bismarchi, D.; Rienzo, M.; Syllos, D.H.; Wang, Y.-P. Anxiety, Depression, and Anger in Bruxism: A Cross-Sectional Study among Adult Attendees of a Preventive Center. *Psychiatry Res* **2021**, *299*, 113844, doi:10.1016/j.psychres.2021.113844.

63. Hermesh, H.; Schapir, L.; Marom, S.; Skopski, R.; Barnea, E.; Weizman, A.; Winocur, E. Bruxism and Oral Parafunctional Hyperactivity in Social Phobia Outpatients. *J Oral Rehabil* **2015**, *42*, 90–97, doi:10.1111/joor.12235.

64. Hilgenberg-Sydney, P.B.; Lorenzon, A.L.; Pimentel, G.; Petterle, R.R.; Bonotto, D. Probable Awake Bruxism - Prevalence and Associated Factors: A Cross-Sectional Study. *Dental Press J Orthod* **2022**, *27*, e2220298, doi:10.1590/2177-6709.27.4.e2220298.oar.

65. de Holanda, T.A.; Marmitt, L.P.; Cesar, J.A.; Svensson, P.; Boscato, N. Sleep Bruxism in Puerperal Women: Data from a Population-Based Survey. *Matern Child Health J* **2023**, *27*, 262–271, doi:10.1007/s10995-022-03576-2.

66. Azario de Holanda, T.; Castagno, C.D.; Barbon, F.J.; Mota Freitas, M.P.; Goettems, M.L.; Boscato, N. Influence of Respiratory Allergy and Restless Sleep on Definite Sleep Bruxism: A Cross-Sectional Clinical Study. *Sleep Med* **2020**, *70*, 43–49, doi:10.1016/j.sleep.2020.02.010.

67. Huhtela, O.S.; Näpänkangas, R.; Suominen, A.L.; Karppinen, J.; Kunttu, K.; Sipilä, K. Association of Psychological Distress and Widespread Pain with Sympatoms of Temporomandibular Disorders and Self-Reported Bruxism in Students. *Clinical and Experimental Dental Research* **2021**, *7*, 1154–1166, doi:10.1002/cre2.472.

68. Insana, S.P.; Gozal, D.; McNeil, D.W.; Montgomery-Downs, H.E. Community Based Study of Sleep Bruxism during Early Childhood. *Sleep Med* **2013**, *14*, 183–188, doi:10.1016/j.sleep.2012.09.027.

69. Itani, O.; Kaneita, Y.; Ikeda, M.; Kondo, S.; Yamamoto, R.; Osaki, Y.; Kanda, H.; Suzuki, K.; Higuchi, S.; Ohida, T. Disorders of Arousal and Sleep-Related Bruxism among Japanese Adolescents: A Nationwide Representative Survey. *Sleep Med* **2013**, *14*, 532–541, doi:10.1016/j.sleep.2013.03.005.

70. Johansson, A.; Unell, L.; Carlsson, G.E.; Söderfeldt, B.; Halling, A.; Widar, F. Associations between Social and General Health Factors and Symptoms Related to Temporomandibular Disorders and Bruxism in a Population of 50-year-old Subjects. *Acta Odontologica Scandinavica* **2004**, doi:10.1080/00016350410001649.

71. Jokubauskas, L.; Baltrušaitytė, A.; Pileičikienė, G.; Žekonis, G. Interrelationships between Distinct Circadian Manifestations of Possible Bruxism, Perceived Stress, Chronotype and Social Jetlag in a Population of Undergraduate Students. *Chronobiol Int* **2019**, *36*, 1558–1569, doi:10.1080/07420528.2019.1660356.

72. Juliatte, T. de P.R.; Costa, P.D.; Canaan, J.D.R.; Fonseca, D.C.; Serra-Negra, J.M.; Andrade, E.F.; Castelo, P.M.; Pereira, L.J. Circadian Preference and Its Relationship with Possible Sleep and Awake Bruxism in Adults Assisted by the Public Health System. *Chronobiol Int* **2022**, *39*, 68–76, doi:10.1080/07420528.2021.1973487.

73. Junqueira, T.H.; Nahás-Scocate, A.C.R.; Valle-Corotti, K.M. do; Conti, A.C. de C.F.; Trevisan, S. Association of Infantile Bruxism and the Terminal Relationships of the Primary Second Molars. *Braz Oral Res* **2013**, *27*, 42–47, doi:10.1590/s1806-83242013000100008.

74. Kirarslan Karagoz, O.; Yildirim, B.; Tekeli Simsek, A.; Koca, C.G.; Igneci, M. Possible Sleep and Awake Bruxism, Chronotype Profile and TMD Symptoms among Turkish Dental Students. *Chronobiol Int* **2021**, *38*, 1367–1374, doi:10.1080/07420528.2021.1931279.

75. Kataoka, K.; Ekuni, D.; Mizutani, S.; Tomofuji, T.; Azuma, T.; Yamane, M.; Kawabata, Y.; Iwasaki, Y.; Morita, M. Association Between Self-Reported Bruxism and Malocclusion in University Students: A Cross-Sectional Study. *Journal of Epidemiology* **2015**, *25*, 423–430, doi:10.2188/jea.JE20140180.

76. Kato, T.; Velly, A.M.; Nakane, T.; Masuda, Y.; Maki, S. Age Is Associated with Self-Reported Sleep Bruxism, Independently of Tooth Loss. *Sleep Breath* **2012**, *16*, 1159–1165, doi:10.1007/s11325-011-0625-7.

77. Kaya, M.; Koroglu, A.; Sahin, O. The Relationship of Psychological Status and Sociodemographic Factors with Bruxism among Undergraduate Dental Students: A National Survey. *Niger J Clin Pract* **2022**, *25*, 944–950, doi:10.4103/njcp.njcp_1980_21.

78. Khatami, R.; Zutter, D.; Siegel, A.; Mathis, J.; Donati, F.; Bassetti, C.L. Sleep-Wake Habits and Disorders in a Series of 100 Adult Epilepsy Patients--a Prospective Study. *Seizure* **2006**, *15*, 299–306, doi:10.1016/j.seizure.2006.02.018.

79. Khayat, N.; Winocur, E.; Emodi Perelman, A.; Friedman-Rubin, P.; Gafni, Y.; Shpack, N. The Prevalence of Posterior Crossbite, Deep Bite, and Sleep or Awake Bruxism in Temporomandibular Disorder (TMD) Patients Compared to a Non-TMD Population: A Retrospective Study. *Cranio* **2021**, *39*, 398–404, doi:10.1080/08869634.2019.1650217.

80. Khoury, S.; Carra, M.C.; Huynh, N.; Montplaisir, J.; Lavigne, G.J. Sleep Bruxism-Tooth Grinding Prevalence, Characteristics and Familial Aggregation: A Large Cross-Sectional Survey and Polysomnographic Validation. *Sleep* **2016**, *39*, 2049–2056, doi:10.5665/sleep.6242.

81. Kilincaslan, A.; Yilmaz, K.; Oflaz, S.B.; Aydin, N. Epidemiological Study of Self-Reported Sleep Problems in Turkish High School Adolescents. *Pediatr Int* **2014**, *56*, 594–600, doi:10.1111/ped.12287.

82. Kim, D.S.; Lee, C.L.; Ahn, Y.M. Sleep Problems in Children and Adolescents at Pediatric Clinics. *Korean J Pediatr* **2017**, *60*, 158–165, doi:10.3345/kjp.2017.60.5.158.

83. Kolak, V.; Pavlovic, M.; Aleksic, E.; Biocanin, V.; Gajic, M.; Nikitovic, A.; Lalovic, M.; Melih, I.; Pesic, D. Probable Bruxism and Psychological Issues among Dental Students in Serbia during the COVID-19 Pandemic. *Int J Environ Res Public Health* **2022**, *19*, 7729, doi:10.3390/ijerph19137729.

84. Lam, M.H.B.; Zhang, J.; Li, A.M.; Wing, Y.K. A Community Study of Sleep Bruxism in Hong Kong Children: Association with Comorbid Sleep Disorders and Neurobehavioral Consequences. *Sleep Med* **2011**, *12*, 641–645, doi:10.1016/j.sleep.2010.11.013.

85. Leal, T.R.; de Lima, L.C.M.; Perazzo, M.F.; Neves, É.T.B.; Paiva, S.M.; Serra-Negra, J.M.C.; Ferreira, F.M.; Granville-Garcia, A.F. Influence of the Practice of Sports, Sleep Disorders, and Habits on Probable Sleep Bruxism in Children with Mixed Dentition. *Oral Diseases* **2023**, *29*, 211–219, doi:10.1111/odi.13917.

86. Le, A.; Khoo, E.; Palamar, J.J. Associations between Oral Health and Cannabis Use among Adolescents and Young Adults: Implications for Orthodontists. *International Journal of Environmental Research and Public Health* **2022**, *19*, 15261, doi:10.3390/ijerph192215261.

87. Levartovsky, S.; Msarwa, S.; Reiter, S.; Eli, I.; Winocur, E.; Sarig, R. The Association between Emotional Stress, Sleep, and Awake Bruxism among Dental Students: A Sex Comparison. *J Clin Med* **2021**, *11*, 10, doi:10.3390/jcm11010010.

88. Lima, L.C.M. de; Leal, T.R.; AraúJo, L.J.S. de; Sousa, M.L.C.; Silva, S.E. da; Serra-Negra, J.M.C.; Ferreira, F. de M.; Paiva, S.M.; Granville-Garcia, A.F. Impact of the COVID-19 Pandemic on Sleep Quality and Sleep Bruxism in Children Eight to Ten Years of Age. *Braz Oral Res* **2022**, *36*, e046, doi:10.1590/1807-3107bor-2022.vol36.0046.

89. Liu, X.; Ma, Y.; Wang, Y.; Jiang, Q.; Rao, X.; Lu, X.; Teng, H. Brief Report: An Epidemiologic Survey of the Prevalence of Sleep Disorders among Children 2 to 12 Years Old in Beijing, China. *Pediatrics* **2005**, *115*, 266–268, doi:10.1542/peds.2004-0815I.

90. Macfarlane, T.V.; Blinkhorn, A.S.; Davies, R.M.; Worthington, H.V. Association between Local Mechanical Factors and Orofacial Pain: Survey in the Community. *Journal of Dentistry* **2003**, *31*, 535–542, doi:10.1016/S0300-5712(03)00108-8.

91. Maluly, M.; Dal Fabbro, C.; Andersen, M.L.; Herrero Babiloni, A.; Lavigne, G.J.; Tufik, S. Sleep Bruxism and Its Associations with Insomnia and OSA in the General Population of Sao Paulo. *Sleep Med* **2020**, *75*, 141–148, doi:10.1016/j.sleep.2020.06.016.

92. Manfredini, D.; Lobbezoo, F.; Giancristofaro, R.A.; Restrepo, C. Association between Proxy-Reported Sleep Bruxism and Quality of Life Aspects in Colombian Children of Different Social Layers. *Clin Oral Invest* **2017**, *21*, 1351–1358, doi:10.1007/s00784-016-1901-5.

93. Manfredini, D.; Winocur, E.; Guarda-Nardini, L.; Lobbezoo, F. Self-Reported Bruxism and Temporomandibular Disorders: Findings from Two Specialised Centres. *J Oral Rehabil* **2012**, *39*, 319–325, doi:10.1111/j.1365-2842.2011.02281.x.

94. Martynowicz, H.; Wieckiewicz, M.; Poreba, R.; Wojakowska, A.; Smardz, J.; Januszewska, L.; Markiewicz-Gorka, I.; Mazur, G.; Pawlas, K.; Gac, P. The Relationship between Sleep Bruxism Intensity and Renalase Concentration—An Enzyme Involved in Hypertension Development. *Journal of Clinical Medicine* **2020**, *9*, 16, doi:10.3390/jcm9010016.

95. Massignan, C.; de Alencar, N.A.; Soares, J.P.; Santana, C.M.; Serra-Negra, J.; Bolan, M.; Cardoso, M. Poor Sleep Quality and Prevalence of Probable Sleep Bruxism in Primary and Mixed Dentitions: A Cross-Sectional Study. *Sleep Breath* **2019**, *23*, 935–941, doi:10.1007/s11325-018-1771-y.

96. Melis, M.; Abou-Atme, Y.S. Prevalence of Bruxism Awareness in a Sardinian Population. *CRANIO®* **2003**, *21*, 144–151, doi:10.1080/08869634.2003.11746243.

97. Melo, P.E.D.; Pontes, J.R.D.S. Deleterious Oral Habits in a Group of Children from a Public School in Sao Paulo City. *Rev. CEFAC* **2014**, *16*, 1945–1952, doi:10.1590/1982-0216201418213.

98. Miamoto, C.B.; Pereira, L.J.; Ramos-Jorge, M.L.; Marques, L.S. Prevalence and Predictive Factors of Sleep Bruxism in Children with and without Cognitive Impairment. *Braz Oral Res* **2011**, *25*, 439–445, doi:10.1590/s1806-83242011000500011.

99. Montero, J.; Gómez-Polo, C. Personality Traits and Dental Anxiety in Self-Reported Bruxism. A Cross-Sectional Study. *J Dent* **2017**, *65*, 45–50, doi:10.1016/j.jdent.2017.07.002.

100. Nagamatsu-Sakaguchi, C.; Minakuchi, H.; Clark, G.T.; Kuboki, T. Relationship between the Frequency of Sleep Bruxism and the Prevalence of Signs and Symptoms of Temporomandibular Disorders in an Adolescent Population. *Int J Prosthodont* **2008**, *21*, 292–298.

101. Nahás-Scocate, A.C.R.; Coelho, F.V.; Almeida, V.C. de Bruxism in Children and Transverse Plane of Occlusion: Is There a Relationship or Not? *Dental Press J. Orthod.* **2014**, *19*, 67–73, doi:10.1590/2176-9451.19.5.067-073.oar.

102. Nakata, A.; Takahashi, M.; Ikeda, T.; Hojou, M.; Araki, S. Perceived Psychosocial Job Stress and Sleep Bruxism among Male and Female Workers. *Community Dent Oral Epidemiol* **2008**, *36*, 201–209, doi:10.1111/j.1600-0528.2007.00388.x.

103. Nazzal, H.; Baccar, M.; Ziad, T.; Al-Musfir, T.; Al Emadi, B.; Matoug-Elwerfelli, M.; Narasimhan, S.; Khan, Y.; Reagu, S. Prevalence of Anxiety, Sleep Bruxism and Temporomandibular Disorders during COVID-19 in Qatari Children and Adolescents: A Cross-Sectional Study. *Eur Arch Paediatr Dent* **2023**, *24*, 787–795, doi:10.1007/s40368-023-00847-6.

104. Nekora-Azak, A.; Yengin, E.; Evlioglu, G.; Ceyhan, A.; Ocak, O.; Issever, H. Prevalence of Bruxism Awareness in Istanbul, Turkey. *Cranio* **2010**, *28*, 122–127, doi:10.1179/crn.2010.017.

105. Nykänen, L.; Manfredini, D.; Lobbezoo, F.; Kämppi, A.; Bracci, A.; Ahlberg, J. Assessment of Awake Bruxism by a Novel Bruxism Screener and Ecological Momentary Assessment among Patients with Masticatory Muscle Myalgia and Healthy Controls. *Journal of Oral Rehabilitation* **2024**, *51*, 162–169, doi:10.1111/joor.13462.

106. Okawara, A.; Matsuyama, Y.; Yoshizawa Araki, M.; Unnai Yasuda, Y.; Ogawa, T.; Tumurkhuu, T.; Ganburged, G.; Bazar, A.; Fujiwara, T.; Moriyama, K. Association between Child Abuse and Poor Oral Habits in Mongolian Adolescents. *Int J Environ Res Public Health* **2022**, *19*, 10667, doi:10.3390/ijerph191710667.

107. Osses-Anguita, Á.E.; Sánchez-Sánchez, T.; Soto-Goñi, X.A.; García-González, M.; Alén Fariñas, F.; Cid-Verdejo, R.; Sánchez Romero, E.A.; Jiménez-Ortega, L. Awake and Sleep Bruxism Prevalence and Their Associated Psychological Factors in First-Year University Students: A Pre-Mid-Post COVID-19 Pandemic Comparison. *International Journal of Environmental Research and Public Health* **2023**, *20*, 2452, doi:10.3390/ijerph20032452.

108. Panek, H.; Nawrot, P.; Mazan, M.; Bielicka, B.; Sumisławska, M.; Pomianowski, R. Coincidence and Awareness of Oral Parafunctions in College Students. *Community Dental Health* **2012**, 74–77, doi:10.1922/CDH_2684Panek04.

109. Peixoto, K.O.; Resende, C.M.B.M. de; Almeida, E.O. de; Almeida-Leite, C.M.; Conti, P.C.R.; Barbosa, G.A.S.; Barbosa, J.S. Association of Sleep Quality and Psychological Aspects with Reports of Bruxism and TMD in Brazilian Dentists during the COVID-19 Pandemic. *J Appl Oral Sci* **2021**, *29*, e20201089, doi:10.1590/1678-7757-2020-1089.

110. Pereira, N.C.; Oltramari, P.V.P.; Conti, P.C.R.; Bonjardim, L.R.; de Almeida-Pedrin, R.R.; Fernandes, T.M.F.; de Almeida, M.R.; Conti, A.C.C.F. Frequency of Awake Bruxism Behaviour in Orthodontic Patients: Randomised Clinical Trial: Awake Bruxism Behaviour in Orthodontic Patients. *Journal of Oral Rehabilitation* **2021**, *48*, 422–429, doi:10.1111/joor.13130.

111. Emodi Perlman, A.; Lobbezoo, F.; Zar, A.; Friedman Rubin, P.; van Selms, M.K.A.; Winocur, E. Self-Reported Bruxism and Associated Factors in Israeli Adolescents. *Journal of Oral Rehabilitation* **2016**, *43*, 443–450, doi:10.1111/joor.12391.

112. González-Aragón Pineda, Á.E.; García Pérez, A.; Rosales-Ibáñez, R.; Stein-Gemora, E. Relationship between the Normative Need for Orthodontic Treatment and Oral Health in Mexican Adolescents Aged 13–15 Years Old. *International Journal of Environmental Research and Public Health* **2020**, *17*, 8107, doi:10.3390/ijerph17218107.

113. Pontes, L. da S.; Prietsch, S.O.M. Sleep bruxism: population based study in people with 18 years or more in the city of Rio Grande, Brazil. *Rev Bras Epidemiol* **2019**, *22*, e190038, doi:10.1590/1980-549720190038.

114. Prado, I.M.; Abreu, L.G.; Silveira, K.S.; Auad, S.M.; Paiva, S.M.; Manfredini, D.; Serra, -Negra Júnia Maria Study of Associated Factors With Probable Sleep Bruxism Among Adolescents. *Journal of Clinical Sleep Medicine 14*, 1369–1376, doi:10.5664/jcsm.7276.

115. Prado, I.M.; Abreu, L.G.; Pordeus, I.A.; Amin, M.; Paiva, S.M.; Serra-Negra, J.M. Diagnosis and Prevalence of Probable Awake and Sleep Bruxism in Adolescents: An Exploratory Analysis. *Braz Dent J* **2023**, *34*, 9–24, doi:10.1590/0103-6440202305202.

116. Prado, I.M.; Paiva, S.M.; Fonseca-Gonçalves, A.; Maia, L.C.; Tavares-Silva, C.; Fraiz, F.C.; Ferreira, F.M.; Duarte, J.; Granville-Garcia, A.F.; Costa, E.M.M.B.; et al. Knowledge of Parents/Caregivers about the Sleep Bruxism of Their Children from All Five Brazilian Regions: A Multicenter Study. *Int J Paediatr Dent* **2019**, *29*, 507–523, doi:10.1111/ipd.12486.

117. Ramos, P.F.C.; de Lima, M. de D.M.; de Moura, M.S.; Bendo, C.B.; Moura, L. de F.A. de D.; Lima, C.C.B. Breathing Problems, Being an Only Child and Having Parents with Possible Sleep Bruxism Are Associated with Probable Sleep Bruxism in Preschoolers: A Population-Based Study. *Sleep Breath* **2021**, *25*, 1677–1684, doi:10.1007/s11325-020-02281-0.

118. Alouda, R.; Alshehri, M.; Alnaghmoosh, S.; Shafique, M.; Al-Khudhairy, M.W. Mother's Work Status on Children's Bruxism in a Subset of Saudi Population. *J Int Soc Prev Community Dent* **2017**, *7*, S170–S178, doi:10.4103/jispcd.JISPCD_384_17.

119. Rao, S.K.; Bhat, M.; David, J. Work, Stress, and Diurnal Bruxism: A Pilot Study among Information Technology Professionals in Bangalore City, India. *Int J Dent* **2011**, *2011*, 650489, doi:10.1155/2011/650489.

120. Raphael, K.G.; Janal, M.N.; Sirois, D.A.; Dubrovsky, B.; Klausner, J.J.; Krieger, A.C.; Lavigne, G.J. Validity of Self-Reported Sleep Bruxism among Myofascial Temporomandibular Disorder Patients and Controls. *J Oral Rehabil* **2015**, *42*, 751–758, doi:10.1111/joor.12310.

121. Rauch, A.; Nitschke, I.; Hahnel, S.; Weber, S.; Zenthöfer, A.; Schierz, O. Prevalence of Temporomandibular Disorders and Bruxism in Seniors. *Journal of Oral Rehabilitation* **2023**, *50*, 531–536, doi:10.1111/joor.13450.

122. Renner, A.C.; da Silva, A.A.M.; Rodriguez, J.D.M.; Simões, V.M.F.; Barbieri, M.A.; Bettiol, H.; Thomaz, E.B.A.F.; da Conceição Saraiva, M. Are Mental Health Problems and Depression Associated with Bruxism in Children? *Community Dentistry and Oral Epidemiology* **2012**, *40*, 277–287, doi:10.1111/j.1600-0528.2011.00644.x.

123. Restrepo, C.; Manfredini, D.; Castrillon, E.; Svensson, P.; Santamaria, A.; Alvarez, C.; Manrique, R.; Lobbezoo, F. Diagnostic Accuracy of the Use of Parental-Reported Sleep Bruxism in a Polysomnographic Study in Children. *Int J Paediatr Dent* **2017**, *27*, 318–325, doi:10.1111/ipd.12262.

124. Ribeiro, M.B.; Manfredini, D.; Tavares-Silva, C.; Costa, L.; Luiz, R.R.; Paiva, S.; Serra-Negra, J.M.; Fonseca-Gonçalves, A.; Maia, L.C. Association of Possible Sleep Bruxism in Children with Different Chronotype Profiles and Sleep Characteristics. *Chronobiology International* **2018**, *35*, 633–642, doi:10.1080/07420528.2018.1424176.

125. Rintakoski, K.; Hublin, C.; Lobbezoo, F.; Rose, R.J.; Kaprio, J. Genetic Factors Account for Half of the Phenotypic Variance in Liability to Sleep-Related Bruxism in Young Adults: A Nationwide Finnish Twin Cohort Study. *Twin Research and Human Genetics* **2012**, *15*, 714–719, doi:10.1017/thg.2012.54.

126. Friedman Rubin, P.; Erez, A.; Peretz, B.; Birenboim-Wilensky, R.; Winocur, E. Prevalence of Bruxism and Temporomandibular Disorders among Orphans in Southeast Uganda: A Gender and Age Comparison. *CRANIO®* **2018**, *36*, 243–249, doi:10.1080/08869634.2017.1331784.

127. Saczuk, K.; Lapinska, B.; Wawrzynkiewicz, A.; Witkowska, A.; Arbildo-Vega, H.I.; Domarecka, M.; Lukomska-Szymanska, M. Temporomandibular Disorders, Bruxism, Perceived Stress, and Coping Strategies among Medical University Students in Times of Social Isolation during Outbreak of COVID-19 Pandemic. *Healthcare (Basel)* **2022**, *10*, 740, doi:10.3390/healthcare10040740.

128. van Selms, M.K.A.; Marpaung, C.; Pogosian, A.; Lobbezoo, F. Geographical Variation of Parental-Reported Sleep Bruxism among Children: Comparison between the Netherlands, Armenia and Indonesia. *Int Dent J* **2019**, *69*, 237–243, doi:10.1111/idj.12450.

129. Seraj, B.; Shahrabi, M.; Ghadimi, S.; Ahmadi, R.; Nikfarjam, J.; Zayeri, F.; Taghi, F.P.; Zare, H. The Prevalence of Bruxism and Correlated Factors in Children Referred to Dental Schools of Tehran, Based on Parent's Report. *Iran J Pediatr* **2010**, *20*, 174–180.

130. Serra-Negra, J.M.; Dias, R.B.; Rodrigues, M.J.; Aguiar, S.O.; Auad, S.M.; Pordeus, I.A.; Lombardo, L.; Manfredini, D. Self-Reported Awake Bruxism and Chronotype Profile: A Multicenter Study on Brazilian, Portuguese and Italian Dental Students. *CRANIO®* **2021**, *39*, 113–118, doi:10.1080/08869634.2019.1587854.

131. Serra-Negra, J.M.; Ramos-Jorge, M.L.; Flores-Mendoza, C.E.; Paiva, S.M.; Pordeus, I.A. Influence of Psychosocial Factors on the Development of Sleep Bruxism among Children. *International Journal of Paediatric Dentistry* **2009**, *19*, 309–317, doi:10.1111/j.1365-263X.2009.00973.x.

132. Shalev-Antsel, T.; Winocur-Arias, O.; Friedman-Rubin, P.; Naim, G.; Keren, L.; Eli, I.; Emodi-Perlman, A. The Continuous Adverse Impact of COVID-19 on Temporomandibular Disorders and Bruxism: Comparison of Pre- during- and Post-Pandemic Time Periods. *BMC Oral Health* **2023**, *23*, 716, doi:10.1186/s12903-023-03447-4.

133. Prakash, J.; Ranvijay, K.; Devi, L.S.; Shenoy, M.; Abdul, N.S.; Shivakumar, G.C.; Gupta, P. Assessment of Symptoms Associated with Temporomandibular Dysfunction and Bruxism among Elderly Population: An Epidemiological Survey. *J Contemp Dent Pract* **2022**, *23*, 393–398.

134. Shahbour, S.A.; Abohamila, N.; EL-Bayoumi, M.H. Prevalence of Sleep Bruxism and Associated Factors in Tanta Preschool Children. *Alexandria Dental Journal* **2022**, *47*, 155–162, doi:10.21608/adjalexu.2022.72061.1187.

135. Shokry, S.M.; El Wakeel, E.E.; Al-Maflehi, N.; RasRas, Z.; Fataftah, N.; Abdul Kareem, E. Association between Self-Reported Bruxism and Sleeping Patterns among Dental Students in Saudi Arabia: A Cross-Sectional Study. *International Journal of Dentistry* **2016**, *2016*, e4327081, doi:10.1155/2016/4327081.

136. Sierwald, I.; John, M.T.; Schierz, O.; Jost-Brinkmann, P.-G.; Reissmann, D.R. Association of Overjet and Overbite with Esthetic Impairments of Oral Health-Related Quality of Life. *J Orofac Orthop* **2015**, *76*, 405–420, doi:10.1007/s00056-015-0300-x.

137. Silva, S.E. da; Lima, L.C.M. de; Leal, T.R.; Firmino, R.T.; Granville-Garcia, A.F. Use of Electronic Devices, Practice of Sports, and Awake Bruxism in Schoolchildren Aged Eight to Ten Years. *Braz Oral Res* **2022**, *36*, e137, doi:10.1590/1807-3107bor-2022.vol36.0137.

138. Tavares Silva, C.; Calabrio, I.R.; Serra-Negra, J.M.; Fonseca-Gonçalves, A.; Maia, L.C. Knowledge of Parents/Guardians about Nocturnal Bruxism in Children and Adolescents. *CRANIO®* **2017**, *35*, 223–227, doi:10.1080/08869634.2016.1201633.

139. de Siqueira, S.R.D.T.; Vilela, T.T.; Florindo, A.A. Prevalence of Headache and Orofacial Pain in Adults and Elders in a Brazilian Community: An Epidemiological Study. *Gerodontology* **2015**, *32*, 123–131, doi:10.1111/ger.12063.

140. Siva, L.; Krishnamoorthy, V.; Durai, K.S.; Shaheed Ahamed, S.S.; Rajakumari, S.; Catherine, N.C. Comparative Evaluation of Body Mass Index among School Children with and without Bruxism of Age Group of 6-12 Years in Kanchipuram District: A Cross-Sectional Study. *J Indian Soc Pedod Prev Dent* **2021**, *39*, 42–46, doi:10.4103/jisppd.jisppd_523_20.

141. Soares, L.G.; Costa, I.R.; Brum Júnior, J.D.S.; Cerqueira, W.S.B.; Oliveira, E.S. de; Douglas de Oliveira, D.W.; Gonçalves, P.F.; Glória, J.C.R.; Tavano, K.T.A.; Flecha, O.D. Prevalence of Bruxism in Undergraduate Students. *Cranio* **2017**, *35*, 298–303, doi:10.1080/08869634.2016.1218671.

142. Pezzini Soares, J.; Klein, D.; Ximenes, M.; Pereira, C.; Antunes, E.; Dias, L.; Borgatto, A.; Cardoso, M.; Bolan, M. Mouth Breathing and Prevalence of Sleep Bruxism among Preschoolers Aged 2 to 5 Years. *Pesquisa Brasileira em Odontopediatria e Clínica Integrada* **2018**, *18*, 3490–3492, doi:10.4034/PBOCI.2018.181.46.

143. Sousa, H.C.S.; Lima, M. de D.M. de; Dantas Neta, N.B.; Tobias, R.Q.; Moura, M.S. de; Moura, L. de F.A. de D. Prevalence and Associated Factors to Sleep Bruxism in Adolescents from Teresina, Piauí. *Rev Bras Epidemiol* **2018**, *21*, e180002, doi:10.1590/1980-549720180002.

144. Somay, E.; Tekkarismaz, N. Evaluation of Sleep Bruxism and Temporomandibular Disorders in Patients Undergoing Hemodialysis. *Niger J Clin Pract* **2020**, *23*, 1375–1380, doi:10.4103/njcp.njcp_630_19.

145. Souza, G.L.N.; Serra-Negra, J.M.; Prado, I.M.; Aguiar, S.O.; Hoffmam, G. de F.E.B.; Pordeus, I.A.; Auad, S.M.; Abreu, L.G. Association of Facial Type with Possible Bruxism and Its Related Clinical Features in Adolescents: A Cross-Sectional Study. *Int Orthod* **2020**, *18*, 758–769, doi:10.1016/j.ortho.2020.08.004.

146. Suwa, S.; Takahara, M.; Shirakawa, S.; Komada, Y.; Sasaguri, K.; Onozuka, M.; Sato, S. Sleep Bruxism and Its Relationship to Sleep Habits and Lifestyle of Elementary School Children in Japan. *Sleep and Biological Rhythms* **2009**, *7*, 93–102, doi:10.1111/j.1479-8425.2009.00394.x.

147. Tachibana, M.; Kato, T.; Kato-Nishimura, K.; Matsuzawa, S.; Mohri, I.; Taniike, M. Associations of Sleep Bruxism with Age, Sleep Apnea, and Daytime Problematic Behaviors in Children. *Oral Dis* **2016**, *22*, 557–565, doi:10.1111/odi.12492.

148. Tay, K.J.; Ujin, Y.A.; Allen, P.F. Impact of Sleep Bruxism on Oral Health-Related Quality of Life. *Int J Prosthodont* **2020**, *33*, 285–291, doi:10.11607/ijp.6782.

149. Phuong, N.T.T.; Ngoc, V.T.N.; Linh, L.M.; Duc, N.M.; Tra, N.T.; Anh, L.Q. Bruxism, Related Factors and Oral Health-Related Quality of Life Among Vietnamese Medical Students. *Int J Environ Res Public Health* **2020**, *17*, E7408, doi:10.3390/ijerph17207408.

150. Tinastepe, N.; Iscan, I. Relationship between Bruxism and Smartphone Overuse in Young Adults. *CRANIO®* **2024**, *42*, 55–62, doi:10.1080/08869634.2021.1909456.

151. Toyama, N.; Ekuni, D.; Taniguchi-Tabata, A.; Yoneda, T.; Kataoka, K.; Yokoi, A.; Uchida, Y.; Fukuhara, D.; Saho, H.; Monirul, I.M.; et al. Associations between Sleep Bruxism, Sleep Quality, and Exposure to Secondhand Smoke in Japanese Young Adults: A Cross-Sectional Study. *Sleep Med* **2020**, *68*, 57–62, doi:10.1016/j.sleep.2019.09.003.

152. Traebert, E.; Nazário, A.; Nunes, R.; Margreiter, S.; Pereira, K.; Costa, S.; Traebert, J. Prevalence of Sleep Bruxism and Association with Oral Health Conditions in Schoolchildren in a Municipality in Southern Brazil. *Pesquisa Brasileira em Odontopediatria e Clínica Integrada* **2020**, *20*, doi:10.1590/pboci.2020.125.

153. Tsuchiya, M.; Tsuchiya, S.; Momma, H.; Mizuno, K.; Nagatomi, R.; Yaegashi, N.; Arima, T.; Japan Environment and Children’s Study Group Prospective Association of Short Sleep Duration in Newborns with Bruxism Behavior in Children: The Japan Environment and Children’s Study (JECS). *Sleep Med* **2022**, *100*, 71–78, doi:10.1016/j.sleep.2022.07.018.

154. Uca, A.U.; Uğuz, F.; Kozak, H.H.; Gümüş, H.; Aksoy, F.; Seyithanoğlu, A.; Kurt, H.G. Antidepressant-Induced Sleep Bruxism: Prevalence, Incidence, and Related Factors. *Clin Neuropharmacol* **2015**, *38*, 227–230, doi:10.1097/WNF.0000000000000108.

155. Uma, U.; Fongpisuttikul, P.; Padungpipatbawon, P.; Luyapan, P. Prevalence, Awareness, and Management of Bruxism in Thai Dental Students: A Cross-Sectional Study. *Cranio* **2021**, 1–7, doi:10.1080/08869634.2021.2015557.

156. Unell, L.; Johansson, A.; Ekbäck, G.; Ordell, S.; Carlsson, G.E. Prevalence of Troublesome Symptoms Related to Temporomandibular Disorders and Awareness of Bruxism in 65- and 75-Year-Old Subjects. *Gerodontology* **2012**, *29*, e772–e779, doi:10.1111/j.1741-2358.2011.00558.x.

157. Us, M.C.; Us, Y.O. Evaluation of the Relationship between Sleep Bruxism and Sleeping Habits in School-Aged Children. *Cranio* **2023**, *41*, 569–577, doi:10.1080/08869634.2021.1890454.

158. van Selms, M.K.A.; Visscher, C.M.; Naeije, M.; Lobbezoo, F. Bruxism and Associated Factors among Dutch Adolescents. *Community Dentistry and Oral Epidemiology* **2013**, *41*, 353–363, doi:10.1111/cdoe.12017.

159. Vieira, K.R.M.; Folchini, C.M.; Heyde, M.D.V.D.; Stuginski-Barbosa, J.; Kowacs, P.A.; Piovesan, E.J. Wake-Up Headache Is Associated With Sleep Bruxism. *Headache* **2020**, *60*, 974–980, doi:10.1111/head.13816.

160. Vieira-Andrade, R.G.; Drumond, C.L.; Martins-Júnior, P.A.; Corrêa-Faria, P.; Gonzaga, G.C.; Marques, L.S.; Ramos-Jorge, M.L. Prevalence of Sleep Bruxism and Associated Factors in Preschool Children. *Pediatr Dent* **2014**, *36*, 46–50.

161. Vlăduțu, D.; Popescu, S.M.; Mercuț, R.; Ionescu, M.; Scrieciu, M.; Glodeanu, A.D.; Stănuși, A.; Rîcă, A.M.; Mercuț, V. Associations between Bruxism, Stress, and Manifestations of Temporomandibular Disorder in Young Students. *International Journal of Environmental Research and Public Health* **2022**, *19*, 5415, doi:10.3390/ijerph19095415.

162. Wetselaar, P.; Vermaire, E. (J. H.); Lobbezoo, F.; Schuller, A.A. The Prevalence of Awake Bruxism and Sleep Bruxism in the Dutch Adult Population. *Journal of Oral Rehabilitation* **2019**, *46*, 617–623, doi:10.1111/joor.12787.

163. Wetselaar, P.; Vermaire, E.J.H.; Lobbezoo, F.; Schuller, A.A. The Prevalence of Awake Bruxism and Sleep Bruxism in the Dutch Adolescent Population. *Journal of Oral Rehabilitation* **2021**, *48*, 143–149, doi:10.1111/joor.13117.

164. Winocur, E.; Messer, T.; Eli, I.; Emodi-Perlman, A.; Kedem, R.; Reiter, S.; Friedman-Rubin, P. Awake and Sleep Bruxism Among Israeli Adolescents. *Front. Neurol.* **2019**, *10*, doi:10.3389/fneur.2019.00443.

165. Winocur-Arias, O.; Winocur, E.; Shalev-Antsel, T.; Reiter, S.; Shifra, L.; Emodi-Perlman, A.; Friedman-Rubin, P. Painful Temporomandibular Disorders, Bruxism and Oral Parafunctions before and during the COVID-19 Pandemic Era: A Sex Comparison among Dental Patients. *Journal of Clinical Medicine* **2022**, *11*, 589, doi:10.3390/jcm11030589.

166. Yachida, W.; Arima, T.; Castrillon, E.E.; Baad-Hansen, L.; Ohata, N.; Svensson, P. Diagnostic Validity of Self-Reported Measures of Sleep Bruxism Using an Ambulatory Single-Channel EMG Device. *J Prosthodont Res* **2016**, *60*, 250–257, doi:10.1016/j.jpor.2016.01.001.

167. Yalçın Yeler, D.; Yılmaz, N.; Koraltan, M.; Aydın, E. A Survey on the Potential Relationships between TMD, Possible Sleep Bruxism, Unilateral Chewing, and Occlusal Factors in Turkish University Students. *CRANIO®* **2017**, *35*, 308–314, doi:10.1080/08869634.2016.1239851.

168. Yıldırım, B.; Kırarslan Karagoz, O.; Tekeli Simsek, A.; Koca, C.; Cicek, M.F. Associations between Self-Reported Bruxism, Sleep Quality, and Psychological Status among Dental Students in Turkey. *Cranio* **2024**, *42*, 63–68, doi:10.1080/08869634.2021.1909458.

169. Yoshinaka, M.; Ikebe, K.; Furuya-Yoshinaka, M.; Hazeyama, T.; Maeda, Y. Prevalence of Torus Palatinus among a Group of Japanese Elderly. *J Oral Rehabil* **2010**, *37*, 848–853, doi:10.1111/j.1365-2842.2010.02100.x.

170. Zani, A.; Lobbezoo, F.; Bracci, A.; Ahlberg, J.; Manfredini, D. Ecological Momentary Assessment and Intervention Principles for the Study of Awake Bruxism Behaviors, Part 1: General Principles and Preliminary Data on Healthy Young Italian Adults. *Front. Neurol.* **2019**, *10*, doi:10.3389/fneur.2019.00169.
