# Supplementary material for: Global Prevalence of Sleep Bruxism and Awake Bruxism in Pediatric and Adult Populations: A Systematic Review and Meta-Analysis
Source: J Clin Med. 2024 Jul 22;13(14):4259. doi: 10.3390/jcm13144259 (PMC11278015; doi:10.3390/jcm13144259)
Supplement: Supplementary file 1 [file jcm-13-04259-s001.zip › Supplementary Material S6 Rejected studies from the meta-analysis..pdf]

Systematic Review

Global Prevalence of Sleep Bruxism and Awake Bruxism in Pediatric and Adult Populations: A Systematic Review and Meta-Analysis

Grzegorz Zieliński [1,*], Agnieszka Pająk [2], Marcin Wójcicki [3]

[1] Department of Sports Medicine, Medical University of Lublin, 20-093 Lublin, Poland
[2] Clinic of Anaesthesiology and Paediatric Intensive Care, Medical University of Lublin, Gebali Str. 6, 20-093 Lublin, Poland
[3] Independent Unit of Functional Masticatory Disorder, Medical University of Lublin, 20-093 Lublin, Poland
***** Correspondence: grzegorz.zielinski@umlub.pl

Table S1. Rejected studies from the meta-analysis.

No.	Author	Reason for rejection
1	Ahlberg et al. 2020 [1]	The cohort has been included in the study [2].
2	Ahlberg et al. 2022 [3]	The cohort has been included in the study [2].
3	Ahlberg et al. 2013 [4]	The same cohort is present in the study as in the previous research [5].
4	Ahlberg et al. 2005 [6]	The same cohort is present in the study as in the previous research [5].
5	Ahlberg et al. 2004 [7]	The cohort has been included in the study [5].
6	Rintakoski et al. 2010 [8]	The cohort has been included in the study [2].
7	Rintakoski et al. 2012 [9]	The cohort has been included in the study [2].
8	Serra-Negra et al. 2013 [10]	The same cohort is present in the study as in the previous research [11].
9	Serra-Negra et al. 2010 [12]	The same cohort is present in the study as in the previous research [11].
10	Yoshinaka et al. 2014 [13]	The same cohort is present in the study as in the previous research [14].
11	Colonna et al. 2021 [15]	Data cannot be obtained based on the description of the results.
12	Soares et al. 2020 [16]	Influential case [17].
13	Quadri et al. 2015 [18]	Influential case[17].
14	Serra-Negra et al. 2013 [19]	Potential authorship bias [20].<br>(Repeated measurements: author, subjects, age, and country)
15	Serra-Negra et al. 2014 [21]	Potential authorship bias [20].<br>(Repeated measurements: author, subjects, age, and country)
16	Serra-Negra et al. 2016 [22]	Potential authorship bias [20].<br>(Repeated measurements: author, subjects, age, and country)
17	Serra-Negra et al. 2019 [23]	Potential authorship bias [20].<br>(Repeated measurements: author, subjects, age, and country)
18	Soares et al. 2016 [24]	Potential authorship bias [20].<br>(Repeated measurements: author, subjects, age, and country)
19	Emodi-Perlman et al. 2012 [25]	Potential authorship bias [20].<br>(Repeated measurements: author, subjects, age, and country)
20	Emodi-Perlman et al. 2021 [26]	Potential authorship bias [20].<br>(Repeated measurements: author, subjects, age, and country)
21	Drumond et al. 2017 [27]	Potential authorship bias [20].<br>(Repeated measurements: author, subjects, age, and country)
22	Duarte et al. 2017 [28]	Potential authorship bias [20].<br>(Repeated measurements: author, subjects, age, and country)
23	Huhtela et al. 2016 [29]	Potential authorship bias [20].<br>(Repeated measurements: author, subjects, age, and country)
24	Maluly et al. 2013 [30]	Potential authorship bias [20].<br>(Repeated measurements: author, subjects, age, and country)
25	Winocur et al. 2011 [31]	Potential authorship bias [20].<br>(Repeated measurements: author, subjects, age, and country)

		Q1	Q2	Q3	Q4	Q5	Q6	Q7	Total score [32]
26	Bucci et al. 2022 [33]	1	3	1	1	1	1	0	8
27	Simões-Zenari et al. 2010 [34]	1	3	1	1	1	0	1	8
28	Zani et al. 2021 [35]	1	3	1	1	1	1	0	8
29	Strausz et al. 2010 [36]	1	3	1	1	1	0	1	8
30	Ng et al. 2009 [37]	1	3	1	1	0	0	1	7
31	Rossi et al. 2013 [38]	1	3	1	1	0	0	1	7
32	Bracci et al. 2018 [39]	1	3	1	1	0	1	0	7
33	Dias et al. 2021 [40]	1	3	1	1	0	1	0	7

References

1. Ahlberg, J.; Piirtola, M.; Lobbezoo, F.; Manfredini, D.; Korhonen, T.; Aarab, G.; Hublin, C.; Kaprio, J. Correlates and Genetics of Self-Reported Sleep and Awake Bruxism in a Nationwide Twin Cohort. *J. Oral Rehabil.* **2020**, *47*, 1110–1119, doi:10.1111/joor.13042.
2. Ahlberg, J.; Lobbezoo, F.; Manfredini, D.; Piirtola, M.; Hublin, C.; Kaprio, J. Self-Reported Sleep Bruxism and Mortality in 1990–2020 in a Nationwide Twin Cohort. *J. Oral Rehabil.* **2023**, *51*, 125–130, doi:10.1111/joor.13441.
3. Ahlberg, J.; Lobbezoo, F.; Hublin, C.; Piirtola, M.; Kaprio, J. Self-Reported Sleep Bruxism in 1990 and 2011 in a Nationwide Twin Cohort: Evidence of Trait Persistence and Genetic Liability. *J. Oral Rehabil.* **2022**, *51*, 119–124, doi:10.1111/joor.13368.
4. Ahlberg, J.; Lobbezoo, F.; Ahlberg, K.; Manfredini, D.; Hublin, C.; Sinisalo, J.; Könönen, M.; Savolainen, A. Self-Reported Bruxism Mirrors Anxiety and Stress in Adults. *Med. Oral Patol. Oral Cir. Bucal* **2013**, *18*, e7–e11, doi:10.4317/medoral.18232.
5. Ahlberg, K.; Jahkola, A.; Savolainen, A.; Könönen, M.; Partinen, M.; Hublin, C.; Sinisalo, J.; Lindholm, H.; Sarna, S.; Ahlberg, J. Associations of Reported Bruxism with Insomnia and Insufficient Sleep Symptoms among Media Personnel with or without Irregular Shift Work. *Head Face Med.* **2008**, *4*, 4, doi:10.1186/1746-160X-4-4.
6. Ahlberg, K.; Ahlberg, J.; Könönen, M.; Alakuijala, A.; Partinen, M.; Savolainen, A. Perceived Orofacial Pain and Its Associations with Reported Bruxism and Insomnia Symptoms in Media Personnel with or without Irregular Shift Work. *Acta Odontol. Scand.* **2005**, *63*, 213–217, doi:10.1080/00016350510019937.
7. Ahlberg, J.; Savolainen, A.; Rantala, M.; Lindholm, H.; Könönen, M. Reported Bruxism and Biopsychosocial Symptoms: A Longitudinal Study. *Community Dent. Oral Epidemiol.* **2004**, *32*, 307–311, doi:10.1111/j.1600-0528.2004.00163.x.
8. Rintakoski, K.; Ahlberg, J.; Hublin, C.; Lobbezoo, F.; Rose, R.J.; Murtomaa, H.; Kaprio, J. Tobacco Use and Reported Bruxism in Young Adults: A Nationwide Finnish Twin Cohort Study. *Nicotine Tob. Res.* **2010**, *12*, 679–683, doi:10.1093/ntr/ntq066.
9. Rintakoski, K.; Hublin, C.; Lobbezoo, F.; Rose, R.J.; Kaprio, J. Genetic Factors Account for Half of the Phenotypic Variance in Liability to Sleep-Related Bruxism in Young Adults: A Nationwide Finnish Twin Cohort Study. *Twin Res. Hum. Genet. Off. J. Int. Soc. Twin Stud.* **2012**, *15*, 714–719, doi:10.1017/thg.2012.54.
10. Serra-Negra, J.M.; Paiva, S.M.; Abreu, M.H.; Flores-Mendoza, C.E.; Pordeus, I.A. Relationship between Tasks Performed, Personality Traits, and Sleep Bruxism in Brazilian School Children - A Population-Based Cross-Sectional Study. *PLOS ONE* **2013**, *8*, e80075, doi:10.1371/journal.pone.0080075.
11. Serra-Negra, J.M.; Ramos-Jorge, M.L.; Flores-Mendoza, C.E.; Paiva, S.M.; Pordeus, I.A. Influence of Psychosocial Factors on the Development of Sleep Bruxism among Children. *Int. J. Paediatr. Dent.* **2009**, *19*, 309–317, doi:10.1111/j.1365-263X.2009.00973.x.
12. Serra-Negra, J.M.; Paiva, S.M.; Seabra, A.P.; Dorella, C.; Lemos, B.F.; Pordeus, I.A. Prevalence of Sleep Bruxism in a Group of Brazilian Schoolchildren. *Eur. Arch. Paediatr. Dent.* **2010**, *11*, 192–195, doi:10.1007/BF03262743.
13. Yoshinaka, M.; Ikebe, K.; Furuya-Yoshinaka, M.; Maeda, Y. Prevalence of Torus Mandibularis among a Group of Elderly Japanese and Its Relationship with Occlusal Force. *Gerodontology* **2014**, *31*, 117–122, doi:10.1111/ger.12017.
14. Yoshinaka, M.; Ikebe, K.; Furuya-Yoshinaka, M.; Hazeyama, T.; Maeda, Y. Prevalence of Torus Palatinus among a Group of Japanese Elderly. *J. Oral Rehabil.* **2010**, *37*, 848–853, doi:10.1111/j.1365-2842.2010.02100.x.
15. Colonna, A.; Segù, M.; Lombardo, L.; Manfredini, D. Frequency of Sleep Bruxism Behaviors in Healthy Young Adults over a Four-Night Recording Span in the Home Environment. *Appl. Sci.* **2021**, *11*, 195, doi:10.3390/app11010195.
16. Soares, J.P.; Giacomin, A.; Cardoso, M.; Serra-Negra, J.M.; Bolan, M. Association of Gender, Oral Habits, and Poor Sleep Quality with Possible Sleep Bruxism in Schoolchildren. *Braz. Oral Res.* **2020**, *34*, e019, doi:10.1590/1807-3107bor-2020.vol34.0019.
17. Viechtbauer, W.; Cheung, M.W.-L. Outlier and Influence Diagnostics for Meta-Analysis. *Res. Synth. Methods* **2010**, *1*, 112–125, doi:10.1002/jrsm.11.
18. Quadri, M.F.A.; Mahnashi, A.; Al Almutahhir, A.; Tubayqi, H.; Hakami, A.; Arishi, M.; Alamir, A. Association of Awake Bruxism with Khat, Coffee, Tobacco, and Stress among Jazan University Students. *Int. J. Dent.* **2015**, *2015*, e842096, doi:10.1155/2015/842096.

19. Serra-Negra, J.M.; Tirsa-Costa, D.; Guimarães, F.H.; Paiva, S.M.; Pordeus, I.A. Evaluation of Parents/Guardian Knowledge about the Bruxism of Their Children: Family Knowledge of Bruxism. *J. Indian Soc. Pedod. Prev. Dent.* **2013**, *31*, 153–158, doi:10.4103/0970-4388.117965.
20. Abou-Setta, A.M.; Rabbani, R.; Lix, L.M.; Turgeon, A.F.; Houston, B.L.; Fergusson, D.A.; Zarychanski, R. Can Authorship Bias Be Detected in Meta-Analysis? *Can. J. Anaesth. J. Can. Anesth.* **2019**, *66*, 287–292, doi:10.1007/s12630-018-01268-6.
21. Serra-Negra, J.M.; Scarpelli, A.C.; Tirsa-Costa, D.; Guimarães, F.H.; Pordeus, I.A.; Paiva, S.M. Sleep Bruxism, Awake Bruxism and Sleep Quality among Brazilian Dental Students: A Cross-Sectional Study. *Braz. Dent. J.* **2014**, *25*, 241–247, doi:10.1590/0103-6440201302429.
22. Serra-Negra, J.M.; Ribeiro, M.B.; Prado, I.M.; Paiva, S.M.; Pordeus, I.A. Association between Possible Sleep Bruxism and Sleep Characteristics in Children. *Cranio J. Craniomandib. Pract.* **2017**, *35*, 315–320, doi:10.1080/08869634.2016.1239894.
23. Serra-Negra, J.M.; Lobbezoo, F.; Correa-Faria, P.; Lombardo, L.; Siciliani, G.; Stellini, E.; Manfredini, D. Relationship of Self-Reported Sleep Bruxism and Awake Bruxism with Chronotype Profiles in Italian Dental Students. *CRANIO®* **2019**, *37*, 147–152, doi:10.1080/08869634.2018.1431600.
24. Soares, K.A.N.; Melo, R.M.C.S.; Gomes, M.C.; Perazzo, M.F.; Granville-Garcia, A.F.; Menezes, V.A. Prevalence and Factors Associated to Bruxism in Preschool Children. *J. Public Health* **2016**, *24*, 209–214, doi:10.1007/s10389-016-0713-z.
25. Emodi-Perlman, A.; Eli, I.; Friedman-Rubin, P.; Goldsmith, C.; Reiter, S.; Winocur, E. Bruxism, Oral Parafunctions, Anamnestic and Clinical Findings of Temporomandibular Disorders in Children. *J. Oral Rehabil.* **2012**, *39*, 126–135, doi:10.1111/j.1365-2842.2011.02254.x.
26. Emodi-Perlman, A.; Hochhauser, T.; Winocur, P.; Friedman-Rubin, P.; Eli, I. The Effect of Smartphones on Daytime Sleepiness, Temporomandibular Disorders, and Bruxism among Young Adults. *Quintessence Int. Berl. Ger. 1985* **2021**, *52*, 548–559, doi:10.3290/j.qi.b1244431.
27. Drumond, C.L.; Souza, D.S.; Serra-Negra, J.M.; Marques, L.S.; Ramos-Jorge, M.L.; Ramos-Jorge, J. Respiratory Disorders and the Prevalence of Sleep Bruxism among Schoolchildren Aged 8 to 11 Years. *Sleep Breath. Schlaf Atm.* **2017**, *21*, 203–208, doi:10.1007/s11325-017-1466-9.
28. Duarte, J.; Serra-Negra, J.M.; Ferreira, F.M.; Paiva, S.M.; Fraiz, F.C. Agreement between Two Different Approaches to Assess Parent-Reported Sleep Bruxism in Children. *Sleep Sci. Sao Paulo Braz.* **2017**, *10*, 73–77, doi:10.5935/1984-0063.20170013.
29. Huhtela, O.S.; Näpänkangas, R.; Joensuu, T.; Raustia, A.; Kunttu, K.; Sipilä, K. Self-Reported Bruxism and Symptoms of Temporomandibular Disorders in Finnish University Students. *J. Oral Facial Pain Headache* **2016**, *30*, 311–317, doi:10.11607/ofph.1674.
30. Maluly, M.; Andersen, M.L.; Dal-Fabbro, C.; Garbuio, S.; Bittencourt, L.; De Siqueira, J.T.T.; Tufik, S. Polysomnographic Study of the Prevalence of Sleep Bruxism in a Population Sample. *J. Dent. Res.* **2013**, *92*, S97–S103, doi:10.1177/0022034513484328.
31. Winocur, E.; Uziel, N.; Lisha, T.; Goldsmith, C.; Eli, I. Self-Reported Bruxism - Associations with Perceived Stress, Motivation for Control, Dental Anxiety and Gagging. *J. Oral Rehabil.* **2011**, *38*, 3–11, doi:10.1111/j.1365-2842.2010.02118.x.
32. Zieliński, G.; Pająk, A.; Wójcicki, M. A Meta-Analysis of the Global Prevalence of Sleep Bruxism and Awake Bruxism in Pediatric and Adult Populations. **2024**, doi:10.17605/OSF.IO/ZE786.
33. Bucci, R.; Manfredini, D.; Lenci, F.; Simeon, V.; Bracci, A.; Michelotti, A. Comparison between Ecological Momentary Assessment and Questionnaire for Assessing the Frequency of Waking-Time Non-Functional Oral Behaviours. *J. Clin. Med.* **2022**, *11*, 5880, doi:10.3390/jcm11195880.
34. Simões-Zenari, M.; Bitar, M.L. Factors Associated to Bruxism in Children from 4 - 6 Years. *Pró-Fono Rev. Atualização Científica* **2010**, *22*, 465–472, doi:10.1590/S0104-56872010000400018.
35. Zani, A.; Lobbezoo, F.; Bracci, A.; Djukic, G.; Guarda-Nardini, L.; Favero, R.; Ferrari, M.; Aarab, G.; Manfredini, D. Smartphone-Based Evaluation of Awake Bruxism Behaviours in a Sample of Healthy Young Adults: Findings from Two University Centres. *J. Oral Rehabil.* **2021**, *48*, 989–995, doi:10.1111/joor.13212.
36. Strausz, T.; Ahlberg, J.; Lobbezoo, F.; Restrepo, C.C.; Hublin, C.; Ahlberg, K.; Könönen, M. Awareness of Tooth Grinding and Clenching from Adolescence to Young Adulthood: A Nine-Year Follow-Up. *J. Oral Rehabil.* **2010**, *37*, 497–500, doi:10.1111/j.1365-2842.2010.02071.x.
37. Ng, E.P.; Ng, D.K.; Chan, C.H. Sleep Duration, Wake/Sleep Symptoms, and Academic Performance in Hong Kong Secondary School Children. *Sleep Breath.* **2009**, *13*, 357–367, doi:10.1007/s11325-009-0255-5.
38. Rossi, D.; Manfredini, D. Family and School Environmental Predictors of Sleep Bruxism in Children. *J. Orofac. Pain* **2013**, *27*, 135–141, doi:10.11607/jop.1057.
39. Bracci, A.; Djukic, G.; Favero, L.; Salmaso, L.; Guarda-Nardini, L.; Manfredini, D. Frequency of Awake Bruxism Behaviours in the Natural Environment. A 7-Day, Multiple-Point Observation of Real-Time Report in Healthy Young Adults. *J. Oral Rehabil.* **2018**, *45*, 423–429, doi:10.1111/joor.12627.
40. Dias, R.; Vaz, R.; Rodrigues, M.J.; Serra-Negra, J.M.; Bracci, A.; Manfredini, D. Utility of Smartphone-Based Real-Time Report (Ecological Momentary Assessment) in the Assessment and Monitoring of Awake Bruxism: A Multiple-Week Interval Study in a Portuguese Population of University Students. *J. Oral Rehabil.* **2021**, *48*, 1307–1313, doi:10.1111/joor.13259.
