# Supplementary material for: Global Prevalence of Sleep Bruxism and Awake Bruxism in Pediatric and Adult Populations: A Systematic Review and Meta-Analysis
Source: J Clin Med. 2024 Jul 22;13(14):4259. doi: 10.3390/jcm13144259 (PMC11278015; doi:10.3390/jcm13144259)
Supplement: Supplementary file 1 [file jcm-13-04259-s001.zip › Supplementary Material S7 Adapted Joanna Briggs classification..pdf]

Systematic Review

Global Prevalence of Sleep Bruxism and Awake Bruxism in Pediatric and Adult Populations: A Systematic Review and Meta-Analysis

Grzegorz Zieliński [1,*], Agnieszka Pająk [2], Marcin Wójcicki [3]

[1] Department of Sports Medicine, Medical University of Lublin, 20-093 Lublin, Poland
[2] Clinic of Anaesthesiology and Paediatric Intensive Care, Medical University of Lublin, Gebali Str. 6, 20-093 Lublin, Poland
[3] Independent Unit of Functional Masticatory Disorder, Medical University of Lublin, 20-093 Lublin, Poland
***** Correspondence: grzegorz.zielinski@umlub.pl

Table S1. Joanna Briggs classification determined by based on [1,2].

Guideline		Checklist	Classification			
			Y	N	U	
**Prevalence data**	Q1	Was the sample frame appropriate to address the target population?	The sample frame included groups of individuals that are representative of the target population.	The sample frame is not representative of the target population.	No clear description of the sample frame.	[1]*
	Q2	Were study participants recruited in an appropriate way?	A random sampling of the population was performed, and the sampling method was clearly reported.	Studies that did not use a random sampling or used a convenience sample.	No clear description about the sampling method.	[1]*
	Q3	Was the sample size adequate?	An a priori sample size calculation was performed, and number of included participants is adequate (>400).	No sample size calculation was performed, or sample size was not adequate.	Unclear whether the sample size was adequate.	[1,3]**
	Q4	Were the study subjects and setting described in detail?	Study sample was described in sufficient detail (place where participants were recruited, geographic location).	No description of the population details.	Some population details were reported, although relevant data are missing.	[1]*
	Q5	Was data analysis conducted with sufficient coverage of the identified sample?	100%-80% of the assumed group was examined.	80% of the announced group was included in the analysis.	No information was included in the analysis relative to the assumptions.	[1]**
	Q6	Were valid methods used for the identification of the condition?	Patients who reported Sleep Bruxism and Awake Bruxism, diagnosed through	Studies that did not define the diagnostic criteria for Sleep	No clear description about which methods were used to diagnose Sleep	[1]**

		clinical evaluation or through electromyography, were included.	Bruxism and Awake Bruxism.	Bruxism and Awake Bruxism. Whether Sleep Bruxism and Awake Bruxism were investigated or not is unclear.	
Q7	Was the condition measured in a standard, reliable way for all participants?	Clear description of the use of polysomnography or electromyography, or clinical examination.	Use of questionnaire or self-report only. No description of the valuation method.	Lack of clear information.	[1,2]**
Q8	Was there appropriate statistical analysis?	When it was detailed which analytical techniques were used.	Did not presented the statistical analysis.	No clear description about the statistical analyses conducted.	[1,2]**
Q9	Was the response rate adequate, and if not, was the low response rate managed appropriately?	The response rate was adequate (more than 80%) or the authors adequately managed and explained why the response rate was low.	Response rate was low (less than 80%) and the authors did not adequately justify the reasons.	No information about response rates.	[1]*

Notes:

- Y— yes;
- N—no;
- U—unclear;
- NA—not applicable;
- AB—awake bruxism
- ‘’*’’ — Questions and answers directly taken from the paper.
- ‘’**’’ — Question modified based on the studies.

References

1. Archer, A.B.; Da-Cas, C.D.; Valesan, L.F.; Cunha, T.C.A.; Januzzi, E.; Garanhani, R.R.; de La Torre Canales, G.; de Souza, B.D.M. Prevalence of Awake Bruxism in the Adult Population: A Systematic Review and Meta-Analysis. *Clin Oral Invest* **2023**, *27*, 7007–7018, doi:10.1007/s00784-023-05302-w.
2. Soares, J.P.; Moro, J.; Massignan, C.; Cardoso, M.; Serra-Negra, J.M.; Maia, L.C.; Bolan, M. Prevalence of Clinical Signs and Symptoms of the Masticatory System and Their Associations in Children with Sleep Bruxism: A Systematic Review and Meta-Analysis. *Sleep Medicine Reviews* **2021**, *57*, 101468, doi:10.1016/j.smrv.2021.101468.
3. Kyriazos, T. Applied Psychometrics: Sample Size and Sample Power Considerations in Factor Analysis (EFA, CFA) and SEM in General. *Psychology* **2018**, *09*, 2207–2230, doi:10.4236/psych.2018.98126.
