# Supplementary material for: Global Prevalence of Sleep Bruxism and Awake Bruxism in Pediatric and Adult Populations: A Systematic Review and Meta-Analysis
Source: J Clin Med. 2024 Jul 22;13(14):4259. doi: 10.3390/jcm13144259 (PMC11278015; doi:10.3390/jcm13144259)
Supplement: Supplementary file 1 [file jcm-13-04259-s001.zip › Supplementary Material S9 Detailed description of the conducted meta-analysis..pdf]

Systematic Review

Global Prevalence of Sleep Bruxism and Awake Bruxism in Pediatric and Adult Populations: A Systematic Review and Meta-Analysis

Grzegorz Zieliński [1,*], Agnieszka Pająk [2], Marcin Wójcicki [3]

[1] Department of Sports Medicine, Medical University of Lublin, 20-093 Lublin, Poland
[2] Clinic of Anaesthesiology and Paediatric Intensive Care, Medical University of Lublin, Gebali Str. 6, 20-093 Lublin, Poland
[3] Independent Unit of Functional Masticatory Disorder, Medical University of Lublin, 20-093 Lublin, Poland

* Correspondence: grzegorz.zielinski@umlub.pl

1. Synthesis methods

The objective of this analysis was to estimate the prevalence of bruxism across the entire sample, with additional stratifications by age and continent, as well as by gender. This approach allows for a comprehensive assessment of demographic variations in bruxism occurrence.

In this meta-analysis, the threshold for statistical significance was established at $\alpha = 0.05$. The distribution of categorical variables pertaining to the characteristics of the study samples was detailed by reporting frequencies and percentages for each category. The distribution of numerical variables was described using the median (*Mdn*) and interquartile range, specifically the first (*Q1*) and third (*Q3*) quartiles, as metrics of dispersion.

Pooling effect size estimation

To estimate the pooled effect size, we employed the Generalized Linear Mixed-Effects Model (GLMM) with a binomial logit link. The GLMM integrates both fixed and random effects, is particularly well-suited for meta-analyses as it adeptly handles variations within and across a range of studies. Unlike the traditional inverse-variance method, the GLMM excels in managing heterogeneous datasets, ensuring a robust analysis of data involving diverse measurement scales such as counts and proportions [1,2].

The overall proportions were calculated employing the logit transformation, followed by back-transformation to the original scale. The selection of this transformation method was guided by specific

characteristics of the dataset and the distribution of proportion values, with the assumption that there were no extreme values (i.e., very small or very large proportions). Confidence intervals for individual study results were estimated using the Agresti-Coull interval [3].

Heterogeneity among study results was quantified using the τ^2 , the square root of tau-squared (τ), and the I^2 statistic [4]. The Higgins and Thompson's H statistic was also calculated to measure the consistency of effect sizes across studies.

Variance components for τ^2 were estimated using the maximum-likelihood (ML) estimator which facilitates an accurate estimation of between-study variance, which is critical given the substantial heterogeneity indicated by our data. The confidence intervals for the I^2 and H statistics, were estimated using methods based on the chi-squared distribution. The presence of heterogeneity was tested using both the Wald and the Likelihood Ratio Test.

The evaluation of subgroup differences within the meta-analysis was performed using the Q-test for heterogeneity. The prediction interval for the prevalence of bruxism was estimated using the t-distribution.

The pooled effect of the studies was graphically depicted using a forest plot. This visualization method systematically represents the individual study results alongside their aggregated effect, facilitating a comprehensive overview of the data.

Leave-one-out diagnostics

We utilized several leave-one-out diagnostics such as externally standardized residual, DFFITS value, Cook's distance, covariance ratio, the leave-one-out amount of (residual) heterogeneity, the leave-one-out test statistic of the test for (residual) heterogeneity, DFBETAS values [5–10].

A case may be considered to be 'influential' if at least one of the following is true:

1. If the absolute value of DFFITS exceeds $3 \times \sqrt{\frac{p}{k-p}}$, where p denotes the number of model coefficients and k the total number of cases. This threshold identifies cases that significantly affect the model's predictions..
2. A case is considered influential if the lower tail probability of a chi-square distribution, calculated using the degrees of freedom defined by the Cook's distance, exceeds 50%. This metric highlights cases that have a substantial impact on the parameter estimates across the model.

3. Influence is attributed to a case if its hat value surpasses $3 \times \frac{p}{k}$. This measure quantifies the leverage of a case, indicating its relative ability to influence the fit of the model disproportionately.
4. A case is marked as influential if any of its DFBETAS values exceed 1.0. This condition reveals cases that, when excluded, lead to a significant change in the estimated regression coefficients, shifting them by more than one standard deviation.

Publication bayes

To evaluate publication bias in the study, a funnel plot was utilized for graphical representation. The asymmetry in the funnel plot was quantitatively assessed using Egger's test as the primary statistical approach. This method involves regressing the standardized effect estimates against their precision, in accordance with the established guidelines by Egger et al. [11]. The regression analysis uses the standard error of the estimates as a predictor, with each study's impact on the analysis adjusted by weighting inversely to their variance.

Characteristics of the statistical tool

Analyses were conducted using the R Statistical language (version 4.3.1; R Core Team, 2023) on Windows 10 Pro 64 bit (build 19045), using the packages *meta* (version 6.5.0; [12]), *dmetar* (version 0.1.0; [13]), *report* (version 0.5.7; [14]), *gtsummary* (version 1.7.2; [15]), *dplyr* (version 1.1.3; [16]) and *psych* (version 2.3.9; [17]).

Characteristics of the meta-analysis sample

The prevalence of bruxism was analyzed across 176 scientific studies (170 publications analyzed 176 populations). Six studies [18–23] analyzed more than one population/age group) with sample sizes varied from 30 to 99416 (*Mdn* = 533.5, *Q1* = 224,3, *Q3* = 1097,3). Of these, 97 studies (55.11%) focused on adult populations (aged over 18 years), while the remaining 79 studies targeted minors (up to 18 years of age). Geographically, the studies were distributed as follows: 63 studies (36.63%) involved individuals in South America, 58 studies (33.72%) in Asia, 43 studies (25.00%) in Europe, and 12 studies (6.82%) in North America.

Additionally, the studies explored the incidence of bruxism with regard to two specific variables: gender and the time of occurrence, distinguishing between events during sleep and those occurring after waking.

1. Results

2.1 Meta-analysis of global bruxism (sleep and awake) prevalence

In the meta-analysis investigating the prevalence of bruxism across global populations, a total of 137 studies were included (39 studies have no data about the overall prevalence), encompassing 389,658 observations and 61,330 recorded events of bruxism. Using a random effects model, the pooled prevalence of bruxism was estimated at 22.22%, with a 95% confidence interval ranging from 19.59% to 25.11%. Additionally, a prediction interval was calculated, extending from 4.23% to 64.87%, indicating substantial variability in bruxism prevalence across different settings and populations.

Quantitative assessment of heterogeneity revealed significant variability among the studies: tau squared $\tau^2 = 0.88$, $\tau = 0.94$, and the $I^2 = 99.4\%$, indicating that 99.4% of the variability in effect estimates could be attributed to heterogeneity rather than chance. This was further supported by an $H = 12.50$, suggesting substantial dispersion in the effect sizes across the studies.

The Wald test resulted in $\chi^2(136) = 21{,}265.78$, $p < 0.001$, indicating significant heterogeneity. Similarly, the LRT yielded a chi-squared value of $\chi^2(136) = 23{,}602.65$, $p < 0.001$, confirming the presence of substantial heterogeneity among the included studies.

The forest plot illustrating the overall effect of the studies on bruxism can be reviewed in the Supplementary Material S10.

The leave-one-out diagnostics conducted revealed that none of the studies were deemed influential within the context of our analysis.

The visualization of publication bias using a funnel plot is available in the Supplementary Material S11.

The results of Egger's test, yielding a t-value of 1.74 with 135 degrees of freedom, resulted in a $p = 0.084$. These findings did not demonstrate statistically significant evidence of publication bias, they also highlighted the presence of considerable residual heterogeneity ($\tau^2 = 154.07$).

1.2 Meta-analysis of global bruxism (sleep and awake) prevalence across continents

The meta-analysis conducted on the global prevalence of bruxism across continents incorporated data from 136 studies, encompassing a total of 355,423 observations and 61,309 events. Utilizing a random effects model, the aggregated prevalence of bruxism was estimated at 22.55%, with a 95% confidence interval ranging from 19.93% to 25.40%. The prediction interval, calculated based on the t-distribution with 134 degrees of freedom, was notably wider, ranging from 4.50% to 64.27%, reflecting substantial variability in the prevalence estimates that could be expected in new studies.

Quantification of heterogeneity revealed extremely high variability across studies, with an I^2 = 99.4%, τ^2 = 0.84, and an H = 12.52, indicating a significant variation beyond chance. Both the Wald and LRT for heterogeneity confirmed this with a $p < 0.001$ suggesting robust evidence against homogeneity.

The subgroup analysis by continent in table 1 revealed significant variability in the prevalence of bruxism, reflecting the diverse geographical patterns of this condition. North America exhibits the highest prevalence rate at 29.07%, with a notably broad 95% confidence interval ranging from 15.85% to 47.14%. This wide interval suggests a high degree of uncertainty or variability in the data, possibly due to fewer studies (only 8 in total) contributing to this regional estimate, as indicated by the highest τ^2 = 1.22, suggesting substantial heterogeneity among the studies.

Europe and South America presented more moderate prevalence rates of 22.17% and 24.89%, respectively, with narrower confidence intervals, indicating more precise estimates. Europe's studies, although more numerous (29 studies), still showed a high level of heterogeneity (I^2 = 98.6%), which was slightly lower than that of South America, which had an I^2 = 97.9%.

Asia, with 43 studies, reported the lowest prevalence of bruxism at 19.10%. However, it also presented a high level of heterogeneity (I^2 = 99.7%), similar to North America, and a high τ^2 = 1.03. This suggests that, despite having a larger number of studies, there is significant variation in the study results, which could be attributed to differences in study methodologies, populations, and diagnostic criteria across the continent.

The Q-statistics further support the presence of significant heterogeneity within each continent's subgroup, with extremely high values observed, particularly in Asia (Q = 12177.51). This reinforces the complexity and variability of bruxism prevalence across different geographical and cultural contexts.

Table 1. Variability in bruxism prevalence: a subgroup analysis by continent

continent	*k*	*proportion*	*CI 95%*	τ^2	*Q*	I^2

Europe	29	0.22	0.18 – 0.27	0.52	2023.04	98.6%
North America	8	0.29	0.16 – 0.47	1.22	3041.78	99.8%
South America	56	0.25	0.21 – 0.29	0.74	2580.68	97.9%
Asia	43	0.19	0.15 – 0.24	1.03	12177.51	99.7%

Note: k – number of studies; *CI 95%* – confidence interval 95%.

The test for subgroup differences, conducted using a random effects model, yielded a $Q(3) = 3.79$, $p = 0.289$, indicating no statistically significant differences in bruxism prevalence between continents.

The forest plot illustrating the overall effect of the studies on bruxism across continents can be reviewed in the Supplementary Material S10.

1.3 Meta-analysis of global sleep bruxism prevalence

The comprehensive meta-analysis examining the global prevalence of sleep bruxism incorporated data from 144 studies, totaling 353,994 observations and 64,574 events. The analysis, employing a random effects model, estimated the prevalence of sleep bruxism at 20.99%, with a 95% confidence interval that stretches from 18.69% to 23.50%. This prevalence estimate highlights the notable incidence of sleep bruxism among the global population studied.

A particularly wide prediction interval, ranging from 4.47% to 60.17% is indicative of the substantial variability expected in future studies and emphasizes the diverse manifestation of sleep bruxism across different populations and study settings.

The analysis revealed profound heterogeneity among the included studies, as evidenced by an $I^2 = 99.3\%$, which suggests that 99.3% of the observed variance is due to differences between studies rather than random chance. The $\tau^2 = 0.77$, with a $\tau = 0.88$, further confirming significant variability in the estimates of sleep bruxism prevalence across the studies.

The Wald and LRT, both yielded a Q-value, respectively at 21777.87 and 24127.89 with 143 degrees of freedom, and $p < 0.001$. These results decisively indicate the presence of heterogeneity among the studies, underscoring the challenges in deriving a singular, cohesive estimate for sleep bruxism prevalence.

The forest plot illustrating the overall effect of the studies on sleep bruxism can be reviewed in the file Supplementary Material S10.

The leave-one-out diagnostics conducted revealed that none of the studies were deemed influential within the context of our analysis.

The visualization of publication bias using a funnel plot is available in the Supplementary Material S11.

The result of the Egger's test yielded a t (142) = 1.13, p = 0.258, which suggests that there is no statistically significant evidence of publication bias in the dataset. In other words, the symmetry of the funnel plot implies that there is a balanced representation of studies with both high and low variance, and that smaller studies with less favorable outcomes are not disproportionately missing from the analysis.

It is also noteworthy that the analysis accounted for multiplicative residual heterogeneity variance (τ^2 = 151.99), which is exceptionally high. This indicates considerable residual heterogeneity among the study estimates even after accounting for sampling variance.

1.4 Meta-analysis of global sleep bruxism prevalence across continents

The meta-analysis assessing the global prevalence of sleep bruxism across continents included data from 140 studies with 351,351 observations and 64,216 reported events. Utilizing a random effects model, the overall estimated prevalence was calculated as 21.48%, with a 95% confidence interval of 19.18% to 23.98%. This model also gave a wide prediction interval from 4.85% to 59.46%, suggesting significant variation in bruxism prevalence expected across different studies and populations.

The high heterogeneity observed in the analysis is quantified by an I^2 = 99.4%, indicating that 99.4% of the total variation among studies is due to heterogeneity rather than chance. This is substantiated by a τ^2 = 0.72 and a τ = 0.85, affirming substantial differences in study results. Heterogeneity was further confirmed through significant Q values from both Wald and LRT statistics (p <0.001), which pointed to the non-random distribution of effect sizes across studies.

The subgroup analysis of sleep bruxism prevalence by continent in table 2 reveals a complex picture of how this condition manifests across different geographical regions, influenced by varying study characteristics, methodologies, and potentially underlying regional factors affecting bruxism rates.

In Europe, where 35 studies were analyzed, the prevalence of sleep bruxism is estimated at 21%. The narrower confidence interval of 18% to 26% suggests a somewhat consistent measurement across

studies, albeit the high $I^2 = 99.1\%$ indicates that the variability among study findings is predominantly due to heterogeneity rather than chance. This could be reflective of different population demographics studied or varying diagnostic criteria used across European countries.

North America presents the highest prevalence at 31%, but this figure comes with a wide confidence interval from 12% to 57%, largely attributable to the small number (only 5) of studies conducted there. Such a wide interval indicates a significant uncertainty in the estimate, compounded by the highest $\tau^2 = 1.52$ among the groups, suggesting that the few studies included may be highly diverse in terms of methodology or population characteristics. The extremely high $I^2 = 99.7\%$ further supports this view, pointing to substantial inconsistencies among the findings of these studies.

In contrast, South America, with the highest number of studies (54), shows a prevalence of 23%. The relatively tighter confidence interval of 19% to 28% alongside a $\tau^2 = 0.75$ suggests that while there is considerable variation among the studies, it is somewhat less than that observed in North America. The high $I^2 = 98.1\%$ still underscores significant heterogeneity, indicating that despite a larger data pool, the consistency across study results is limited.

Asia, with 46 studies, reports the lowest prevalence at 19%, and its confidence interval ranges from 15% to 23%. The $\tau^2 = 0.71$, closely aligned with that of South America, and the extremely high $Q = 10727.90$ combined with an $I^2 = 99.6\%$ highlight that heterogeneity is also a major factor in Asian studies. This suggests that, similar to other continents, varying study designs, populations, and diagnostic criteria across Asian countries contribute to the diverse findings.

Table 2. Variability in sleep bruxism prevalence: a subgroup analysis by continent

continent	*k*	*proportion*	*CI 95%*	τ^2	*Q*	I^2
Europe	35	0.21	0.18 – 0.26	0.49	3855.24	99.1%
North America	5	0.31	0.12 – 0.57	1.52	1575.75	99.7%
South America	54	0.23	0.19 – 0.28	0.75	2800.83	98.1%
Asia	46	0.19	0.15 – 0.23	0.71	10727.90	99.6%

Note: k – number of studies; *CI 95%* – confidence interval 95%.

The test for subgroup differences across the continents yielded a $Q(3) =$ of 3.07, $p = 0.380$, suggesting that the variations in bruxism prevalence between the continents are not statistically significant, despite the apparent differences in point estimates. This indicates that while there are numerical differences in the reported prevalence rates, these differences could be attributed to the high heterogeneity within each subgroup rather than true differences in prevalence across continents.

The forest plot illustrating the overall effect of the studies on sleep bruxism across continents can be reviewed in the Supplementary Material S10.

1.5 Meta-analysis of global sleep bruxism prevalence among females

The meta-analysis conducted on the global prevalence of sleep bruxism among females encompasses a substantial dataset, including 78 studies (one study was excluded as an influential) and a total of 305,145 observations, from which 28,165 events of sleep bruxism were reported. The results derived from this comprehensive analysis, which employed a random effects model, suggest a central estimate of sleep bruxism prevalence at 11.68%, with a 95% confidence interval ranging from 9.07% to 14.07%. This prevalence rate indicates that sleep bruxism is a relatively common condition among females globally, albeit with significant variability.

The prediction interval, ranging from 1.94% to 47.00%, is notably wide, signaling that the true prevalence of sleep bruxism among different female populations could vary dramatically.

The quantification of heterogeneity in this analysis reveals extremely high values. The τ^2 =0.90, with τ = 0.95. These high values point to significant differences in the outcomes of the studies included in this meta-analysis. Furthermore, the I^2 = 99.3%, (*CI 95*: 99.3% – 99.4%). This is corroborated by an H = 12.01 (*CI 95%*: 11.59 – 12.54), far exceeding the value of 1, which would indicate homogeneity. These metrics confirm that the variance seen in the prevalence estimates is overwhelmingly due to true diversity among the study findings, rather than random error.

The test of heterogeneity, assessed through both the Wald and LRT, provides a $p < 0.001$, confirming the presence of significant heterogeneity among the studies. This result underscores the need for caution in interpreting the pooled prevalence estimate, as the underlying studies are highly diverse in nature.

The forest plot depicting the impact of various studies on sleep bruxism prevalence among females can be accessed in the document labeled Supplementary Material S10.

The leave-one-out diagnostic analysis identified one study, "Tay, 2020," as influential. Consequently, this study was excluded from the pooled effect estimation to ensure the robustness and reliability of our analysis.

The visualization of publication bias using a funnel plot is available in the Supplementary Material S11.

The Egger linear regression test produced a t (76) = 1.57, accompanying a p = 0.120. indicating no statistically significant evidence of asymmetry within the funnel plot. This outcome suggests that the meta-analysis is relatively free from publication bias, implying that the effect sizes reported by smaller studies do not systematically differ from those reported by larger studies.

Delving deeper into the regression coefficients provides further clarity. The bias coefficient is calculated at 2.86 with a SE = 1.82. Although this coefficient is positive–typically indicating a potential overestimation of effect sizes in smaller studies–the associated large standard error and the nonsignificant p-value largely diminish concerns regarding its impact on the overall analysis.

Additionally, the intercept of the regression, estimated at β_0 = -2.24 with a notably precise SE = 0.10, suggests robustness in the central tendency of the effect sizes. The small standard error of the intercept reinforces the stability and reliability of this estimate, supporting the conclusion that the reported prevalence rates are consistent and unbiased by the size of the studies included.

1.6 Meta-analysis of global sleep bruxism prevalence among males

In the meta-analysis exploring the global prevalence of sleep bruxism among males, a comprehensive aggregation of data from 75 studies (from which 1 study was eliminated as influential) encompassing 300,283 observations and 27,243 events was conducted. The results from this analysis, utilizing a random effects model, indicate a pooled prevalence rate of 8.48%, with a CI 95% 7.25% to 9.89%. This suggests that while sleep bruxism is a notable health concern among males globally, the prevalence is somewhat moderate.

The prediction interval, spanning from 2.11% to 28.47%, reveals a substantial variance in sleep bruxism rates across different populations or study settings. Such a broad interval underscores the influence of diverse factors–possibly including genetic, behavioral, and environmental elements–on sleep bruxism prevalence among different male demographics. The wide range necessitates a cautious interpretation of the pooled prevalence rate, as the actual prevalence in any given population could vary significantly from the central estimate.

Further insights are gained from the measures of heterogeneity within the meta-analysis. The τ^2 = 0.53, and the τ = 0.73, indicating a significant variance between the studies beyond what would be expected by chance alone. The I^2 = 99.3%, points to almost all the variance across studies being due to heterogeneity rather than sampling error. This is further corroborated by the H = 11.65, significantly exceeds the threshold of 1, confirming substantial inconsistency across the included studies.

The tests of heterogeneity, including the Wald and likelihood ratio test (LRT), report significant $p < 0.001$, affirming the presence of considerable heterogeneity among the studies analyzed. This finding is critical as it suggests that the differences in study outcomes are not random but rather reflect variations in how sleep bruxism is studied or reported across different contexts or populations.

The forest plot depicting the impact of various studies on sleep bruxism prevalence among males can be accessed in the Supplementary Material S10.

The leave-one-out diagnostic analysis identified one study, "Shahbour, 2022," as influential. Consequently, this study was excluded from the pooled effect estimation to ensure the robustness and reliability of our analysis.

The visualization of publication bias using a funnel plot is available in the Supplementary Material S11.

The Egger linear regression test of funnel plot asymmetry for the meta-analysis on sleep bruxism prevalence among males does not indicate significant publication bias. The test results, with a $t = -0.74$ and a $p = 0.462$, confirm that there is no statistically significant asymmetry in the funnel plot, suggesting an absence of publication bias at a conventional level of significance. This outcome is crucial as it supports the notion that the findings of the meta-analysis are not unduly influenced by the non-publication of small studies with negative or null results.

Despite the lack of publication bias, the analysis reveals other complexities. The negative bias coefficient of -1.30, although not statistically significant given its $SE = 1.75$, hints at a potential underrepresentation of smaller studies with lower effect sizes. However, the non-significant nature of this result, combined with the large standard error, diminishes the concern that this could meaningfully skew the overall analysis.

The intercept of the regression, at $\beta_0 = -2.10$ with a very small $SE = 0.10$, further indicates the robustness of the meta-analysis in capturing the central tendency of the effect sizes, despite the high heterogeneity. This suggests that the central estimate of the prevalence of sleep bruxism among males is reliable and provides a solid foundation for understanding the condition's impact at a global level.

1.7 Meta-analysis of global sleep bruxism prevalence by age (adults vs. minors)

The meta-analysis conducted to compare the global prevalence of sleep bruxism among adults and minors has provided a comprehensive insight into the age-related dynamics of this condition. A total of 144 studies encompassing 353,994 participants were analyzed, revealing a pooled prevalence rate of 20.99% under a random effects model, with the 95% confidence interval (CI) ranging from 18.69% to 23.50%.

The prediction interval, extending from 4.47% to 60.17%, indicates an enormously wide range of possible true prevalence rates across different settings or populations.

The heterogeneity metrics further elaborate the complexity of the data analyzed. With a $\tau^2 = 0.77$ and $I^2 = 99.3\%$, there is an exceedingly high level of variability among the study results, which is far beyond what would be expected by chance. This is supported by an $H = 12.34$, significantly greater than 1.0, indicating substantial inconsistency across studies.

The meta-analysis of global sleep bruxism prevalence, segregated by age into adults and minors in table 3, provides a nuanced view of how this condition varies across different life stages. By analyzing 80 studies involving adults and 64 studies involving minors, the research indicates a slightly higher prevalence of sleep bruxism among adults at 23% compared to 19% among minors, with both groups showing a substantial amount of variation as indicated by their respective CI 95% of 19% to 26% for adults and 16% to 23% for minors.

Despite the apparent difference in prevalence rates between adults and minors, the overlapping confidence intervals indicate that these differences might not be as pronounced as the point estimates suggest. This implies that while age may play a role in the prevalence of sleep bruxism, the effect is not sufficiently distinct to consider it entirely separate between the two groups.

Table 3. Variability in sleep bruxism prevalence: a subgroup analysis by age

age	*k*	*proportion*	*CI 95%*	τ^2	*Q*	I^2
Adults	80	0.23	0.19 – 0.26	0.78	8215.8	99.0%
Minors	64	0.19	0.16 – 0.23	0.72	9381.0	99.3%

Despite the apparent differences in prevalence rates between adults and minors, the test for subgroup differences, with a $Q = 2.02$, $p = 0.155$, indicates that these differences are not statistically significant. This suggests that while there may be a tendency for adults to exhibit a slightly higher

prevalence of sleep bruxism compared to minors, these differences are not robust enough to reach conventional levels of statistical significance.

The forest plot illustrating the overall effect of the studies on sleep bruxism by age can be reviewed in the file Supplementary Material S10.

1.8 Meta-analysis of global sleep bruxism prevalence by age and continent

The meta-analysis examining the global prevalence of sleep bruxism by age and continent includes a comprehensive aggregation of data from 140 studies encompassing 351, 351 observations and 64,216 events was conducted.

The overall prevalence rate derived from a random effects model stands at 21.48%, with a 95% confidence interval of 19.18% to 23.98%, indicating moderate certainty about the global average. A significant range in the prediction interval from 4.85% to 59.46% suggests substantial variation in sleep bruxism prevalence across various settings and populations.

The heterogeneity within the meta-analysis is exceptionally high, as indicated by an $I^2 = 99.4\%$, alongside a $\tau^2 = 0.72$ and $\tau = 0.85$. These values underscore the vast differences in study outcomes, which may be attributable to methodological disparities, demographic variations, or different diagnostic criteria used across studies. The heterogeneity is further confirmed by the Q test (Wald = 21506.21, LRT = 23658.28) both with a $p < 0.001$, suggesting that the variability among the studies is statistically significant and not due to chance.

Subgroup by continent and age group analysis reported a in table 4 reveals nuanced insights. For instance, the prevalence in European adults is notably higher at 22.55% compared to European minors at 16.17%, with a relatively lower but still substantial heterogeneity in European adults ($\tau^2 = 0.47$, $I^2 = 99.0\%$). North American data show higher prevalence rates, particularly among adults at 36.39%, though this subgroup has a notably wide confidence interval, reflecting high uncertainty possibly due to the small number of studies (k = 2). This is supported by a very high $\tau^2 = 3.32$, indicating extreme variability.

In South America and Asia, the prevalence rates tend to be lower among minors compared to adults, with Asian minors showing the lowest prevalence at 14.40%. The heterogeneity remains very high across all subgroups, suggesting that even within continents and age groups, there are significant differences in study outcomes.

Table 4. Variability in sleep bruxism prevalence: a subgroup analysis by age

continent	*age*	*k*	*proportion*	*CI 95%*	τ^2	Q	I^2
Europe	Minors	6	0.16	0.10 – 0.25	0.44	260.30	98.1%
Europe	Adults	29	0.23	0.18 – 0.27	0.47	2803.02	99.0%
North America	Minors	3	0.28	0.16 – 0.45	0.38	147.98	98.6%
North America	Adults	2	0.36	0.04 – 0.88	3.31	102.31	99.0%
South America	Minors	34	0.24	0.20 – 0.28	0.48	1202.59	97.3%
South America	Adults	20	0.23	0.16 – 0.32	1.18	1579.70	98.8%
Asia	Minors	19	0.14	0.10 – 0.20	0.78	2527.53	99.3%
Asia	Adults	27	0.23	0.18 – 0.28	0.54	2612.05	99.0%

k – number of studies; *CI 95%* – confidence interval 95%.

The test for subgroup differences across continent-age categories shows a $Q(7) = 10.04$, $p = 0.187$, suggesting that while there are differences in prevalence rates across different subgroups, these differences are not statistically significant. This could imply that factors other than just continent and age might be influencing the prevalence rates, such as cultural differences, healthcare access, and diagnostic criteria.

The forest plot illustrating the overall effect of the studies on sleep bruxism by continent and age can be reviewed in the file Supplementary Material S10.

1.9 Meta-analysis of sleep bruxism prevalence among females by age and continent

The meta-analysis focused on the prevalence of sleep bruxism among females across different age groups and continents includes a comprehensive aggregation of data from 75 studies, from which 1 study, (Tay, 2020) was initially eliminated as influential, encompassing 302, 502 observations and 27,913 events was conducted. The meta-analysis reports a global prevalence of 12.00% according to a random effects model, with a confidence interval of 9.87% to 14.50%. This result, however, masks significant heterogeneity across studies, as evidenced by an $I^2 = 99.3\%$ and a $\tau^2 = 0.90$. The very wide prediction interval ranging from 1.99% to 47.79% underscores the considerable variability in prevalence rates across different populations and studies.

The heterogeneity in the data is further highlighted by the *Q* tests for heterogeneity (Wald = 11043.05 and LRT = 10860.50), both yielding $p < 0.001$, indicating that the differences observed across

studies are statistically significant and not due to random variation. This suggests that factors beyond simple random chance are influencing the observed rates of sleep bruxism.

Based in results in table 5, breaking down the results by continent and age, the prevalence of sleep bruxism among European adults is relatively higher at 14.66%, with a confidence interval from 10.66% to 19.81%. This group also exhibits high heterogeneity (τ^2 = 0.57, I^2 = 99.3%). In contrast, European minors show a much lower prevalence of 5.31%, which although lower, still presents considerable variability (I^2 = 95.1%).

In North America, due to the presence of only one study per subgroup, heterogeneity could not be calculated. However, the prevalence rates are notably different with minors at 15.14% and adults at a strikingly high 78.26%. The latter number, although based on a single study, suggests a potentially significant geographic or methodological influence on reported prevalence rates.

South American results show a more consistent pattern between minors and adults, with prevalence rates of 11.21% and 14.69% respectively. However, both subgroups show substantial heterogeneity, especially among adults (τ^2 = 1.49, I^2 = 98.8%), indicating significant variation within this continental cohort.

Asian subgroups show lower prevalence rates, with minors at 6.83% and adults at 13.29%, and both subgroups exhibit high heterogeneity (minors τ^2 = 0.65, I^2 = 98.1% and adults τ^2 = 0.49, I^2 = 98.3%).

Table 5. Variability in sleep bruxism prevalence among females: a subgroup analysis by age

continent	*age*	*k*	*proportion*	*CI 95%*	τ^2	*Q*	I^2
Europe	Minors	3	0.05	0.04 – 0.08	0.10	40.50	95.1%
Europe	Adults	17	0.15	0.11 – 0.20	0.57		99.3%
North America	Minors	1	0.15	0.14 – 0.16	-	0.00	-
North America	Adults	1	0.78	0.64 – 0.88	-	0.00	-
South America	Minors	18	0.11	0.08 – 0.15	0.59	494.78	96.6%
South America	Adults	10	0.15	0.07 – 0.27	1.49	779.61	98.8%
Asia	Minors	11	0.07	0.04 – 0.11	0.65	520.43	98.1%
Asia	Adults	13	0.13	0.09 – 0.18	0.49	707.96	98.3%

The test for subgroup differences across continent and age categories reveals a Q (7) = 121.17, $p < 0.001$, indicating significant differences in the prevalence of sleep bruxism among different demographic and geographic groups, probably through small group sizes.

The forest plot illustrating the overall effect of the studies on sleep bruxism by continent and age among females can be reviewed in the file Supplementary Material S10.

The leave-one-out diagnostic analysis identified one study, "Tay, 2020," as influential. Consequently, this study was excluded from the pooled effect estimation to ensure the robustness and reliability of our analysis.

1.10 Meta-analysis of sleep bruxism prevalence among males by age and continent

The meta-analysis, which investigated the prevalence of sleep bruxism among males across various age groups and continents, meticulously compiled data from 71 studies. Notably, one study–Nakata (2007)–was initially excluded due to its influential nature. This extensive analysis encompassed 297,640 observations and identified 27,065 events.

The findings of the meta-analysis revealed a global prevalence of sleep bruxism among males of 8.29%, as determined under a random effects model. The associated confidence interval ranged from 7.14% to 9.60%, which is significantly lower compared to that observed in females, which stands at 12.00% with a confidence interval of 9.87% to 14.50%.

A substantial range in the prediction interval from 2.30% to 25.76% indicates a significant variance in the prevalence rates across different studies, suggestive of diverse underlying influences.

The heterogeneity quantified in this analysis is notably high with an $I^2 = 99.1\%$, $\tau^2 = 0.45$, and an $H = 10.47$. Both the Wald (7564.28) and LRT (8060.22) tests for heterogeneity return a $p < 0.001$, confirming that the variance among the studies is not a product of chance but likely due to different factors impacting the prevalence rates across regions and ages.

The data presented in Table 6 underscores significant geographical and age-related disparities in the prevalence of sleep bruxism among males. In Europe, the prevalence rates for minors and adults are relatively low at 6% and 8% respectively, yet both groups exhibit extremely high heterogeneity, as indicated by I^2 values exceeding 95%. This suggests that the factors contributing to sleep bruxism in these populations might vary widely across different studies or demographic subsets.

North America presents a stark contrast, particularly in the minors subgroup, where the prevalence is notably higher at 27%. However, this figure is derived from a single study, which precludes any assessment of heterogeneity or broader generalizability to the North American minor population as a whole.

In South America, the prevalence among minors is higher than in adults, at 11% compared to 8%. Both age groups show significant heterogeneity, with slightly more variability observed among minors. This could point to age-specific factors or varying research methodologies that impact the reported prevalence.

Asian populations also show substantial heterogeneity with almost identical rates of prevalence in minors and adults, both at around 7%. The exceptionally high tau-squared values, particularly in minors, highlight substantial inter-study variability, suggesting that cultural, methodological, or demographic differences might be influencing these outcomes.

Table 6. Variability in sleep bruxism prevalence among males: a subgroup analysis by age

continent	*age*	*k*	*proportion*	*CI 95%*	τ^2	*Q*	I^2
Europe	Minors	3	0.06	0.03 – 0.20	0.20	48.36	95.9%
Europe	Adults	17	0.08	0.06 -0.10	0.27	479.31	96.7%
North America	Minors	1	0.27	0.26 – 0.29	-	-	-
North America	Adults	-	-	-	-	-	-
South America	Minors	17	0.11	0.08 – 0.14	0.40	447.38	96.4%
South America	Adults	9	0.08	0.06 – 0.11	0.23	69.33	88.5%
Asia	Minors	11	0.07	0.04 – 0.10	0.64	564.64	98.2%
Asia	Adults	12	0.07	0.05 – 0.10	0.39	453.79	97.6%

The test for subgroup differences yields a Q = 339.82 with 6 degrees of freedom, accompanied by a p-< 0.001. This result indicates that the variations in prevalence rates across different demographic and geographic subgroups are statistically significant. The notably high prevalence rate within the North American minors subgroup, which is considerably greater than those observed in other groups, likely contributes to these differences. However, the impact of this subgroup on the overall analysis should be interpreted with caution due to its small size.

The forest plot illustrating the overall effect of the studies on sleep bruxism by continent and age among males can be reviewed in the Supplementary Material S10.

The leave-one-out diagnostic analysis identified one study, "Nakata, 2007," as influential. Consequently, this study was excluded from the pooled effect estimation to ensure the robustness and reliability of our analysis.

1.11 Meta-analysis of global sleep bruxism prevalence among females by age

The meta-analysis examining global sleep bruxism prevalence among females by age shows significant findings and high levels of heterogeneity. A total of 78 studies were included with initially excluded Tay, 2020 study as influential, encompassing 305,145 observations and 28,165 events. The overall prevalence of sleep bruxism among females, as derived from a random effects model, stands at 11.68% with a 95% confidence interval ranging from 9.66% to 14.07%. Notably, the prediction interval, which spans from 1.94% to 47.00%, suggests a substantial variation in prevalence estimates across different populations and settings.

The quantification of heterogeneity within this meta-analysis is particularly pronounced. The $\tau^2 = 0.90$ and $\tau = 0.95$ indicate considerable variation, corroborated by an $I^2 = 99.3\%$, suggesting that 99.3% of the total variation across studies is due to heterogeneity rather than chance. This is further supported by an extremely high heterogeneity statistic $H = 12.01$, indicating the presence of substantial dispersion in effect estimates among the included studies.

Both the Wald test and the LRT for heterogeneity report extremely high Q values of 11112.81 and 10954.02, respectively, with 77 degrees of freedom and a $p < 0.001$, affirming the presence of significant heterogeneity among the studies.

When breaking down the data into subgroups of minors and adults, notable differences emerge. The prevalence among minors is 8.90%, with a narrower confidence interval of 6.96% to 11.30%, and among adults, it is higher at 14.49%, with a confidence interval of 11.14% to 18.64%. The heterogeneity within these subgroups remains high, with $\tau^2 = 0.62$ and 1.00, respectively, and both groups showing an $I^2 = 98\%$.

Furthermore, the test for subgroup differences reveals a $Q(1) = 7.23$ with $p = 0.007$, indicating statistically significant differences between the prevalence rates of minors and adults. This suggests that age is a significant factor influencing the prevalence of sleep bruxism among females.

The forest plot illustrating the overall effect of the studies on sleep bruxism among females by age can be reviewed in the Supplementary Material S10.

1.12 Meta-analysis of global sleep bruxism prevalence among males by age

The meta-analysis of global sleep bruxism prevalence among males by age, encompassing 74 studies (with initially excluded Shahbour, 2022 study as influential) with 300,283 observations and 27,243 events, indicates that the overall prevalence of sleep bruxism among males is approximately 8.48%. This finding is supported by a 95% confidence interval of 7.25% to 9.89%. Additionally, the prediction interval ranging from 2.11% to 28.47% suggests a considerable degree of variability in prevalence rates across different settings and populations.

The analysis reveals extremely high heterogeneity, characterized by $\tau^2 = 0.53$ and an $I^2 = 99.3\%$, indicating that nearly all the variability in prevalence estimates across studies is due to heterogeneity rather than chance. This high level of heterogeneity is further substantiated by an $H = 11.65$, which highlights substantial differences in the effect sizes among the included studies.

Both the Wald and the LRT for heterogeneity confirm these observations, with $Q = 9910.63$ and 10276.97 respectively, and a $p < 0.001$, strongly indicating significant heterogeneity across the studies.

When dissecting the data into age subgroups, the prevalence in minors is slightly higher at 8.96% with a 95% confidence interval of 7.00% to 11.40%, compared to 8.11% among adults, whose confidence interval ranges from 6.64% to 9.88%. The heterogeneity within these subgroups is notably high, with $\tau^2 = 0.59$ for minors and 0.47 for adults, and $I^2 = 99.4\%$ and 98.7% respectively.

The test for subgroup differences presents a $Q = 0.38$ with one degree of freedom and a $p =$ 0.536, indicating that the differences in prevalence between minors and adults are not statistically significant. This suggests that age, within this data set, does not play a major role in differentiating the prevalence of sleep bruxism among males.

The forest plot illustrating the overall effect of the studies on sleep bruxism by age among males can be reviewed in the Supplementary Material S10.

1.13 Meta-analysis of global awake bruxism prevalence

The meta-analysis focused on the global prevalence of awake bruxism, incorporating data from 59 studies with 42,388 observations and 10,091 reported events. The results from a random effects model reveal a mean prevalence rate of 23.29%, with a 95% confidence interval ranging from 18.78% to 28.52%. This indicates that nearly one in four individuals may experience awake bruxism, a condition characterized by involuntary teeth grinding or jaw clenching while awake.

The prediction interval, spanning from 3.49% to 71.86%, underscores an extensive variability in prevalence rates across different populations and study conditions. This wide range suggests that factors such as geographic location, cultural differences, diagnostic criteria, and study methodologies may significantly influence the reported rates of awake bruxism.

The analysis demonstrates an extremely high level of heterogeneity among the studies, quantified by a $\tau^2 = 1.11$ and a $\tau = 1.05$, culminating in an $I^2 = 98.8\%$. This suggests that almost all the variability in prevalence rates across studies can be attributed to heterogeneity rather than sampling error. The $H = 9.01$ reinforces this interpretation, indicating that the average variance between the study results is about nine times greater than what would be expected by chance alone.

The tests for heterogeneity, including the Wald test and the LRT, provide Q = 4704.63 and 5953.13, respectively, both with 58 degrees of freedom and a p <0.001. These findings confirm the presence of significant heterogeneity, validating the need for cautious interpretation of the pooled prevalence rate.

The forest plot illustrating the overall effect of the studies on awake bruxism can be reviewed in the Supplementary Material S10.

The leave-one-out diagnostics conducted revealed that none of the studies were deemed influential within the context of our analysis.

The visualization of publication bias using a funnel plot is available in the the Supplementary Material S11.

The Egger linear regression test of funnel plot asymmetry, yields a t (57) = -1.27, p = 0.208. This p-value, being greater than the significance threshold of 0.05, indicates that there is no statistically significant evidence of publication bias in the dataset under analysis. This suggests that the funnel plot is relatively symmetric and that the meta-analysis results are unlikely to be substantially influenced by the selective publication of studies with more favorable outcomes.

The sample estimates from the test provide additional insights. The estimated bias is -2.74, with a SE = 2.15. This bias estimate, which is not significantly different from zero based on the p-value, suggests there isn't a consistent directional error that would indicate larger or smaller studies systematically reporting different results. The intercept of $\beta_0 = -0.74$ with a SE = 0.20, which is also part of the regression output, further supports the conclusion of minimal asymmetry in the plot.

1.14 Meta-analysis of global awake bruxism prevalence by age

In this meta-analysis assessing the global prevalence of awake bruxism, the pooled data from 59 studies, involving 42,388 observations and 10,091 events, reveal a prevalence rate of 23.29%, as indicated by a 95% confidence interval of 18.78% to 28.52%. The wide prediction interval, ranging from 3.49% to 71.86%, underscores the considerable variability in prevalence estimates across the included studies.

The heterogeneity metrics are remarkably high, with τ^2 = 1.11, tau at 1.05, and an I^2 = 98.8%, suggesting that almost all the variability in prevalence rates is due to differences between studies rather than chance. Such high heterogeneity is confirmed by both the Wald and LRT, with significant Q values indicating substantial differences in study outcomes.

Diving deeper into the age-specific data, the prevalence of awake bruxism is reported to be higher in adults at 24.26%, with a confidence interval between 19.35% and 29.96%. In contrast, minors exhibit a lower prevalence at 19.48%, though with a wider confidence interval from 10.51% to 33.26%. The heterogeneity remains high within these subgroups, with τ^2 =of 1.01 for adults and 1.4776 for minors, and I^2 = 98.6% and 99.1%, respectively. This indicates that, within each age group, the variability in prevalence estimates is still primarily driven by factors other than sampling variability.

The test for subgroup differences yields a Q = 0.50 with one degree of freedom and a p = 0.480, suggesting no statistically significant difference in the prevalence of awake bruxism between adults and minors. This finding indicates that age may not be a significant factor in influencing the prevalence of awake bruxism, at least within the context of this analysis.

The forest plot illustrating the overall effect of the studies on sleep bruxism by age can be reviewed in the file Supplementary Material S10.

1.15 Meta-analysis of global awake bruxism prevalence among females

The presented meta-analysis focuses on the prevalence of awake bruxism among females, incorporating data from 26 studies totaling 23,943 observations, with 4,476 events of awake bruxism reported. The findings from this specific subset reveal a pooled prevalence estimate of 17.07%, as indicated by the random effects model with a 95% confidence interval ranging from 12.39% to 23.05%. This prevalence suggests that awake bruxism is a significant concern among females, though the variation is notable.

The prediction interval for this analysis stretches from 2.63% to 61.04%, illustrating a substantial spread in the prevalence estimates across different studies.

Regarding heterogeneity, the analysis shows extremely high levels with a $\tau^2 = 0.93$ and a $\tau = 0.96$. The I^2 statistic stands at 98.3%, indicating that nearly all of the variability in the prevalence rates across the included studies is due to heterogeneity rather than chance. This is further supported by the $H = 7.66$, suggests that the true effect sizes vary considerably across studies.

The tests for heterogeneity, including the Wald and LRT, provide further confirmation of significant variability among the studies. The Wald test results in a Q (25) = 1467.34, $p < 0.001$, and the LRT shows a Q =1875.09, $p < 0.001$, both underscoring the presence of significant heterogeneity within the data.

The forest plot illustrating the overall effect of the studies on awake bruxism among females can be reviewed in the file Supplementary Material S10.

The leave-one-out diagnostics conducted revealed that none of the studies were deemed influential within the context of our analysis.

The visualization of publication bias using a funnel plot is available in the Supplementary Material S11.

The results of the Egger's test in this context show a t (24) = -1.21, $p = 0.237$. This p-value, being significantly above the conventional alpha level, suggests that there is no statistically significant evidence of publication bias within the meta-analysis. This implies that the funnel plot, which visualizes the relationship between study sizes and their effect estimates, is symmetric enough to suggest an unbiased dissemination of research findings across the spectrum of study sizes included in the analysis.

In terms of specific estimates provided by the test, the reported bias is -3.24 with a SE = 2.67. Although this suggests a tendency towards a negative bias, the lack of statistical significance (as indicated by the p-value) means that this observation does not provide strong evidence of systematic skewness in the reporting of study results. The intercept of $\beta_0 = -1.06$, with its $SE = 0.23$, further supports the conclusion that there is no substantial asymmetry in the funnel plot. The intercept measures the extent of asymmetry at the level of largest studies (i.e., those with the smallest standard error); a non-significant intercept enhances the credibility of the meta-analysis, indicating that the largest, most precise studies are not biased in a way that would distort the overall analysis.

1.16 Meta-analysis of global awake bruxism prevalence among males

The meta-analysis concerning global awake bruxism prevalence among males, encompassing data from 26 studies with a total of 23,943 observations and 2,269 recorded events of bruxism, presents a pooled prevalence of 8.33%, as per the random effects model. The 95% confidence interval for this estimate ranges from 6.33% to 10.89%, indicating a moderate level of prevalence but with considerable uncertainty about the precise rate.

The prediction interval, spanning from 1.86% to 30.31%, is notably wide. This broad range suggests there is substantial variability in the bruxism rates reported by individual studies, potentially reflecting diverse population characteristics, diagnostic criteria, or cultural influences.

Quantifying the heterogeneity in this analysis, the τ^2 = 0.55 with a corresponding tau of 0.74. The I^2 = 96.7%, with a 95% confidence interval from 95.9% to 97.3%. This high I^2 value suggests that the vast majority of the variability observed between the studies' results is due to true differences in study conditions rather than random chance. The heterogeneity measure *H*, with a value of 5.48 (CI: 4.94 to 6.09), further supports the presence of substantial variation between the studies, indicating that the effect sizes are considerably diverse.

The tests for heterogeneity, both the LRT, confirm significant differences in study outcomes with $p < 0.001$. These results underscore the substantial heterogeneity among the included studies, suggesting that factors such as methodological diversity, geographic and demographic variations, or differences in how bruxism is assessed and reported are influencing the overall prevalence estimates.

The forest plot illustrating the overall effect of the studies on awake among males bruxism can be reviewed in the file Supplementary Material S10.

The leave-one-out diagnostics conducted revealed that none of the studies were deemed influential within the context of our analysis.

The visualization of publication bias using a funnel plot is available in the Supplementary Material S11.

The Egger linear regression test of funnel plot asymmetry is employed in this meta-analysis to evaluate the presence of publication bias. In the context of assessing the prevalence of awake bruxism among males, the results from the Egger's test yield a t (24) = -1.55, accompanying a p = 0.133. This p-value, which falls above the conventional significance threshold, indicates that there is no statistically significant evidence of publication bias in the dataset. This suggests that the funnel plot, a graphical tool

used to assess such biases, is reasonably symmetric. A symmetric funnel plot implies that the smaller studies in the meta-analysis do not show a systematic tendency to report higher or lower prevalence rates compared to larger studies, which is crucial for affirming the reliability of the meta-analysis results.

The reported bias is -2.98 with a SE = 1.92. Although this points to a potential minor skew in the data, the nonsignificant t-test result negates a conclusive assertion of bias presence. Additionally, the intercept value being $\beta 0$ = -1.79 with a SE = 0.22 indicates the point of asymmetry at the largest studies–those with the smallest standard errors. The fact that this intercept is not significantly different from zero reinforces the conclusion that there is no substantial publication bias affecting the meta-analysis.

1.17 Meta-analysis of awake bruxism prevalence by continent

The meta-analysis examining the prevalence of awake bruxism by continent incorporates data from 56 studies, with 40,745 observations and 9,541 events, revealing substantial variation in awake bruxism prevalence across different global regions. The overall pooled prevalence estimate from the random effects model is 23.08%, with a 95% confidence interval ranging from 18.47% to 28.44%. This indicates a moderately high general prevalence, but the accompanying prediction interval, which stretches from 3.41% to 71.83%, suggests extreme variability in bruxism prevalence across different settings and studies.

This variability is further quantified through heterogeneity metrics, where τ^2 = 1.12 and τ = 1.06, and the I^2 = 98.8%. Such an I^2 value indicates that nearly all the variability in prevalence estimates is due to true differences between studies rather than random chance. The H = 8.97 confirms these findings, suggesting a very high level of inconsistency among the study results.

The tests for heterogeneity – both the Wald and the LRT – yield $p < 0.001$, underscoring the presence of significant heterogeneity. This confirms that the studies included in the meta-analysis are not estimating the same underlying effect size, but rather a range of effect sizes influenced by various factors.

The prevalence rates broken down by continent shown in table 7. Europe shows the lowest bruxism prevalence at 17.90%, with a very narrow confidence interval but still substantial heterogeneity (I^2 = 99.0%). South America exhibits the highest prevalence at 29.86%, albeit with a wider confidence interval, reflecting more variability in the estimates and slightly less, yet still very high, heterogeneity (I^2 = 97.8%). Asia has a prevalence rate of 24.91%, with associated confidence intervals and heterogeneity metrics similar to those of Europe.

Table 7. Variability in awake bruxism prevalence: a subgroup analysis by continent

continent	*k*	*proportion*	*CI 95%*	τ^2	*Q*	I^2
Europe	22	0.18	0.13 – 0.25	0.88	2034.28	99.0%
South America	15	0.30	0.19 – 0.44	1.44	648.92	97.8%
Asia	19	0.25	0.18 – 0.34	0.91	1161.37	98.5%

Note: k – number of studies; *CI 95%* – confidence interval 95%.

Despite these differences, the test for subgroup differences across continents ($Q = 3.84$, $df = 2$, $p = 0.147$) indicates that these variations are not statistically significant. This suggests that while numerically different, the variability within each continent's estimates might be influencing the perception of between-continent differences.

The forest plot illustrating the overall effect of the studies on awake bruxism can be reviewed in the file Supplementary Material S10.

1.18 Meta-analysis of global awake bruxism prevalence among females by age

The meta-analysis under discussion provides a comprehensive assessment of the global prevalence of awake bruxism among females, segmented by age into minors and adults. Analyzing data from 26 studies encompassing 23,943 observations, the meta-analysis suggests that the overall estimated prevalence of awake bruxism in females is 17.07% according to a random effects model. This model also yields a notably wide prediction interval ranging from 2.63% to 61.04%, highlighting substantial variability in prevalence estimates across different studies.

This variability is further quantified through statistical measures of heterogeneity, which are exceptionally high. The $I^2 = 98.3\%$ indicates that a vast majority of the variability in prevalence estimates is likely due to genuine differences in study conditions rather than chance. Such a high value of I^2 is corroborated by the τ^2 and τ values of 0.93 and 0.96, respectively, and an $H = 7.66$, which confirms the significant dispersion of effect sizes among the included studies.

The heterogeneity tests, namely the Wald and LRT, both return p-values effectively at zero, further validating the presence of significant heterogeneity among the studies analyzed. This underscores the complexity and diversity of factors influencing the prevalence of awake bruxism, which may include diagnostic criteria, population demographics, and methodological differences.

When examining the results by age subgroups, the prevalence in minors is estimated at 11.28%, with a CI 95% stretching from 5.19% to 22.79%. Adults, however, exhibit a higher prevalence rate of

18.35%, with a *CI 95%* 13.02% to 25.21%. Despite the apparent difference in prevalence rates, the test for subgroup differences, which yields a Q =1.43 with p = 0.233, indicates that these differences are not statistically significant. This suggests that while numerically different, the prevalence rates between minors and adults do not diverge sufficiently when accounting for the high levels of variability within each subgroup.

The forest plot illustrating the overall effect of the studies on awake bruxism can be reviewed in the file Supplementary Material S10.

1.19 Meta-analysis of global awake bruxism prevalence among males by age

The meta-analysis investigating the global prevalence of awake bruxism among males, segmented by age, incorporates data from 26 studies with a total of 23,943 observations and 2,269 events of bruxism. The synthesized findings via a random effects model suggest an overall prevalence of 8.33%, with the *CI 95%* ranging from 6.33% to 10.89%. The prediction interval, however, extends from 1.86% to 30.31%, indicating a significant variance in prevalence estimates across different studies, similar to the trends observed in male groups.

The heterogeneity within these studies is substantial, as reflected by an I^2 = 96.7%. This high degree of heterogeneity is indicative of varying study conditions, methodologies, and demographic factors across the included studies. The τ^2 = 0.55 with a τ = 0.74, and an H = 5.48, all of which underscore the considerable dispersion of effect sizes among the studies.

Both the Wald and LRT for heterogeneity confirm this observation with $p < 0.001$, affirming that the variability among the study results is unlikely to be due to chance. This suggests that factors unique to each study, possibly including geographic location, diagnostic criteria, and age distribution, significantly impact the reported prevalence rates.

By brokering down into age-specific subgroups, the results reveal that minors have a lower estimated prevalence of awake bruxism at 5.62%, with a confidence interval from 2.31% to 13.05%. Adults, on the other hand, show a higher prevalence of 8.91%, with a confidence interval ranging from 6.75% to 11.69%. Despite these numerical differences, the test for subgroup differences yields a Q = 1.00 with p = 0.317, indicating that these differences are not statistically significant.

This suggests that while there appears to be a trend towards higher prevalence in adults, the data does not strongly differentiate between the two age groups in terms of statistical significance, likely due to the high levels of variability and heterogeneity within each subgroup.

The forest plot illustrating the overall effect of the studies on awake bruxism can be reviewed in the file Supplementary Material S10.

1.20 Meta-analysis of awake bruxism prevalence among females by continent

In the current meta-analysis focused on the prevalence of awake bruxism among females by continent, data from 23 studies encompassing 22,300 observations revealed an overall prevalence of 17.50% using a random effects model. The confidence interval for this estimate is relatively wide, ranging from 12.51% to 23.94%, and the prediction interval extends from 2.72% to 61.67%, indicating significant variability in the prevalence rates across different populations and studies.

The heterogeneity in this analysis is remarkably high, as evidenced by an $I^2 = 98.4\%$. This value suggests that almost all the variability in prevalence estimates is due to true differences in study characteristics rather than random chance. This interpretation is supported by the $\tau^2 = 0.91$ and the $\tau = 0.95$, indicating substantial variability between the true effect sizes. The H statistic further confirms this substantial dispersion with a value of $H = 7.89$.

The tests for heterogeneity, including both the Wald and LRT, show extremely significant results with $p < 0.001$, reinforcing the presence of significant heterogeneity among the studies. This suggests that factors specific to each study or population, potentially including geographic, cultural, and methodological differences, play critical roles in the observed prevalence rates.

Breaking down the results by continent as shown in table 8, the estimated prevalence rates vary notably. Europe exhibits the lowest prevalence at 12.02%, with a *CI 95%* from 6.58% to 20.96%. South America shows a markedly higher prevalence at 26.45%, with a *CI 95%* from 17.23% to 38.31%. Asia also shows a higher prevalence than Europe at 22.48%, with a CI 95% from 16.72% to 29.52%. The τ^2 values decrease from Europe to South America and Asia, indicating decreasing levels of heterogeneity among studies within these continents, though all still display significant heterogeneity.

Table 8. Variability in awake bruxism prevalence: a subgroup analysis by continent among females

continent	*k*	*proportion*	*CI 95%*	τ^2	*Q*	I^2
Europe	11	0.12	0.07 – 0.21	1.23	924.73	98.9%
South America	5	0.26	0.17 – 0.38	0.35	72.64	94.5%
Asia	7	0.22	0.17 – 0.30	0.24	295.51	98.0%

Note: k – number of studies; *CI 95%* – confidence interval 95%.

The test for subgroup differences across continents yields a $Q = 5.25$ with a $p = 0.072$. This value suggests a trend towards significant differences in awake bruxism prevalence among continents, although it does not reach conventional levels of statistical significance α. This indicates that while there are apparent differences in the prevalence rates among continents, these differences might be influenced by the high heterogeneity within each subgroup.

The forest plot illustrating the overall effect of the studies on awake bruxism can be reviewed in the Supplementary Material S10.

1.21 Meta-analysis of awake bruxism prevalence among males by continent

The meta-analysis designed to assess the prevalence of awake bruxism among males across different continents included 23 studies, covering 22,300 participants and identifying 2,029 cases of bruxism. Employing a random effects model, the overall estimated prevalence is calculated at 8.08%, with a confidence interval ranging from 6.01% to 10.79%. Notably, the prediction interval spans a wide range from 1.75% to 30.22%, suggesting a significant variation in prevalence estimates that could be related to diverse population characteristics or methodological differences across studies.

The quantification of heterogeneity in this analysis is particularly notable, with an $I^2 = 96.5\%$, indicating that nearly all variability in prevalence estimates is attributable to genuine differences across studies rather than chance. This is further emphasized by a $\tau^2 = 0.56$ and a $\tau = 0.75$. The $H = 5.37$ supports this interpretation, indicating a substantial dispersion of effect sizes among the included studies.

The Wald and LRT for heterogeneity both yield extremely low p-values ($p < 0.001$), underscoring the significant heterogeneity across the studies. This suggests a strong influence of non-random factors, potentially cultural, and methodological variations.

As reported in table 9, when examining the results by continent, distinct differences emerge in the prevalence estimates. Europe reports the lowest prevalence at 5.38%, with a confidence interval from 3.44% to 8.32%. The $\tau^2 = 0.59$, indicating notable heterogeneity within this subgroup. South America

shows a higher prevalence at 9.71%, with a narrower confidence interval from 8.05% to 11.67% and an exceptionally low $\tau^2 = 0$, suggesting no heterogeneity within this subgroup. Asia presents the highest prevalence of 13.82%, with a confidence interval from 9.95% to 18.90%. Here too, substantial heterogeneity is evident with a tau $\tau^2 = 0.24$.

Table 9. Variability in awake bruxism prevalence: a subgroup analysis by continent among males

Continent	*k*	*proportion*	*CI 95%*	τ^2	*Q*	I^2
Europe	11	0.05	0.03 – 0.08	0.59	171.76	94.2%
South America	5	0.10	0.08 – 0.12	0.0	0.97	0.0%
Asia	7	0.14	0.10 – 0.19	0.24	118.25	94.9%

Note: k – number of studies; *CI 95%* – confidence interval 95%.

The test for subgroup differences across continents yields a Q value of 11.55, $p < 0.003$, indicating statistically significant differences in awake bruxism prevalence among continents.

The forest plot illustrating the overall effect of the studies on awake bruxism can be reviewed in the Supplementary Material S10.

1.22 Meta-analysis of awake bruxism prevalence by continent and age

This comprehensive meta-analysis, which pooled data from 56 studies comprising 40,745 observations and 9,541 events, aimed to elucidate the prevalence of awake bruxism by continent and age group. The analysis revealed an overall estimated prevalence of 23.08%, with a confidence interval ranging from 18.47% to 28.44%. The broad prediction interval from 3.41% to 71.83% underscores the highly variable nature of bruxism prevalence across different populations and studies.

The heterogeneity quantified in this meta-analysis is exceptionally high, with an $I^2 = 98.8\%$, indicating that nearly all the variability in the reported prevalence rates can be attributed to real differences in study characteristics rather than random variance. This is further corroborated by a $\tau^2 = 1.12$ and a $\tau = 1.06$, pointing to significant differences in the true effect sizes across the studies. The $H = 8.97$ confirms substantial dispersion in effect sizes among the included studies.

Both the Wald and LRT for heterogeneity yielded p-values of zero, highlighting the significant variability and confirming that this is not due to chance. These findings emphasize the complex nature of awake bruxism, influenced by a matrix of factors including geographic, demographic, and methodological variations.

The meta-analysis detailed in Table 10 offers a nuanced look at the prevalence of awake bruxism across different continents and age groups, revealing a complex pattern influenced by geographic and demographic factors. The results underscore the variation not only in prevalence rates but also in the degree of heterogeneity among the subgroups analyzed.

Starting with Europe, the contrast between minors and adults in terms of bruxism prevalence is striking. Minors show a notably lower prevalence at 6%, which, despite being based on just 2 studies, suggests a lesser degree of awake bruxism in younger populations within Europe. However, the high level of heterogeneity (I^2 = 92.5%) in this subgroup indicates that even within this narrow age range, factors contributing to bruxism are varied and possibly influenced by diverse cultural or environmental conditions. On the other hand, the adult population in Europe shows a higher prevalence at 20%, supported by a more substantial number of studies (20 in total). The even higher heterogeneity seen here (I^2 = 98.7%) could reflect a wider range of contributing factors such as stress, lifestyle, and possibly even differences in how bruxism is diagnosed and reported across European countries.

Moving to South America, the findings are intriguing due to the higher prevalence rates observed, especially in adults (33%). This region also exhibits significant variability, particularly among minors where the prevalence rate of 24% comes with a very broad confidence interval (0.08 to 0.54) and an extremely high heterogeneity (I^2 = 99.0%). This suggests that there are specific local factors, possibly including socio-economic and cultural influences, that might exacerbate or mitigate the incidence of bruxism. The adult group, while also showing substantial heterogeneity (I^2 = 95.9%), indicates a consistently higher prevalence across studies compared to minors, hinting at age-related factors that might influence the condition's manifestation.

Asia presents a more consistent picture between minors and adults, with prevalence rates of 22% and 25% respectively. The lower heterogeneity in minors (I^2 =95.9% with a lower τ^2 value) compared to adults (I^2 = 98.5%) could indicate more uniform diagnostic criteria or reporting practices within this demographic across the Asian studies. However, the high heterogeneity in adults suggests diverse underlying factors and possibly a range of responses to cultural, environmental, and biological influences.

Table 10. Variability in awake bruxism prevalence: a subgroup analysis by continent and age

continent	*age*	*k*	*proportion*	*CI 95%*	τ^2	*Q*	I^2
Europe	minors	2	0.06	0.04 – 0.10	0.14	13.37	92.5%
Europe	adults	20	0.20	0.14 – 0.27	0.79	1499.74	98.7%
South America	minors	5	0.24	0.08 – 0.54	2.23	412.04	99.0%

continent	*age*	*k*	*proportion*	*CI 95%*	τ^2	*Q*	I^2
South America	adults	10	0.33	0.21 – 0.48	0.95	220.62	95.9%
Asia	minors	3	0.22	0.18 – 0.28	0.06	49.06	95.9%
Asia	adults	16	0.25	0.17 – 0.36	1.09	987.94	98.5%

Note: k – number of studies; *CI 95%* – confidence interval 95%.

The test for subgroup differences yielded a $Q = 28.13$ with a $p < 0.001$, indicating statistically significant differences in awake bruxism prevalence across different continental and age subgroups. This variation could be influenced by a range of factors, including genetic predispositions, cultural differences in stress management, diagnostic criteria, and awareness of the condition.

The forest plot illustrating the overall effect of the studies on awake bruxism can be reviewed in the Supplementary Material S10.

1.23 Meta-analysis of awake bruxism prevalence by continent and age among females

The current meta-analysis focuses on the prevalence of awake bruxism among females across different continents and age groups, revealing both widespread and disparate patterns among 23 studies encompassing 22,300 observations and 4,173 events.

The overall pooled prevalence from the random effects model is 17.5%, with a notably wide prediction interval ranging from 2.72% to 61.67%. This wide prediction interval and the high heterogeneity ($I^2 = 98.4\%$) underscore the substantial variability in bruxism prevalence among females, which could be influenced by multiple factors including genetic predispositions, stress levels, cultural differences, and methodological variations across studies.

The quantified heterogeneity is profound, with $\tau^2 = 0.91$ and $\tau = 0.95$, suggesting that the differences between studies are not just random but reflect true variability in the underlying population prevalence rates. This is further supported by the very high $H = 7.89$, indicating that the average study estimate is about 8 times more variable than would be expected if the studies were homogenous.

The minors in Europe according to the subgroup analysis in table 11 provides deeper reports that have the lowest reported prevalence at 2.85%, based on a single study, suggesting potentially lower rates of awake bruxism or perhaps underreporting or less awareness of the condition in this demographic. The Europe adults show a prevalence of 13.73% with extremely high heterogeneity ($I^2 = 98.9\%$). This suggests that adult females in Europe may experience a range of influences affecting

bruxism prevalence, possibly related to lifestyle, stress, or healthcare access. Adults in South Africa report a higher prevalence at 26.45%. The lower heterogeneity (I^2 = 94.5%) compared to their European counterparts might indicate a more uniform set of factors influencing awake bruxism across the studies or more consistent diagnostic criteria. Minors in Asia show a prevalence of 14.74% with no heterogeneity (I^2 = 0.0%), suggesting a consistent reporting and perhaps a uniform environmental or genetic influence in this demographic. Lastly, the adults in Aisa also report a high prevalence of 26.65% with substantial heterogeneity (I^2 = 95.9%), indicating diverse factors influencing the condition much like in Europe.

Table 11. Variability in awake bruxism prevalence: a subgroup analysis by continent and age among females

continent	*age*	*k*	*proportion*	*CI 95%*	τ^2	*Q*	I^2
Europe	minors	1	0.03	0.02 – 0.05	-	0.00	-
Europe	adults	10	0.14	0.08 – 0.23	1.10	855.24	98.9%
South America	adults	5	0.26	0.17 – 0.38	0.35	72.64	94.5%
Asia	minors	2	0.15	0.14 – 0.16	0.00	0.73	0.0%
Asia	adults	5	0.27	0.20 – 0.34	0.16	98.54	95.9%

Note: k – number of studies; *CI 95%* – confidence interval 95%.

The test for subgroup differences confirms significant variability between these groups (Q = 72.17, $p < 0.001$), highlighting the influence of both geographic and age-related factors on awake bruxism prevalence among females. The notable differences in heterogeneity and prevalence rates between continents and age groups suggest that local environmental, cultural, and healthcare factors play critical roles in the manifestation and reporting of awake bruxism among females.

The forest plot illustrating the overall effect of the studies on awake bruxism can be reviewed in the Supplementary Material S10.

1.24 Meta-analysis of awake bruxism prevalence by continent and age among males

Current meta-analysis delves into the prevalence of awake bruxism among males, segmented by continent and age, with 23 studies encompassing 22,300 observations and 2029 events. The findings reveal an overall prevalence of 8.08%, as estimated by the random effects model, with a *CI 95%* of 6.01% to 10.79%. This prevalence is notably lower than what is typically observed in female cohorts in similar studies. The prediction interval, ranging from 1.75% to 30.22%, suggests substantial variation in prevalence rates across different populations and settings.

The heterogeneity metrics are particularly telling. With $\tau^2 = 0.56$ and $\tau = 0.75$, the variability between studies remains high, although not as pronounced as in some female cohorts. The $I^2 = 96.5\%$ confirms that a significant portion of the variability in effect estimates is due to heterogeneity rather than chance. This is further evidenced by the high $H = 5.37$, indicating that the average study estimate is over five times more variable than one would expect if the studies were homogeneous.

The results from Table 12 subgroup analysis offer a nuanced view of the prevalence of awake bruxism among males, differentiated by age and geography. The findings reveal distinct patterns that suggest a complex reciprocation of environmental, cultural, and possibly genetic factors.

In Europe, the prevalence among minors is notably low at just 1.25%. This could potentially be attributed to lower stress levels, differing social structures, or more conservative diagnostic standards within pediatric populations. In contrast, European adults exhibit a prevalence of 6.11%, accompanied by significant heterogeneity ($I^2 = 94.0\%$). Despite the relatively narrow confidence interval, the high degree of variability indicates that multiple factors distinctly influence awake bruxism within this demographic, suggesting that adult males in Europe, while generally exhibiting lower rates of bruxism compared to other groups, are affected by a diverse array of contributing factors.

Turning to South America, adults in this region show a higher prevalence rate of 9.71%, with an $I^2 = 0.0\%$, indicating no observed heterogeneity. This uniformity across studies might reflect consistent diagnostic criteria or shared environmental and cultural influences that affect the expression of awake bruxism in South American adult males.

In Asia, the situation is markedly different for minors and adults. Minors report a prevalence of 8.88%, but with a broad confidence interval ranging from 4.51% to 16.73% and high heterogeneity ($I^2 = 97.0\%$). This suggests that a variety of factors influence awake bruxism in this group, leading to significant variability in reported rates. Asian adults, however, show the highest prevalence at 16.50%, with a moderate level of heterogeneity ($I^2 = 91.4\%$). Such findings might indicate specific cultural, environmental, or genetic predispositions that elevate the likelihood of awake bruxism among adult males in this region.

Table 12. Variability in awake bruxism prevalence: a subgroup analysis by continent and age among males

continent	*age*	*k*	*proportion*	*CI 95%*	τ^2	Q	I^2
Europe	minors	1	0.01	0.01 – 0.03	-	0.00	-
Europe	adults	10	0.06	0.04 – 0.09	0.44	149.09	94.0%
South America	adults	5	0.10	0.08 – 0.12	0.00	0.97	0.0%

continent	*age*	*k*	*proportion*	*CI 95%*	τ^2	*Q*	I^2
Asia	minors	2	0.09	0.05 – 0.17	0.26	33.33	97.0%
Asia	adults	5	0.17	0.13 – 0.21	0.10	46.59	91.4%

Note: k – number of studies; *CI 95%* – confidence interval 95%.

The significant test for subgroup differences ($Q = 53.34$, $p < 0.001$) confirms that geographical and age-related factors play a crucial role in the prevalence of awake bruxism among males. These findings underscore the complexity of awake bruxism as a global health concern, influenced by a myriad of factors ranging from genetics and lifestyle to healthcare practices and cultural norms. The marked differences between continents and age groups highlight the need for targeted research and tailored interventions to effectively manage and mitigate awake bruxism in diverse male populations.

The forest plot illustrating the overall effect of the studies on awake bruxism can be reviewed in the file titled the Supplementary Material S10.

1.25 Meta-analysis of global prevalence of sleep bruxism as assessed by polysomnography

The present meta-analysis examines the global prevalence of sleep bruxism as assessed through polysomnography. It includes data from four studies, notably featuring two influential cases: Raphael (2015) and Maluly (2013). This comprehensive dataset encompasses a total of 830 observations and 186 documented instances of sleep bruxism.

The random effects model, optimal for heterogeneous data, suggests a central prevalence estimate of 43.42%, with a 95% confidence interval ranging from 16.65% to 74.68%. This extensive range indicates considerable variation across the studies, likely attributable to differences in population demographics, diagnostic criteria, or methodological approaches.

The prediction interval, spanning from 0.11% to 99.81%, underscores the substantial diversity in sleep bruxism prevalence among different populations as assessed by future studies. This wide interval suggests that while some populations may exhibit almost no occurrence of sleep bruxism, in others, nearly all individuals could be affected, depending on genetic, environmental, and behavioral factors.

Heterogeneity quantification reveals extremely high variability among the included studies (I^2 = 98.5%). The $\tau^2 = 1.81$ and $\tau = 1.35$ further confirm significant dispersion in effect sizes beyond what would be expected by sampling error alone. This is also reflected in the high $H = 8.16$, indicating that

the true effect size varies substantially across the studies, much more than would be expected if the studies were homogeneous.

Both the Wald and LRT for heterogeneity confirm this observation, with $p < 0.001$, indicating that the differences in prevalence estimates across studies are indeed statistically significant and not due to random chance. These tests, therefore, reinforce the presence of high heterogeneity, suggesting that factors unique to each study or population significantly influence the prevalence of sleep bruxism.

The forest plot illustrating the overall effect of the studies on awake bruxism can be reviewed in the file the Supplementary Material S10.

The leave-one-out diagnostics conducted revealed that two of the studies were deemed influential within the context of our analysis.

References

1. Stijnen, T.; Hamza, T.H.; Özdemir, P. Random Effects Meta-Analysis of Event Outcome in the Framework of the Generalized Linear Mixed Model with Applications in Sparse Data. *Stat. Med.* **2010**, *29*, 3046–3067, doi:10.1002/sim.4040.
2. Schwarzer, G.; Chemaitelly, H.; Abu-Raddad, L.J.; Rücker, G. Seriously Misleading Results Using Inverse of Freeman-Tukey Double Arcsine Transformation in Meta-Analysis of Single Proportions. *Res. Synth. Methods* **2019**, *10*, 476–483, doi:10.1002/jrsm.1348.
3. Agresti, A.; Coull, B.A. Approximate Is Better than "Exact" for Interval Estimation of Binomial Proportions. *Am. Stat.* **1998**, *52*, 119–126, doi:10.2307/2685469.
4. Higgins, J.P.T.; Thompson, S.G. Quantifying Heterogeneity in a Meta-analysis. *Stat. Med.* **2002**, *21*, 1539–1558, doi:10.1002/sim.1186.
5. Belsley, D.A.; Kuh, E.; Welsch, R.E. *Regression Diagnostics: Identifying Influential Data and Sources of Collinearity*; Wiley Series in Probability and Statistics; 1st ed.; Wiley, 1980; ISBN 978-0-471-05856-4.
6. Cook, R.D.; Weisberg, S. *Residuals and Influence in Regression*; New York: Chapman and Hall, 1982;
7. Hedges, L.V.; Olkin, I. *Statistical Methods for Meta-Analysis*; 1st edition.; Academic Press: Orlando, 1985; ISBN 978-0-12-336380-0.
8. Viechtbauer, W. Conducting Meta-Analyses in R with the Metafor Package. *J. Stat. Softw.* **2010**, *36*, doi:10.18637/jss.v036.i03.
9. Viechtbauer, W.; Cheung, M.W.-L. Outlier and Influence Diagnostics for Meta-Analysis. *Res. Synth. Methods* **2010**, *1*, 112–125, doi:10.1002/jrsm.11.
10. Schmid, C.H.; Stijnen, T.; White, I.R. *Handbook of Meta-Analysis*; Schmid, C.H., Stijnen, T., White, I., Eds.; 1st ed.; Chapman and Hall/CRC: First edition. | Boca Raton : Taylor and Francis, [2020] | Series: Chapman & Hall/CRC handbooks of modern statistical methods, 2020; ISBN 978-1-315-11940-3.
11. Egger, M.; Smith, G.D.; Schneider, M.; Minder, C. Bias in Meta-Analysis Detected by a Simple, Graphical Test. *BMJ* **1997**, *315*, 629–634, doi:10.1136/bmj.315.7109.629.
12. Balduzzi, S.; Rücker, G.; Schwarzer, G. How to Perform a Meta-Analysis with R: A Practical Tutorial. *Evid. Based Ment. Health* **2019**, *22*, 153–160, doi:10.1136/ebmental-2019-300117.
13. Harrer, M.; Cuijpers, P.; Furukawa, T.; Ebert, D. Dmetar: Companion r Package for the Guide 'Doing Meta-Analysis in r' (R Package Version 0.0.9000) Available online: https://dmetar.protectlab.org/ (accessed on 16 November 2023).
14. Makowski, D.; Lüdecke, D.; Patil, I.; Thériault, R.; Ben-Shachar, M.; Wiernik, B. Automated Results Reporting as a Practical Tool to Improve Reproducibility and Methodological Best Practices Adoption. Available online: URL: https://easystats.github.io/report/ (accessed on 16 November 2023).
15. Sjoberg, D., D.; Whiting, K.; Curry, M.; Lavery, J., A.; Larmarange, J. Reproducible Summary Tables with the Gtsummary Package. *R J.* **2021**, *13*, 570, doi:10.32614/RJ-2021-053.
16. Wickham, H.; François, R.; Henry, L.; Müller, K.; Vaughan, D.; Software, P.; PBC Dplyr: A Grammar of Data Manipulation 2023.
17. Revelle, W. Psych: Procedures for Psychological, Psychometric, and Personality Research 2024.
18. Emodi-Perlman, A.; Eli, I.; Smardz, J.; Uziel, N.; Wieckiewicz, G.; Gilon, E.; Grychowska, N.; Wieckiewicz, M. Temporomandibular Disorders and Bruxism Outbreak as a Possible Factor of Orofacial Pain Worsening during the COVID-19 Pandemic-Concomitant Research in Two Countries. *J. Clin. Med.* **2020**, *9*, 3250, doi:10.3390/jcm9103250.
19. de Holanda, T.A.; Marmitt, L.P.; Cesar, J.A.; Svensson, P.; Boscato, N. Sleep Bruxism in Puerperal Women: Data from a Population-Based Survey. *Matern. Child Health J.* **2023**, *27*, 262–271, doi:10.1007/s10995-022-03576-2.

20. Manfredini, D.; Winocur, E.; Guarda-Nardini, L.; Lobbezoo, F. Self-Reported Bruxism and Temporomandibular Disorders: Findings from Two Specialised Centres. *J. Oral Rehabil.* **2012**, *39*, 319–325, doi:10.1111/j.1365-2842.2011.02281.x.
21. van Selms, M.K.A.; Marpaung, C.; Pogosian, A.; Lobbezoo, F. Geographical Variation of Parental-Reported Sleep Bruxism among Children: Comparison between the Netherlands, Armenia and Indonesia. *Int. Dent. J.* **2019**, *69*, 237–243, doi:10.1111/idj.12450.
22. Serra-Negra, J.M.; Dias, R.B.; Rodrigues, M.J.; Aguiar, S.O.; Auad, S.M.; Pordeus, I.A.; Lombardo, L.; Manfredini, D. Self-Reported Awake Bruxism and Chronotype Profile: A Multicenter Study on Brazilian, Portuguese and Italian Dental Students. *CRANIO®* **2021**, *39*, 113–118, doi:10.1080/08869634.2019.1587854.
23. Tavares Silva, C.; Calabrio, I.R.; Serra-Negra, J.M.; Fonseca-Gonçalves, A.; Maia, L.C. Knowledge of Parents/Guardians about Nocturnal Bruxism in Children and Adolescents. *CRANIO®* **2017**, *35*, 223–227, doi:10.1080/08869634.2016.1201633.
